# Synergistic Silylium‐Ion and Palladium Catalysis Enables Migratory Alkene Silylation With Allylsilanes by Formal C–H/C–Si Metathesis

**DOI:** 10.1002/anie.5617321

**Published:** 2026-09-12

**Authors:** Longhui Duan, Zheng‐Wang Qu, Stefan Grimme, Elisabeth Irran, Hendrik F. T. Klare, Martin Oestreich

**Affiliations:** ^1^ Technische Universität Berlin Institut für Chemie Berlin Germany; ^2^ Rheinische Friedrich‐Wilhelms‐Universität Bonn Mulliken Center for Theoretical Chemistry, Clausius‐Institut für Physikalische und Theoretische Chemie Bonn Germany

**Keywords:** allylsilanes, palladium, proton‐into‐silylium ion conversion, silyl‐Heck reaction, silylium ions

## Abstract

A conceptually distinct approach to the Heck‐type silylation of terminal alkenes to yield allylsilanes under synergistic silylium‐ion and palladium catalysis is reported. The combination of an electrophilic silylium carborate, such as [Me_3_Si(HCB_11_H_5_Br_6_)], and the nucleophilic phosphine‐coordinated palladium(0) complex (*o*‐Tol_3_P)_2_Pd, leads to the formation of the cationic silylpalladium adduct [(o‐Tol_3_P)_2_Pd–SiMe_3_]^+^, which catalyzes the silylation of allylbenzene derivatives using an allylsilane as the stoichiometric silicon electrophile. Mechanistic studies including DFT calculations support a mechanism involving regioselective stepwise ionic addition of the silylpalladium cation across the double bond followed by *β*‐hydride elimination to form a cationic palladium hydride. Exploiting the Brønsted acidity of this intermediate facilitates the regeneration of the active silylpalladium complex by productive proton‐into‐silylium ion interconversion through deallylative protolysis (protodesilylation) of the allylsilane reagent, thereby maintaining the catalytic turnover. Hence, there is no need for an exogeneous base, whereas the conventional silyl‐Heck reaction requires the addition of (over)stoichiometric amounts of a base to trap the formed acid (HX) waste. Moreover, the established silyl‐Heck reaction relies on the oxidative addition of the silicon–halogen bond of a halosilane electrophile as the critical step, while this synergistic process makes use of an allylsilane as the coupling partner, releasing propene as the sole by‐product.

## Graphical abstract

Merging silylium‐ion and palladium catalysis provides a mechanistically distinct route to allylsilanes by formal C–H/Si–H metathesis. Using allylsilanes as silicon proelectrophiles, various allylarenes undergo migrative silylation, selectively yielding (*E*)‐allylsilanes. The catalytically active silylpalladium complex is regenerated by proton‐into‐silylium ion interconversion by protolysis of the allylsilane reagent, producing propene as the sole by‐product.

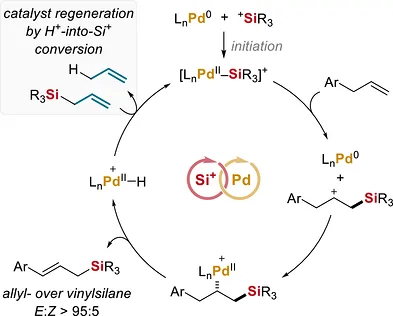

## 1 Introduction

The palladium‐catalyzed silyl‐Heck reaction offers a practical route to synthetically valuable vinyl‐ and allylsilanes starting from simple terminal alkenes [1, 2, 3, 4]. Based on seminal work by the Tanaka group [5], Watson and co‐workers turned this into broadly applicable methodology for carbon–silicon bond formation [6, 7, 8, 9, 10]. The mechanism of this transformation follows the same elementary steps as the classical Mizoroki–Heck reaction by using (pseudo)halosilanes instead of traditional carbon‐based electrophiles (Scheme 1A). Oxidative addition of the silicon–halogen bond [11] in **2** to the low‐valent palladium(0) complex **II** generates the silylpalladium(II) complex **III**, which undergoes *syn*‐silylpalladation [12] (migratory insertion) of the alkene **1** to yield adduct *syn*‐**IV**. A subsequent internal C–C bond rotation (not shown) allows for *syn*‐*β*‐hydride elimination to yield allylsilane **3** or vinylsilane **4** as products along with the hydridopalladium(II) halide **V**. Reductive elimination regenerates the catalytically active palladium(0) complex **II**, eventually closing the catalytic cycle. This last step brings together the proton emerging from the alkene reactant and the halide leaving group of the silicon electrophile in form of HX, which in turn requires the addition of stoichiometric amounts of a sacrificial base (usually Et_3_N).

**SCHEME 1:**
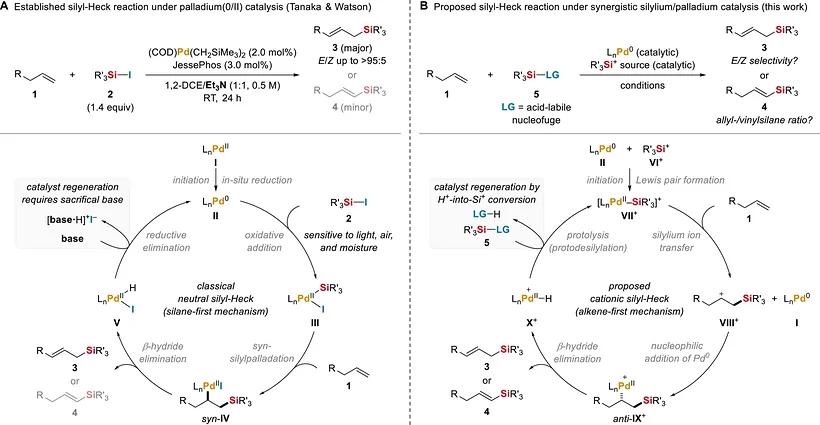
The silyl‐Heck reaction: (A) Watson's protocol under established Pd(0)/Pd(II) redox catalysis versus (B) the design of a cationic version enabled by synergistic silylium‐ion/palladium catalysis and maintained by proton‐into‐silylium interconversion. The counterion in the cationic cycle is omitted for clarity.

Motivated by our research program on silylium ions (R_3_Si^+^) [13, 14] and our strategy of “proton‐into‐silylium ion generation” [15, 16, 17] as a general concept [18, 19] to achieve catalytic transformations with these superelectrophiles [20, 21, 22, 23, 24, 25], we entertained the idea to facilitate this palladium‐catalyzed process by means of a silylium‐ion initiator (Scheme 1B) [26]. Distinct from the above‐described catalytic cycle, the addition of a Lewis acidic silylium source **VI** ^+^ to the Lewis basic palladium(0) catalyst **II** should give rise to a palladium‐stabilized silylium ion, i.e. silylpalladium cation **VII** ^+^ [27]. This Lewis pair formation is equal to a redox process at the metal, generating palladium in the formal oxidation state +II. Precedence comes from the Gagné laboratory, who reported the synthesis of cationic phosphine‐coordinated silylpalladium(II) complexes [L_2_Pd–SiR_3_]^+^X^–^ (L_2_ = XantPhos, 2 Cy_3_P; X^–^ = BAr^F^_4_^–^) [28, 29, 30, 31], which can be regarded as palladium(0) adducts of silylium ions [32]. These complexes with Pd→SiR_3_^+^ *Z*‐type coordination [33, 34] have been demonstrated to maintain electrophilic silylium‐ion and nucleophilic palladium(0) character, capable of activating the polar C(sp^3^)–O [28, 31] and C(sp)–CN [29] bond in ethers and nitriles, respectively. Related cationic N‐heterocyclic carbene‐ligated silylplatinum(II) complexes have also been described [35, 36, 37, 38]. We hypothesized that the cationic silylpalladium(II) complex **VII** ^+^ would be able to transfer the silylium ion to a terminal alkene [39, 40], eventually arriving at the *β*‐silicon‐stabilized carbenium ion **VIII** ^+^, which would then be further stabilized by the reduced palladium(0) complex concomitantly formed by the loss of the silylium ion. Overall, this two‐step process would correspond to an ionic *trans*‐silylpalladation to give the alkylpalladium(II) complex *anti*‐**IX** ^+^. Conventional *syn*‐*β*‐hydride elimination would then lead to the allyl‐ or vinylsilane products **3** or **4** under release of the cationic palladium(II) hydride **X** ^+^. Unlike *neutral* hydridic palladium(II) complexes, we envisioned that this *cationic* intermediate is better described as a protonated palladium complex that is sufficiently Brønsted acidic to promote the protolysis (protodesilylation) of a suitable organosilane **5** decorated with an acid‐labile leaving, nucleofugal group. This controlled “proton‐into‐silylium ion interconversion” **X** ^+^ → **VII** ^+^ would be the key step to secure the catalytic turnover of this cationic Heck‐type reaction.

Herein, we describe the successful experimental realization of this concept. The identification of an effective phosphine ligand to stabilize the cationic silylpalladium(II) catalyst and the use of bench‐stable allylsilanes instead of air‐ and moisture sensitive (pseudo)halosilanes as electrophilic silicon reagents were key to accomplish a base‐free silyl‐Heck‐type reaction of allylbenzene derivatives. This method features excellent selectivity for allylsilanes over vinylsilanes as coupling products and provides access to a variety of allylsilanes with exclusive *E*‐stereoselectivity under release of propene as the sole by‐product.

## 2 Results and Discussion

### 2.1 Reaction Development

Our study commenced with the coupling of allylbenzene (**1a**) and allyltriphenylsilane (**5a**) as the model system (Table 1). We chose the allyl moiety in **5a** (LG = allyl) as an acid‐labile leaving group as we knew from our previous study that protodeallylation is a facile process [15, 19]. In initial experiments, we tested commercially available palladium(0) phosphine complexes as catalysts and silylium carborate [Me_3_Si(HCB_11_H_5_Br_6_)] [41] as the initiator. Whereas conventional (Ph_3_P)_4_Pd failed to promote the reaction (entry 1), product formation was detected with the formal 14‐valence‐electron complex (Cy_3_P)_2_Pd (entry 2), indicating that a reactive low‐valent palladium complex is essential for turnover. Exclusive formation of the allylsilane **3aa** over vinylsilane **4aa** was observed, affording product **3aa** in a moderate yield of 52% and with excellent *E*/*Z* selectivity. Similar to reported silyl‐Heck reactions [6, 8], non‐productive consumption of the starting materials **1a** and **5a** by isomerization to the corresponding alkenes **6a** and **7a** was responsible for the diminished yield (see dashed box). Independent control experiments confirmed that these internal alkenes are unreactive under the reaction conditions (see Section 6.1 in the Supporting Information for details). To minimize these background isomerizations and enhance the reaction rate of the desired transformation, we systematically evaluated different phosphine ligands using the allylsilane **5a** as limiting reagent. We selected the palladium(II) complex (COD)Pd(CH_2_SiMe_3_)_2_ (COD = cycloocta‐1,5‐diene) [42] as the precatalyst, which has been successfully applied in reported silyl‐Heck reactions and is known to readily generate palladium(0) complexes even with sterically demanding phosphines [6, 8]. A series of both mono‐ and bidentate phosphine ligands **L1–L11** was examined (entries 3–11; see Sections 2.2 and 2.3 in the Supporting Information for a complete ligand screening). This survey revealed that the reaction is highly sensitive to the electronic and steric properties of the ligand. In contrast to the established silyl‐Heck reaction (Scheme 1A), which requires an electron‐rich phosphine ligand for the critical oxidative addition of the halosilane [6, 8], the opposite is assumed for the present cationic version (Scheme 1B). An electron‐deficient phosphine ligand should secure productive catalysis by (1) supporting reversible binding of the silylium ion in complex **VII** ^+^ to facilitate its transfer to the alkene **1** (**VII** ^+^ → **VIII** ^+^) and (2) ensuring sufficient Brønsted acidity of the palladium(II) hydride **X** ^+^ to enable turnover through deprotonation by the silicon electrophile **5** (**X** ^+^ → **VII** ^+^). Conversely, an electron‐rich phosphine ligand could inhibit catalysis by generation of a catalytically inactive silylphosphonium salt (*vide infra*). Indeed, changing the *para*‐substituent from hydrogen in parent Ph_3_P (**L1**) to electron‐donating substituents such as methyl in (*p*‐Tol)_3_P (**L2**) resulted in a diminished yield of product **3aa**, whereas the electron‐withdrawing trifluoromethyl group in **L3** led to improved results (entries 3–5). Ultimately, sterically hindered triarylphosphine (*o*‐Tol)_3_P (**L4**), which is characterized by even lower Lewis basicity (LB) and nucleophilicity (*N*) parameters due to the three *ortho*‐methyl substituents [43], was identified as the optimal ligand, furnishing (*E*)‐allylsilane **3aa** in a synthetically useful yield of 78% with excellent selectivity (entry 6). The steric and electronic properties of this ligand also thwart irreversible Lewis adduct formation with the silylium ion. A further increase of the steric demand such as in Mes_3_P (**L5**) was detrimental to the catalytic turnover (entry 7). For comparison, we also probed more electron‐rich monophosphine ligands **L6–L8**, including JessePhos (**L6**), which is the privileged ligand in Watson's silyl‐Heck reaction [8], as well as representative bidentate ligands **L9–L11**. All of these ligands were much less effective or showed no catalytic activity at all (entries 8–11). A ligand‐to‐metal ratio of 2:1 was found to be crucial to ensure the clean formation of the low‐valent, bisligated palladium(0) species and to avoid the presence of free phosphine, which would immediately form a Lewis adduct with the silylium ion, thereby inhibiting product formation (see Section 2.5 in the Supporting Information). Employing preformed complex (*o*‐Tol_3_P)_2_Pd resulted in a slightly lower isolated yield compared to the in‐situ protocol, probably caused by contamination with free phosphine (72% in entry 12 vs 78% in entry 6) [44]. The effect of different reaction conditions was also investigated. Among the various solvents tested, dichloromethane proved to be most effective, while significantly less product formation was detected in aromatic solvents such as benzene, toluene, and 1,2‐dichlorobenzene (see Section 2.4 in the Supporting Information). Lowering the reaction temperature from room temperature to 0°C resulted in partial isomerization of the starting materials and only trace amounts of the target product (not shown). Control experiments confirmed that both the palladium catalyst and silylium‐ion initiator are essential to the reaction (entries 13 and 14). In the absence of [Me_3_Si(HCB_11_H_5_Br_6_)], only isomerization was observed while without palladium no reaction occurred at all, indicating that the metal (likely a palladium hydride intermediate) is responsible for the double‐bond migration. Notably, increasing the catalyst loading to 2.0 mol% palladium and 3.0 mol% silylium ion provided the coupling product in excellent isolated yield of 91% while maintaining flawless allyl/vinyl‐ and *E*/*Z*‐selectivity (entry 15). The catalytic cycle can be also initiated with Reed's benzenium acid [H(C_6_H_6_)][HCB_11_H_5_Br_6_] [45] (assuming the role of Brønsted acidic complex **X** ^+^ in Scheme 1B), albeit with diminished efficiency (entry 16). In stark contrast, conventional triflic acid gave no product (not shown), and replacing [Me_3_Si(HCB_11_H_5_Br_6_)] by arene‐stabilized silylium borate [Et_3_Si(toluene)][B(C_6_F_5_)_4_] was also significantly less efficient, even at a higher catalyst loading of 10 mol% (entry 17), highlighting the vital role of the counterion for catalytic turnover. Finally, we also tested other silicon electrophiles such as tetraphenylsilane (dephenylation), triphenylsilane (dehydrogenation) [39, 46, 47], and triphenyl(vinyl)silane (devinylation) [48, 49, 50, 51] as proton‐into‐silylium ion generators [15, 16, 17], but these did not bring about any improvements (entries 18–20).

**TABLE 1:** Reaction development.^a^

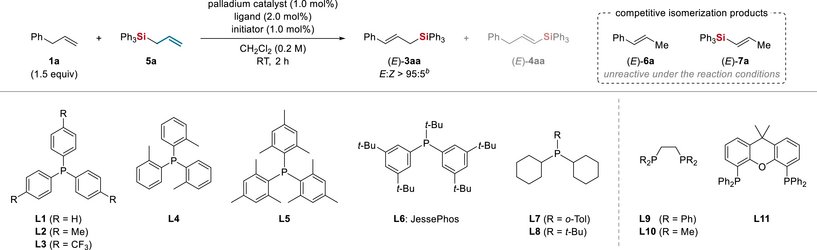						
Entry	Palladium catalyst	Ligand	Catalytic initiator	Silicon electrophile	Yield of 3aa [%]^b^ ^,^ ^c^	3aa:4aa ratio^d^
1	(Ph_3_P)_4_Pd	—	[Me_3_Si(HCB_11_H_5_Br_6_)]	Ph_3_Si–allyl (**5a**)	n.d.	—
2	(Cy_3_P)_2_Pd	—	[Me_3_Si(HCB_11_H_5_Br_6_)]	Ph_3_Si–allyl (**5a**)	52	>95:5
3	(COD)Pd(CH_2_SiMe_3_)_2_	**L1**	[Me_3_Si(HCB_11_H_5_Br_6_)]	Ph_3_Si–allyl (**5a**)	36	>95:5
4	(COD)Pd(CH_2_SiMe_3_)_2_	**L2**	[Me_3_Si(HCB_11_H_5_Br_6_)]	Ph_3_Si–allyl (**5a**)	25	>95:5
5	(COD)Pd(CH_2_SiMe_3_)_2_	**L3**	[Me_3_Si(HCB_11_H_5_Br_6_)]	Ph_3_Si–allyl (**5a**)	45	>95:5
6	(COD)Pd(CH_2_SiMe_3_)_2_	**L4**	[Me_3_Si(HCB_11_H_5_Br_6_)]	Ph_3_Si–allyl (**5a**)	78	>95:5
7	(COD)Pd(CH_2_SiMe_3_)_2_	**L5**	[Me_3_Si(HCB_11_H_5_Br_6_)]	Ph_3_Si–allyl (**5a**)	20	>95:5
8	(COD)Pd(CH_2_SiMe_3_)_2_	**L6**	[Me_3_Si(HCB_11_H_5_Br_6_)]	Ph_3_Si–allyl (**5a**)	n.d.	—
9	(COD)Pd(CH_2_SiMe_3_)_2_	**L7**	[Me_3_Si(HCB_11_H_5_Br_6_)]	Ph_3_Si–allyl (**5a**)	10	>95:5
10	(COD)Pd(CH_2_SiMe_3_)_2_	**L8**	[Me_3_Si(HCB_11_H_5_Br_6_)]	Ph_3_Si–allyl (**5a**)	14	>95:5
11	(COD)Pd(CH_2_SiMe_3_)_2_	**L9–L11**	[Me_3_Si(HCB_11_H_5_Br_6_)]	Ph_3_Si–allyl (**5a**)	n.d.	—
12	(*o*‐Tol_3_P)_2_Pd	—	[Me_3_Si(HCB_11_H_5_Br_6_)]	Ph_3_Si–allyl (**5a**)	72	>95:5
13	None	**L4**	[Me_3_Si(HCB_11_H_5_Br_6_)]	Ph_3_Si–allyl (**5a**)	n.d.	—
14	(COD)Pd(CH_2_SiMe_3_)_2_	**L4**	None	Ph_3_Si–allyl (**5a**)	n.d.	—
15^e^	(COD)Pd(CH_2_SiMe_3_)_2_	**L4**	[Me_3_Si(HCB_11_H_5_Br_6_)]	Ph_3_Si–allyl (**5a**)	91	>95:5
16	(COD)Pd(CH_2_SiMe_3_)_2_	**L4**	[H(C_6_H_6_)][HCB_11_H_5_Br_6_]	Ph_3_Si–allyl (**5a**)	32	>95:5
17	(COD)Pd(CH_2_SiMe_3_)_2_	**L4**	[Et_3_Si(toluene)][B(C_6_F_5_)_4_]	Ph_3_Si–allyl (**5a**)	43	>95:5
18	(COD)Pd(CH_2_SiMe_3_)_2_	**L4**	[Me_3_Si(HCB_11_H_5_Br_6_)]	Ph_3_Si–Ph	n.d.	—
19	(COD)Pd(CH_2_SiMe_3_)_2_	**L4**	[Me_3_Si(HCB_11_H_5_Br_6_)]	Ph_3_Si–H	14	>95:5
20	(COD)Pd(CH_2_SiMe_3_)_2_	**L4**	[Me_3_Si(HCB_11_H_5_Br_6_)]	Ph_3_Si–vinyl	n.d.	—

^a^ Unless otherwise noted, all reactions were performed on a 0.20 mmol scale under an argon atmosphere in CH_2_Cl_2_ (1.0 mL, 0.2 M) at room temperature for 2 h using the indicated amounts (with 1.0 mol% of diphosphine ligands). See the Supporting Information for details and the full optimization study.

^b^ *E*:*Z* > 95:5 in all cases, as estimated by ^1^H NMR spectroscopy of the crude reaction mixture.

^c^ Yields refer to isolated yields after flash column chromatography on silica gel.

^d^ **3aa**:**4aa** ratios determined by ^1^H NMR spectroscopy of the crude reaction mixture.

^e^ (COD)Pd(CH_2_SiMe_3_)_2_ (2.0 mol%), (*o*‐Tol)_3_P (3.0 mol%), and [Me_3_Si(HCB_11_H_5_Br_6_)] (3.0 mol%) were used. n.d. = not detected (neither **3aa** nor **4aa**).

### 2.2 Substrate Scope

With the optimized reaction conditions in hand (Table 1, entry 15), we explored the substrate scope of the migratory alkene silylation (Scheme 2). The model reaction proceeded even more efficiently on a larger scale of 1.0 mmol, affording allylsilane **3aa** in nearly quantitative yield of 96%. We then examined the electronic effect of different substituents at the *para*‐position of the aryl ring in the parent substrate. A similar tolerance toward functional groups was found as typically observed in other catalytic processes under superacidic conditions. Electron‐donating groups such as methyl, *tert*‐butyl and also trimethylsilyl led to the formation of the corresponding allylsilanes **3ba–3da** in high yields (81%–86%). Conversely, electron‐withdrawing substituents such as the trifluoromethyl group or halogens generally showed lower efficacy. While the halogenated products **3fa–3ha** were still obtained in consistent yields of 71%–79% under the standard reaction conditions, the reaction was significantly slower for the CF_3_‐substituted substrate **1e**, requiring an extended reaction time of 12 h to achieve a moderate yield of 48% for the coupling product **3ea**. Likely due to the pronounced electron‐withdrawing effect of the CF_3_ group, the formation of the corresponding vinylsilane **4ea** was detected for the first time, resulting in a lower allyl‐/vinylsilane selectivity of 84:16. The compatibility of the bromine substituent at the aryl group of the alkene substrate **1** **h** is noteworthy, as no competing oxidative addition of the palladium(0) catalyst is observed. Substitution in the *meta*‐position as in **3ia–3ka** and in the *ortho*‐position as in **3la–3na** was also tolerated following the same reactivity trend. However, the formation of the vinylsilane became more pronounced with increasing steric hindrance of the *ortho*‐substituent, as evident for bromine‐substituted substrate **1n**, which was also substantially less reactive. A further increase of the steric bulk as in mesityl‐substituted allylsilane **1o** eventually resulted in a reversed selectivity and the preferential formation of the vinylsilane **4oa** (**3oa**:**4oa** = 30:70). The probable cause for this outcome is that *β*‐hydride elimination from intermediate **IX** ^+^ (see Scheme 1B) is hindered at the benzylic site with increasing steric demand of the aryl substituent. This is where the allyl/vinyl ratio is determined. Substrates with an extended *π*‐system including biphenyl‐ and naphthyl‐substituted **1p–1r** were also converted into the corresponding allylsilanes **3pa–3ra** with comparable yields of 65%–74%. While a lower allyl/vinyl selectivity was observed for **1q** with the sterically more hindered 1‐naphthyl group, the selectivity was again flawless for the 2‐naphthyl derivative **1r**. Heteroaromatic substrates, such as allylthiophenes **1s** and **1t,** were also compatible under the protocol, although only moderate yields were obtained for the corresponding products **3sa** and **3ta**, presumably due to competing Lewis adduct formation with the silylium ion. No reaction occurred with purely aliphatic *α*‐olefins such as 1‐octene (**1u**) and styrene derivatives (not shown). Notably, bis(allyl)silane **5h** underwent twofold deallylative coupling with allylbenzene (**1a**) to furnish bis(cinnamyl)silane **3ah** in 72% isolated yield as a single isomer (see gray box).

**SCHEME 2:**
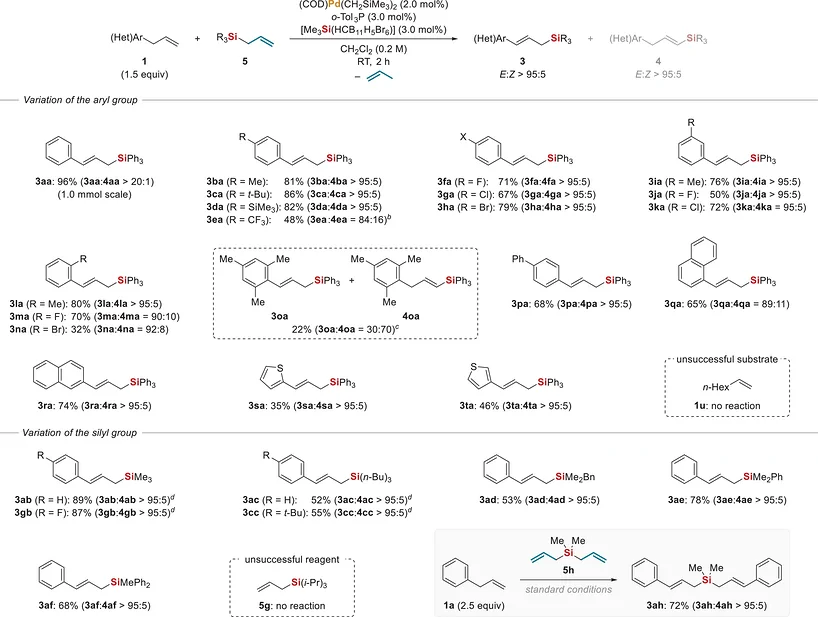
Substrate scope of the migratory alkene silylation. a Unless otherwise noted, all reactions were performed on a 0.20 mmol scale under an argon atmosphere in CH_2_Cl_2_ (1.0 mL, 0.2 M) at room temperature for 2 h using the indicated amounts (see the Supporting Information for details). All yields refer to isolated yields after flash column chromatography on silica gel. The allyl‐/vinylsilane (**3**:**4**) and *E*:*Z* ratios were estimated by ^1^H NMR spectroscopy of the crude reaction mixture. b The reaction time was 12 h. c Combined yield of inseparable allyl‐ and vinylsilane. d Using 0.2 mmol of the corresponding alkene **1** and 0.3 mmol of the corresponding allylsilane **5**.

We next evaluated the effect of different silyl groups in the allylsilane reagent. In addition to allyltriphenylsilane (**5a**), different trialkyl‐ (**5b–d**) as well as dialkyl(aryl)‐ (**5e**) and alkyldi‐aryl‐substituted allylsilanes (**5f**) were tolerated, exclusively affording the corresponding allylsilanes throughout. However, as the steric demand of the silyl group increased, the yields decreased (e.g. 89% for Me_3_Si‐substituted **3ab** versus 52% for *n*‐Bu_3_Si‐substituted **3ac**), and highly bulky allyltriisopropylsilane (**5g**) failed to undergo the desired transformation.

### 2.3 Mechanistic Study

To gain insight into the reaction mechanism, a combined experimental and computational study was conducted. In our initial experiments, we targeted the independent synthesis of the key intermediates to probe their actual role in the individual steps of the catalytic cycle. The cationic silylpalladium(II) complex **9b**, which is the assumed catalytically active species when allyltrimethylsilane (**5b**) is used, was prepared by treatment of palladium(0) complex (*o*‐Tol_3_P)_2_Pd (**8**) [44] with stochiometric amounts of silylium carborate [Me_3_Si(HCB_11_H_5_Br_6_)] [41] in *n*‐hexane at room temperature (Scheme 3, top). Clean silylium transfer from the carborate anion to the palladium complex was verified by multinuclear NMR spectroscopic analysis in CD_2_Cl_2_ at 293 K. A downfield shift from *δ* = –6.8 ppm for (*o*‐Tol_3_P)_2_Pd to *δ* = 24.7 ppm for Pd–Si^+^ complex **9b** was monitored in the ^31^P{^1^H} NMR spectrum. The ^1^H,^31^P‐HMQC experiment revealed weak ^4^ *J*_P,H_ coupling between the protons of the methyl substituents of the silyl group and the phosphorus atom. The detected silicon resonance in the ^1^H,^29^Si‐HMBC spectrum at *δ* = 70.9 ppm is substantially shifted upfield relative to that of the counterion‐stabilized silylium ion [Me_3_Si(HCB_11_H_5_Br_6_)] at *δ* = 93.0 ppm. This pronounced shielding indicates increasing electron donation from the palladium(0) center and the coordinated phosphine ligands, which together act as a stronger stabilizing donor for the silylium ion than the carborate counterion. The ^29^Si NMR chemical shift is in line with the spectroscopic data for the related palladium silylium complex [(Cy_3_P)_2_Pd–SiEtMe_2_]^+^ (*δ*(^29^Si) = 73.2 ppm) reported by Gagné and co‐workers [28]. The successful synthesis of the Pd–Si^+^ complex **9b** required *n*‐hexane as solvent and a prolonged reaction time of 7 days due to the poor solubility. Attempts to generate the palladium silylium adduct under the standard conditions in CD_2_Cl_2_ or aromatic solvents failed, leading to the defined breakdown into silylphosphonium salt **10b**. The molecular structure of this decomposition product was confirmed by its independent synthesis (see Section 5.4 in the Supporting Information) as well as x‐ray diffraction analysis (Scheme 3, top) [52, 53, 54, 55, 56]. The distinct instability of the cationic silylpalladium(II) complex **9b** in solution hampered its crystallographic characterization.

**SCHEME 3:**
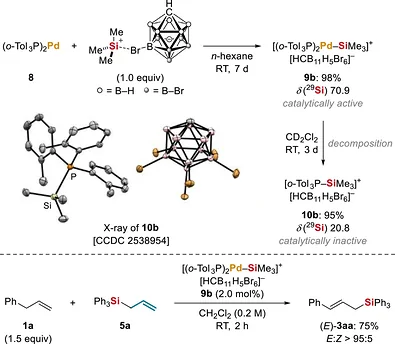
Independent preparation and verification of the catalytic activity of [(o‐Tol_3_P)_2_Pd–SiMe_3_]^+^. Experimental ^29^Si NMR chemical shift determined by ^1^H,^29^Si‐HMQC NMR spectroscopy (500/99 MHz, 298 K, optimized for *J* = 7 Hz) in CD_2_Cl_2_.

We then probed the catalytic activity of the isolated Pd–Si^+^ complex **9b** in the model reaction of allylbenzene (**1a**) and allyltriphenylsilane (**5a**) using 2.0 mol% catalyst loading (Scheme 3, bottom). The desired allylsilane **3aa** was indeed obtained in a slightly lower yield of 75% with unchanged selectivity. Conversely, the silylphosphonium salt **10b** exhibited no reactivity under the standard reaction conditions (not shown). This result, taken together with the control experiments showing that neither the palladium(0) complex nor the silylium ion alone can catalyze this transformation (entries 13 and 14 in Table 1), provides strong evidence that the Pd–SiR_3_^+^ complex is the catalytically active species that maintains the catalytic cycle (i.e. **VII** ^+^ in Scheme 1B).

To gain a deeper understanding of the role of the cationic palladium(II) hydride intermediate (i.e. **X** ^+^ in Scheme 1B) [57], we next turned to its independent synthesis by protonation of the low‐valent palladium(0) complex (*o*‐Tol_3_P)_2_Pd with stoichiometric amounts of the superacidic benzenium salt [H(C_6_H_6_)][HCB_11_H_5_Br_6_] [15, 45] (Scheme 4, top). We found that the presence of small amounts of acetonitrile is essential to stabilize the resulting cationic palladium(II) hydride in form of *trans*‐[L_2_Pd(H)(MeCN)]^+^X^–^ (**11**·MeCN, L = *o*‐Tol_3_P, X = HCB_11_H_5_Br_6_). Without the presence of a Lewis basic donor molecule, rapid decomposition to the corresponding phosphonium salt **12** [52] (*δ*(^31^P) = –12.3 ppm) was monitored by NMR spectroscopy in CD_2_Cl_2_. The ^31^P{^1^H} NMR spectrum of Pd–H^+^ complex **11**·MeCN shows one singlet at *δ* = 17.5 ppm for the pair of chemically equivalent phosphorus atoms. In the ^1^H NMR spectrum, a characteristic triplet is observed for the hydride at *δ* = –14.8 ppm with a ^2^ *J*_H,P_ coupling constant of 12.0 Hz. The spectroscopic data is consistent with previously reported cationic palladium(II) hydride complexes of type [L_2_Pd(H)(MeCN)]^+^ (L = Cy_3_P, *t*‐Bu_3_P) [58, 59]. Unambiguous evidence of the NMR spectroscopic assignment of Pd–H^+^ complex **11**·MeCN was eventually provided by its crystallographic characterization [52] (Scheme 4, top). The molecular structure features a distorted square‐planar coordination geometry with the two phosphine ligands *trans* to each other. The Pd–N distance of 2.110(2) Å is characteristic of complexes in which acetonitrile is *cis* to two phosphines and *trans* to a strongly *trans*‐influencing ligand such as hydride [58, 59, 60]. The Pd–P bond lengths of 2.3146(6) and 2.3206(6) Å are considerably longer than those commonly observed in neutral *trans*‐palladium–phosphine complexes.

**SCHEME 4:**
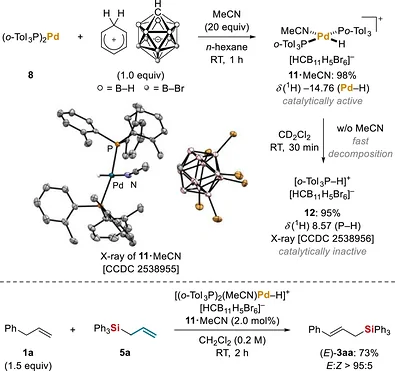
Independent preparation and verification of the catalytic activity of [(*o*‐Tol_3_P)_2_Pd–H]^+^. Experimental ^1^H NMR chemical shifts (500 MHz, 298 K) in CD_2_Cl_2_. The P–H resonance of phosphonium salt **12** appears as a doublet with a ^1^ *J*_H,P_ coupling constant of 486 Hz.

The ability of the Pd–H^+^ complex **11**·MeCN to act as a catalytic initiator for the model reaction was then tested using 2.0 mol% catalyst loading, affording the desired allylsilane **3aa** in 73% yield with again excellent selectivity (Scheme 4, bottom). The phosphonium salt **12**, in turn, showed no catalytic activity under the standard reaction conditions (not shown).

The treatment of Pd–H^+^ complex **11**·MeCN with equimolar amounts of allyltrimethylsilane (**5b**) in the absence of substrate **1** led to almost complete consumption of the allylsilane over 12 h and the release of propylene, as monitored by time‐dependent ^1^H NMR spectroscopy (see Section 6.5 in the Supporting Information). This control experiment indicated the acidic nature of the cationic palladium(II) hydride complex **11**·MeCN, which is sufficiently acidic to promote the protodesilylation of **5b** [61, 62].

### 2.4 DFT Calculations

To further shed light on the mechanism and to distinguish between the proposed operative ionic mechanism and a catalytic cycle that involves the conventional (concerted) organometallic elementary steps, we performed density functional theory (DFT) calculations using allylbenzene (**1a**) and allyltrimethylsilane (**5b**) as model substrates (Scheme 5) [63, 64, 65, 66, 67, 68, 69, 70, 71, 72, 73, 74, 75, 76, 77, 78]. The initiation step, that is, the silylium ion transfer from [Me_3_Si(HCB_11_H_5_Br_6_)] to the low‐valent palladium(0) complex (*o*‐Tol_3_P)_2_Pd (**8**) to form the silylium‐ion‐like complex [(*o*‐Tol_3_P)_2_Pd–SiMe_3_]^+^ (**9b** ^+^), was computed to be exergonic by Δ*G* = –19.4 kcal/mol (not shown). Subsequent silylium ion transfer from **9b** ^+^ to substrate **1a** via **TS1** ^+^ (Δ*G* ^‡^ = +32.4 kcal/mol) leads to the electrophilic carbocation **13ab** ^+^ under regeneration of palladium(0) complex **8**. The nucleophilic palladium(0) center of **8** is capable of transiently stabilizing the electrophilic carbocation **13ab** ^+^ [79, 80, 81], giving rise to carbenium‐ion‐like alkylpalladium(II) intermediate intermediate **14ab** ^+^ that constitutes a viable minimum on the potential energy surface. However, no transition state is found for this Lewis adduct formation. Facile *β*‐hydride elimination of **14ab** ^+^ is –7.0 kcal/mol exergonic over a rather low barrier of 3.6 kcal/mol (via **TS2** ^+^) to give the desired allylsilane product **3ab** along with cationic palladium(II) hydride complex **11** ^+^. The competing formation of the vinylsilane product **4ab** proceeds through exergonic *β‐*hydride elimination of **14ab** ^+^ (Δ*G* = –1.9 kcal/mol) over a higher barrier of 11.0 kcal/mol (via **TS3** ^+^) and is therefore less favorable both kinetically and thermodynamically. The silylium ion transfer from silylpalladium(II) complex **9b** ^+^ to the alkene substrate **1a** is the rate‐limiting step, although the barrier via **TS1** ^+^ seems to be overestimated in our DFT calculations. Hydrometalation of the allylsilane reagent **5b** by palladium(II) hydride complex **11** ^+^ was found to be only mildly endergonic (Δ*G* = 1.8 kcal/mol) over a rather low barrier of Δ*G* ^‡^ = +6.8 kcal/mol via transition state **TS4** ^+^, consistent with a concerted *syn*‐addition process rather than a direct proton transfer from **11** ^+^ to allylsilane **5b** via kinetically less favorable **TS5** ^+^ (Δ*G* ^‡^ = +12.2 kcal/mol). Subsequent palladium(0) dissociation from **1** **4vb** ^+^ followed by silylium ion transfer via **TS7** ^+^ (Δ*G* ^‡^ = +23.3 kcal/mol) regenerates the silylpalladium(II) complex **9b** ^+^, thereby closing the catalytic cycle. The corresponding concerted *β*‐silyl elimination [82] from intermediate **14vb** ^+^ via **TS6** ^+^ to directly arrive at **9b** ^+^ has a substantially larger free energy barrier of Δ*G* ^‡^ = +31.4 kcal/mol, rendering a stepwise ionization–desilylation pathway kinetically more favorable. These results indicate that *β*‐hydride and *β*‐silyl eliminations from palladium(0)‐stabilized carbenium ion intermediates **14** ^+^ proceed through fundamentally distinct mechanisms and that these intermediates play a decisive role in dictating the selectivity of the reaction. The total reaction of allylbenzene (**1a**) and allyltrimethylsilane (**5b**) to yield allylsilane **3ab** is exergonic with a calculated Gibbs free‐energy of –5.2 kcal/mol, whereas the formation of vinylsilane **4ab** is nearly energy neutral. Overall, the DFT calculations and the above control experiments support the proposed catalytic cycle outlined in Scheme 5.

**SCHEME 5:**
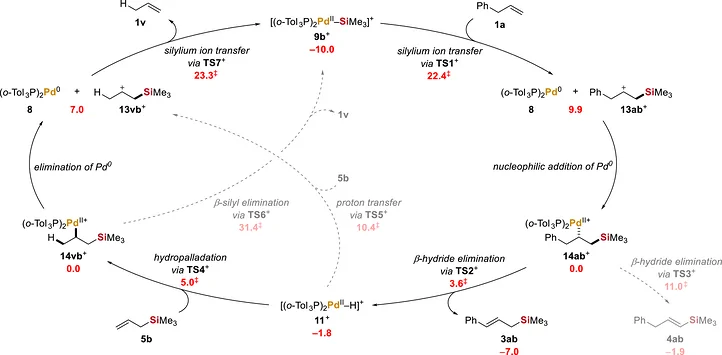
DFT‐computed relative free energies for the proposed catalytic cycle. Gibbs free reaction energies and barriers (labeled with ‡) are given in kcal/mol and were computed at the PW6B95‐D3/def2‐QZVP + COSMO‐RS //TPSS‐D3/def2‐TZVP + COSMO level of theory in CH_2_Cl_2_ solution with **14vb** ^+^ taken as the energy reference. The counterion [HCB_11_H_5_Br_6_]^–^ is omitted for clarity.

## 3 Conclusion

In summary, an ionic silyl‐Heck‐type reaction enabled by synergistic silylium‐ion and palladium catalysis is disclosed. In this process, the formed Brønsted acid–which is neutralized by an exogeneous base in conventional silyl‐Heck reactions–is used to regenerate the catalytically active silylium ion by productive proton‐into‐silylium ion generation using an allylsilane as the electrophilic coupling partner. This depropenative silylation protocol provides access to a broad range of allylsilanes with excellent allyl/vinyl and *Z*/*E* selectivities, only releasing propene as the by‐product. The overall transformation is an example of synergistic main‐group Lewis acid/transition metal participation [83] and can be also classified as a C–H/C–Si (Type 2) metathesis reaction [84]. The present work also includes the independent synthesis, characterization, and catalytic competence of two key intermediates, namely the palladium(II) complexes [(*o*‐Tol_3_P)_2_PdSiMe_3_][HCB_11_H_5_Br_6_] and [(*o*‐Tol_3_P)_2_(MeCN)Pd(H)][HCB_11_H_5_Br_6_], which serve as silylium and proton sources, respectively. These experimental investigations supported by DFT calculations led to the formulation of an ionic mechanism that differs fundamentally from the established silyl‐Heck reaction.

## Author Contributions

**Longhui Duan**: investigation, funding acquisition, writing – original draft, methodology, validation, formal analysis, data curation. **Zheng‐Wang Qu**: investigation, writing – original draft, methodology, validation, formal analysis, data curation. **Stefan Grimme**: writing – review and editing, supervision, resources, funding acquisition. **Elisabeth Irran**: validation, formal analysis. **Hendrik F. T. Klare**: conceptualization, writing – original draft, validation, data curation, formal analysis. **Martin Oestreich**: conceptualization, funding acquisition, writing – original draft, project administration, supervision, resources.

## Conflicts of Interest

The authors declare no conflicts of interest.

## Supporting information



Additional supporting information can be found online in the Supporting Information section. The authors have cited additional references within the Supporting Information [85, 86, 87, 88, 89, 90, 91, 92, 93, 94, 95, 96, 97, 98, 99, 100, 101, 102, 103, 104, 105, 106, 107, 108].**Supporting File**: anie74708‐sup‐0001‐SuppMat.pdf.

## Data Availability

The data that support the findings of this study are available from the corresponding author upon reasonable request.
