# Supplementary material for: Synergistic Silylium‐Ion and Palladium Catalysis Enables Migratory Alkene Silylation With Allylsilanes by Formal C–H/C–Si Metathesis
Source: Angew Chem Int Ed Engl. 2026 Sep 12;65(44):e5617321. doi: 10.1002/anie.5617321 (PMC13703927; doi:10.1002/anie.5617321)
Supplement: Supplementary file 1 [file ANIE-65-e5617321-s001.pdf]

Synergistic Silylium-Ion and Palladium Catalysis Enables Migratory Alkene Silylation with Allylsilanes by Formal C–H/C–Si Metathesis

Longhui Duan,[a] Zheng-Wang Qu,*[b] Stefan Grimme,[b] Elisabeth Irran,[a]
Hendrik F. T. Klare,*[a] and Martin Oestreich*[a]

[a]Institut für Chemie, Technische Universität Berlin, 10623 Berlin, Germany

[b]Mulliken Center for Theoretical Chemistry, Clausius-Institut für Physikalische und Theoretische Chemie, Rheinische Friedrich-Wilhelms-Universität Bonn, 53115 Bonn, Germany

qu@thch.uni-bonn.de

hendrik.klare@tu-berlin.de

martin.oestreich@tu-berlin.de

Supporting Information

Table of Contents

**1**	**General Information**	**S3**
**2**	**Optimization Study**	**S5**
2.1	Evaluation of Palladium Catalysts	S5
2.2	Ligand Screening with Allyltriphenylsilane (**5a**)	S5
2.3	Ligand Screening with Allyltrimethylsilane (**5b**)	S6
2.4	Evaluation of Solvent Effects	S7
2.5	Evaluation of the Palladium/Ligand/Silylium-Ion Ratio	S7
2.6	Effects of Deviations from the Standard Conditions	S8
**3**	**Experimental Details for the Substrate Synthesis**	**S9**
3.1	General Procedure for the Synthesis of Allylarenes **1** (**GP 1**)	S9
3.2	General Procedure for the Synthesis of Allylsilanes **5** (**GP 2**)	S9
3.3	Characterization Data for Allylarenes **1**	S10
3.4	Characterization Data for Allylsilanes **5**	S17
**4**	**Experimental Details for the Cationic Silyl-Heck Reactions under Synergistic Silylium-Ion and Palladium Catalysis**	**S20**
4.1	General Procedure for the Cationic Silyl-Heck Reactions (**GP 3**)	S20
4.2	Characterization Data for Allylsilanes **3**	S20
**5**	**Experimental Details for the Independent Preparation and Characterization of the Key Intermediates**	**S38**
5.1	Preparation of (COD)Pd(CH_2SiMe_3)$_2$	S38
5.2	Preparation of (*o*-Tol$_3$P)$_2$Pd (**8**)	S39
5.3	Preparation of [(*o*-Tol$_3$P)$_2$PdSiMe$_3$][HCB$_{11}$H$_5$Br$_6$] (**9b**)	S39
5.4	Preparation of [*o*-Tol$_3$PSiMe$_3$][HCB$_{11}$H$_5$Br$_6$] (**10b**)	S40
5.5	Preparation of [(*o*-Tol$_3$P)$_2$Pd(H)(MeCN)][HCB$_{11}$H$_5$Br$_6$] (**11**·MeCN)	S41
5.6	Preparation of [*o*-Tol$_3$PH][HCB$_{11}$H$_5$Br$_6$] (**12**)	S42
**6**	**Experimental Mechanistic Study**	**S43**
6.1	Control Experiments on the Isomerization	S43
6.2	Equilibrium Studies on (*o*-Tol$_3$P)$_2$Pd and Free Phosphine	S45
6.3	Catalytic Activity of [(*o*-Tol$_3$P)$_2$PdSiMe$_3$][HCB$_{11}$H$_5$Br$_6$] (**9b**)	S46
6.4	Catalytic Activity of [(*o*-Tol$_3$P)$_2$Pd(H)(MeCN)][HCB$_{11}$H$_5$Br$_6$] (**11**·MeCN)	S46
6.5	Time-Dependent ^{1}H NMR Spectroscopy of the Reaction of Allyltrimethylsilane (**5b**) with [(*o*-Tol$_3$P)$_2$Pd(H)(MeCN)][HCB$_{11}$H$_5$Br$_6$] (**11**·MeCN)	S47
**7**	**Crystallographic Data**	**S48**
**8**	**NMR Spectra**	**S54**
**9**	**Computational Data**	**S179**
**10**	**References**	**S198**

1 General Information

All reactions were performed in flame-dried glassware using an MBraun glovebox (O_2 < 0.5 ppm, H_2O < 1.0 ppm) or conventional Schlenk techniques under a static pressure of argon (glovebox) or nitrogen (fume hood) unless otherwise stated. All given elevated temperatures refer to external oil bath temperatures. Standard solvents and reagents were obtained from commercial suppliers and used as received unless otherwise stated. The glassware was dried overnight at 150 °C or flame dried using a heat gun. Liquids and solutions were transferred with syringes. All plastic syringes and needles used in the glovebox were dried overnight at 60 °C. Technical grade solvents for extraction and chromatography were distilled prior to use. Tetrahydrofuran (THF) was dried over sodium and freshly distilled prior to use. Dry benzene (C_6H_6) and *n*-pentane were obtained from an MBraun solvent purification system (SPS-800), degassed by three freeze-pump-thaw cycles, and stored in a glovebox over thermally activated 4 Å molecular sieves. Dichloromethane (CH_2Cl_2), toluene (C_7H_8), fluorobenzene (C_6H_5F), chlorobenzene (C_6H_5Cl), bromobenzene (C_6H_5Br), 1,2-dichlorobenzene (1,2-$C_6H_4Cl_2$) and acetonitrile (MeCN) were dried over CaH_2, distilled, degassed by three freeze-pump-thaw cycles and stored in a glovebox over thermally activated 4 Å molecular sieves. Dichloro(1,5-cyclooctadiene)palladium(II) ((COD)$PdCl_2$) and tris(*o*-tolyl)phosphine (*o*-Tol_3P) were purchased from BLDpharm, and *o*-Tol_3P was dried at 100 °C under vacuum overnight prior to use. Silylium carborate [$Me_3Si(HCB_{11}H_5Br_6)$] [41, 85–87], Reed's benzenium salt [$H(C_6H_6)$][$HCB_{11}H_5Br_6$] [15, 85], and [Et_3Si(toluene)][$B(C_6F_5)_4$] [88] were synthesized according to reported procedures. Allylsilanes **5b** and **5h** were purchased from BLDpharm, whereas allylsilanes **5a** and **5c**–**g** were synthesized according to reported procedures [89–91]. All liquids were degassed by three freeze-pump-thaw cycles and stored in a glovebox over thermally activated 4 Å molecular sieves. Thin-layer chromatography (TLC) was performed on Macherey-Nagel Alugram® Xtra SIL G/UV254 silica gel 60 pre-coated aluminum-backed plates (200 μm layer thickness). Product spots were visualized under UV light (λ_{max} = 254 nm) and with a ceric ammonium molybdate stain. Column chromatography was performed on Grace 60 silica gel (40–63 μm, 230–400 mesh, ASTM). ^1H, ^{13}C, ^{19}F, ^{29}Si and ^{31}P NMR spectra were recorded in $CDCl_3$, CD_2Cl_2, C_6D_6 or 1,2-$C_6D_4Cl_2$ on a Bruker AV400 and AV500 instrument, respectively. Chemical shifts are reported in parts per million

(ppm) and are referenced to the residual solvent resonance as the internal standard ($CHCl_3$: δ = 7.26 ppm for ^{1}H NMR and $CDCl_3$: δ = 77.16 ppm for ^{13}C NMR; $CDHCl_2$: δ = 5.32 ppm for ^{1}H NMR and CD_2Cl_2: δ = 53.84 ppm for ^{13}C NMR; C_6D_5H: δ = 7.16 ppm for ^{1}H NMR and C_6D_6: δ = 128.39 ppm for ^{13}C NMR; 1,2-$C_6D_3HCl_2$: δ = 6.94 and 7.20 ppm for ^{1}H NMR and 1,2-$C_6D_4Cl_2$: δ = 127.1, 130.1, and 132.5 ppm for ^{13}C NMR). ^{19}F, ^{29}Si and ^{31}P NMR spectra are referenced in compliance with the unified scale for NMR chemical shifts as recommended by the IUPAC stating the chemical shift relative to CCl_3F, Me_4Si and H_3PO_4, respectively. Data are reported as follows: chemical shift, multiplicity (s = singlet, d = doublet, t = triplet, q = quartet, quint = quintet, sept = septet, m = multiplet, br = broad), coupling constants (Hz), and integration. Infrared (IR) spectra were recorded on an Agilent Technologies Cary 630, and the signals are reported in wavenumbers (cm^{-1}). Melting points were determined on a Leica Galen III melting point apparatus and are uncorrected. High resolution mass spectrometry (HRMS) was conducted by the Center for Mass Spectrometry at the Institut für Chemie, Technische Universität Berlin, using an LTQ Orbitrap XL mass analyzer by Thermo Fisher Scientific. Data for the single-crystal structure determination were collected with an Agilent SuperNova diffractometer equipped with a CCD area Atlas detector and a mirror monochromator by utilizing Cu-Kα radiation (λ = 1.5418 Å). The following software packages were used: CrysAlisPro for data collection, cell refinement, and data reduction [92], SHELXS-2018 for structure solution [93], SHELXL-2018 for structure refinement [94], and Mercury for graphics [95].

2 Optimization Study

2.1 Evaluation of Palladium Catalysts[a]

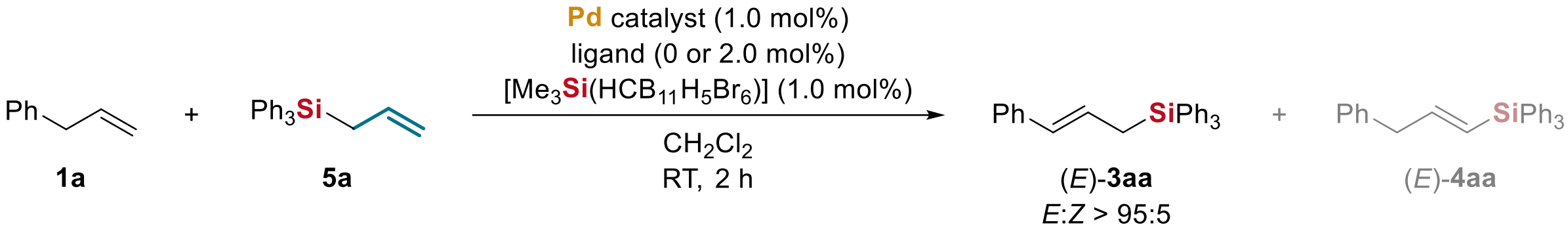

Entry	Palladium complex	Ligand	Yield of **3aa** [%][b]
1	(o-Tol$_3$P)$_2$Pd	–	72
2	(Cy$_3$P)$_2$Pd	–	52
3	(PPh$_3$)$_4$Pd	–	n.d.
4	(dppe)Pd	–	n.d.
5	Pd$_2$(dba)$_2$	Cy$_3$P	n.d.
6	PdCl$_2$	Cy$_3$P	n.d.

[a]Reaction conditions: **1a** (0.30 mmol), **5a** (0.20 mmol), palladium catalyst (1.0 mol%), ligand (2.0 mol%) and [$Me_3Si(HCB_{11}H_5Br_6$] (1.0 mol%) in CH_2Cl_2 (1.0 mL, 0.2 M) at room temperature for 2 h. [b]The yield of **3aa** was determined by ^1H NMR spectroscopy using CH_2Br_2 as an internal standard. **3aa**: **4aa** > 95:5 in all cases as verified by ^1H NMR spectroscopy of the crude reaction mixtures. n.d. = not detected.

2.2 Ligand Screening with Allyltriphenylsilane (5a)[a]

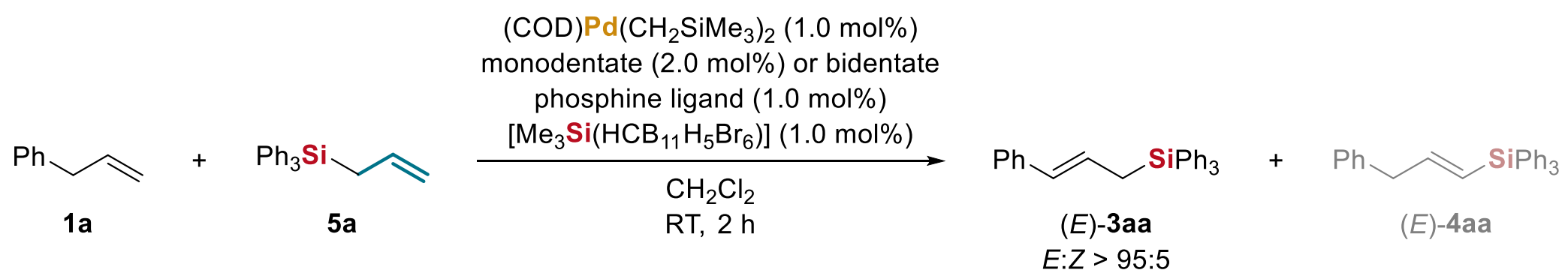

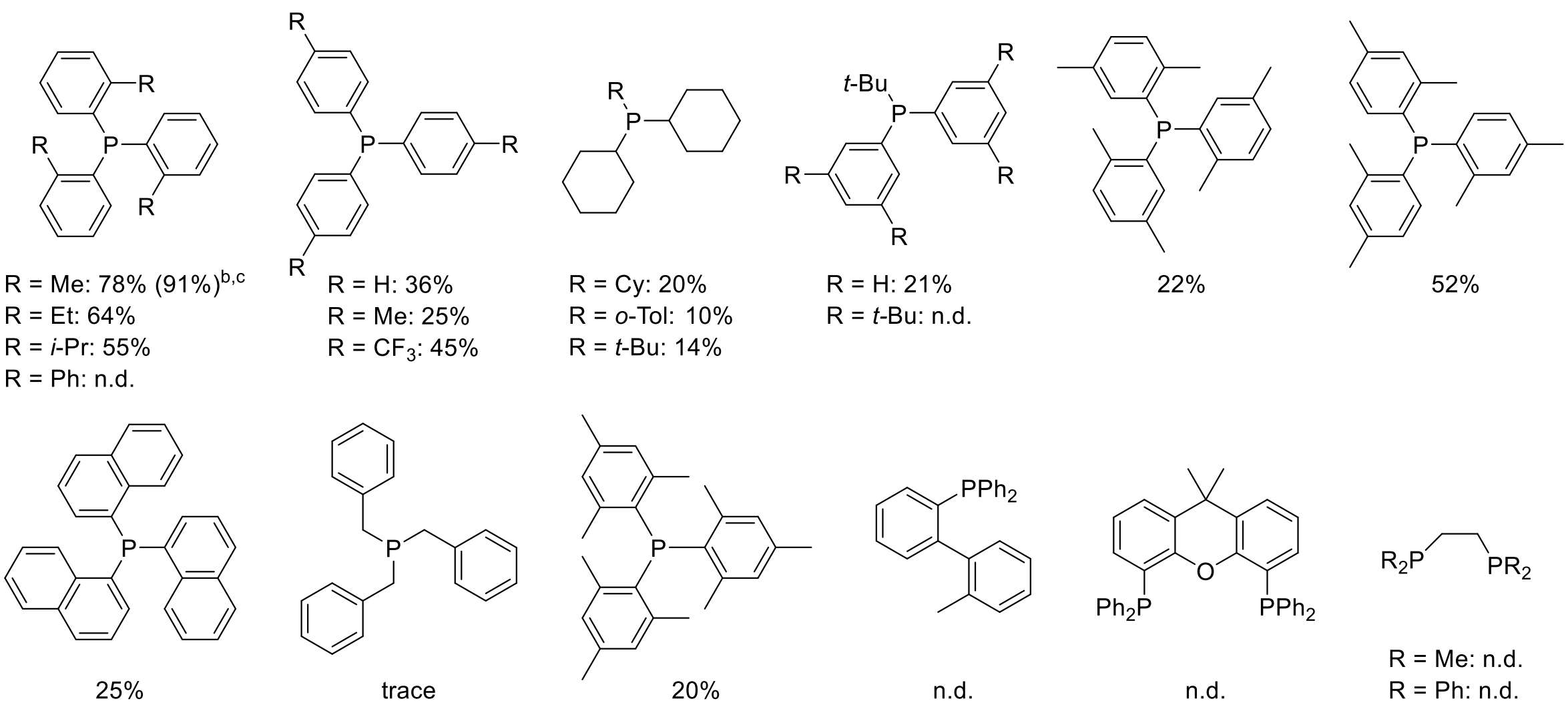

[a]Reaction conditions: **1a** (0.30 mmol), **5a** (0.20 mmol), (COD)Pd(CH_2SiMe_3)$_2$ (1.0 mol%),

monophosphine ligand (2.0 mol%) or bidentate phosphine ligand (1.0 mol%) and $[Me_3Si(HCB_{11}H_5Br_6)]$ (1.0 mol%) in CH_2Cl_2 (1.0 mL, 0.2 M) at room temperature for 2 h. The yield of **3aa** was determined by ^1H NMR spectroscopy using CH_2Br_2 as an internal standard. **3aa**:**4aa** > 95:5 in all cases as verified by ^1H NMR spectroscopy of the crude reaction mixtures. [b]$(COD)Pd(CH_2SiMe_3)_2$ (2.0 mol%), *o*-Tol_3P (3.0 mol%) and $[Me_3Si(HCB_{11}H_5Br_6)]$ (3.0 mol%) were used. [c]Isolated yield after flash column chromatography on silica gel is given in parentheses. n.d. = not detected (neither **3aa** nor **4aa**).

2.3 Ligand Screening with Allyltrimethylsilane (5b)[a]

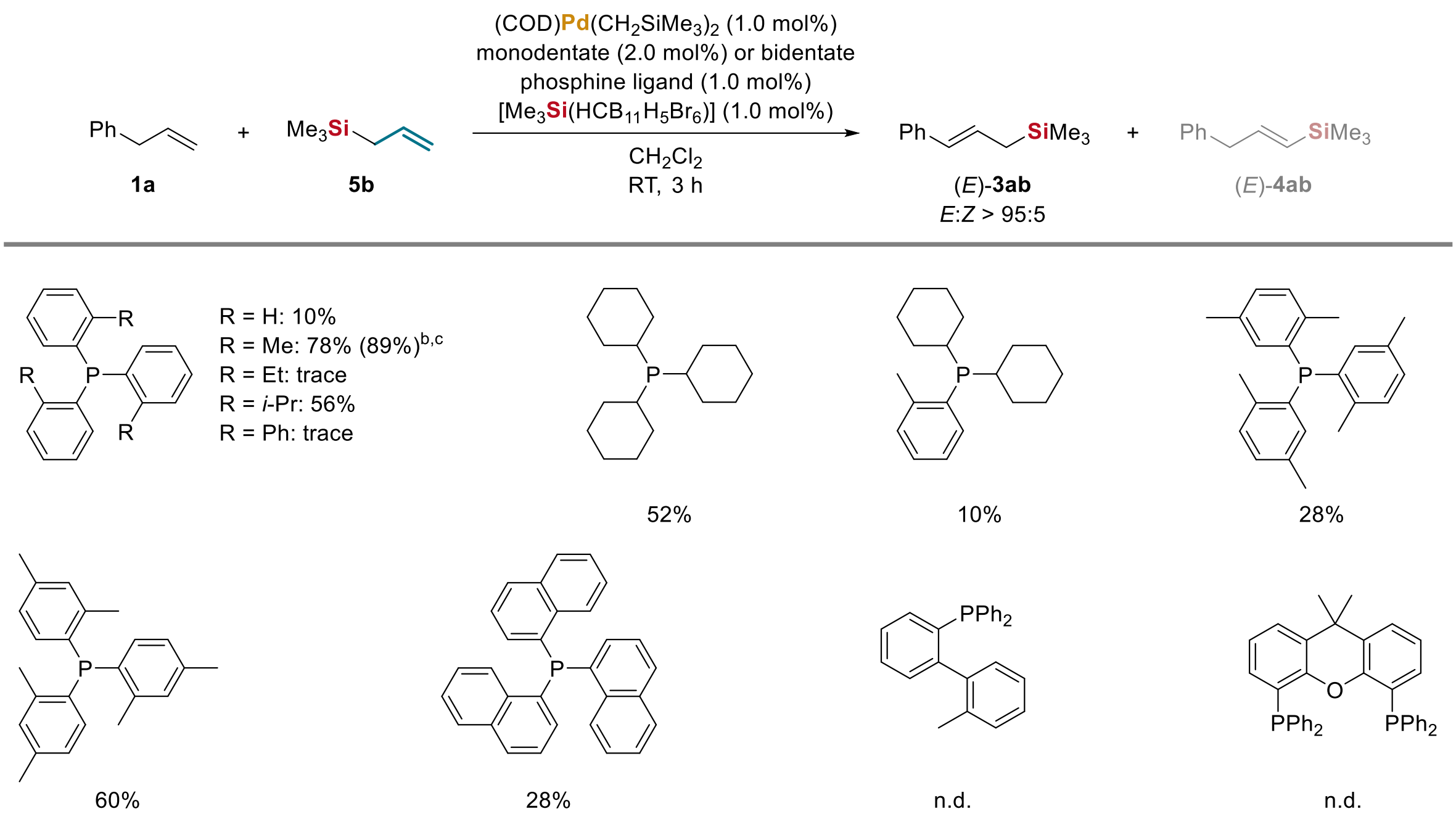

[a]Reaction conditions: **1a** (0.30 mmol), **5b** (0.20 mmol), $(COD)Pd(CH_2SiMe_3)_2$ (1.0 mol%), monophosphine ligand (2.0 mol%) or bidentate phosphine ligand (1.0 mol%) and $[Me_3Si(HCB_{11}H_5Br_6)]$ (1.0 mol%) in CH_2Cl_2 (1.0 mL, 0.2 M) at room temperature for 3 h. The yield of **3aa** was determined by ^1H NMR spectroscopy using CH_2Br_2 as an internal standard. **3aa**:**4aa** > 95:5 in all cases as verified by ^1H NMR spectroscopy of the crude reaction mixtures. [b]**1a** (0.20 mmol), **5b** (0.30 mmol), $(COD)Pd(CH_2SiMe_3)_2$ (2.0 mol%), *o*-Tol_3P (3.0 mol%) and $[Me_3Si(HCB_{11}H_5Br_6)]$ (3.0 mol%) were used. [c]Isolated yield after flash column chromatography on silica gel is given in parentheses. n.d. = not detected (neither **3aa** nor **4aa**).

2.4 Evaluation of Solvent Effects[a]

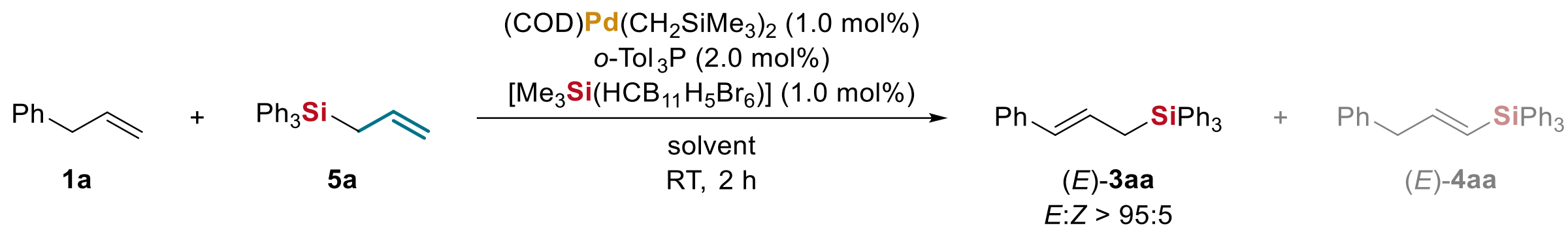

Entry	Solvent	Yield of **3aa** [%][b]
1	CH_2Cl_2	78
2	toluene	30
3	benzene	25
4	1,2-$Cl_2C_6H_4$	53
5	1,2-$F_2C_6H_4$	16

[a]Reaction conditions: **1a** (0.30 mmol), **5a** (0.20 mmol), (COD)Pd(CH_2SiMe_3)$_2$ (1.0 mol%), *o*-Tol_3P (2.0 mol%) and [$Me_3Si(HCB_{11}H_5Br_6)$] (1.0 mol%) in the indicated solvent (1.0 mL, 0.2 M) at room temperature for 2 h. [b]The yield of **3aa** was determined by ^1H NMR spectroscopy using CH_2Br_2 as an internal standard. **3aa**:**4aa** > 95:5 in all cases as verified by ^1H NMR spectroscopy of the crude reaction mixtures.

2.5 Evaluation of the Palladium/Ligand/Silylium-Ion Ratio[a]

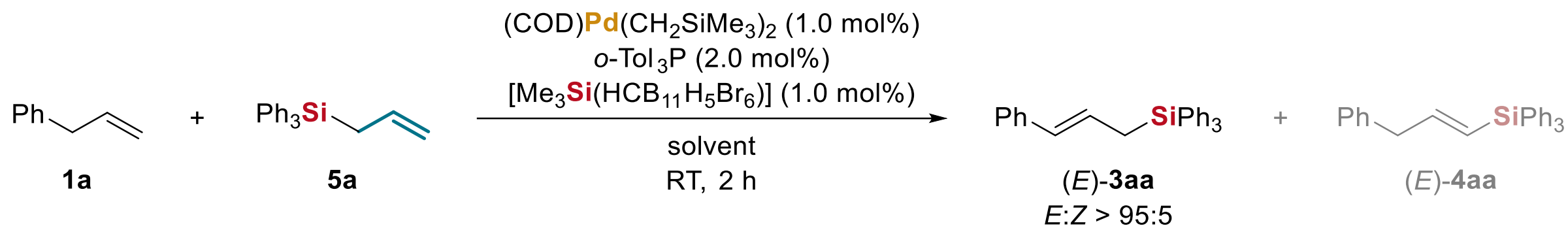

Entry	Palladium complex	Ligand	[$Me_3Si(HCB_{11}H_5Br_6)$]	Yield of **3aa** [%][b]
1	2.0 mol%	2.0 mol%	3.0 mol%	37
2	2.0 mol%	3.0 mol%	3.0 mol%	91
3	2.0 mol%	4.0 mol%	3.0 mol%	64
4	2.0 mol%	3.0 mol%	2.0 mol%	56

[a]Reaction conditions: **1a** (0.30 mmol), **5a** (0.20 mmol), (COD)Pd(CH_2SiMe_3)$_2$ (x mol%), *o*-Tol_3P (y mol%), [$Me_3Si(HCB_{11}H_5Br_6)$] (z mol%) in CH_2Cl_2 (1.0 mL, 0.2 M) at room temperature for 2 h. [b]The yield of **3aa** was determined by ^1H NMR spectroscopy using CH_2Br_2 as an internal standard. **3aa**:**4aa** > 95:5 in all cases as verified by ^1H NMR spectroscopy of the crude reaction mixtures.

2.6 Effects of Deviations from the Standard Conditions[a]

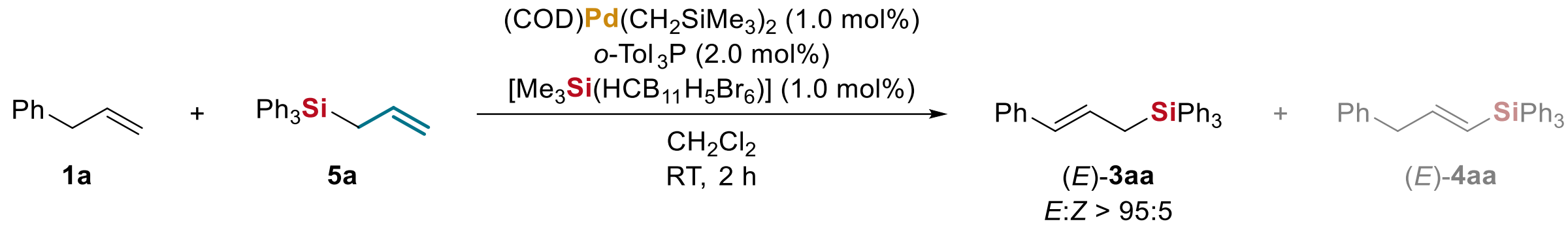

Entry	Deviation from the standard conditions	Yield of **3aa** [%][b]
1	$[H(C_6H_6)][HCB_{11}H_5Br_6]$ instead of $[Me_3Si(HCB_{11}H_5Br_6)]$	32
2	$[Et_3Si(toluene)][B(C_6F_5)_4]$ instead of $[Me_3Si(HCB_{11}H_5Br_6)]$	43
3	TfOH instead of $[Me_3Si(HCB_{11}H_5Br_6)]$	n.d.
4	without $(COD)Pd(CH_2SiMe_3)_2$	n.d.
5	without $[Me_3Si(HCB_{11}H_5Br_6)]$	n.d.

[a]Reaction conditions: **1a** (0.30 mmol), **5a** (0.20 mmol), $(COD)Pd(CH_2SiMe_3)_2$ (1.0 mol%), *o*-Tol_3P (2.0 mol%). For entry 1: $[H(C_6H_6)][HCB_{11}H_5Br_6]$ (2.0 mol%). For entry 2: $[Et_3Si(toluene)][B(C_6F_5)_4]$ (2.0 mol%). For entry 3: HOTf (2.0 mol%). [b]The yield of **3aa** was determined by ^1H NMR spectroscopy using CH_2Br_2 as an internal standard. **3aa**:**4aa** > 95:5 in all cases as verified by ^1H NMR spectroscopy of the crude reaction mixtures. n.d. = not detected (neither **3aa** nor **4aa**).

3 Experimental Details for the Substrate Synthesis

3.1 General Procedure for the Synthesis of Allylarenes 1 (GP 1)

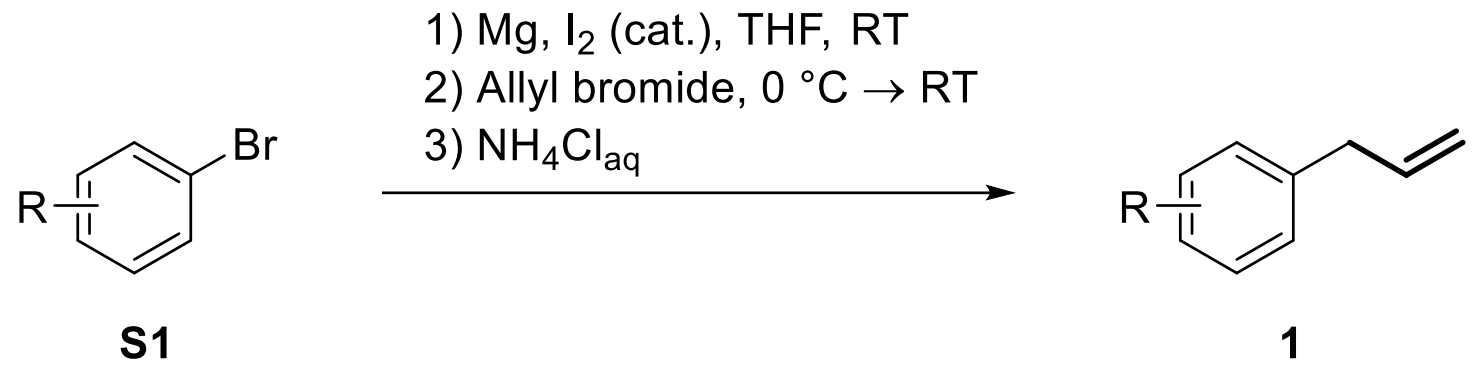

An oven-dried 100-mL round-bottom Schlenk flask equipped with a Teflon-coated magnetic stir bar is charged with magnesium turnings (21.0 mmol, 1.1 equiv) and a catalytic amount of iodine under nitrogen atmosphere. A solution of the corresponding aryl bromide **S1** (20.0 mmol, 1.0 equiv) in anhydrous tetrahydrofuran (40 mL) is added dropwise, and the reaction mixture is refluxed for 2 hours. After completion, the reaction mixture is allowed to cool to room temperature and then cooled to 0 °C using an ice bath. At this temperature, allyl bromide (40.0 mmol, 2.0 equiv) is added dropwise under vigorous stirring. The reaction is stirred until complete conversion is monitored by thin-layer chromatography (TLC). The reaction is then quenched by the careful addition of saturated aqueous NH_4Cl solution (50 mL), and the resulting mixture is extracted with Et_2O (3 × 50 mL). The combined organic layers are dried over anhydrous $MgSO_4$, filtered, and concentrated under reduced pressure. Purification of the residue by flash column chromatography on silica gel using *n*-pentane as the eluent affords the desired allylarene **1** as a colorless oil in analytically pure form.

3.2 General Procedure for the Synthesis of Allylsilanes 5 (GP 2)

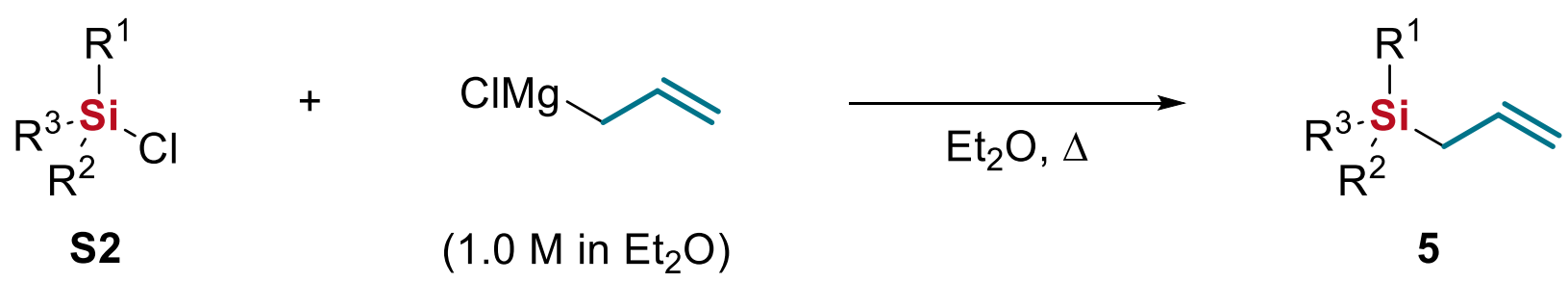

An oven-dried round-bottom Schlenk flask equipped with a magnetic stir bar is charged with a solution of the corresponding chlorosilane **S2** (10 mmol, 1.0 equiv) in anhydrous diethyl ether (15 mL) under nitrogen atmosphere. The solution is cooled to 0 °C using an ice bath, and allylmagnesium bromide (1.0 M in Et_2O, 12 mmol, 1.2 equiv) is added dropwise. After completion, the reaction mixture is allowed to warm to room temperature and stirred overnight. The reaction is then quenched by the careful addition of saturated aqueous NH_4Cl solution, and the resulting mixture is extracted with Et_2O (3 × 20 mL). The combined organic

phases are dried over anhydrous $MgSO_4$, filtered, and concentrated under reduced pressure. Purification of the residue either by flash column chromatography on silica gel using *n*-pentane as eluent or by distillation affords the desired allylsilane **5** as a colorless oil in analytically pure form.

3.3 Characterization Data for Allylarenes 1

Allylbenzene (**1a**) [CAS: 300-57-2]

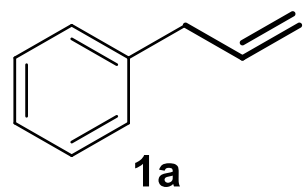

1a
C_9H_{10}
M = 118.18 g/mol

Prepared according to **GP 1**. Flash column chromatography on silica gel using *n*-pentane as the eluent afforded allylbenzene (**1a**) as a colorless oil (90%). **R*f*** = 0.80 (*n*-pentane). **^{1}H NMR** (500 MHz, $CDCl_3$, 298 K): δ 7.30 (t, *J* = 7.6 Hz, 2H), 7.24–7.13 (m, 3H), 6.03–5.90 (m, 1H), 5.17–4.93 (m, 2H), 3.40 (d, *J* = 6.8 Hz, 2H). **^{13}C{^{1}H} NMR** (126 MHz, $CDCl_3$, 298 K): δ 140.2, 137.6, 128.7, 128.6, 126.2, 115.9, 40.4. The NMR spectroscopic data are in accordance with those reported [96].

1-Allyl-4-methylbenzene (**1b**) [CAS: 3333-13-9]

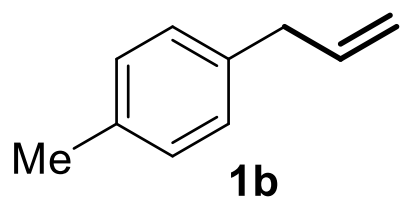

1b
$C_{10}H_{12}$
M = 132.21 g/mol

Prepared according to **GP 1**. Flash column chromatography on silica gel using *n*-pentane as the eluent afforded allylarene **1b** as a colorless oil (86% yield). **R*f*** = 0.75 (*n*-pentane). **^{1}H NMR** (500 MHz, $CDCl_3$, 298 K): δ 7.16–6.95 (m, 4H), 5.96 (m, 1H), 5.19–4.84 (m, 2H), 3.36 (d, *J* = 5.3 Hz, 2H), 2.33 (s, 3H). **^{13}C NMR** (126 MHz, $CDCl_3$, 298 K): δ 137.9, 137.2, 135.7, 129.3, 128.6, 115.7, 40.0, 21.1. The NMR spectroscopic data are in accordance with those reported [97–99].

1-Allyl-4-(*tert*-butyl)benzene (**1c**) [CAS: 27798-45-4]

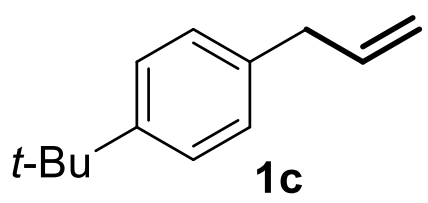

1c
$C_{13}H_{18}$
M = 174.29 g/mol

Prepared according to **GP 1**. Flash column chromatography on silica gel using *n*-pentane as

the eluent afforded allylarene **1c** as a colorless oil (84% yield). **R*f*** = 0.65 (*n*-pentane). **^{1}H NMR** (500 MHz, $CDCl_3$, 298 K): δ 7.37 (d, *J* = 8.3 Hz, 2H), 7.17 (d, J = 7.9 Hz, 2H), 6.02 (dq, *J* = 10.0, 6.8 Hz, 1H), 5.41–4.73 (m, 2H), 3.41 (d, *J* = 6.8 Hz, 2H), 1.36 (s, 9H). **^{13}C NMR** (126 MHz, $CDCl_3$, 298 K): δ 149.1, 137.8, 137.2, 128.4, 125.5, 115.8, 39.9, 34.5, 31.6. The NMR spectroscopic data are in accordance with those reported [97, 99, 100].

(4-Allylphenyl)trimethylsilane (**1d**) [CAS: 18036-85-6]

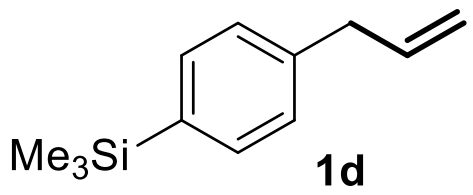

1d

$C_{12}H_{18}Si$
M = 190.36 g/mol

Prepared according to **GP 1**. Flash column chromatography on silica gel using *n*-pentane as the eluent afforded allylarene **1d** as a colorless oil (78% yield). **R*f*** = 0.75 (*n*-pentane). **^{1}H NMR** (500 MHz, $CDCl_3$, 298 K): δ 7.37 (d, *J* = 7.6 Hz, 2H), 7.11 (d, *J* = 7.6 Hz, 2H), 5.88 (ddt, *J* = 16.9, 10.1, 6.8 Hz, 1H), 5.29–4.64 (m, 2H), 3.30 (d, *J* = 6.8 Hz, 2H), 0.17 (s, 9H). **^{13}C NMR** (126 MHz, $CDCl_3$, 298 K): δ 140.9, 137.9, 137.5, 133.7, 128.2, 116.0, 40.4, –0.9. The NMR spectroscopic data are in accordance with those reported [101].

1-Allyl-4-(trifluoromethyl)benzene (**1e**) [CAS: 1813-97-4]

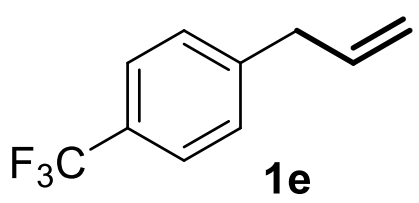

1e

$C_{10}H_9F_3$
M = 186.18 g/mol

Prepared according to **GP 1**. Flash column chromatography on silica gel using *n*-pentane as the eluent afforded allylarene **1e** as a colorless oil (65% yield). **R*f*** = 0.8 (*n*-pentane). **^{1}H NMR** (500 MHz, $CDCl_3$, 298 K): δ 7.55 (d, *J* = 8.0 Hz, 2H), 7.31 (d, *J* = 7.9 Hz, 2H), 5.96 (ddt, *J* = 16.8, 10.3, 6.7 Hz, 1H), 5.33–4.86 (m, 2H), 3.45 (d, *J* = 6.7 Hz, 2H). **^{19}F NMR** (471 MHz, $CDCl_3$, 298 K): δ –62.41. **^{13}C{^{1}H} NMR** (126 MHz, $CDCl_3$, 298 K): δ 144.3, 136.5 (d, *J* = 27.7 Hz), 129.1 (d, *J* = 37.2 Hz), 125.5 (dd, *J* = 34.2, 4.5 Hz), 123.4, 117.2, 116.9, 116.6, 40.1. The NMR spectroscopic data are in accordance with those reported [96, 97].

1-Allyl-4-fluorobenzene (**1f**) [CAS: 1737-16-2]

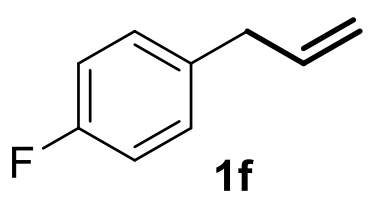

1f

C_9H_9F
M = 136.17 g/mol

Prepared according to **GP 1**. Flash column chromatography on silica gel using *n*-pentane as the eluent afforded allylarene **1f** as a colorless oil (67% yield). **R*f*** = 0.8 (*n*-pentane). **^{1}H NMR** (500 MHz, $CDCl_3$, 298 K): δ 7.18–7.09 (m, 2H), 7.05–6.88 (m, 2H), 6.06–5.86 (m, 1H), 5.13–4.98 (m, 2H), 3.36 (d, *J* = 6.7 Hz, 2H). **^{19}F NMR** (471 MHz, $CDCl_3$, 298 K): δ –117.56. **^{13}C{^{1}H} NMR** (126 MHz, $CDCl_3$, 298 K): δ 161.62 (d, *J* = 244.1 Hz), 137.44, 135.78, 130.13, 130.06, 116.07, 115.37, 115.20, 39.52. The NMR spectroscopic data are in accordance with those reported [97, 102].

1-Allyl-4-chlorobenzene (**1g**) [CAS: 1745-18-2]

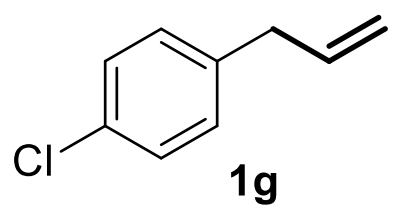

1g
C_9H_9Cl
M = 152.62 g/mol

Prepared according to **GP 1**. Flash column chromatography on silica gel using *n*-pentane as the eluent afforded allylarene **1g** as a colorless oil (75% yield). **R*f*** = 0.8 (*n*-pentane). **^{1}H NMR** (500 MHz, $CDCl_3$, 298 K): δ 7.28–7.14 (m, 3H), 7.07 (d, *J* = 7.2 Hz, 1H), 6.08–5.81 (m, 1H), 5.23–5.02 (m, 2H), 3.37 (d, *J* = 6.7 Hz, 2H). **^{13}C{^{1}H} NMR** (126 MHz, $CDCl_3$, 298 K): δ 142.24, 136.67, 134.37, 129.77, 128.88, 126.92, 126.43, 116.64, 39.96. The NMR spectroscopic data are in accordance with those reported [97, 102].

1-Allyl-4-bromobenzene (**1h**) [CAS: 2294-43-1]

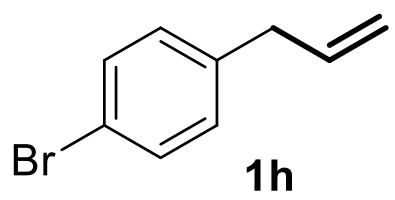

1h
C_9H_9Br
M = 197.08 g/mol

Prepared according to **GP 1**. Flash column chromatography on silica gel using *n*-pentane as the eluent afforded allylarene **1h** as a colorless oil (77% yield). **R*f*** = 0.8 (*n*-pentane). **^{1}H NMR** (500 MHz, $CDCl_3$, 298 K): δ 7.47–7.34 (m, 2H), 7.07 (d, *J* = 8.3 Hz, 2H), 5.93 (ddt, *J* = 16.8, 10.2, 6.7 Hz, 1H), 5.16–4.99 (m, 2H), 3.34 (d, *J* = 6.7 Hz, 2H). **^{13}C{^{1}H} NMR** (126 MHz, $CDCl_3$, 298 K): δ 139.15, 136.92, 133.30, 131.61, 130.51, 120.04, 116.42, 39.71. The NMR spectroscopic data are in accordance with those reported [98, 100, 102].

1-Allyl-3-methylbenzene (**1i**) [CAS: 3333-20-8]

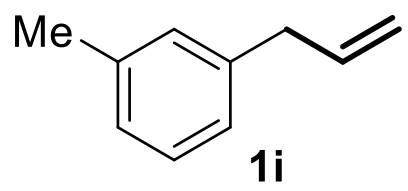

1i
$C_{10}H_{12}$
M = 132.21 g/mol

Prepared according to **GP 1**. Flash column chromatography on silica gel using *n*-pentane as the eluent afforded allylarene **1i** as a colorless oil (84% yield). **R_f** = 0.75 (*n*-pentane). **^{1}H NMR** (500 MHz, $CDCl_3$, 298 K): δ 7.20 (t, *J* = 7.9 Hz, 1H), 7.10–6.92 (m, 3H), 5.99 (ddt, *J* = 16.8, 9.6, 6.6 Hz, 1H), 5.16–5.01 (m, 2H), 3.37 (d, *J* = 6.7 Hz, 2H), 2.35 (s, 3H). **^{13}C NMR** (126 MHz, $CDCl_3$, 298 K): δ 140.2, 138.1, 137.7, 129.5, 128.5, 127.0, 125.7, 115.8, 40.4, 21.5. The NMR spectroscopic data are in accordance with those reported [98].

1-Allyl-3-fluorobenzene (**1j**) [CAS: 30984-53-3]

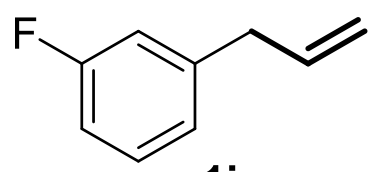

1j
C_9H_9F
M = 136.17 g/mol

Prepared according to **GP 1**. Flash column chromatography on silica gel using *n*-pentane as the eluent afforded allylarene **1j** as a colorless oil (92% yield). **R_f** = 0.80 (*n*-pentane). **^{1}H NMR** (500 MHz, $CDCl_3$, 298 K): δ 7.28 (q, *J* = 7.5 Hz, 1H), 7.00 (d, *J* = 7.7 Hz, 1H), 6.93 (d, *J* = 9.2 Hz, 2H), 6.14–5.84 (m, 1H), 5.29–5.00 (m, 2H), 3.42 (d, *J* = 6.8 Hz, 2H). **^{19}F NMR** (471 MHz, $CDCl_3$, 298 K): δ –113.79 (td, *J* = 9.3, 6.0 Hz). **^{13}C NMR** (126 MHz, $CDCl_3$, 298 K): δ 163.2 (d, *J* = 245.3 Hz), 142.8 (d, *J* = 7.0 Hz), 136.8, 129.9 (d, *J* = 8.3 Hz), 124.4 (d, *J* = 3.4 Hz), 116.5, 115.6 (d, *J* = 21.1 Hz), 113.1 (d, *J* = 21.1 Hz), 40.0. The NMR spectroscopic data are in accordance with those reported [103].

1-Allyl-3-chlorobenzene (**1k**) [CAS: 3840-17-3]

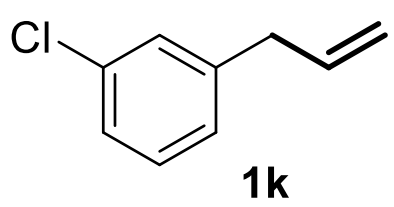

1k
C_9H_9Cl
M = 152.62 g/mol

Prepared according to **GP 1**. Flash column chromatography on silica gel using *n*-pentane as the eluent afforded allylarene **1k** as a colorless oil (54% yield). **R_f** = 0.8 (*n*-pentane). **^{1}H NMR** (500 MHz, $CDCl_3$, 298 K): δ 7.26–7.11 (m, 3H), 7.08 (d, *J* = 7.1 Hz, 1H), 6.09–5.78 (m, 1H), 5.42–4.55 (m, 2H), 3.37 (dd, *J* = 6.7, 1.5 Hz, 2H). **^{13}C NMR** (126 MHz, $CDCl_3$, 298 K): δ

142.2, 136.7, 134.3, 129.8, 128.9, 126.9, 126.4, 116.7, 39.9. The NMR spectroscopic data are in accordance with those reported [102].

1-Allyl-2-methylbenzene (**1l**) [CAS: 1587-04-8]

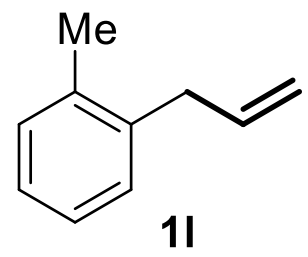

1l
$C_{10}H_{12}$
M = 132.21 g/mol

Prepared according to **GP 1**. Flash column chromatography on silica gel using *n*-pentane as the eluent afforded allylarene **1l** as a colorless oil (85% yield). **R*f*** = 0.75 (*n*-pentane). **^{1}H NMR** (500 MHz, $CDCl_3$, 298 K): δ 7.25–7.02 (m, 4H), 5.97 (ddt, *J* = 16.6, 10.0, 6.3 Hz, 1H), 5.07 (d, *J* = 9.9 Hz, 1H), 5.01 (d, *J* = 16.0 Hz, 1H), 3.39 (d, *J* = 6.3 Hz, 2H), 2.31 (s, 3H). **^{13}C NMR** (126 MHz, $CDCl_3$, 298 K): δ 138.3, 136.8, 136.5, 130.3, 129.3, 126.4, 126.2, 115.8, 37.9, 19.5. The NMR spectroscopic data are in accordance with those reported [98].

1-Allyl-2-fluorobenzene (**1m**) [CAS: 56314-65-9]

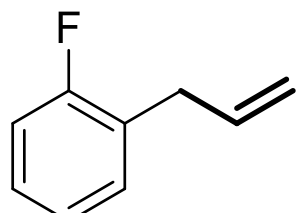

1m
C_9H_9F
M = 136.17 g/mol

Prepared according to **GP 1**. Flash column chromatography on silica gel using *n*-pentane as the eluent afforded allylarene **1m** as a colorless oil (84% yield). **R*f*** = 0.80 (*n*-pentane). **^{1}H NMR** (500 MHz, $CDCl_3$, 298 K): δ 7.25–7.15 (m, 2H), 7.08 (td, *J* = 7.4, 1.3 Hz, 1H), 7.03 (m, 1H), 5.98 (ddt, *J* = 17.9, 9.5, 6.5 Hz, 1H), 5.34–4.77 (m, 2H), 3.43 (d, *J* = 6.5 Hz, 2H). **^{19}F NMR** (471 MHz, $CDCl_3$, 298 K): δ –118.65 (dt, *J* = 12.7, 6.4 Hz). **^{13}C{^{1}H} NMR** (126 MHz, $CDCl_3$, 298 K): δ 161.2 (d, *J* = 245.2 Hz), 136.0, 130.8 (d, *J* = 4.7 Hz), 128.0 (d, *J* = 7.4 Hz), 127.1 (d, *J* = 15.7 Hz), 124.1 (d, *J* = 3.5 Hz), 116.3, 115.4 (d, *J* = 22.2 Hz), 33.2. The NMR spectroscopic data are in accordance with those reported [103].

1-Allyl-2-bromobenzene (**1n**) [CAS: 42918-20-7]

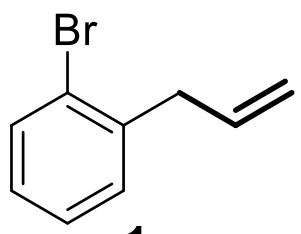

1n
C_9H_9Cl
M = 152.62 g/mol

Prepared according to **GP 1**. Flash column chromatography on silica gel using *n*-pentane as the eluent afforded allylarene **1n** as a colorless oil (69% yield). **R*f*** = 0.75 (*n*-pentane). **^{1}H NMR** (500 MHz, $CDCl_3$, 298 K): δ 7.54 (d, *J* = 8.0 Hz, 1H), 7.29–7.17 (m, 2H), 7.13–6.96 (m, 1H), 5.97 (ddt, *J* = 16.8, 10.0, 6.5 Hz, 1H), 5.12 (d, *J* = 10.1 Hz, 1H), 5.08 (d, *J* = 17.0 Hz, 2H), 3.51 (d, *J* = 6.5 Hz, 2H). **^{13}C NMR** (126 MHz, $CDCl_3$, 298 K): δ 139.6, 135.7, 132.9, 130.6, 128.0, 127.6, 124.7, 116.7, 40.4. The NMR spectroscopic data are in accordance with those reported [98, 102].

2-Allyl-1,3,5-trimethylbenzene (**1o**) [CAS: 4810-05-3]

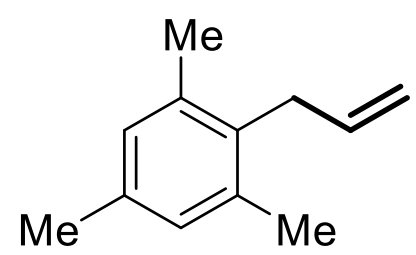

1o
$C_{12}H_{16}$
M = 160.26 g/mol

Prepared according to **GP 1**. Flash column chromatography on silica gel using *n*-pentane as the eluent afforded allylarene **1o** as a colorless oil (78% yield). **R*f*** = 0.80 (*n*-pentane). **^{1}H NMR** (500 MHz, $CDCl_3$, 298 K): δ 6.86 (s, 2H), 5.90 (ddt, *J* = 17.4, 10.4, 5.7 Hz, 1H), 4.99 (dt, *J* = 10.1, 1.9 Hz, 1H), 4.88 (dq, *J* = 17.0, 1.9 Hz, 1H), 3.37 (dd, *J* = 5.8, 1.9 Hz, 2H), 2.27 (s, 9H). **^{13}C{^{1}H} NMR** (126 MHz, $CDCl_3$, 298 K): δ 136.7, 135.7, 135.5, 133.2, 128.9, 114.8, 33.5, 20.1, 19.9. The NMR spectroscopic data are in accordance with those reported [98, 104].

4-Allyl-1,1'-biphenyl (**1p**) [CAS: 20120-35-8]

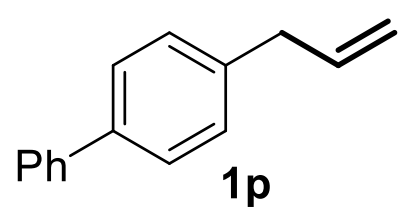

1p
$C_{15}H_{14}$
M = 194.28 g/mol

Prepared according to **GP 1**. Flash column chromatography on silica gel using *n*-pentane as the eluent afforded allylarene **1p** as a colorless oil (78% yield). **R*f*** = 0.45 (*n*-pentane). **^{1}H NMR** (500 MHz, $CDCl_3$, 298 K): δ 7.59 (d, *J* = 7.3 Hz, 2H), 7.54 (d, *J* = 7.8 Hz, 2H), 7.44 (t, *J* = 7.7 Hz, 2H), 7.34 (t, *J* = 7.4 Hz, 1H), 7.28 (d, *J* = 7.9 Hz, 2H), 6.02 (ddt, *J* = 16.8, 10.1, 6.7 Hz, 1H), 5.61–4.65 (m, 2H), 3.45 (d, *J* = 6.8 Hz, 2H). **^{13}C{^{1}H} NMR** (126 MHz, $CDCl_3$, 298 K): δ 141.2, 139.3, 139.2, 137.4, 129.1, 128.8, 127.3, 127.2, 127.1, 116.1, 40.0. The NMR spectroscopic data are in accordance with those reported [99, 100].

1-Allylnaphthalene (**1q**) [CAS: 2489-86-3]

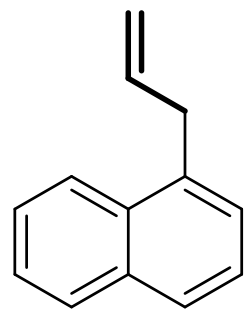

1q
$C_{13}H_{12}$
M = 168.24 g/mol

Prepared according to **GP 1**. Flash column chromatography on silica gel using *n*-pentane as the eluent afforded allylarene **1q** as a colorless oil (68% yield). **R*f*** = 0.45 (*n*-pentane). ^{1}H NMR (500 MHz, $CDCl_3$, 298 K): δ 8.05 (d, *J* = 8.2 Hz, 1H), 7.88 (dd, *J* = 7.7, 1.7 Hz, 1H), 7.76 (d, *J* = 8.2 Hz, 1H), 7.56–7.46 (m, 2H), 7.46–7.40 (m, 1H), 7.37 (d, *J* = 6.9 Hz, 1H), 6.14 (ddt, *J* = 16.7, 10.4, 6.3 Hz, 1H), 5.28–4.76 (m, 2H), 3.87 (d, *J* = 6.1 Hz, 2H). **^{13}C{^{1}H} NMR** (126 MHz, $CDCl_3$, 298 K): δ 137.1, 136.3, 134.0, 132.2, 128.8, 127.1, 126.4, 126.0, 125.8, 125.7, 124.2, 116.3, 37.4. The NMR spectroscopic data are in accordance with those reported [98, 102].

2-Allylnaphthalene (**1r**) [CAS: 2489-87-4]

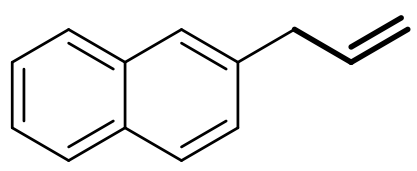

1r
$C_{13}H_{12}$
M = 168.24 g/mol

Prepared according to **GP 1**. Flash column chromatography on silica gel using *n*-pentane as the eluent afforded allylarene **1r** as a colorless oil (72% yield). **R*f*** = 0.45 (*n*-pentane). **^{1}H NMR** (500 MHz, $CDCl_3$, 298 K): δ 7.93–7.78 (m, 3H), 7.67 (s, 1H), 7.54–7.40 (m, 2H), 7.37 (d, *J* = 8.4 Hz, 1H), 6.10 (ddt, *J* = 16.9, 10.1, 6.7 Hz, 1H), 5.49–5.01 (m, 2H), 3.59 (d, *J* = 6.7 Hz, 2H). **^{13}C NMR** (126 MHz, $CDCl_3$, 298 K): δ 137.7, 137.5, 133.8, 132.3, 128.1, 127.8, 127.6, 127.5, 126.8, 126.1, 125.4, 116.2, 40.5. The NMR spectroscopic data are in accordance with those reported [100].

2-Allylthiophene (**1s**) [CAS: 20849-87-0]

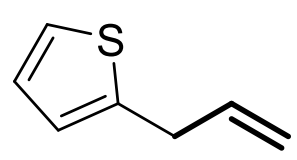

1s
C_7H_8S
M = 124.20 g/mol

Prepared according to **GP 1**. Flash column chromatography on silica gel using *n*-pentane as the eluent afforded allylarene **1s** as a colorless oil (35% yield). **R*f*** = 0.80 (*n*-pentane, $KMnO_4$).

^{1}H NMR (500 MHz, $CDCl_3$, 298 K): δ 7.33–7.00 (m, 1H), 6.94 (dd, *J* = 5.1, 3.4 Hz, 1H), 6.81 (dt, *J* = 3.5, 1.2 Hz, 1H), 6.00 (ddt, *J* = 16.7, 10.0, 6.6 Hz, 1H), 5.22–5.02 (m, 2H), 3.59 (dd, *J* = 6.7, 1.4 Hz, 2H). **^{13}C{^{1}H} NMR** (126 MHz, $CDCl_3$, 298 K): δ 143.1, 136.7, 127.0, 124.7, 123.7, 116.3, 34.3. The NMR spectroscopic data are in accordance with those reported [103].

3-Allylthiophene (**1t**) [CAS: 33934-92-8]

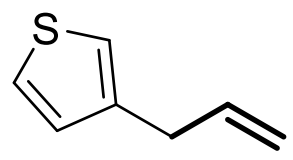

1t
C_7H_8S
M = 124.20 g/mol

Prepared according to a reported procedure with slight modifications [105]. A solution of 3-bromothiophene (1.87 mL, 20.0 mmol, 1.0 equiv) in Et_2O (40.0 mL) was cooled to –78 °C under a nitrogen atmosphere, followed by dropwise addition of *n*-BuLi (8.4 mL, 2.5 M solution in *n*-hexane, 21.0 mmol, 1.05 equiv). The mixture was stirred for additional 1 h. Allyl bromide was then added dropwise under vigorous stirring, and the reaction mixture was stirred until complete conversion was monitored by thin-layer chromatography (TLC). The reaction was quenched by the careful addition of saturated aqueous NH_4Cl solution (50 mL), and the obtained mixture was extracted with Et_2O (3 × 50 mL). The combined organic layers were dried over anhydrous $MgSO_4$, filtered, and concentrated under reduced pressure. Purification of the residue by flash column chromatography on silica gel using *n*-pentane as the eluent afforded allylarene **1t** as a colorless oil in analytically pure form (62% yield). **R_f** = 0.80 (*n*-pentane, $KMnO_4$). **^{1}H NMR** (500 MHz, $CDCl_3$, 298 K): δ 7.29 (dd, *J* = 5.0, 3.0 Hz, 1H), 7.00 (s, 1H), 6.97 (d, *J* = 5.0 Hz, 1H), 6.02 (ddt, *J* = 16.8, 9.9, 6.6 Hz, 1H), 5.38–4.38 (m, 2H), 3.43 (d, *J* = 6.7 Hz, 2H). **^{13}C NMR** (126 MHz, $CDCl_3$, 298 K): δ 140.6, 136.9, 128.4, 125.6, 120.8, 115.9, 34.8. The NMR spectroscopic data are in accordance with those reported [103].

3.4 Characterization Data for Allylsilanes 5

Allyltriphenylsilane (**5a**)

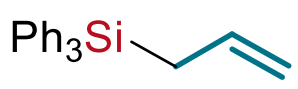

5a
$C_{21}H_{20}Si$
M = 300.48 g/mol

Prepared according to **GP 2**. The crude solid was purified by crystallization from *n*-pentane to

afford allylsilane **5a** as a white crystalline solid (92% yield). **R*f*** = 0.40 (*n*-pentane). **^{1}H NMR** (500 MHz, $CDCl_3$, 298 K): δ 7.61–7.59 (m, 6H), 7.48–7.40 (m, 9H), 5.94 (ddt, *J* = 17.0, 10.1, 7.8 Hz, 1H), 5.56–4.61 (m, 2H), 2.47 (d, *J* = 7.9 Hz, 2H). **^{13}C{^{1}H} NMR** (126 MHz, $CDCl_3$, 298 K): δ 135.9, 134.7, 133.9, 129.7, 128.0, 115.2, 21.4. **^{1}H/^{29}Si HMQC NMR** (500/99 MHz, $CDCl_3$, 298 K, optimized for *J* = 7 Hz): δ 7.62/–13.48, 2.48/–13.48.

Allyltributylsilane (**5c**)

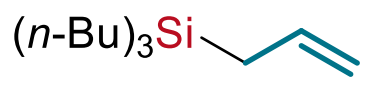

5c

$C_{15}H_{32}Si$

M = 240.51 g/mol

Prepared according to **GP 2**. Flash chromatography on silica gel using *n*-pentane afforded allylsilane **5c** as a colorless oil (85% yield). **R*f*** = 0.80 (*n*-pentane, $KMnO_4$). **^{1}H NMR** (500 MHz, $CDCl_3$, 298 K): δ 5.79 (ddt, *J* = 16.5, 10.1, 8.1 Hz, 1H), 5.28–4.61 (m, 2H), 1.54 (d, *J* = 8.2 Hz, 2H), 1.48–1.03 (m, 12H), 0.89 (t, *J* = 7.0 Hz, 9H), 0.72–0.23 (m, 6H). **^{13}C{^{1}H} NMR** (126 MHz, $CDCl_3$, 298 K): δ 135.7, 112.6, 31.8, 26.9, 26.2, 22.8, 20.7, 14.3, 13.9, 12.1. **^{1}H/^{29}Si HMQC NMR** (500/99 MHz, $CDCl_3$, 298 K, optimized for *J* = 7 Hz): δ 1.58/1.76, 0.55/1.76.

Allyl(benzyl)dimethylsilane (**5d**)

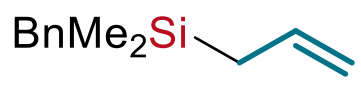

5d

$C_{12}H_{18}Si$

M = 190.36 g/mol

Prepared according to **GP 2**. Flash column chromatography on silica gel using *n*-pentane as the eluent afforded allylsilane **5d** as a colorless oil (84% yield). **R*f*** = 0.60 (*n*-pentane). **^{1}H NMR** (500 MHz, $CDCl_3$, 298 K): δ 7.21 (t, *J* = 7.7 Hz, 2H), 7.07 (t, *J* = 7.4 Hz, 1H), 7.00 (d, *J* = 7.5 Hz, 2H), 5.77 (ddt, *J* = 16.2, 10.5, 8.2 Hz, 1H), 5.16–4.44 (m, 2H), 2.11 (s, 2H), 1.52 (dt, *J* = 8.0, 1.2 Hz, 2H), –0.01 (s, 6H). **^{13}C{^{1}H} NMR** (126 MHz, $CDCl_3$, 298 K): δ 140.2, 134.9, 128.4, 128.3, 124.2, 113.4, 25.3, 22.9, –3.8. **^{1}H/^{29}Si HMQC NMR** (500/99 MHz, $CDCl_3$, 298 K, optimized for *J* = 7 Hz): δ 2.11/0.81, 1.52/0.81, –0.01/0.81.

Allyldimethyl(phenyl)silane (**5e**)

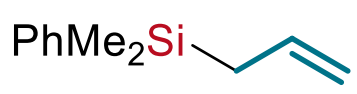

5e

$C_{11}H_{16}Si$

M = 176.33 g/mol

Prepared according to **GP 2**. Flash column chromatography on silica gel using *n*-pentane as the eluent afforded allylsilane **5e** as a colorless oil (82% yield). **R*f*** = 0.50 (*n*-pentane). **^{1}H NMR** (500 MHz, $CDCl_3$, 298 K): δ 7.56–7.48 (m, 2H), 7.41–7.33 (m, 3H), 5.78 (ddt, *J* = 16.9, 10.2, 8.1 Hz, 1H), 4.89–4.84 (m, 2H), 1.76 (dt, *J* = 8.0, 1.2 Hz, 2H), 0.29 (s, 6H). **^{13}C{^{1}H} NMR** (126 MHz, $CDCl_3$, 298 K): δ 138.9, 134.8, 133.8, 129.1, 127.9, 113.5, 23.8, –3.3. **^{1}H/^{29}Si HMQC NMR** (500/99 MHz, $CDCl_3$, 298 K, optimized for *J* = 7 Hz): δ 1.76/–4.99, 0.26/–4.99.

Allyl(methyl)diphenylsilane (**5f**)

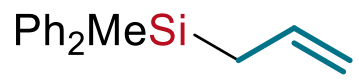

5f

$C_{16}H_{18}Si$

M = 238.41 g/mol

Prepared according to **GP 2**. Flash column chromatography on silica gel using *n*-pentane as the eluent afforded allylsilane **5f** as a colorless oil (65% yield). **R*f*** = 0.25 (*n*-pentane). **^{1}H NMR** (500 MHz, $CDCl_3$, 298 K): δ 7.53 (dd, *J* = 7.6, 1.7 Hz, 4H), 7.44–7.29 (m, 6H), 6.06–5.52 (m, 1H), 5.27–4.57 (m, 2H), 2.09 (dd, *J* = 7.9, 1.2 Hz, 2H), 0.57 (d, *J* = 1.3 Hz, 3H). **^{13}C{^{1}H} NMR** (126 MHz, $CDCl_3$, 298 K): δ 136.8, 134.7, 134.2, 129.4, 128.0, 114.4, 22.3, –4.6. **^{1}H/^{29}Si HMQC NMR** (500/99 MHz, $CDCl_3$, 298 K, optimized for *J* = 7 Hz): δ 7.52/–9.87, 2.09/–9.87, 0.57/–9.87.

Allyltriisopropylsilane (**5g**)

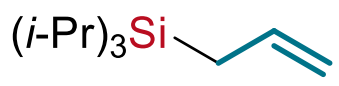

5g

$C_{12}H_{26}Si$

M = 198.43 g/mol

Prepared according to **GP 2**. Flash column chromatography on silica gel using *n*-pentane as the eluent afforded allylsilane **5g** as a colorless oil (86% yield). **R*f*** = 0.80 (*n*-pentane, $KMnO_4$). **^{1}H NMR** (500 MHz, $CDCl_3$, 298 K): δ 6.30–5.62 (m, 1H), 4.92 (d, *J* = 16.9 Hz, 1H), 4.81 (d, *J* = 10.7 Hz, 1H), 1.64 (d, *J* = 7.3 Hz, 2H), 1.06–0.97 (m, 21H). **^{13}C{^{1}H} NMR** (126 MHz, $CDCl_3$, 298 K): δ 136.4, 112.9, 18.9, 17.5, 11.2. **^{1}H/^{29}Si HMQC NMR** (500/99 MHz, $CDCl_3$, 298 K, optimized for *J* = 7 Hz): δ 1.64/4.87, 1.08/4.87.

4 Experimental Details for the Cationic Silyl-Heck Reactions under Synergistic Silylium-Ion and Palladium Catalysis

4.1 General Procedure for the Cationic Silyl-Heck Reactions (GP 3)

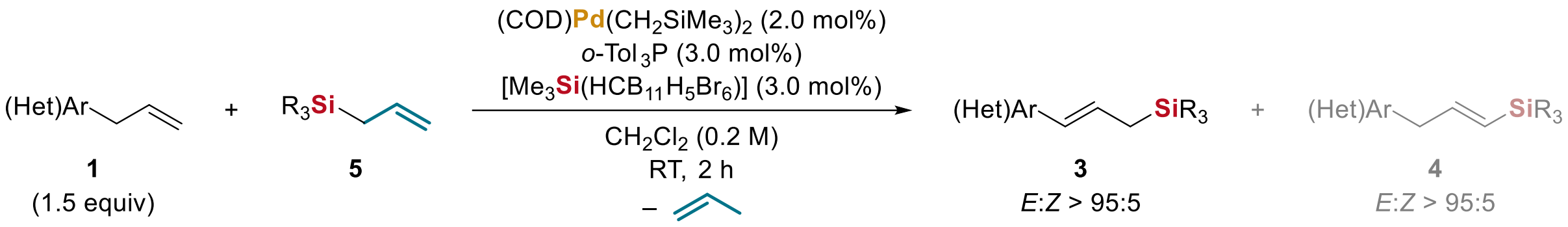

In an argon-filled glovebox, $(COD)Pd(CH_2SiMe_3)_2$ (2.0 mol%) and *o*-Tol_3P (3.0 mol%) are dissolved in degassed anhydrous CH_2Cl_2 (1.0 mL). The mixture is stirred at room temperature for 1 h. Subsequently, silylium carborate $[Me_3Si(HCB_{11}H_5Br_6)]$ (3.0 mol%) is added, followed by the addition of allylarene **1** and allylsilane **5**. The resulting reaction mixture is stirred for additional 2 h at room temperature. Upon completion of the reaction, the mixture is removed from the glovebox and filtered through a short pad of silica gel. The filtrate is concentrated under reduced pressure, and the residue is purified by flash column chromatography on silica gel to afford the desired allylsilane (*E*)-**3**. For allyl-/vinylsilane mixtures, an additional recrystallization from ethyl acetate/*n*-hexane is required to obtain the allylsilane (*E*)-**3** in analytically pure form.

4.2 Characterization Data for Allylsilanes 3

(*E*)-Triphenyl(3-phenylallyl)silane (**3aa**)

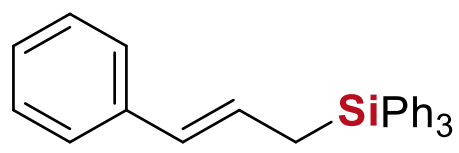

3aa

Allylsilane **3aa** was prepared from allylbenzene (**1a**, 39.7 µL, 0.30 mmol, 1.0 equiv) and allyltriphenylsilane (**5a**, 60.1 mg, 0.20 mmol, 1.0 equiv) using $(COD)Pd(CH_2SiMe_3)_2$ (1.6 mg, 2.0 mol%), *o*-Tol_3P (1.8 mg, 3.0 mol%) and $[Me_3Si(HCB_{11}H_5Br_6)]$ (4.2 mg, 3.0 mol%) in CH_2Cl_2 (1.0 mL) at room temperature following **GP 3** (**3aa**:**4aa** > 95:5 from the crude ^{1}H NMR spectrum). Flash column chromatography on silica gel using *n*-pentane/dichloromethane as the eluent afforded allylsilane **3aa** as a white solid (68.5 mg, 91% yield). For a 1.0-mmol scale, a 96% yield of **3aa** was obtained.

R_f = 0.45 (*n*-pentane/dichloromethane = 6:1). **m. p.** = 75 °C. **IR** (ATR): ṽ = 3066, 3020, 2914, 2688, 2314, 2127, 1956, 1886, 1819, 1640, 1590 cm^{-1}. **^{1}H NMR** (500 MHz, $CDCl_3$, 298 K): δ

7.63 (dd, *J* = 8.0, 1.8 Hz, 6H), 7.51–7.42 (m, 9H), 7.32–7.19 (m, 5H), 6.34–6.33 (m, 2H), 2.63–2.62 (m, 2H). **^{13}C{^{1}H} NMR** (126 MHz, $CDCl_3$, 298 K): δ 138.4, 135.9, 134.6, 130.5, 129.7, 128.5, 128.0, 126.6, 126.3, 125.8, 20.6. **^{1}H/^{29}Si HMQC NMR** (500/99 MHz, $CDCl_3$, 298 K, optimized for *J* = 7 Hz): δ 7.63/–13.54, 2.63/–13.54. **HRMS (APCI)**: calculated for $C_{27}H_{25}Si^+$ [M–H]$^+$: 377.1720; Found 377.1714.

(*E*)-Triphenyl(3-(*p*-tolyl)allyl)silane (**3ba**)

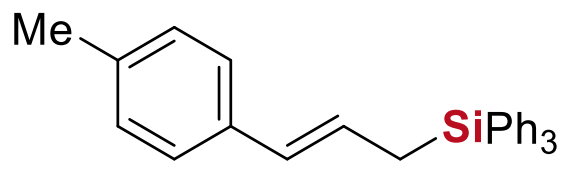

3ba

Allylsilane **3ba** was prepared from 1-allyl-4-methylbenzene (**1b**, 45.8 μL, 0.30 mmol, 1.5 equiv) and allyltriphenylsilane (**5a**, 60.1 mg, 0.20 mmol, 1.0 equiv) using (COD)Pd(CH_2SiMe_3)$_2$ (1.6 mg, 2.0 mol%), *o*-Tol_3P (1.8 mg, 3.0 mol%) and [$Me_3Si(HCB_{11}H_5Br_6)$] (4.2 mg, 3.0 mol%) in CH_2Cl_2 (1.0 mL) at room temperature following **GP 3** (**3ba**:**4ba** > 95:5 from the crude ^{1}H NMR spectrum). Flash column chromatography on silica gel using *n*-pentane/dichloromethane as the eluent afforded allylsilane **3ba** as a white solid (63.3 mg, 81% yield).

R_f = 0.45 (*n*-pentane/dichloromethane = 6:1). **m. p.** = 99 °C. **IR** (ATR): ṽ = 3045, 3018, 2916, 2865, 1957, 1892, 1819, 1771, 1510, 1426, 1151, 1108, 962, 851, 783, 698 cm^{-1}. **^{1}H NMR** (500 MHz, $CDCl_3$, 298 K): δ 7.55 (d, *J* = 7.3 Hz, 6H), 7.43–7.35 (m, 9H), 7.09–7.04 (m, 4H), 6.26–6.20 (m, 2H), 2.53 (d, *J* = 6.9 Hz, 2H), 2.30 (s, 3H). **^{13}C{^{1}H} NMR** (126 MHz, $CDCl_3$, 298 K): δ 136.2, 135.9, 135.6, 134.7, 130.4, 129.7, 129.2, 128.00, 127.97, 125.7, 125.2, 21.2, 20.4. **^{1}H/^{29}Si HMQC NMR** (500/99 MHz, $CDCl_3$, 298 K, optimized for *J* = 7 Hz): δ 7.55/–13.70, 2.53/–13.70. **HRMS (APCI)**: calculated for $C_{28}H_{27}Si^+$ [M–H]$^+$: 391.1877; Found 391.1869.

(*E*)-(3-(4-(*tert*-Butyl)phenyl)allyl)triphenylsilane (**3ca**)

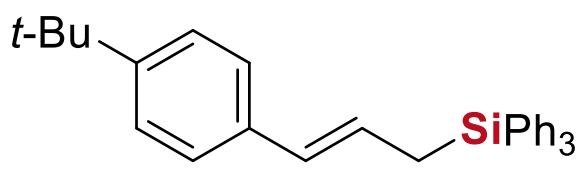

3ca

Allylsilane **3ca** was prepared from 1-allyl-4-(*tert*-butyl)benzene (**1c**, 52.3 mg, 0.30 mmol, 1.5 equiv) and allyltriphenylsilane (**5a**, 60.1 mg, 0.20 mmol, 1.0 equiv) using (COD)Pd(CH_2SiMe_3)$_2$ (1.6 mg, 2.0 mol%), *o*-Tol_3P (1.8 mg, 3.0 mol%) and $Me_3Si[HCB_{11}H_5Br_6]$ (4.2 mg, 3.0 mol%) in CH_2Cl_2 (1.0 mL) at room temperature following **GP 3** (**3ca**:**4ca** > 95:5

from the crude ^{1}H NMR spectrum). Flash column chromatography on silica gel using *n*-pentane/dichloromethane as the eluent afforded allylsilane **3ca** as a white solid (74.4 mg, 86% yield).

R*f* = 0.50 (*n*-pentane/dichloromethane = 6:1). **m. p.** = 110 °C. **IR** (ATR): ṽ = 3067, 3021, 2960, 1642, 1512, 1427, 1394, 1362, 1153, 1110, 963, 854, 814, 700 cm^{-1}. **^{1}H NMR** (500 MHz, $CDCl_3$, 298 K): δ 7.45 (d, *J* = 7.1 Hz, 6H), 7.32–7.25 (m, 9H), 7.17–7.16 (m, 2H), 7.03 (d, *J* = 8.1 Hz, 2H), 6.19–6.09 (m, 2H), 2.43 (d, *J* = 7.2 Hz, 2H), 1.20 (s, 9H). **^{13}C{^{1}H} NMR** (126 MHz, $CDCl_3$, 298 K): δ 149.5, 135.9, 135.7, 134.7, 130.2, 129.7, 128.00, 128.79, 125.58, 125.56, 125.4, 34.6, 31.5, 20.5. **^{1}H/^{29}Si HMQC NMR** (500/99 MHz, $CDCl_3$, 298 K, optimized for *J* = 7 Hz): δ 7.57/–13.66, 2.55/–13.66. **HRMS (APCI)**: calculated for $C_{31}H_{33}Si^+$ [M–H]$^+$: 433.2346; Found 433.2334.

(*E*)-Trimethyl(4-(3-(triphenylsilyl)prop-1-en-1-yl)phenyl)silane (**3da**)

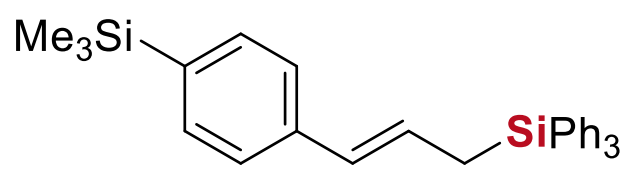

3da

Allylsilane **3da** was prepared from (4-allylphenyl)trimethylsilane (**1d**, 57.1 mg, 0.30 mmol, 1.5 equiv) and allyltriphenylsilane (**5a**, 60.1 mg, 0.20 mmol, 1.0 equiv) using (COD)Pd(CH_2SiMe_3)$_2$ (1.6 mg, 2.0 mol%), *o*-Tol_3P (1.8 mg, 3.0 mol%) and [$Me_3Si(HCB_{11}H_5Br_6)$] (4.2 mg, 3.0 mol%) in CH_2Cl_2 (1.0 mL) at room temperature following **GP 3** (**3da**:**4da** > 95:5 from the crude ^{1}H NMR spectrum). Flash column chromatography on silica gel using *n*-pentane/dichloromethane as the eluent afforded allylsilane **3da** as a white solid (73.6 mg, 82% yield).

R*f* = 0.40 (*n*-pentane/dichloromethane = 6:1). **m. p.** = 88 °C. **IR** (ATR): ṽ = 3066, 3014, 2953, 1962, 1902, 1821, 1595, 1427, 1247, 1110, 964, 837, 724, 700 cm^{-1}. **^{1}H NMR** (500 MHz, $CDCl_3$, 298 K): δ 7.54 (d, *J* = 8.2 Hz, 6H), 7.44–7.35 (m, 11H), 7.18–7.16 (m, 2H), 6.28–6.27 (m, 2H), 2.53 (d, *J* = 5.9 Hz, 2H), 0.24 (s, 9H). **^{13}C{^{1}H} NMR** (126 MHz, $CDCl_3$, 298 K): δ 138.9, 138.5, 135.9, 134.6, 133.6, 130.5, 129.8, 128.0, 126.8, 125.2, 20.7, –1.0. **^{1}H/^{29}Si HMQC NMR** (500/99 MHz, $CDCl_3$, 298 K, optimized for *J* = 7 Hz): 7.54/–13.62, 2.53/–13.62, 0.24/–4.68. **HRMS (APCI)**: calculated for $C_{30}H_{33}Si_2^+$ [M–H]$^+$: 449.2115; Found 449.2105.

(*E*)-Triphenyl(3-(4-(trifluoromethyl)phenyl)allyl)silane (**3ea**)

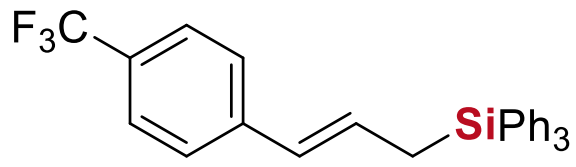

3ea

Allylsilane **3ea** was prepared from 1-allyl-4-(trifluoromethyl)benzene (**1e**, 55.9 mg, 0.30 mmol, 1.5 equiv) and allyltriphenylsilane (**5a**, 60.1 mg, 0.20 mmol, 1.0 equiv) using (COD)Pd(CH_2SiMe_3)$_2$ (1.6 mg, 2.0 mol%), *o*-Tol_3P (1.8 mg, 3.0 mol%) and [$Me_3Si(HCB_{11}H_5Br_6)$] (4.2 mg, 3.0 mol%) in CH_2Cl_2 (1.0 mL) at room temperature following **GP 3** (**3ea**:**4ea** = 84:16 from the crude ^{1}H NMR spectrum). Flash column chromatography on silica gel using *n*-pentane/dichloromethane as the eluent afforded an isomeric mixture. Further purification by crystallization from *n*-hexane/ethyl acetate furnished allylsilane **3ea** as a white solid (42.7 mg, 48% yield).

R_f = 0.45 (*n*-pentane/dichloromethane = 6:1). **m. p.** = 86 °C. **IR** (ATR): ṽ = 3067, 3018, 2920, 1958, 1888, 1611, 1427, 1322, 1160, 1107, 1066, 965, 862, 817, 772, 698 cm^{-1}. **^{1}H NMR** (500 MHz, $CDCl_3$, 298 K): δ 7.54 (d, *J* = 6.4 Hz, 6H), 7.48–7.42 (m, 5H), 7.39–7.36 (m, 6H), 7.26–7.23 (m, 2H), 6.40–6.34 (m, 1H), 6.26 (d, *J* = 15.8 Hz, 1H), 2.58 (d, *J* = 7.9 Hz, 2H). **^{19}F NMR** (471 MHz, $CDCl_3$, 298 K): δ –62.38. **^{13}C{^{1}H} NMR** (126 MHz, $CDCl_3$, 298 K): δ 141.8, 135.9, 134.3, 129.9, 129.5, 129.2, 128.1, 125.8, 125.53, 125.50, 20.94. **^{1}H/^{29}Si HMQC NMR** (500/99 MHz, $CDCl_3$, 298 K, optimized for *J* = 7 Hz): δ 7.54/–13.33, 2.58/–13.33. **HRMS (APCI)**: calculated for $C_{28}H_{24}F_3Si^+$ [M–H]$^+$: 445.1594; Found 445.1592.

(*E*)-(3-(4-Fluorophenyl)allyl)triphenylsilane (**3fa**)

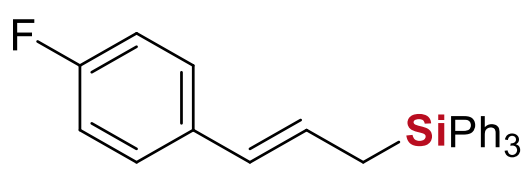

3fa

Allylsilane **3fa** was prepared from 1-allyl-4-fluorobenzene (**1f**, 40.5 μL, 0.30 mmol, 1.5 equiv) and allyltriphenylsilane (**5a**, 60.1 mg, 0.20 mmol, 1.0 equiv) using (COD)Pd(CH_2SiMe_3)$_2$ (1.6 mg, 2.0 mol%), *o*-Tol_3P (1.8 mg, 3.0 mol%) and [$Me_3Si(HCB_{11}H_5Br_6)$] (4.2 mg, 3.0 mol%) in CH_2Cl_2 (1.0 mL) at room temperature following **GP 3** (**3fa**:**4fa** > 95:5 from the crude ^{1}H NMR spectrum). Flash column chromatography on silica gel using *n*-pentane/dichloromethane as the eluent afforded allylsilane **3fa** as a white solid (55.9 mg, 71% yield).

R*f* = 0.45 (*n*-pentane/dichloromethane = 6:1). **m. p.** = 89 °C. **IR** (ATR): ṽ = 3067, 3017, 2913, 1957, 1885, 1599, 1505, 1426, 1396, 1225, 1155, 1109, 1027, 962, 852, 815, 698 cm^{-1}. **^{1}H NMR** (500 MHz, $CDCl_3$, 298 K): δ 7.58 (d, *J* = 7.5 Hz, 6H), 7.47–7.38 (m, 9H), 7.16–7.13 (m, 2H), 6.96–6.92 (m, 2H), 6.27–6.16 (m, 2H), 2.56 (d, *J* = 7.4 Hz, 2H). **^{19}F NMR** (471 MHz, $CDCl_3$, 298 K): δ –116.35. **^{13}C{^{1}H} NMR** (126 MHz, $CDCl_3$, 298 K): δ 161.9 (d, $J_{C,F}$ = 239.4 Hz), 135.9, 134.5, 129.8, 129.3, 128.1, 127.2, 127.1, 126.10, 126.08, 115.4, 115.3, 20.5. **^{1}H/^{29}Si HMQC NMR** (500/99 MHz, $CDCl_3$, 298 K, optimized for *J* = 7 Hz): δ 7.58/–13.53, 2.56/–13.53. **HRMS (APCI)**: calculated for $C_{27}H_{24}FSi^+$ $[M–H]^+$: 395.1626; Found 395.1618.

(*E*)-(3-(4-Chlorophenyl)allyl)triphenylsilane (**3ga**)

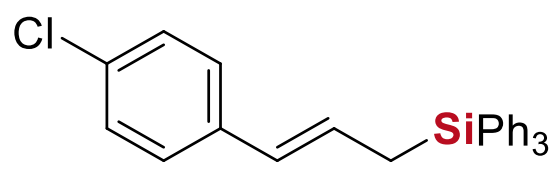

3ga

Allylsilane **3ga** was prepared from 1-allyl-4-chlorobenzene (**1g**, 45.8 mg, 0.30 mmol, 1.5 equiv) and allyltriphenylsilane (**5a**, 60.1 mg, 0.20 mmol, 1.0 equiv) using $(COD)Pd(CH_2SiMe_3)_2$ (1.6 mg, 2.0 mol%), *o*-Tol_3P (1.8 mg, 3.0 mol%) and $[Me_3Si(HCB_{11}H_5Br_6)]$ (4.2 mg, 3.0 mol%) in CH_2Cl_2 (1.0 mL) at room temperature following **GP 3** (**3ga**:**4ga** > 95:5 from the crude ^{1}H NMR spectrum). Flash column chromatography on silica gel using *n*-pentane/dichloromethane as the eluent and subsequent crystallization from *n*-hexane/ethyl acetate afforded allylsilane **3ga** as a white solid (55.0 mg, 67% yield).

R*f* = 0.45 (*n*-pentane/dichloromethane = 6:1). **m. p.** = 98 °C. **IR** (ATR): ṽ = 3067, 3018, 2926, 2687, 2300, 1891, 1821, 1640, 1487, 1426, 1263, 1109, 1010, 963, 854, 808, 733, 698 cm^{-1}. **^{1}H NMR** (500 MHz, $CDCl_3$, 298 K): δ 7.58–7.52 (m, 6H), 7.46–7.43 (m, 3H), 7.40–7.37 (m, 6H), 7.23–7.19 (m, 2H), 7.13–7.08 (m, 2H), 6.29–6.19 (m, 2H), 2.56–2.53 (m, 2H). **^{13}C{^{1}H} NMR** (126 MHz, $CDCl_3$, 298 K): δ 136.9, 135.9, 134.5, 132.1, 129.9, 129.3, 128.7, 128.1, 127.2, 127.0, 20.7. **^{1}H/^{29}Si HMQC NMR** (500/99 MHz, $CDCl_3$, 298 K, optimized for *J* = 7 Hz): δ 7.56/–13.51, 2.55/–13.51. **HRMS (APCI)**: calculated for $C_{27}H_{24}ClSi^+$ $[M–H]^+$: 411.1330; Found 411.1322.

(*E*)-(3-(4-Bromophenyl)allyl)triphenylsilane (**3ha**)

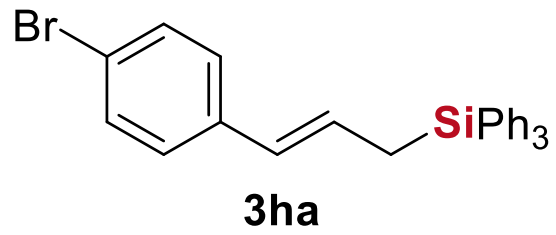

3ha

Allylsilane **3ha** was prepared from 1-allyl-4-bromobenzene (**1h**, 59.1 mg, 0.30 mmol, 1.5 equiv) and allyltriphenylsilane (**5a**, 60.1 mg, 0.20 mmol, 1.0 equiv) using (COD)Pd(CH_2SiMe_3)$_2$ (1.6 mg, 2.0 mol%), *o*-Tol$_3$P (1.8 mg, 3.0 mol%) and [$Me_3Si(HCB_{11}H_5Br_6)$] (4.2 mg, 3.0 mol%) in CH_2Cl_2 (1.0 mL) at room temperature following **GP 3** (**3ha**:**4ha** > 95:5 from the crude ^{1}H NMR spectrum). Flash column chromatography on silica gel using *n*-pentane/dichloromethane as the eluent and subsequent crystallization from *n*-hexane/ethyl acetate afforded allylsilane **3ha** as a white solid (71.6 mg, 79% yield).

R*f* = 0.45 (*n*-pentane/dichloromethane = 6:1). **m. p.** = 108 °C. **IR** (ATR): ṽ = 3066, 3017, 2926, 2688, 1957, 1891, 1772, 1484, 1426, 1393, 1149, 1108, 1071, 962, 852, 805, 697 cm^{-1}. **^{1}H NMR** (500 MHz, $CDCl_3$, 298 K): δ 7.56 (d, *J* = 6.5 Hz, 6H), 7.46–7.35 (m, 11H), 7.04 (d, *J* = 8.4 Hz, 2H), 6.29–6.17 (m, 2H), 2.55 (d, *J* = 7.8 Hz, 2H). **^{13}C{^{1}H} NMR** (126 MHz, $CDCl_3$, 298 K): δ 137.3, 135.9, 134.4, 131.6, 129.8, 129.3,128.1, 127.4, 127.3, 120.2, 20.7. **^{1}H/^{29}Si HMQC NMR** (500/99 MHz, $CDCl_3$, 298 K, optimized for *J* = 7 Hz): δ 7.56/–13.51, 2.55/–13.51. **HRMS (APCI)**: calculated for $C_{27}H_{24}BrSi^+$ [M–H]$^+$: 455.0825; Found 455.0814.

(*E*)-Triphenyl(3-(*m*-tolyl)allyl)silane (**3ia**)

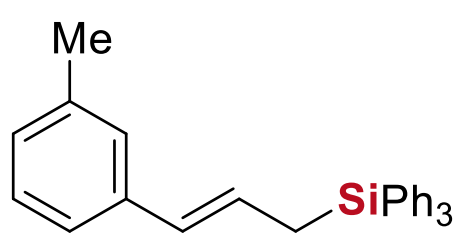

3ia

Allylsilane **3ia** was prepared from 1-allyl-3-methylbenzene (**1i**, 39.7 mg, 0.30 mmol, 1.5 equiv) and allyltriphenylsilane (**5a**, 60.1 mg, 0.20 mmol, 1.0 equiv) using (COD)Pd(CH_2SiMe_3)$_2$ (1.6 mg, 2.0 mol%), *o*-Tol$_3$P (1.8 mg, 3.0 mol%) and [$Me_3Si(HCB_{11}H_5Br_6)$] (4.2 mg, 3.0 mol%) in CH_2Cl_2 (1.0 mL) at room temperature following **GP 3** (**3ia**:**4ia** > 95:5 from the crude ^{1}H NMR spectrum). Flash column chromatography on silica gel using *n*-pentane/dichloromethane as the eluent afforded allylsilane **3ia** as a white solid (59.3 mg, 76% yield).

R*f* = 0.45 (*n*-pentane/dichloromethane = 6:1). **m. p.** = 59 °C. **IR** (ATR): ṽ = 3045, 3013, 2915, 2860, 1957, 1886, 1639, 1485, 1426, 1260, 1151, 1108, 960, 836, 781, 697 cm^{-1}. **^{1}H NMR** (500 MHz, $CDCl_3$, 298 K): δ 7.63 (d, *J* = 6.0 Hz, 6H), 7.50–7.47 (m, 3H), 7.44–7.41 (m, 6H), 7.21–7.17 (m, 1H), 7.07–7.02 (m, 3H), 6.32 (m, 2H), 2.61 (d, *J* = 3.4 Hz, 2H), 2.36 (s, 3H). **^{13}C{^{1}H} NMR** (126 MHz, $CDCl_3$, 298 K): δ 138.3, 138.0, 135.9, 134.6, 130.6, 129.7, 128.4, 128.0, 127.4, 126.6, 126.1, 122.9, 21.5, 20.5. **^{1}H/^{29}Si HMQC NMR** (500/99 MHz, $CDCl_3$, 298

K, optimized for J = 7 Hz): δ 7.63/–13.58, 2.61/–13.58. **HRMS (APCI)**: calculated for $C_{28}H_{27}Si^+$ [M–H]$^+$: 391.1877; Found 391.1868.

(*E*)-(3-(3-Fluorophenyl)allyl)triphenylsilane (**3ja**)

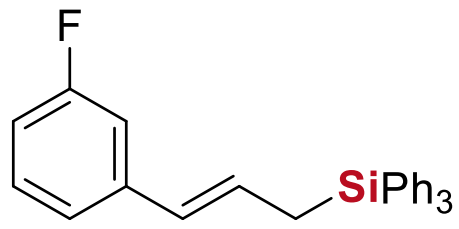

3ja

Allylsilane **3ja** was prepared from 1-allyl-3-fluorobenzene (**1j**, 40.8 mg, 0.30 mmol, 1.5 equiv) and allyltriphenylsilane (**5a**, 60.1 mg, 0.20 mmol, 1.0 equiv) using (COD)Pd(CH_2SiMe_3)$_2$ (1.6 mg, 2.0 mol%), *o*-Tol_3P (1.8 mg, 3.0 mol%) and [$Me_3Si(HCB_{11}H_5Br_6)$] (4.2 mg, 3.0 mol%) in CH_2Cl_2 (1.0 mL) at room temperature following **GP 3** (**3ja**:**4ja** > 95:5 from the crude ^{1}H NMR spectrum). Flash column chromatography on silica gel using *n*-pentane/dichloromethane as the eluent and subsequent crystallization from *n*-hexane/ethyl acetate afforded allylsilane **3ja** as a white solid (39.4 mg, 50% yield).

R*f* = 0.50 (*n*-pentane/dichloromethane = 6:1). **m. p.** = 75 °C. **IR** (ATR): ṽ = 3067, 3018, 2912, 1958, 1887, 1607, 1579, 1485, 1426, 1267, 1139, 1108, 956, 872, 791, 697 cm^{-1}. **^{1}H NMR** (500 MHz, $CDCl_3$, 298 K): δ 7.55 (d, J = 6.5 Hz, 6H), 7.45–7.42 (m, 3H), 7.40–7.37 (m, 6H), 7.20–7.16 (m, 1H), 6.93 (d, J = 7.8 Hz, 1H), 6.88–6.82 (m, 2H), 6.30–6.25 (m, 1H), 6.22 (d, J = 15.8 Hz, 1H), 2.55 (d, J = 7.5 Hz, 2H). **^{19}F NMR** (471 MHz, $CDCl_3$, 298 K): δ –114.00 (td, J = 9.5, 6.3 Hz). **^{13}C{^{1}H} NMR** (126 MHz, $CDCl_3$, 298 K): δ 163.3 (d, $J_{C,F}$ = 245.0 Hz), 140.7 (d, J = 8.0 Hz), 135.9, 134.4, 129.8, 129.4, 128.1, 121.6, 113.3 (d, J = 21.5 Hz), 112.2 (d, J = 22.0 Hz), 20.7. **^{1}H/^{29}Si HMQC NMR** (500/99 MHz, $CDCl_3$, 298 K, optimized for J = 7 Hz): δ 7.55/–13.49, 2.55/–13.49. **HRMS (APCI)**: calculated for $C_{27}H_{24}Si^+$ [M–H]$^+$: 395.1626; Found 395.1623.

(*E*)-(3-(3-Chlorophenyl)allyl)triphenylsilane (**3ka**)

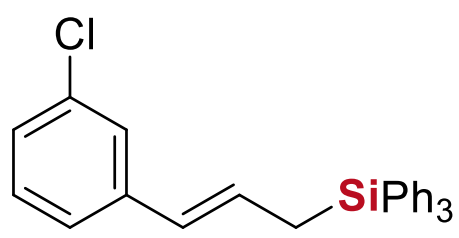

3ka

Allylsilane **3ka** was prepared from 1-allyl-3-chlorobenzene (**1k**, 55.9 mg, 0.30 mmol, 1.5 equiv) and allyltriphenylsilane (**5a**, 60.1 mg, 0.20 mmol, 1.0 equiv) using (COD)Pd(CH_2SiMe_3)$_2$ (1.6 mg, 2.0 mol%), *o*-Tol_3P (1.8 mg, 3.0 mol%) and

[$Me_3Si(HCB_{11}H_5Br_6)$] (4.2 mg, 3.0 mol%) in CH_2Cl_2 (1.0 mL) at room temperature following **GP 3** (**3ka**:**4ka** = 95:5 from the crude ^{1}H NMR spectrum). Flash column chromatography on silica gel using *n*-pentane/dichloromethane as the eluent and subsequent crystallization from *n*-hexane/ethyl acetate afforded allylsilane **3ka** as a white solid (59.2 mg, 72% yield).

R_f = 0.45 (*n*-pentane/dichloromethane = 6:1). **m. p.** = 64 °C. **IR** (ATR): ṽ = 3066, 3015, 2923, 1957, 1886, 1640, 1590, 1474, 1426, 1260, 1109, 961, 764, 697 cm^{-1}. **^{1}H NMR** (500 MHz, $CDCl_3$, 298 K): δ 7.57–7.55 (m, 6H), 7.54–7.44 (m, 3H), 7.41–7.38 (m, 6H), 7.18–7.11 (m, 3H), 7.05 (d, *J* = 7.5 Hz, 1H), 6.30 (dt, *J* = 15.7 Hz, 7.9 Hz, 1H), 6.20 (d, *J* = 15.8 Hz, 1H), 2.55 (d, *J* = 7.4 Hz, 2H). **$^{13}C\{^1H\}$ NMR** (126 MHz, $CDCl_3$, 298 K): δ 140.2, 135.9, 134.5, 134.4, 129.8, 129.7, 129.2, 128.2, 128.1, 126.5, 125.7, 123.9, 20.7. **$^1H/^{29}Si$ HMQC NMR** (500/99 MHz, $CDCl_3$, 298 K, optimized for *J* = 7 Hz): δ 7.56/–13.43, 2.55/–13.43. **HRMS (APCI)**: calculated for $C_{27}H_{24}ClSi^+$ [M–H]$^+$: 412.1330; Found 412.1329.

(*E*)-Triphenyl(3-(*o*-tolyl)allyl)silane (**3la**)

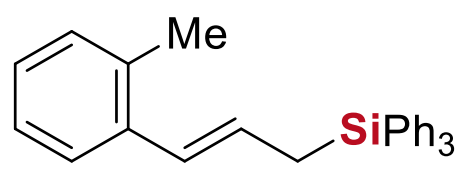

3la

Allylsilane **3la** was prepared from 1-allyl-2-methylbenzene (**1l**, 39.7 mg, 0.30 mmol, 1.5 equiv) and allyltriphenylsilane (**5a**, 60.1 mg, 0.20 mmol, 1.0 equiv) using (COD)Pd(CH_2SiMe_3)$_2$ (1.6 mg, 2.0 mol%), *o*-Tol_3P (1.8 mg, 3.0 mol%) and [$Me_3Si(HCB_{11}H_5Br_6)$] (4.2 mg, 3.0 mol%) in CH_2Cl_2 (1.0 mL) at room temperature following **GP 3** (**3la**:**4la** > 95:5 from the crude ^{1}H NMR spectrum). Flash column chromatography on silica gel using *n*-pentane/dichloromethane as the eluent afforded allylsilane **3la** as a white solid (62.5 mg, 80% yield).

R_f = 0.45 (*n*-pentane/dichloromethane = 6:1). **m. p.** = 58 °C. **IR** (ATR): ṽ = 3066, 3014, 2688, 1954, 1887, 1483, 1426, 1263, 1108, 962, 789, 723, 696 cm^{-1}. **^{1}H NMR** (500 MHz, $CDCl_3$, 298 K): δ 7.75 (d, *J* = 7.8 Hz, 6H), 7.60–7.52 (m, 9H), 7.43 (d, *J* = 5.7 Hz, 1H), 7.26–7.24 (m, 3H), 6.66 (d, *J* = 15.6 Hz, 1H), 6.34 (dt, *J* = 15.7 Hz, 7.9 Hz, 1H), 2.76 (d, *J* = 8.1 Hz, 2H), 2.32 (s, 3H). **$^{13}C\{^1H\}$ NMR** (126 MHz, $CDCl_3$, 298 K): δ 137.5, 135.9, 134.8, 134.6, 130.1, 129.7, 128.6, 128.0, 127.5, 126.6, 126.1, 125.5, 20.9, 19.8. **$^1H/^{29}Si$ HMQC NMR** (500/99 MHz, $CDCl_3$, 298 K, optimized for *J* = 7 Hz): δ 7.75/–13.33, 2.73/–13.33. **HRMS (APCI)**: calculated for $C_{28}H_{27}Si^+$ [M–H]$^+$: 391.1877; Found 391.1878.

(*E*)-(3-(2-Fluorophenyl)allyl)triphenylsilane (**3ma**)

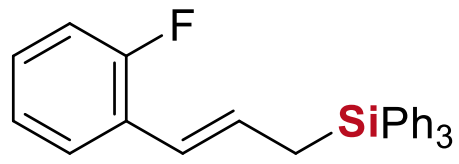

3ma

Allylsilane **3ma** was prepared from 1-allyl-2-fluorobenzene (**1m**, 40.9 mg, 0.30 mmol, 1.5 equiv) and allyltriphenylsilane (**5a**, 60.1 mg, 0.20 mmol, 1.0 equiv) using (COD)Pd(CH_2SiMe_3)$_2$ (1.6 mg, 2.0 mol%), *o*-Tol_3P (1.8 mg, 3.0 mol%) and [$Me_3Si(HCB_{11}H_5Br_6)$] (4.2 mg, 3.0 mol%) in CH_2Cl_2 (1.0 mL) at room temperature following **GP 3** (**3ma**:**4ma** = 90:10 from the crude ^{1}H NMR spectrum). Flash column chromatography on silica gel using *n*-pentane/dichloromethane as the eluent and subsequent crystallization from *n*-hexane/ethyl acetate afforded allylsilane **3ma** as a white solid (55.2 mg, 70% yield). **R*f*** = 0.50 (*n*-pentane/dichloromethane = 6:1). **m. p.** = 82 °C. **IR** (ATR): ṽ = 3043, 2911, 1953, 1889, 1639, 1483, 1426, 1228, 1150, 1108, 1027, 963, 802, 726, 697 cm^{-1}. **^{1}H NMR** (500 MHz, $CDCl_3$, 298 K): δ 7.56 (d, *J* = 6.2 Hz, 6H), 7.45–7.36 (m, 9H), 7.24–7.21 (m, 1H), 7.11–7.09 (m, 1H), 7.01–6.95 (m, 2H), 6.45 (d, *J* = 15.9 Hz, 1H), 6.34 (dt, *J* = 15.9, 7.9 Hz, 1H), 2.59 (d, *J* = 7.9 Hz, 2H). **^{19}F NMR** (471 MHz, $CDCl_3$, 298 K): δ –119.02 (dt, *J* = 12.1, 6.1 Hz). **^{13}C{^{1}H} NMR** (126 MHz, $CDCl_3$, 298 K): δ 159.8 (d, *J* = 247.6 Hz), 135.9, 134.5, 129.8, 129.3 (d, *J* = 4.6 Hz), 128.1, 127.7 (d, *J* = 8.2 Hz), 127.0 (d, *J* = 3.8 Hz), 126.1 (d, *J* = 12.4 Hz), 124.0 (d, *J* = 3.5 Hz), 122.8 (d, *J* = 3.4 Hz), 115.6 (d, *J* = 22.2 Hz), 21.2. **^{1}H/^{29}Si HMQC NMR** (500/99 MHz, $CDCl_3$, 298 K, optimized for *J* = 7 Hz): δ 7.56/–13.07, 2.59/–13.07. **HRMS (APCI)**: calculated for $C_{27}H_{24}FSi^+$ [M–H]$^+$: 395.1626; Found 395.1623.

(*E*)-(3-(2-Bromophenyl)allyl)triphenylsilane (**1na**)

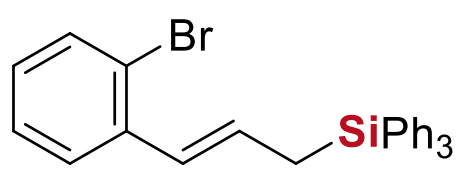

3na

Allylsilane **3na** was prepared from 1-allyl-2-bromobenzene (**1n**, 59.1 mg, 0.30 mmol, 1.5 equiv) and allyltriphenylsilane (**5a**, 60.1 mg, 0.20 mmol, 1.0 equiv) using (COD)Pd(CH_2SiMe_3)$_2$ (1.6 mg, 2.0 mol%), *o*-Tol_3P (1.8 mg, 3.0 mol%) and [$Me_3Si(HCB_{11}H_5Br_6)$] (4.2 mg, 3.0 mol%) in CH_2Cl_2 (1.0 mL) at room temperature following **GP 3** (**3na**:**4na** = 92:8 from the crude ^{1}H NMR spectrum). Flash column chromatography on

silica gel using *n*-pentane/dichloromethane as the eluent and subsequent crystallization from *n*-hexane/ethyl acetate afforded allylsilane **3na** as a white solid (29.2 mg, 32% yield).

R*f* = 0.45 (*n*-pentane/dichloromethane = 6:1). **m. p.** = 64 °C. **IR** (ATR): ṽ = 3066, 3014, 2652, 1632, 1585, 1424, 1263, 1161, 1106, 1020, 967, 839, 753, 697 cm^{-1}. **^{1}H NMR** (500 MHz, $CDCl_3$, 298 K): δ 7.57–7.37 (m, 16H), 7.27 (d, *J* = 8.6 Hz, 1H), 7.16 (t, *J* = 7.5 Hz, 1H), 7.00 (t, *J* = 7.4 Hz, 1H), 6.67 (d, *J* = 15.4 Hz, 1H), 6.19 (dt, *J* = 15.9 Hz, 8.1 Hz, 1H), 2.62 (d, *J* = 8.2 Hz, 2H). **^{13}C{^{1}H} NMR** (126 MHz, $CDCl_3$, 298 K): δ 138.2, 135.9, 134.5, 132.9, 129.8, 129.4, 128.1, 127.9, 127.4, 126.9, 123.0, 21.1. **^{1}H/^{29}Si HMQC NMR** (500/99 MHz, $CDCl_3$, 298 K, optimized for *J* = 7 Hz): δ 7.57/–14.00, 2.62/–14.00. **HRMS (APCI)**: calculated for $C_{27}H_{23}BrSi^+$ $[M–H]^+$: 455.0825; Found 455.0822.

(*E*)-(3-Mesitylallyl)triphenylsilane (**3oa**)

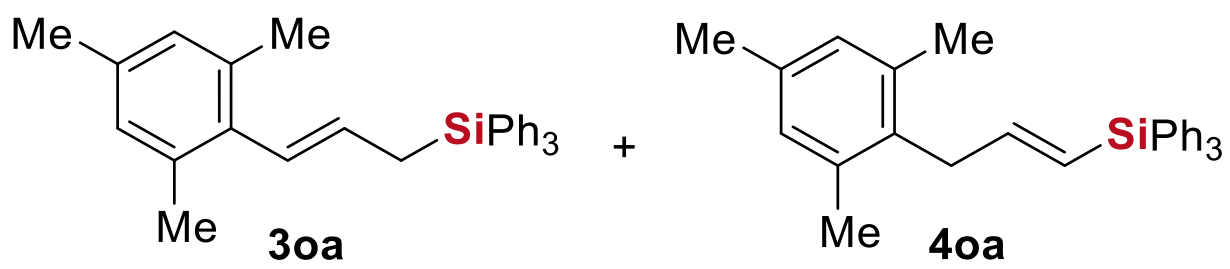

Allylsilane **3oa** was prepared from 2-allyl-1,3,5-trimethylbenzene (**1o**, 48.1 mg, 0.30 mmol, 1.5 equiv) and allyltriphenylsilane (**5a**, 60.1 mg, 0.20 mmol, 1.0 equiv) using $(COD)Pd(CH_2SiMe_3)_2$ (1.6 mg, 2.0 mol%), *o*-Tol_3P (1.8 mg, 3.0 mol%) and $[Me_3Si(HCB_{11}H_5Br_6)]$ (4.2 mg, 3.0 mol%) in CH_2Cl_2 (1.0 mL) at room temperature following **GP 3** (**3oa**:**4oa** = 30:70 from the crude ^{1}H NMR spectrum). Flash column chromatography on silica gel using *n*-pentane/dichloromethane as the eluent afforded a mixture of **3oa** and **4oa** (18.4 mg, 22% yield), which is difficult to separate. **R*f*** = 0.4 (pentane/dichloromethane = 6/1). The following spectroscopic data were confirmed by an **H,H COSY** (500 MHz, $CDCl_3$, 298 K, NUS 50%) experiment.

Spectroscopic data for **3oa**: **^{1}H NMR** (500 MHz, $CDCl_3$, 298 K): δ 7.60 (d, *J* = 6.5 Hz, 6H), 7.43–7.34 (m, 9H), 6.81 (s, 2H), 6.34–6.28 (m, 1H), 5.79 (dt, *J* = 15.9 Hz, 7.8 Hz, 1H), 2.62 (d, *J* = 6.9 Hz, 2H), 2.25 (s, 3H), 2.06 (s, 6H). **^{1}H/^{29}Si HMQC NMR** (500/99 MHz, $CDCl_3$, 298 K, optimized for *J* = 7 Hz): δ 2.62/–13.65.

Spectroscopic data for **4oa**: **^{1}H NMR** (500 MHz, $CDCl_3$, 298 K): δ 7.50 (d, *J* = 6.5 Hz, 6H), 7.43–7.34 (m, 9H), 6.88 (s, 2H), 6.34–6.28 (m, 1H), 6.03 (d, *J* = 18.5 Hz, 1H), 3.57 (d, *J* = 5.0 Hz, 2H), 2.29 (s, 3H), 2.28 (s, 6H). **^{1}H/^{29}Si HMQC NMR** (500/99 MHz, $CDCl_3$, 298 K,

optimized for J = 7 Hz): δ 6.32/–18.02, 6.03/–18.02.

(E)-(3-([1,1'-Biphenyl]-4-yl)allyl)triphenylsilane (**3pa**)

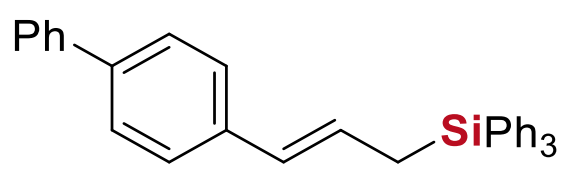

3pa

Allylsilane **3pa** was prepared from 4-allyl-1,1'-biphenyl (**1p**, 58.3 mg, 0.30 mmol, 1.5 equiv) and allyltriphenylsilane (**5a**, 60.1 mg, 0.20 mmol, 1.0 equiv) using (COD)Pd(CH_2SiMe_3)$_2$ (1.6 mg, 2.0 mol%), o-Tol$_3$P (1.8 mg, 3.0 mol%) and [$Me_3Si(HCB_{11}H_5Br_6)$] (4.2 mg, 3.0 mol%) in CH_2Cl_2 (1.0 mL) at room temperature following **GP 3** (**3pa**:**4pa** > 95:5 from the crude ^{1}H NMR spectrum). Flash column chromatography on silica gel using n-pentane/dichloromethane as the eluent afforded allylsilane **3pa** as a white solid (61.5 mg, 68% yield).

R$_f$ = 0.50 (n-pentane/dichloromethane = 6:1). **m. p.** = 120 °C. **IR** (ATR): ṽ = 3046, 3022, 2914, 2689, 1956, 1888, 1821, 1700, 1638, 1598, 1484, 1426, 1263, 1107, 963, 857, 695 cm^{-1}. **^{1}H NMR** (500 MHz, $CDCl_3$, 298 K): δ 7.61–7.51 (m, 10H), 7.48–7.40 (m, 11H), 7.36–7.32 (m, 1H), 7.29 (d, J = 7.9 Hz, 2H), 6.36–6.35 (m, 2H), 2.61–2.60 (m, 2H). **^{13}C{^{1}H} NMR** (126 MHz, $CDCl_3$, 298 K): δ 114.1, 139.4, 137.4, 135.9, 134.6, 130.0, 129.8, 128.9, 128.1, 127.3, 127.2, 127.0, 126.6, 126.2, 20.7. **^{1}H/^{29}Si HMQC NMR** (500/99 MHz, $CDCl_3$, 298 K, optimized for J = 7 Hz): δ 7.56/–13.71, 2.61/–13.71. **HRMS (APCI)**: calculated for $C_{33}H_{29}Si^+$ [M–H]$^+$: 453.2033; Found 453.1978.

(E)-(3-(Naphthalen-1-yl)allyl)triphenylsilane (**3qa**)

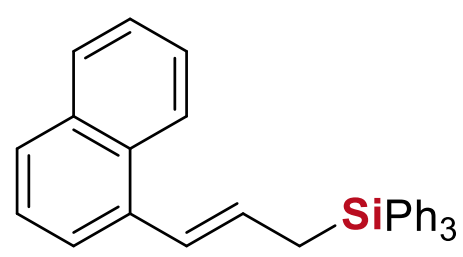

3qa

Allylsilane **3qa** was prepared from 1-allylnaphthalene (**1q**, 50.5 mg, 0.30 mmol, 1.5 equiv) and allyltriphenylsilane (**5a**, 60.1 mg, 0.20 mmol, 1.0 equiv) using (COD)Pd(CH_2SiMe_3)$_2$ (1.6 mg, 2.0 mol%), o-Tol$_3$P (1.8 mg, 3.0 mol%) and [$Me_3Si(HCB_{11}H_5Br_6)$] (4.2 mg, 3.0 mol%) in CH_2Cl_2 (1.0 mL) at room temperature following **GP 3** (**3qa**:**4qa** = 90:10 from the crude ^{1}H NMR spectrum). Flash column chromatography on silica gel using n-pentane/dichloromethane as the eluent and subsequent crystallization from n-hexane/ethyl acetate afforded allylsilane **3qa** as a white solid (55.5 mg, 65% yield).

R*f* = 0.45 (*n*-pentane/dichloromethane = 6:1). **m. p.** = 105 °C. **IR** (ATR): ṽ = 3046, 3011, 2877, 2112, 1587, 1484, 1424, 1144, 1108, 997, 970, 770, 741, 698 cm^{-1}. **^{1}H NMR** (500 MHz, $CDCl_3$, 298 K): δ 7.84 (d, *J* = 8.1 Hz, 1H), 7.78 (d, *J* = 8.4 Hz, 1H), 7.74 (d, *J* = 6.9 Hz, 1H), 7.70–7.60 (m, 6H), 7.53–7.47 (m, 4H), 7.46–7.37 (m, 9H), 7.02 (d, *J* = 15.5 Hz, 1H), 6.35 (dt, *J* = 15.7, 8.0 Hz, 1H), 2.73 (d, *J* = 8.1 Hz, 2H). **^{13}C{^{1}H} NMR** (126 MHz, $CDCl_3$, 298 K): δ 136.3, 136.0, 134.6, 133.7, 131.2, 129.8, 129.4, 128.4, 128.1, 128.0, 127.0, 125.8, 125.7, 124.4, 123.5, 21.2. **^{1}H/^{29}Si HMQC NMR** (500/99 MHz, $CDCl_3$, 298 K, optimized for *J* = 7 Hz): δ 7.68/–13.29, 2.73/–13.29. **HRMS (APCI)**: calculated for $C_{31}H_{27}Si^+$ $[M–H]^+$: 427.1877; Found 427.1870.

(*E*)-(3-(Naphthalen-2-yl)allyl)triphenylsilane (**3ra**)

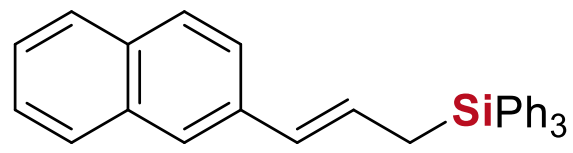

3ra

Allylsilane **3ra** was prepared from 2-allylnaphthalene (**1r**, 50.5 mg, 0.30 mmol, 1.5 equiv) and allyltriphenylsilane (**5a**, 60.1 mg, 0.20 mmol, 1.0 equiv) using $(COD)Pd(CH_2SiMe_3)_2$ (1.6 mg, 2.0 mol%), *o*-Tol_3P (1.8 mg, 3.0 mol%) and $[Me_3Si(HCB_{11}H_5Br_6)]$ (4.2 mg, 3.0 mol%) in CH_2Cl_2 (1.0 mL) at room temperature following **GP 3** (**3ra**:**4ra** > 95:5 from the crude ^{1}H NMR spectrum). Flash column chromatography on silica gel using *n*-pentane/dichloromethane as the eluent and subsequent crystallization from *n*-hexane/ethyl acetate afforded allylsilane **3ra** as a white solid (63.1 mg, 74% yield).

R*f* = 0.45 (*n*-pentane/dichloromethane = 6:1). **m. p.** = 80 °C. **IR** (ATR): ṽ = 3046, 3002, 2685, 2113, 1624, 1588, 1484, 1425, 1153, 1107, 957, 809, 784, 715, 697 cm^{-1}. **^{1}H NMR** (500 MHz, $CDCl_3$, 298 K): δ 7.76 (t, *J* = 7.9 Hz, 2H), 7.71 (d, *J* = 8.5 Hz, 1H), 7.62–7.52 (m, 7H), 7.49–7.35 (m, 12H), 6.92–5.88 (m, 2H), 2.63 (d, *J* = 6.4 Hz, 2H). **^{13}C{^{1}H} NMR** (126 MHz, $CDCl_3$, 298 K): δ 135.9, 135.8, 135.4, 134.6, 133.9, 132.6, 130.6, 129.8, 128.1, 127.99, 127.7, 126.9, 126.2, 125.4, 124.9, 123.7, 20.8. **^{1}H/^{29}Si HMQC NMR** (500/99 MHz, $CDCl_3$, 298 K, optimized for *J* = 7 Hz): δ 7.60/–13.56, 2.63/–13.56. **HRMS (APCI)**: calculated for $C_{31}H_{27}Si^+$ $[M–H]^+$: 427.1877; Found 427.1869.

(*E*)-Triphenyl(3-(thiophen-2-yl)allyl)silane (**3sa**)

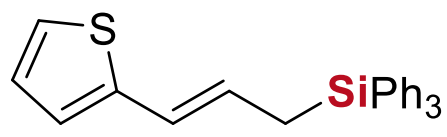

3sa

Allylsilane **3sa** was prepared from 2-allylthiophene (**1s**, 37.3 mg, 0.30 mmol, 1.5 equiv) and allyltriphenylsilane (**5a**, 60.1 mg, 0.20 mmol, 1.0 equiv) using (COD)Pd(CH_2SiMe_3)$_2$ (1.6 mg, 2.0 mol%), *o*-Tol$_3$P (1.8 mg, 3.0 mol%) and [$Me_3Si(HCB_{11}H_5Br_6)$] (4.2 mg, 3.0 mol%) in CH_2Cl_2 (1.0 mL) at room temperature following **GP 3** (**3sa**:**4sa** > 95:5 from the crude ^{1}H NMR spectrum). Flash column chromatography on silica gel using *n*-pentane/dichloromethane as the eluent afforded allylsilane **3sa** as a white solid (26.7 mg, 35% yield).

R$_f$ = 0.45 (*n*-pentane/dichloromethane = 6:1). **m. p.** = 32 °C. **IR** (ATR): ṽ = 3066, 3013, 2912, 2689, 1957, 1887, 1701, 1587, 1484, 1425, 1262, 1107, 951, 778, 732, 692 cm^{-1}. **^{1}H NMR** (500 MHz, $CDCl_3$, 298 K): δ 7.63–7.52 (m, 6H), 7.48–7.34 (m, 9H), 7.03 (d, *J* = 5.0 Hz, 1H), 6.95–6.87 (m, 1H), 6.74 (d, *J* = 3.5 Hz, 1H), 6.39 (d, *J* = 15.6 Hz, 1H), 6.14 (dt, *J* = 15.8, 8.1 Hz, 1H), 2.52 (d, *J* = 8.1 Hz, 2H). **^{13}C{^{1}H} NMR** (126 MHz, $CDCl_3$, 298 K): δ 143.6, 135.9, 134.4, 129.8, 128.1, 127.3, 126.5, 123.6, 123.5, 122.8, 20.4. **^{1}H/^{29}Si HMQC NMR** (500/99 MHz, $CDCl_3$, 298 K, optimized for *J* = 7 Hz): δ 7.58/–13.71, 2.52/–13.71. **HRMS (APCI)**: calculated for $C_{25}H_{23}SSi^+$ [M–H]$^+$: 383.1284; Found 383.1281.

(*E*)-Triphenyl(3-(thiophen-3-yl)allyl)silane (**3ta**)

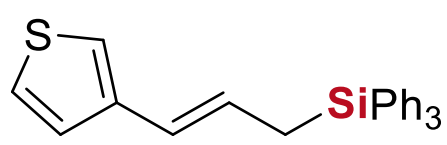

3ta

Allylsilane **3ta** was prepared from 3-allylthiophene (**1t**, 37.3 mg, 0.30 mmol, 1.5 equiv) and allyltriphenylsilane (**5a**, 60.1 mg, 0.20 mmol, 1.0 equiv) using (COD)Pd(CH_2SiMe_3)$_2$ (1.6 mg, 2.0 mol%), *o*-Tol$_3$P (1.8 mg, 3.0 mol%) and [$Me_3Si(HCB_{11}H_5Br_6)$] (4.2 mg, 3.0 mol%) in CH_2Cl_2 (1.0 mL) at room temperature following **GP 3** (**3ta**:**4ta** > 95:5 from the crude ^{1}H NMR spectrum). Flash column chromatography on silica gel using *n*-pentane/dichloromethane as the eluent afforded allylsilane **3ta** as a white solid (35.2 mg, 46% yield).

R$_f$ = 0.45 (*n*-pentane/dichloromethane = 6:1). **m. p.** = 80 °C. **IR** (ATR): ṽ = 3066, 3010, 2914, 1958, 1888, 1642, 1462, 1157, 1109, 960, 833, 772, 698 cm^{-1}. **^{1}H NMR** (500 MHz, $CDCl_3$, 298 K): δ 7.56 (d, *J* = 6.4 Hz, 6H), 7.49–7.40 (m, 3H), 7.42–7.35 (m, 6H), 7.19 (dd, *J* = 5.1, 3.0 Hz, 1H), 7.04 (d, *J* = 5.0 Hz, 1H), 6.92 (d, *J* = 2.8 Hz, 1H), 6.31 (d, *J* = 15.7 Hz, 1H), 6.13

(dt, J = 15.8, 8.0 Hz, 1H), 2.52 (d, J = 8.0 Hz, 2H). **$^{13}C\{^1H\}$ NMR** (126 MHz, $CDCl_3$, 298 K): δ 140.9, 135.9, 134.6, 129.7, 128.0, 126.2, 125.6, 125.1, 124.9, 119.7, 20.3. **$^1H/^{29}Si$ HMQC NMR** (500/99 MHz, $CDCl_3$, 298 K, optimized for J = 7 Hz): δ 7.45/–13.56, 2.52/–13.56. **HRMS (APCI)**: calculated for $C_{25}H_{23}SSi^+$ $[M–H]^+$: 383.1284; Found 383.1281.

(*E*)-Cinnamyltrimethylsilane (**3ab**)

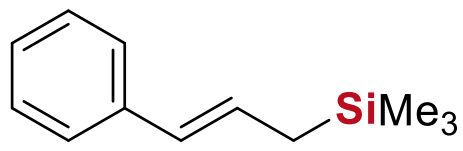

3ab

Allylsilane **3ab** was prepared from allylbenzene (**1a**, 23.7 mg, 0.20 mmol, 1.0 equiv) and allyltrimethylsilane (**5b**, 34.3 mg, 0.30 mmol, 1.5 equiv) using (COD)Pd($CH_2SiMe_3)_2$ (1.6 mg, 2.0 mol%), *o*-Tol_3P (1.8 mg, 3.0 mol%) and [$Me_3Si(HCB_{11}H_5Br_6)$] (4.2 mg, 3.0 mol%) in CH_2Cl_2 (1.0 mL) at room temperature following **GP 3** (**3ab**:**4ab** > 95:5 from the crude ^1H NMR spectrum). Flash column chromatography on silica gel using *n*-pentane as the eluent afforded allylsilane **3ab** as a colorless oil (33.9 mg, 89% yield).

R_f = 0.80 (*n*-pentane). **IR** (ATR): ṽ = 3016, 2953, 2895, 1645, 1599, 1506, 1402, 1227, 1153, 1092, 962, 838, 765, 694 cm^{-1}. **^1H NMR** (500 MHz, $CDCl_3$, 298 K): δ 7.32–7.28 (m, 4H), 7.17–7.14 (m, 1H), 6.25–6.24 (m, 2H), 1.68 (d, J = 6.5 Hz, 2H), 0.06 (s, 9H). **$^{13}C\{^1H\}$ NMR** (126 MHz, $CDCl_3$, 298 K): δ 138.7, 128.6, 128.4, 128.0, 126.3, 125.7, 24.1, –1.7. **$^1H/^{29}Si$ HMQC NMR** (500/99 MHz, $CDCl_3$, 298 K, optimized for J = 7 Hz): δ 1.62/0.95, 0.00/0.95. **HRMS (APCI)**: calculated for $C_{12}H_{19}Si^+$ $[M–H]^+$: 191.1251; Found 191.1252.

(*E*)-(3-(4-Fluorophenyl)allyl)trimethylsilane (**3gb**)

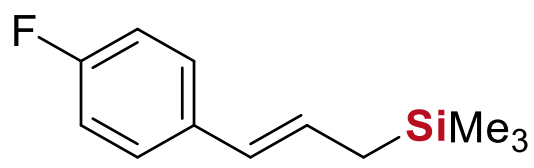

3gb

Allylsilane **3gb** was prepared from 4-fluoroallylbenzene (**1g**, 27.3 mg, 0.20 mmol, 1.0 equiv) and allyltrimethylsilane (**5b**, 34.3 mg, 0.30 mmol, 1.5 equiv) using (COD)Pd($CH_2SiMe_3)_2$ (1.6 mg, 2.0 mol%), *o*-Tol_3P (1.8 mg, 3.0 mol%) and [$Me_3Si(HCB_{11}H_5Br_6)$] (4.2 mg, 3.0 mol%) in CH_2Cl_2 (1.0 mL) at room temperature following **GP 3** (**3gb**:**4gb** > 95:5 from the crude ^1H NMR spectrum). Flash column chromatography on silica gel using *n*-pentane as the eluent afforded allylsilane **3gb** as a colorless oil (36.2 mg, 87% yield).

R_f = 0.80 (*n*-pentane). **IR** (ATR): ṽ = 3022, 2954, 2917, 2870, 1973, 1866, 1641, 1599, 1460,

1408, 1376, 1194, 1145, 1080, 884, 740, 693 cm^{-1}. **^{1}H NMR** (500 MHz, $CDCl_3$, 298 K): δ 7.26–7.23 (m, 2H), 6.96 (t, *J* = 8.7 Hz, 2H), 6.21–6.14 (m, 2H), 1.65 (d, *J* = 7.4 Hz, 2H), 0.05 (s, 9H). **^{13}C{^{1}H} NMR** (126 MHz, $CDCl_3$, 298 K): δ 161.7 (d, $J_{C,F}$ = 245.2 Hz), 134.8 (d, *J* = 3.5 Hz), 127.7, 127.3, 127.0, 126.9, 115.5, 115.3, 24.0, –1.7. **^{19}F NMR** (471 MHz, $CDCl_3$) δ –116.8. **^{1}H/^{29}Si HMQC NMR** (500/99 MHz, $CDCl_3$, 298 K, optimized for *J* = 7 Hz): δ 1.65/0.92, 0.05/0.92. **HRMS (APCI)**: calculated for $C_{12}H_{18}FSi^{+}$ [M–H]$^{+}$: 209.1156; Found 209.1159.

(*E*)-Tributyl(cinnamyl)silane (**3ac**)

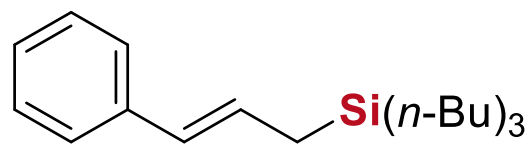

3ac

Allylsilane **3ac** was prepared from allylbenzene (**1a**, 23.7 mg, 0.20 mmol, 1.0 equiv) and allyltributylsilane (**5c**, 72.2 mg, 0.30 mmol, 1.5 equiv) using (COD)Pd(CH_2SiMe_3)$_2$ (1.6 mg, 2.0 mol%), *o*-Tol_3P (1.8 mg, 3.0 mol%) and [$Me_3Si(HCB_{11}H_5Br_6)$] (4.2 mg, 3.0 mol%) in CH_2Cl_2 (1.0 mL) at room temperature following **GP 3** (**3ac**:**4ac** > 95:5 from the crude ^{1}H NMR spectrum). Flash column chromatography on silica gel using *n*-pentane as the eluent afforded allylsilane **3ac** as a colorless oil (32.9 mg, 52% yield).

R_f = 0.75 (*n*-pentane). **IR** (ATR): ṽ = 2955, 2917, 2869, 1897, 1640, 1512, 1460, 1408, 1268, 1145, 1080, 1014, 961, 884, 758, 722 cm^{-1}. **^{1}H NMR** (500 MHz, $CDCl_3$, 298 K): δ 7.29–7.26 (m, 4H), 7.17–7.15 (m, 1H), 6.24 (d, *J* = 5.0 Hz, 2H), 1.71 (d, *J* = 5.2 Hz, 2H), 1.35–1.33 (m, 12H), 0.90 (t, *J* = 6.8 Hz, 9H), 0.57–0.54 (m, 6H). **^{13}C{^{1}H} NMR** (126 MHz, $CDCl_3$, 298 K): δ 138.8, 128.6, 128.4, 128.2, 126.3, 125.6, 26.9, 26.2, 20.1, 13.9, 12.2. **^{1}H/^{29}Si HMQC NMR** (500/99 MHz, $CDCl_3$, 298 K, optimized for *J* = 7 Hz): δ 1.69/2.95, 0.56/2.95. **HRMS (APCI)**: calculated for $C_{21}H_{37}Si^{+}$ [M–H]$^{+}$: 317.2659; Found 317.2657.

(*E*)-Tributyl(3-(4-(tert-butyl)phenyl)allyl)silane (**3cc**)

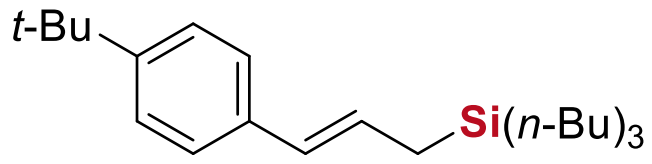

3cc

Allylsilane **3cc** was prepared from 1-allyl-4-(*tert*-butyl)benzene (**1c**, 34.9 mg, 0.20 mmol, 1.0 equiv) and allyltributylsilane (**5c**, 72.2 mg, 0.30 mmol, 1.5 equiv) using (COD)Pd(CH_2SiMe_3)$_2$ (1.6 mg, 2.0 mol%), *o*-Tol_3P (1.8 mg, 3.0 mol%) and [$Me_3Si(HCB_{11}H_5Br_6)$] (4.2 mg, 3.0 mol%) in CH_2Cl_2 (1.0 mL) at room temperature following **GP 3** (**3cc**:**4cc** > 95:5 from the crude ^{1}H

NMR spectrum). Flash column chromatography on silica gel using *n*-pentane as the eluent and subsequent crystallization from *n*-hexane/ethyl acetate afforded allylsilane **3cc** as a colorless oil (41.0 mg, 55% yield).

R*f* = 0.70 (*n*-pentane). **IR** (ATR): ṽ = 3059, 3022, 2953, 1641, 1598, 1494, 1447, 1405, 1246, 1146, 1020, 960, 858, 739, 693 cm^{-1}. **^{1}H NMR** (500 MHz, $CDCl_3$, 298 K): δ 7.32 (d, *J* = 8.4 Hz, 2H), 7.26–7.23 (m, 2H), 6.28–6.16 (m, 2H), 1.69 (d, *J* = 6.6 Hz, 2H), 1.52–1.28 (m, 21H), 0.92–0.85 (m, 9H), 0.59–0.55 (m, 6H). **^{13}C{^{1}H} NMR** (126 MHz, $CDCl_3$, 298 K): δ 149.2, 136.1, 128.4, 128.0, 127.5, 125.5, 125.3, 125.1, 34.6, 26.9, 26.2, 20.0, 13.9, 12.2. **^{1}H/^{29}Si HMQC NMR** (500/99 MHz, $CDCl_3$, 298 K, optimized for *J* = 7 Hz): δ 1.69/2.91, 0.58/2.91. **HRMS (APCI)**: calculated for $C_{25}H_{44}Si^+$ $[M]^+$:372.3207; Found 372.3204.

(*E*)-Benzyl(cinnamyl)dimethylsilane (**3ad**)

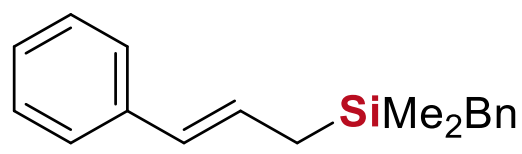

3ad

Allylsilane **3ad** was prepared from allylbenzene (**1a**, 35.5 mg, 0.30 mmol, 1.5 equiv) and allyl(methyl)diphenysilane (**5d**, 38.1 mg, 0.20 mmol, 1.0 equiv) using (COD)Pd(CH_2SiMe_3)$_2$ (1.6 mg, 2.0 mol%), *o*-Tol_3P (1.8 mg, 3.0 mol%) and [$Me_3Si(HCB_{11}H_5Br_6)$] (4.2 mg, 3.0 mol%) in CH_2Cl_2 (1.0 mL) at room temperature following **GP 3** (**3ad**:**4ad** > 95:5 from the crude ^{1}H NMR spectrum). Flash column chromatography on silica gel using *n*-pentane as the eluent afforded allylsilane **3ad** as a colorless oil (28.2 mg, 53% yield).

R*f* = 0.75 (pentane/dichloromethane = 50/1). **IR** (ATR): ṽ = 3060, 3023, 2955, 1641, 1598, 1493, 1450, 1250, 1207, 1155, 1056, 962, 907, 821, 731 cm^{-1}. **^{1}H NMR** (500 MHz, $CDCl_3$, 298 K): δ 7.29 (d, *J* = 4.3 Hz, 4H), 7.23 (t, *J* = 7.4 Hz, 2H), 7.21–7.15 (m, 1H), 7.10 (t, *J* = 7.6 Hz, 1H), 7.03 (d, *J* = 7.4 Hz, 2H), 6.41–5.69 (m, 2H), 2.16 (s, 2H), 1.68 (d, *J* = 7.5 Hz, 2H), 0.04 (s, 3H). **^{13}C{^{1}H} NMR** (126 MHz, $CDCl_3$, 298 K): δ 140.0, 138.5, 128.9, 128.6, 128.4, 128.3, 127.3, 126.4, 125.7, 124.2, 25.5, 22.2, –3.6 (two overlapping signals). **^{1}H/^{29}Si HMQC NMR** (500/99 MHz, $CDCl_3$, 298 K, optimized for *J* = 7 Hz): δ 2.16/1.50, 1.68/1.50, 0.04/1.50. **HRMS (APCI)**: calculated for $C_{17}H_{21}Si^+$ $[M–H]^+$: 267.1564; Found 267.1560.

(E)-Cinnamyldimethyl(phenyl)silane (**3ae**)

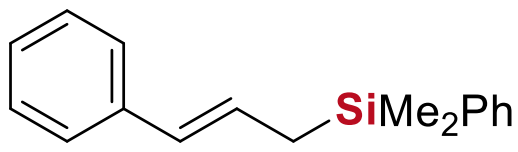

3ae

Allylsilane **3ae** was prepared from allylbenzene (**1a**, 35.5 mg, 0.30 mmol, 1.5 equiv) and allyldimethyl(phenyl)silane (**5e**, 35.3 mg, 0.20 mmol, 1.0 equiv) using (COD)Pd(CH_2SiMe_3)$_2$ (1.6 mg, 2.0 mol%), *o*-Tol_3P (1.8 mg, 3.0 mol%) and [$Me_3Si(HCB_{11}H_5Br_6)$] (4.2 mg, 3.0 mol%) in CH_2Cl_2 (1.0 mL) at room temperature following **GP 3** (**3ae**:**4ae** > 95:5 from the crude ^{1}H NMR spectrum). Flash column chromatography on silica gel using *n*-pentane/dichloromethane as the eluent afforded allylsilane **3ae** as a colorless oil (39.4 mg, 78% yield).

R*f* = 0.45 (pentane/dichloromethane = 6/1). **IR** (ATR): ṽ = 3022, 2955, 2919, 1249, 1641, 1599, 1494, 1426, 1249, 1147, 1113, 1021, 962, 815, 733, 696 cm^{-1}. **^{1}H NMR** (500 MHz, $CDCl_3$, 298 K): δ 7.57–7.55 (m, 2H), 7.40–7.34 (m, 3H), 7.30–7.29 (m, 4H), 7.20–7.16 (m, 1H), 6.30–6.21 (m, 2H), 1.94 (d, J = 6.8 Hz, 2H), 0.37 (s, 6H). **^{13}C{^{1}H} NMR** (126 MHz, $CDCl_3$, 298 K): δ 138.7, 138.6, 133.8, 129.2, 129.1, 128.6, 128.0, 127.3, 126.4, 125.7, 23.2, –3.1. **^{1}H/^{29}Si HMQC NMR** (500/99 MHz, $CDCl_3$, 298 K, optimized for J = 7 Hz): δ 7.56/–4.33, 6.28/–4.33, 1.94/–4.33, 0.38/–4.33. **HRMS (APCI)**: calculated for $C_{17}H_{21}Si^+$ [M–H]$^+$: 253.1407; Found 253.1405.

(E)-Cinnamyl(methyl)diphenylsilane (**3af**)

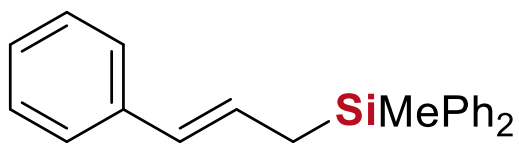

3af

Allylsilane **3af** was prepared from allylbenzene (**1a**, 35.5 mg, 0.30 mmol, 1.5 equiv) and allyl(methyl)diphenysilane (**5f**, 47.7 mg, 0.20 mmol, 1.0 equiv) using (COD)Pd(CH_2SiMe_3)$_2$ (1.6 mg, 2.0 mol%), *o*-Tol_3P (1.8 mg, 3.0 mol%) and [$Me_3Si(HCB_{11}H_5Br_6)$] (4.2 mg, 3.0 mol%) in CH_2Cl_2 (1.0 mL) at room temperature following **GP 3** (**3af**:**4af** > 95:5 from the crude ^{1}H NMR spectrum). Flash column chromatography on silica gel using *n*-pentane/dichloromethane as the eluent afforded allylsilane **3af** as a colorless oil (42.7 mg, 68% yield).

R*f* = 0.50 (pentane/dichloromethane = 50/1). **IR** (ATR): ṽ = 3067, 3022, 2921, 2247, 1640, 1594, 1488, 1252, 1148, 1111, 1009, 962, 906, 852, 791, 728 cm^{-1}. **^{1}H NMR** (500 MHz,

$CDCl_3$, 298 K): δ 7.57 (d, *J* = 5.2 Hz, 4H), 7.42–7.37 (m, 6H), 7.29–7.26 (m, 4H), 7.18–7.17 (m, 1H), 6.28–6.22 (m, 2H), 2.25 (d, *J* = 7.5 Hz, 2H), 0.62 (s, 3H). **$^{13}C\{^1H\}$ NMR** (126 MHz, $CDCl_3$, 298 K): δ 138.4, 136.6, 134.7, 129.8, 129.6, 128.0, 126.7, 126.5, 125.8, 21.6, –4.4. **$^1H/^{29}Si$ HMQC NMR** (500/99 MHz, $CDCl_3$, 298 K, optimized for *J* = 7 Hz): δ 6.15/–9.40, 2.14/–9.40. **HRMS (APCI)**: calculated for $C_{17}H_{21}Si^+$ $[M–H]^+$: 315.1564; Found 315.1562.

(*E*, *E*)-Dicinnamyldimethylsilane (**3ah**)

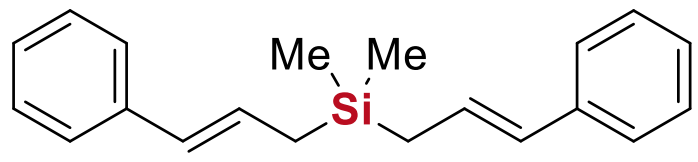

3ah

Bis(cinnamyl)silane **3ah** was prepared from allylbenzene (**1a**, 58.1 mg, 0.50 mmol, 2.5 equiv) and diallyldimethylsilane (**5h**, 38.1 mg, 0.20 mmol, 1.0 equiv) using $(COD)Pd(CH_2SiMe_3)_2$ (1.6 mg, 2.0 mol%), *o*-Tol_3P (1.8 mg, 3.0 mol%) and $[Me_3Si(HCB_{11}H_5Br_6)]$ (4.2 mg, 3.0 mol%) in CH_2Cl_2 (1.0 mL) at room temperature following **GP 3**. Flash column chromatography on silica gel using *n*-pentane as the eluent afforded bis(cinnamyl)silane **3ah** as a colorless oil (42.1 mg, 72% yield).

R_f = 0.50 (pentane/dichloromethane = 50/1). **IR** (ATR): ṽ = 3059, 3022, 2954, 2907, 2126, 1995, 1641, 1494, 1249, 1149, 1072, 1019, 961, 908, 860, 735, 694 cm^{-1}. **^1H NMR** (500 MHz, $CDCl_3$, 298 K): δ 7.24–7.18 (m, 8H), 7.15–7.08 (m, 2H), 6.42–5.91 (m, 4H), 2.38–1.37 (m, 4H), 0.05 (s, 6H). **$^{13}C\{^1H\}$ NMR** (126 MHz, $CDCl_3$, 298 K): δ 138.5, 129.0, 128.6, 128.6, 127.3, 126.5, 125.7, 22.5, –3.5. **$^1H/^{29}Si$ HMQC NMR** (500/99 MHz, $CDCl_3$, 298 K, optimized for *J* = 7 Hz): δ 1.86/1.89, 0.05/1.89. **HRMS (APCI)**: calculated for $C_{20}H_{25}Si^+$ $[M–H]^+$: 293.1720; Found 293.1718.

5 Experimental Details for the Independent Preparation and Characterization of the Key Intermediates

5.1 Preparation of (COD)Pd(CH_2SiMe_3)$_2$ [CAS: 225931-80-6]

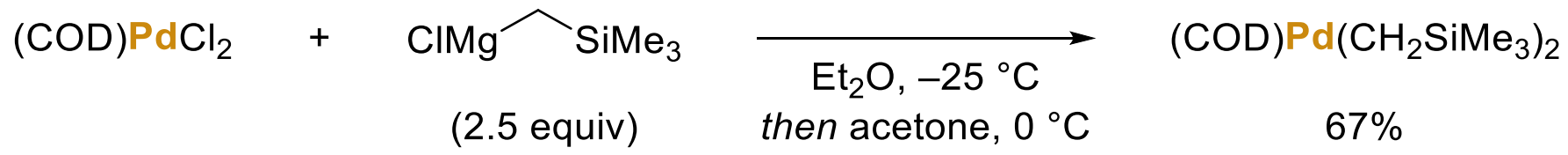

(COD)Pd(CH_2SiMe_3)$_2$ was prepared according to a reported procedure with minor modifications [106, 107]. An oven-dried 50-mL Schlenk flask equipped with a magnetic stir bar was charged with (COD)PdCl_2 (1.00 g, 3.60 mmol, 1.0 equiv) inside an argon-filled glovebox. The flask was sealed, removed from the glovebox, and connected to a Schlenk line under nitrogen atmosphere. Anhydrous diethyl ether (20 mL) was added via syringe, and the resulting yellow suspension was cooled to –25 °C. Freshly prepared Me_3SiCH_2MgCl (9.0 mL of a 1.0 M solution in Et_2O, 9.0 mmol, 2.5 equiv) was added dropwise over 10 min. During the addition, the suspension gradually darkened. Upon completion of the addition, the cooling bath was removed, and the mixture was allowed to warm to 0 °C and stirred for additional 2 h at this temperature, resulting in a deep brown suspension. Acetone (0.50 mL, 6.8 mmol) was then added dropwise via syringe at 0 °C, whereupon a black suspension formed within 1–2 min. After stirring the reaction mixture for additional 5 min, all volatiles were removed under high vacuum at 0 °C. Dry *n*-hexane (50 mL) was added to the resulting black residue, and the mixture was vigorously agitated at 0 °C to ensure efficient extraction. The suspension was filtered through a medium-porosity frit precooled to 0 °C into a second precooled Schlenk flask. The filter cake was washed with cold *n*-pentane (2 × 25 mL). The combined filtrates were concentrated under reduced pressure and further dried under high vacuum at 0 °C for 2 h to afford (COD)Pd(CH_2SiMe_3)$_2$ as a gray solid (0.90 g, 2.35 mmol, 67% yield). The complex was immediately transferred into an inert-atmosphere glovebox and stored at –30 °C to minimize any decomposition.

^{1}H NMR (500 MHz, C_6D_6, 298 K): δ 5.15 (s, 4H), 2.53–1.51 (m, 8H), 0.77 (s, 4H), 0.33 (s, 18H). **^{1}H/^{29}Si HMQC NMR** (500/99 MHz, C_6D_6, 298 K, optimized for J = 7 Hz): δ 0.77/–0.15, 0.33/–0.15. **^{13}C NMR** (126 MHz, C_6D_6, 298 K): δ 112.5, 29.6, 12.1, 4.3. The spectroscopic data are in accordance with those reported in the literature [106, 107].

5.2 Preparation of (o-Tol$_3$P)$_2$Pd (8) [69861-71-8]

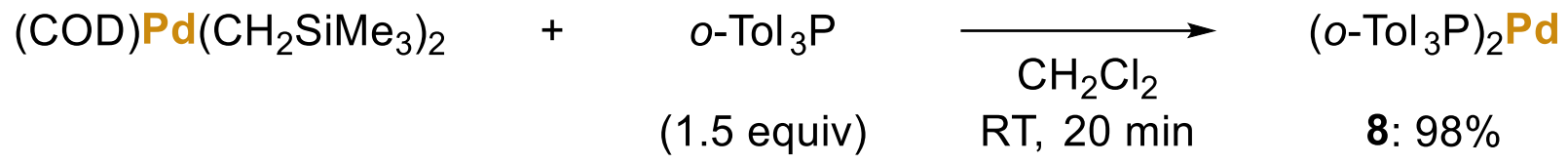

In an argon-filled glovebox, an oven-dried vial was charged with (COD)Pd(CH_2SiMe_3)$_2$ (191.5 mg, 0.5 mmol, 1.0 equiv) in anhydrous CH_2Cl_2 (1.0 mL, 0.5 M), followed by the addition of tri(*o*-tolyl)phosphine (228.3 mg, 0.75 mmol, 1.5 equiv) under vigorous stirring. The reaction mixture was stirred at room temperature for 20 min, whereupon a large amount of a bright yellow solid precipitated. *n*-Hexane (1.0 mL) was added, and the resulting suspension was filtered. The obtained solid was washed with *n*-hexane (3 × 0.5 mL) and dried under vacuum to afford (*o*-Tol$_3$P)$_2$Pd (**8**) as a yellow solid (263.6 mg, 98% yield).

^{1}H NMR (500 MHz, C_6D_6, 298 K): δ 7.07–7.05 (m, 18H), 6.85–6.82 (m, 6H), 2.97 (s, 18H). **^{31}P{^{1}H} NMR** (202 MHz, C_6D_6, 298 K) δ –6.81. The spectroscopic data are in accordance with those reported in the literature [108].

5.3 Preparation of [(o-Tol$_3$P)$_2$PdSiMe$_3$][HCB$_{11}$H$_5$Br$_6$] (9b)

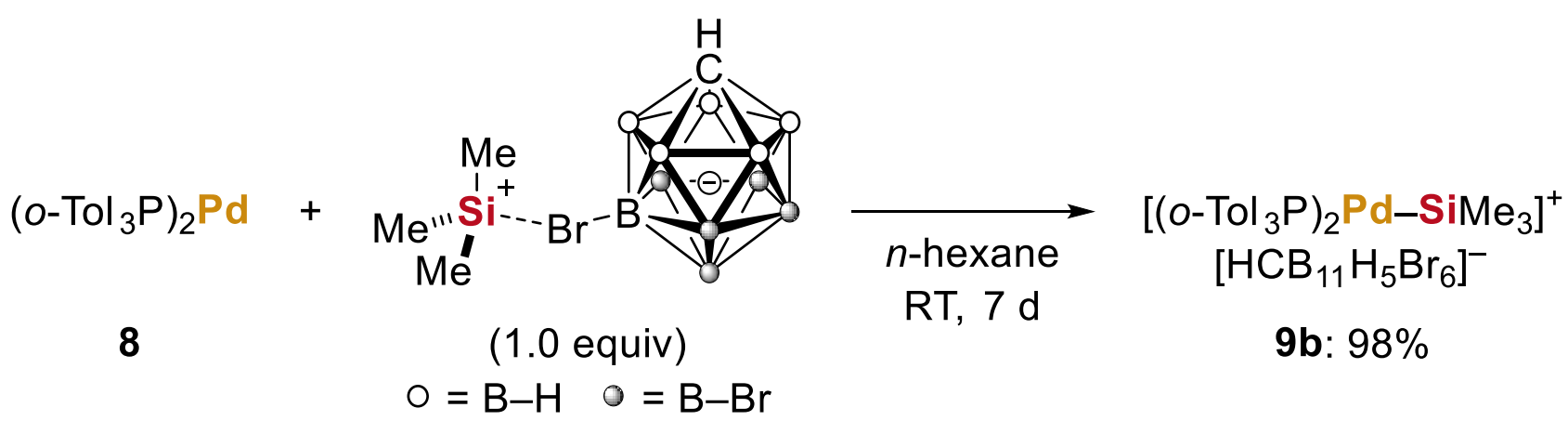

In an argon-filled glovebox, an oven-dried vial was charged with a suspension of [$Me_3Si(HCB_{11}H_5Br_6)$] (28.8 mg, 0.04 mmol) in anhydrous *n*-hexane (1.0 mL). Freshly prepared (*o*-Tol$_3$P)$_2$Pd (**8**, 27.6 mg, 0.04 mmol) was added in portions under vigorous stirring, and the resulting reaction mixture was stirred at room temperature for 7 days. After completion, additional *n*-hexane was added (1.0 mL), and the suspension was filtered. The obtained solid was washed with *n*-hexane (3 × 0.5 mL) and dried under vacuum to afford silylpalladium complex **9b** as a deep-yellow solid (55.1 mg, 98% yield).

^{1}H NMR (500 MHz, CD_2Cl_2, 223 K): δ 8.27 (d, J = 8.1 Hz, 2H), 7.59–7.56 (m, 3H), 7.53–7.52 (m, 2H), 7.44–7.43 (m, 2H), 7.39–7.36 (m, 2H), 7.28–7.13 (m, 11H), 6.91 (d, J = 7.5 Hz, 2H), 3.01 (s, 6H), 2.67 (s, 1H, [$HCB_{11}H_5Br_6$]$^-$), 2.42–2.29 (m, 5H, [$HCB_{11}H_5Br_6$]$^-$), 1.60 (s, 6H), 1.48 (s, 6H), –0.12 (s, 9H, $SiCH_3$). **^{13}C NMR** (126 MHz, CD_2Cl_2, 223 K): δ 144.3, 143.4 (d, J = 8.8 Hz), 141.9 (t, J = 6.5 Hz), 140.8 (t, J = 7.8 Hz), 140.3 (t, J = 16.8 Hz), 136.4 (d, J = 2.8

Hz), 134.6 (d, J = 12.7 Hz), 133.5, 133.2, 133.0, 132.8 (d, J = 10.6 Hz), 132.5, 132.3, 132.1, 132.0, 131.4, 128.1 (d, J = 13.9 Hz), 127.3, 126.3, 126.2, 126.18, 126.15, 123.12 (q, J = 25.2 Hz), 123.11 (d, J = 24.7 Hz), 111.1 (d, J = 81.9 Hz), 41.3 ($[HCB_{11}H_5Br_6]^-$), 31.7, 24.0 (t, J = 5.8 Hz), 23.74 (t, J = 5.6 Hz), 23.73, 22.4, 21.4 (d, J = 8.4 Hz), 14.1, 7.6 (t, J = 4.6 Hz), 1.5. **$^{31}P\{^1H\}$ NMR** (202 MHz, CD_2Cl_2, 223 K): δ 24.75. **$^1H/^{29}Si$ HMQC NMR** (500/99 MHz, CD_2Cl_2, 223 K, optimized for J = 7 Hz): δ –0.12/70.90. **^{11}B NMR** (160 MHz, CD_2Cl_2, 223 K): δ –1.8 (s, 1B), –9.9 (s, 5B), –20.4 (m, 5B).

5.4 Preparation of [*o*-Tol$_3$PSiMe$_3$][HCB$_{11}$H$_5$Br$_6$] (10b)

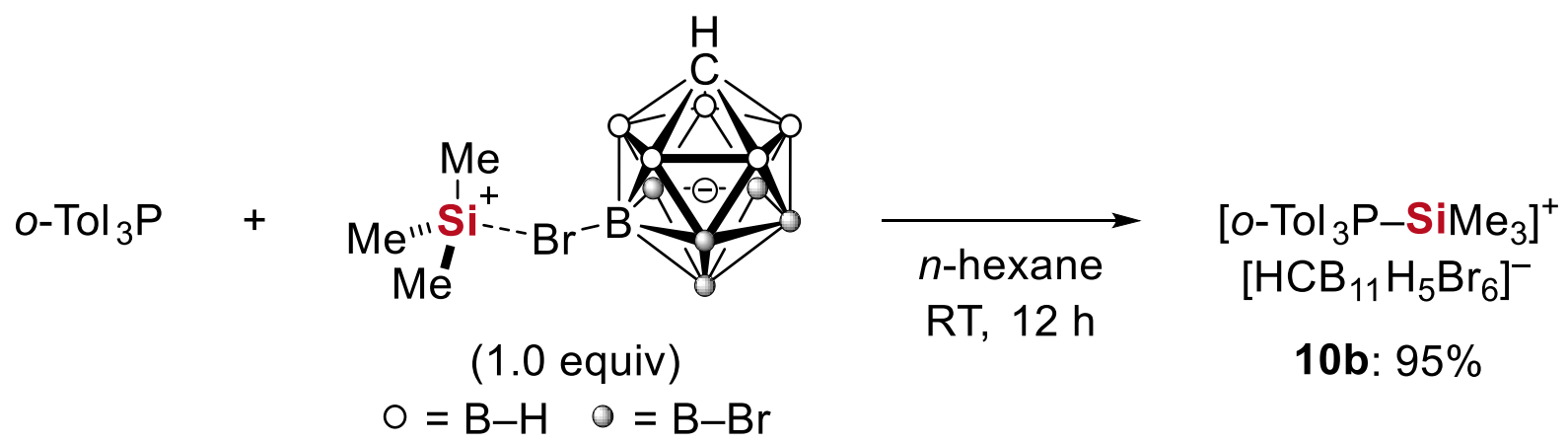

In an argon-filled glove box, [$Me_3Si(HCB_{11}H_5Br_6)$] (41.4 mg, 0.06 mmol) was suspended in *n*-hexane (1.0 mL), followed by the addition of tri(*o*-tolyl)phosphine (18.4 mg, 0.06 mmol). After stirring the reaction mixture for 12 h, additional *n*-hexane (1.0 mL) was added, and the suspension was filtered. The obtained solid was washed with *n*-hexane (3 × 0.5 mL) and dried for 10 min under high vacuum to give silylphosphonium salt **10b** as a white solid (56.6 mg, 95% yield).

^1H NMR (500 MHz, 1,2-$C_6D_4Cl_2$, 298 K): δ 7.22 (t, J = 7.7 Hz, 3H), 7.10 (t, J = 6.5 Hz, 3H), 7.02–6.94 (m, 3H), 6.64 (dd, J = 13.6, 7.9 Hz, 3H), 2.79 (s, 1H, $[HCB_{11}H_5Br_6]^-$), 2.44 (s, 2H, $[HCB_{11}H_5Br_6]^-$), 2.13 (d, J = 49.9 Hz, 3H, $[HCB_{11}H_5Br_6]^-$), 1.88 (s, 9H), 0.24 (d, J = 8.0 Hz, 9H). **$^{31}P\{^1H\}$ NMR** (202 MHz, 1,2-$C_6D_4Cl_2$, 298 K): δ 3.26. **$^1H/^{29}Si$ HMQC NMR** (500/99 MHz, 1,2-$C_6D_4Cl_2$, 298 K, optimized for J = 7 Hz): δ 0.24/20.82. **^{13}C NMR** (126 MHz, 1,2-$C_6D_4Cl_2$, 298 K): δ 134.7 (d, J = 9.4 Hz), 134.6, 133.4 (d, J = 9.7 Hz), 129.0 (d, J = 354.9 Hz), 128.4 (d, J = 10.6 Hz), 116.9 (d, J = 58.6 Hz), 41.4 ($HCB_{11}H_5Br_6$), 23.7 (d, J = 5.7 Hz), 1.0 (d, J = 11.2 Hz).

Single crystals of [*o*-Tol$_3$PSiMe$_3$][HCB$_{11}$H$_5$Br$_6$] (**10b**) suitable for X-ray diffraction analysis were obtained from a solution of silylphosphonium salt **10b** in dichloromethane by diffusion with *n*-hexane in a *J*-Young NMR tube at room temperature overnight (see Section 7.1). CCDC 2538954 contains the supplementary crystallographic data. This data is provided free of charge by The Cambridge Crystallographic Data Centre.

5.5 Preparation of [(o-Tol$_3$P)$_2$Pd(H)(MeCN)][HCB$_{11}$H$_5$Br$_6$] (11·MeCN)

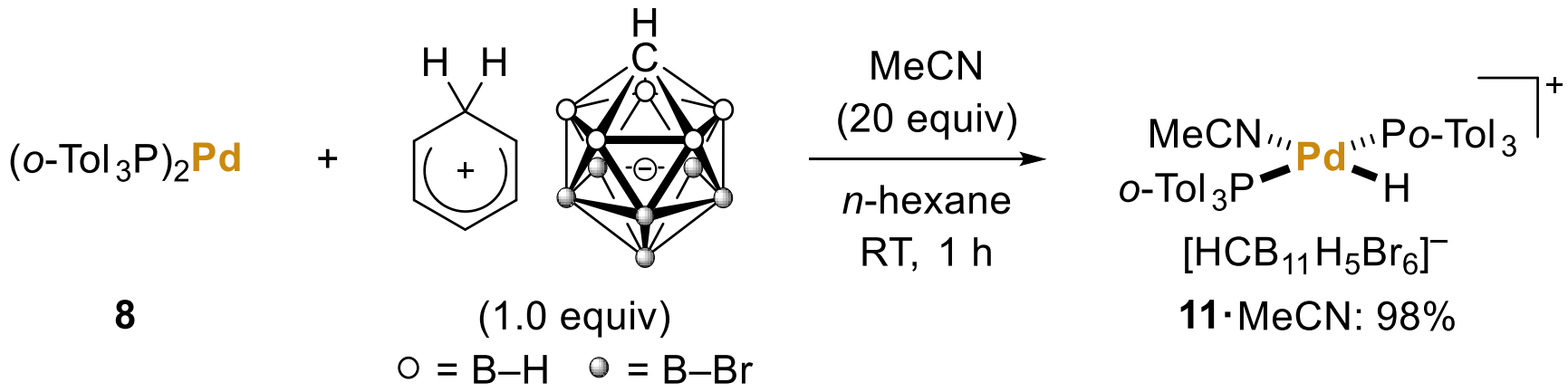

In an argon-filled glovebox, an oven-dried vial was charged with a suspension of benzenium salt [H(C$_6$H$_6$)][HCB$_{11}$H$_5$Br$_6$] (27.8 mg, 0.04 mmol) in *n*-hexane (1.0 mL). Freshly prepared (*o*-Tol$_3$P)$_2$Pd (**8**, 27.6 mg, 0.04 mmol) was added in portions under vigorous stirring, followed by slow addition of MeCN (21.0 μL, 0.4 mmol, 10.0 equiv). The reaction mixture was stirred at room temperature for 1 h. After completion, *n*-hexane was added, and the resulting suspension was filtered. The obtained solid was washed with *n*-hexane (3 × 0.5 mL) and dried under vacuum to afford palladium hydride complex **11**·MeCN as a pale white solid (53.8 mg, 98% yield).

^{1}H NMR (500 MHz, CD$_2$Cl$_2$, 298 K): δ 7.56–7.46 (m, 6H), 7.46–7.32 (m, 12H), 7.31–7.18 (m, 6H), 2.31 (s, 3H, CH$_3$CN), 2.27 (s, 12H), 1.94–1.21 (m, 4H), 0.89 (d, J = 7.4 Hz, 1H), –14.76 (t, $J_{H,P}$ = 12.0 Hz, 1H, Pd–H). **^{13}C NMR** (126 MHz, CD$_2$Cl$_2$, 298 K): δ 142.7 (t, J = 5.4 Hz), 137.0, 134.8 (t, J = 7.6 Hz), 132.6, 132.1, 127.6 (t, J = 23.4 Hz), 127.1 (t, J = 5.3 Hz), 41.6 ([HCB$_{11}$H$_5$Br$_6$]), 34.5, 23.3 (t, J = 4.3 Hz, two overlapping resonances), 22.7, 14.2, 2.3. **^{31}P{^{1}H} NMR** (202 MHz, CD$_2$Cl$_2$, 298 K): δ 17.46. **HRMS (APCI)**: calculated for $C_{42}H_{43}P_2Pd^+$ [M–MeCN]$^+$: 715.1875; Found 715.1872.

Single crystals of *trans*-[(*o*-Tol$_3$P)$_2$Pd(H)(MeCN)][HCB$_{11}$H$_5$Br$_6$] (**11**·MeCN) suitable for X-ray diffraction analysis were obtained from a solution of palladium hydride complex **11**·MeCN in 1,2-dichlorobenzene by diffusion with *n*-hexane in a *J*-Young NMR tube at room temperature overnight (see Section 7.2). CCDC 2538955 contains the supplementary crystallographic data. This data is provided free of charge by The Cambridge Crystallographic Data Centre.

5.6 Preparation of [*o*-Tol$_3$PH][HCB$_{11}$H$_5$Br$_6$] (12)

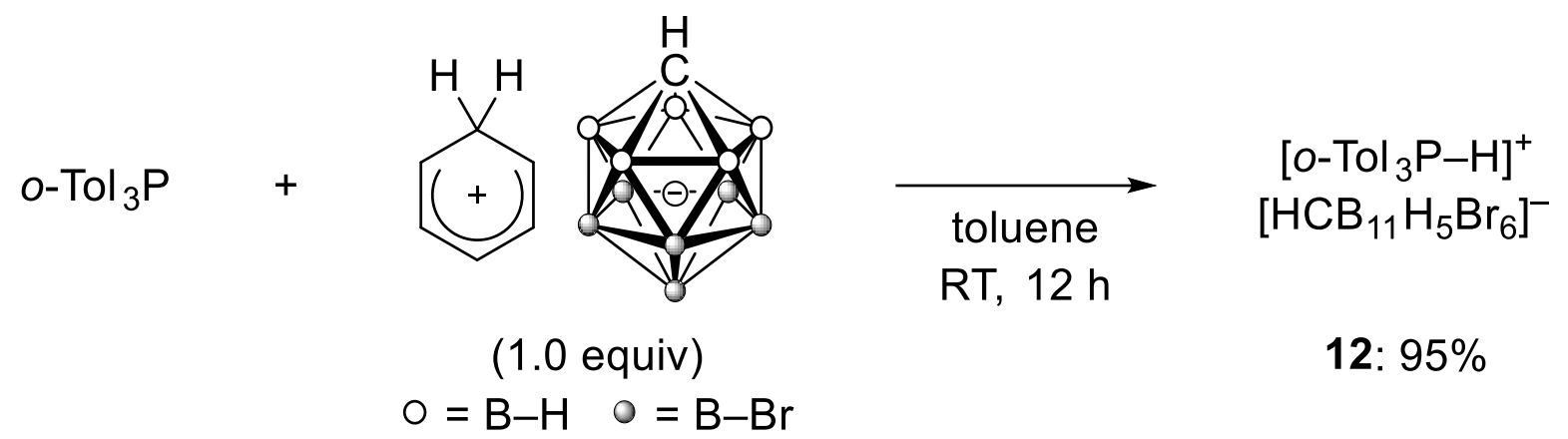

In an argon-filled glove box, an oven-dried vial was charged with a suspension of benzenium salt [H(C$_6$H$_6$)][HCB$_{11}$H$_5$Br$_6$] (41.8 mg, 0.06 mmol) in toluene (1.0 mL), followed by the addition of *o*-Tol$_3$P (18.4 mg, 0.06 mmol). After stirring the reaction mixture for 12 h, *n*-hexane (1.0 mL) was added. The resulting suspension was filtered, and the obtained solid was washed with *n*-hexane (3 × 0.5 mL) and dried for 10 min under high vacuum to give phosphonium salt **12** as a white solid (52.5 mg, 95% yield).

^{1}H NMR (500 MHz, CD$_2$Cl$_2$, 298 K): δ 8.57 (d, $J_{H,P}$ = 485.7 Hz, 1H), 7.84 (t, *J* = 7.7 Hz, 3H), 7.63 (t, *J* = 6.9 Hz, 3H), 7.21 (dd, *J* = 15.9, 7.8 Hz, 3H), 2.83 (s, 1H), 2.58 (s, 2H), 2.47 (s, 9H), 2.02 (m, 3H). **^{31}P{^{1}H} NMR** (202 MHz, CD$_2$Cl$_2$, 298 K): δ –12.31. **^{13}C NMR** (126 MHz, CD$_2$Cl$_2$, 298 K): δ 144.0 (d, *J* = 9.3 Hz), 136.9 (d, *J* = 2.8 Hz), 135.1 (d, *J* = 12.3 Hz), 133.5 (d, *J* = 11.2 Hz), 128.8 (d, *J* = 13.3 Hz), 112.0 (d, *J* = 87.6 Hz), 41.6 (HCB$_{11}$H$_5$Br$_6$), 21.7 (d, *J* = 8.4 Hz).

Single crystals of [*o*-Tol$_3$PH][HCB$_{11}$H$_5$Br$_6$] (**12**) suitable for X-ray diffraction analysis were obtained from a solution of phosphonium salt **12** in dichloromethane by diffusion with *n*-hexane in a *J*-Young NMR tube at room temperature overnight (see Section 7.3). CCDC 2538956 contains the supplementary crystallographic data. This data is provided free of charge by The Cambridge Crystallographic Data Centre.

6 Experimental Mechanistic Study

6.1 Control Experiments on the Isomerization

6.1.1 Isomerization of Allylbenzene (**1a**) under the Standard Reaction Conditions

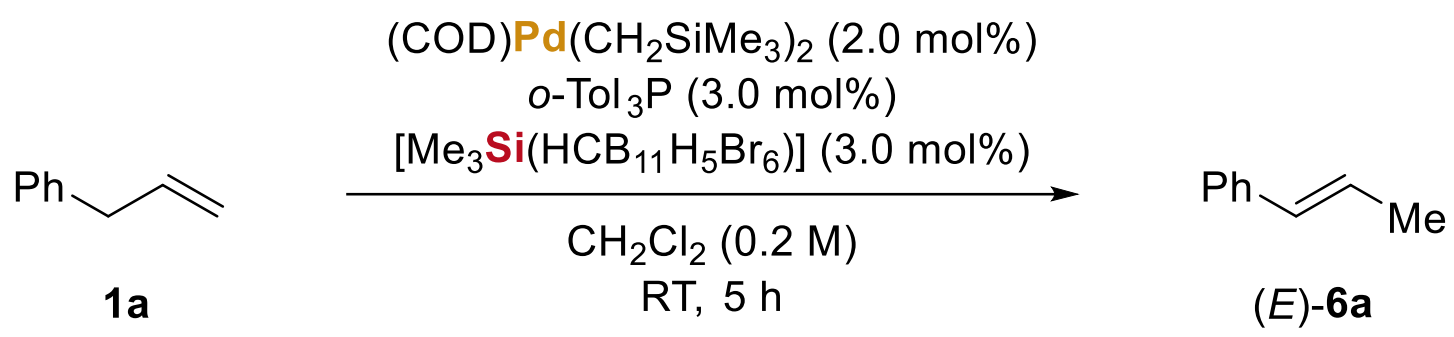

1a (*E*)-**6a**

In an argon-filled glovebox, (COD)Pd(CH_2SiMe_3)$_2$ (3.2 mg, 0.008 mmol, 2.0 mol%) and *o*-Tol$_3$P (3.6 mg, 0.012 mmol, 3.0 mol%) were dissolved in degassed anhydrous CH_2Cl_2 (2.0 mL, 0.2 M). After stirring at room temperature for 1 h, [$Me_3Si(HCB_{11}H_5Br_6)$] (8.4 mg, 0.012 mmol, 3.0 mol%) was added, followed by the addition of allylbenzene (**1a**, 47.3 mg, 0.40 mmol, 1.0 equiv). The resulting reaction mixture was stirred for additional 5 h at room temperature. Upon completion, the mixture was removed from the glovebox and filtered through a short pad of silica gel. The filtrate was concentrated under reduced pressure, and the residue was purified by flash column chromatography on silica gel using *n*-pentane as the eluent to afford the isomerized allylbenzene (*E*)-**6a** (45.4 mg, 96% yield).

6.1.2 Reactivity of Internal Alkene (*E*)-**6a** under the Standard Reaction Conditions

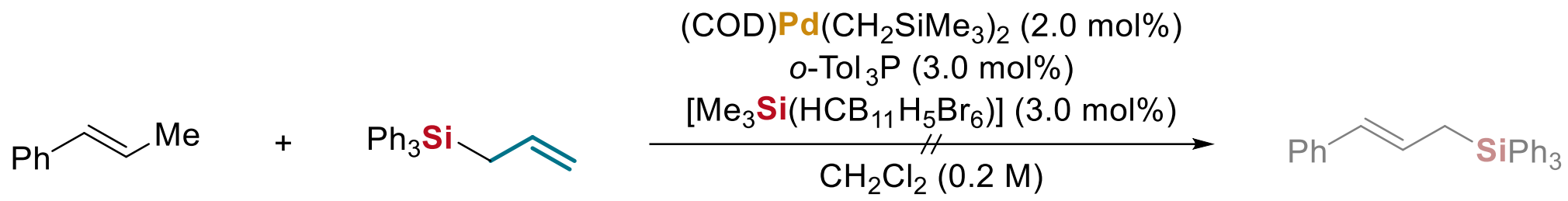

(*E*)-**6a** **5a** **3aa**

Alkene (*E*)-**6a** was commercially obtained from Sigma-Aldrich. In an argon-filled glovebox, an oven-dried 10.0-mL Schlenk tube equipped with a magnetic stir bar was charged with (COD)Pd(CH_2SiMe_3)$_2$ (3.2 mg, 0.008 mmol, 2.0 mol%) and *o*-Tol$_3$P (3.6 mg, 0.012 mmol, 3.0 mol%), followed by the addition of anhydrous dichloromethane (2.0 mL, 0.2 M). After stirring for 5 min, internal alkene (*E*)-**6a** (47.3 mg, 0.40 mmol, 1.0 equiv) and allylsilane **5a** (90.2 mg, 0.60 mmol, 1.5 equiv) were added sequentially. The reaction mixture was stirred at room temperature for 2 h. Upon completion, the crude mixture was filtered through a short pad of silica gel, and all volatiles were removed under reduced pressure. The obtained residue was directly subjected to ^{1}H NMR spectroscopy using dibromomethane as an internal standard. Spectroscopic analysis verified that, except from partial isomerization of allylsilane **5a** (28% NMR yield), no reaction had occurred and that the starting material (*E*)-**6a** was fully recovered.

6.1.3 Isomerization of Allylsilane **5a** under the Standard Reaction Conditions

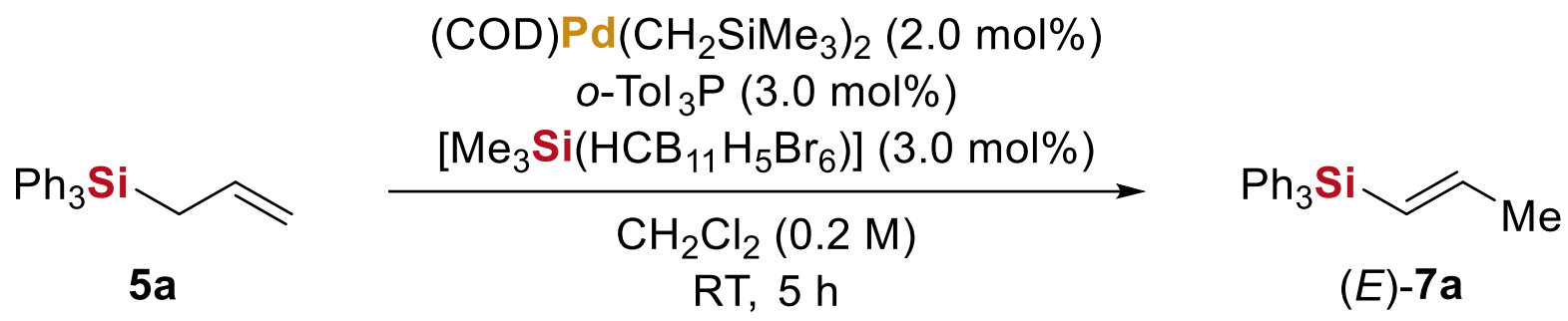

In an argon-filled glovebox, $(COD)Pd(CH_2SiMe_3)_2$ (3.2 mg, 0.008 mmol, 2.0 mol%) and *o*-Tol_3P (3.6 mg, 0.012 mmol, 3.0 mol%) were dissolved in degassed anhydrous CH_2Cl_2 (2.0 mL, 0.2 M). After stirring at room temperature for 1 h, $[Me_3Si(HCB_{11}H_5Br_6)]$ (8.4 mg, 0.012 mmol, 3.0 mol%) was added, followed by the addition of allylsilane **5a** (120.2 mg, 0.40 mmol, 1.0 equiv). The resulting reaction mixture was stirred for additional 5 h at room temperature. Upon completion, the mixture was removed from the glovebox and filtered through a short pad of silica gel. The filtrate was concentrated under reduced pressure, and the residue was purified by flash column chromatography on silica gel using *n*-pentane/CH_2Cl_2 = 10:1 as the eluent to afford the isomerized allylsilane (*E*)-**7a** (78.1 mg, 65% yield).

6.1.4 Reactivity of Internal Allylsilane (*E*)-**7a** under the Standard Reaction Conditions

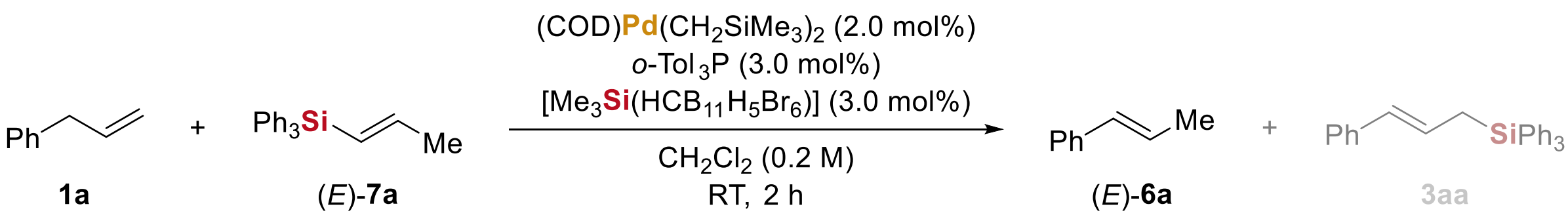

In an argon-filled glovebox, $(COD)Pd(CH_2SiMe_3)_2$ (3.2 mg, 0.008 mmol, 2.0 mol%) and *o*-Tol_3P (3.6 mg, 0.012 mmol, 3.0 mol%) were dissolved in degassed anhydrous CH_2Cl_2 (2.0 mL, 0.2 M). After stirring at room temperature for 1 h, $[Me_3Si(HCB_{11}H_5Br_6)]$ (8.4 mg, 0.012 mmol, 3.0 mol%) was added, followed by the addition of allylbenzene (**1a**, 47.3 mg, 0.40 mmol, 1.0 equiv) and vinylsilane (*E*)-**7a** (180.3 mg, 0.60 mmol, 1.5 equiv). The resulting reaction mixture was stirred for additional 5 h at room temperature. Upon completion, the mixture was removed from the glovebox and filtered through a short pad of silica gel. All volatiles were removed under reduced pressure, and the obtained residue was directly subjected to ^1H NMR spectroscopy using dibromomethane as an internal standard. Spectroscopic analysis verified that, except for partial isomerization of **1a** to (*E*)-**6a** (36% NMR yield), no reaction had occurred and that the starting material (*E*)-**7a** was fully recovered.

6.2 Equilibrium Studies on (o-$Tol_3P)_2Pd$ and Free Phosphine

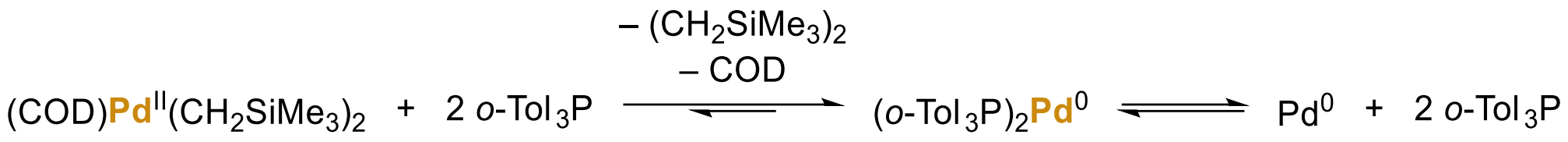

When $(COD)Pd(CH_2SiMe_3)_2$ was present in excess relative to o-Tol_3P, the $^{31}P\{^1H\}$ NMR spectrum showed a single resonance at δ = –6.84 ppm, which is assigned to (o-$Tol_3P)_2Pd$ (Figure S1A). No signal corresponding to free o-Tol_3P (δ = –30.21 ppm) was detected, indicating quantitative formation of the bis(phosphine) palladium(0) complex under these conditions. In contrast, when $(COD)Pd(CH_2SiMe_3)_2$ and o-Tol_3P were combined in a 1:2 molar ratio, the $^{31}P\{^1H\}$ NMR spectrum showed two distinct resonances, one at δ = –6.84 ppm for (o-$Tol_3P)_2Pd$ and an additional signal at δ = –30.21 ppm for o-Tol_3P (Figure S1B). The presence of uncoordinated phosphine indicates that under stoichiometric ligand conditions, (o-$Tol_3P)_2Pd$ exists in equilibrium with free o-Tol_3P in solution. These observations show that the composition of the (o-$Tol_3P)_2Pd$ is dependent on the relative stoichiometry of the palladium precursor and phosphine. Only the use of excess amounts of $(COD)Pd(CH_2SiMe_3)_2$ allows the exclusive formation of (o-$Tol_3P)_2Pd$ without the presence of free phosphine.

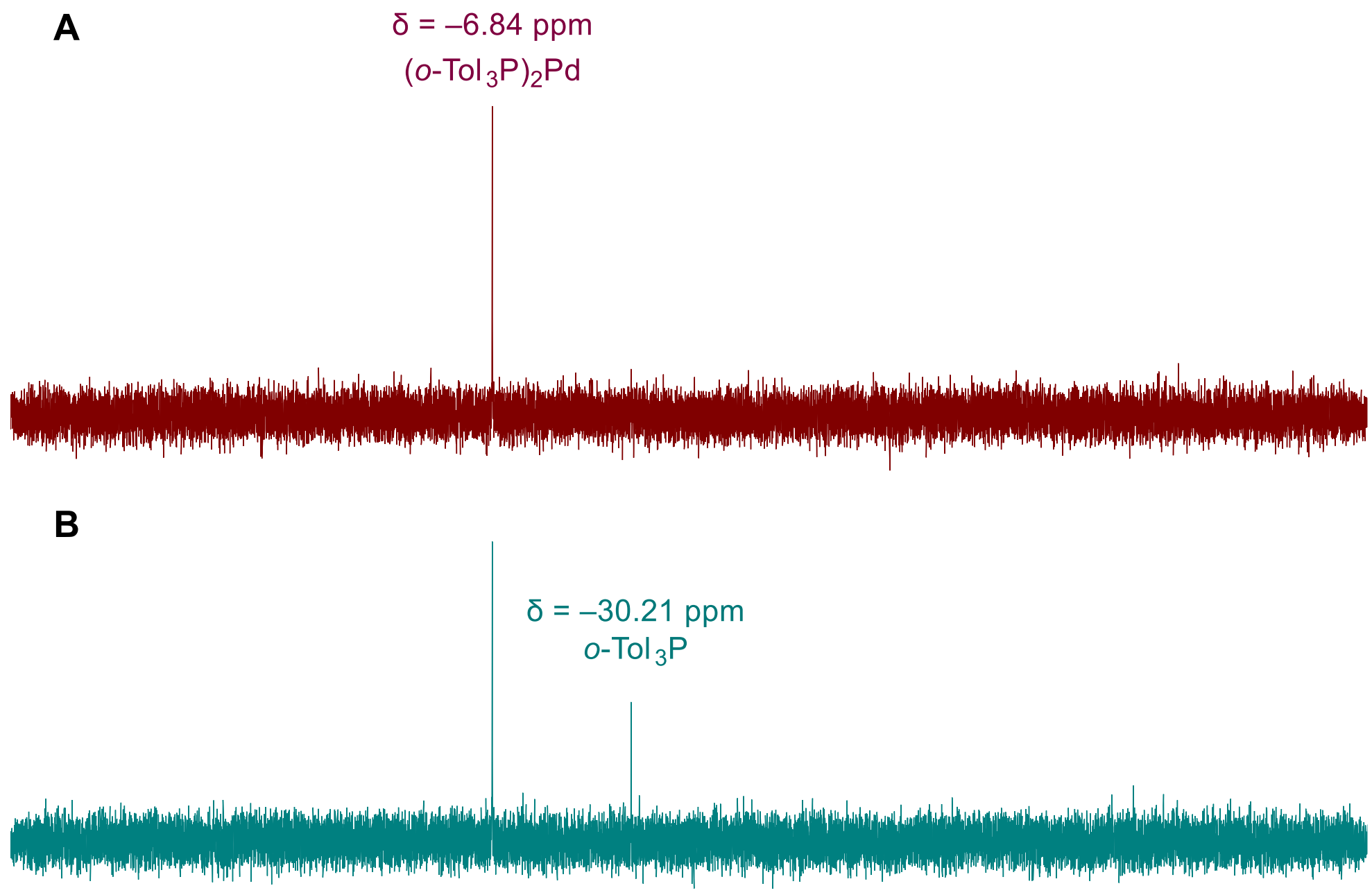

Figure S1. $^{31}P\{^1H\}$ NMR spectra (202 MHz, CD_2Cl_2) of the equilibrium between (o-$Tol_3P)_2Pd$ and free phosphine. (**A**) $(COD)Pd(CH_2SiMe_3)_2$ (19.5 mg, 0.05 mmol, 1.0 equiv) and o-Tol_3P (22.8 mg, 0.75 mmol, 1.5 equiv) were used. (**B**) $(COD)Pd(CH_2SiMe_3)_2$ (19.5 mg, 0.05 mmol, 1.0 equiv) and o-Tol_3P (30.4 mg, 0.10 mmol, 2.0 equiv) were used.

6.3 Catalytic Activity of [(*o*-Tol$_3$P)$_2$PdSiMe$_3$][HCB$_{11}$H$_5$Br$_6$] (9b)

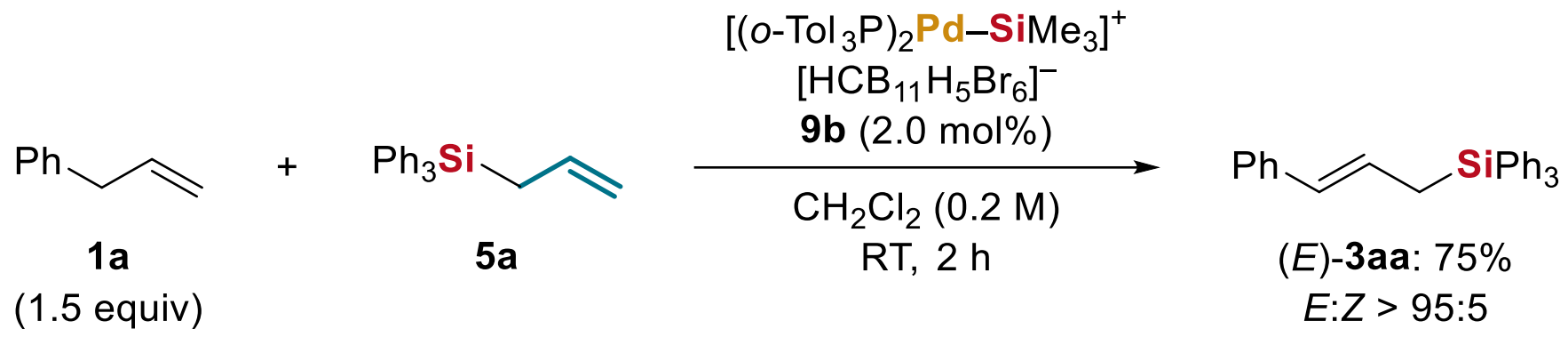

In an argon-filled glovebox, [(*o*-Tol$_3$P)$_2$PdSiMe$_3$][HCB$_{11}$H$_5$Br$_6$] (**9b**, 5.6 mg, 2.0 mol%) was dissolved in degassed anhydrous CH_2Cl_2 (0.5 mL), followed by the addition of allylbenzene (**1a**, 39.7 µL, 0.30 mmol, 1.5 equiv) and allyltriphenylsilane (**5a**, 60.1 mg, 0.20 mmol, 1.0 equiv). The resulting reaction mixture was stirred for 2 h at room temperature. Upon completion, the reaction mixture was removed from the glovebox and filtered through a short pad of silica gel. The filtrate was concentrated under vacuum, and the residue was purified by flash column chromatography on silica gel, affording the desired allylsilane **3aa** in 75% yield.

6.4 Catalytic Activity of [(o-Tol$_3$P)$_2$Pd(H)(MeCN)][HCB$_{11}$H$_5$Br$_6$] (11·MeCN)

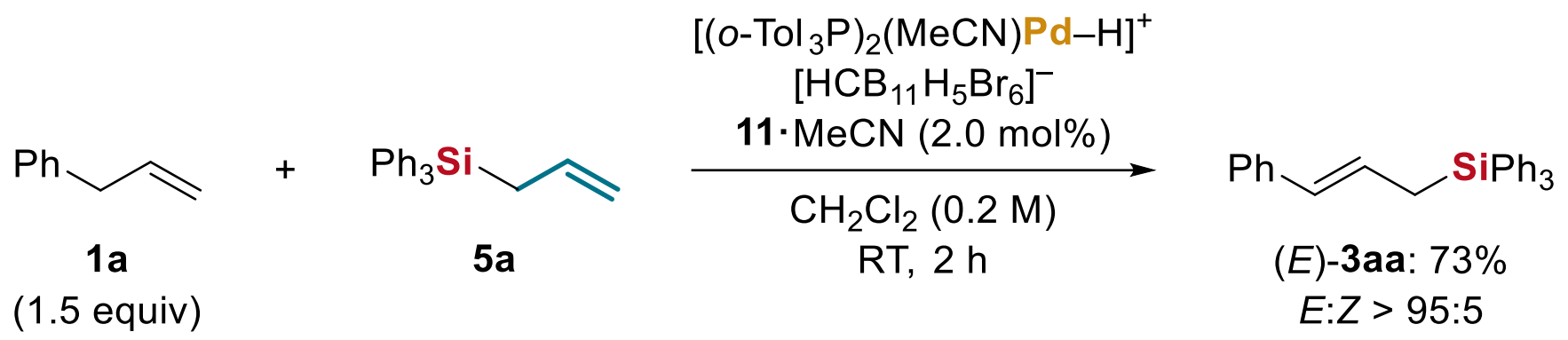

In an argon-filled glovebox, [(o-Tol$_3$P)$_2$Pd(H)(MeCN)][HCB$_{11}$H$_5$Br$_6$] (**11**·MeCN, 5.5 mg, 2.0 mol%) was dissolved in degassed anhydrous CH_2Cl_2 (0.5 mL), followed by the addition of allylbenzene (**1a**, 39.7 µL, 0.30 mmol, 1.5 equiv) and allyltriphenylsilane (**5a**, 60.1 mg, 0.2 mmol, 1.0 equiv). The resulting reaction mixture was stirred for 2 h at room temperature. Upon completion, the reaction mixture was removed from the glovebox and filtered through a short pad of silica gel. The filtrate was concentrated under vacuum, and the residue was purified by flash column chromatography on silica gel, affording the desired allylsilane **3aa** in 73% yield.

6.5 Time-Dependent ^{1}H NMR Spectroscopy of the Reaction of Allyltrimethylsilane (5b) with [(*o*-Tol$_3$P)$_2$Pd(H)(MeCN)][HCB$_{11}$H$_5$Br$_6$] (11·MeCN)

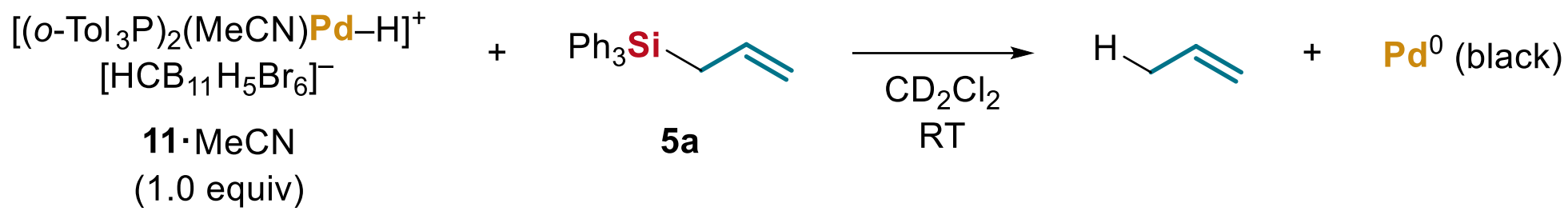

In an argon-filled glove box, [(*o*-Tol$_3$P)$_2$Pd(H)(MeCN)][HCB$_{11}$H$_5$Br$_6$] (**11**·MeCN, 20.9 mg, 0.03 mmol) was suspended in CD_2Cl_2 (0.5 mL), followed by the addition of allyltrimethylsilane (**5b**, 34.3 mg, 0.03 mmol). The mixture was transferred into a *J*-Young NMR tube and directly subjected to ^{1}H NMR spectroscopy. The time-dependent spectroscopic monitoring revealed nearly complete consumption of the allylsilane **5b** and the Pd–H$^+$ complex **11**·MeCN after 12 h (without stirring) and the formation of propene. While the propene signal gradually increased, a slightly increasing amount of the isomerization product (**5b** → (*E*)-**7b**) was also observed. Overall, this NMR spectroscopic analysis indicated proton transfer from the Pd–H$^+$ complex **11** to the allylsilane **1b** with formation of propene.

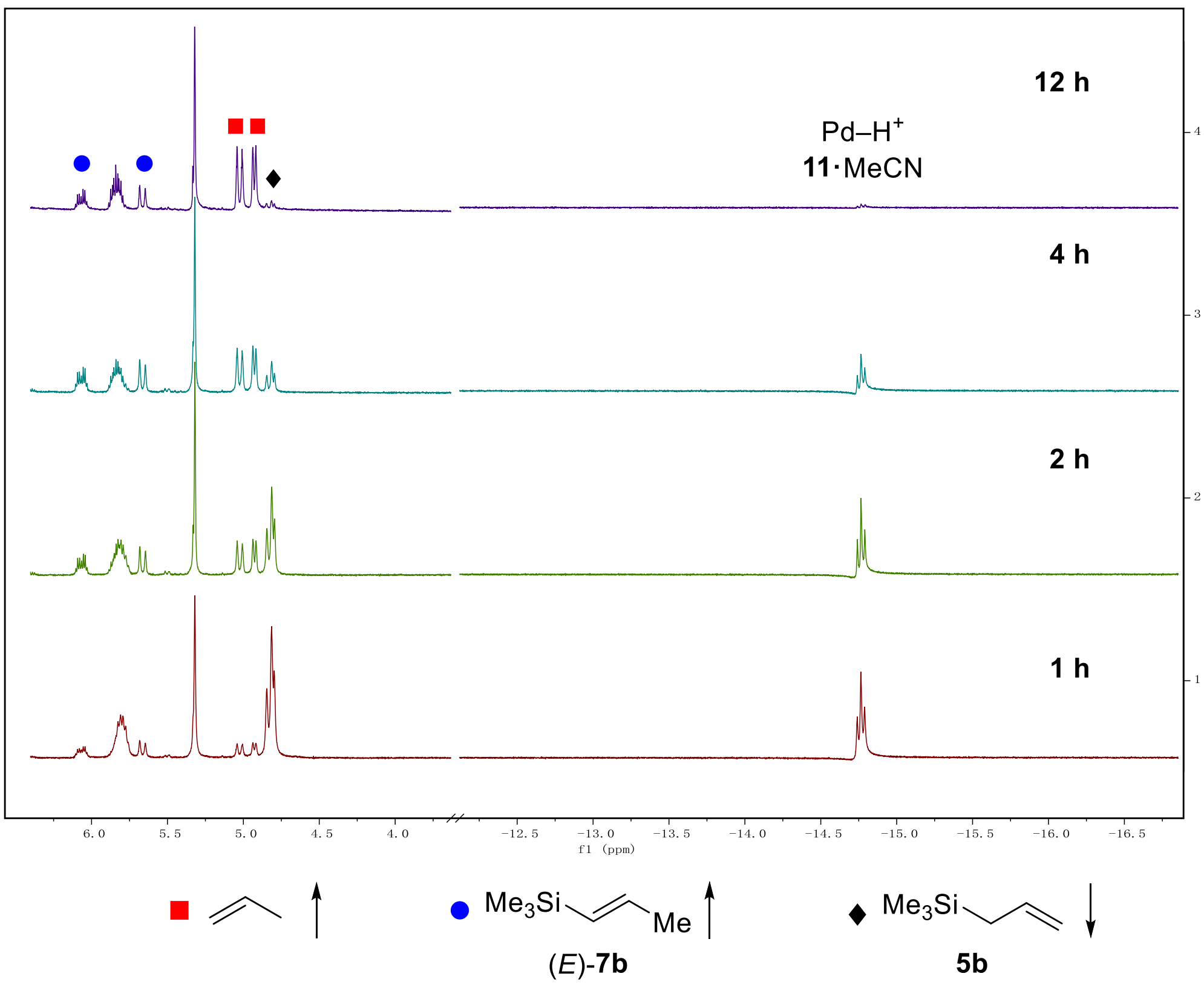

Figure S2. Time-dependent ^{1}H NMR spectroscopic analysis (CD_2Cl_2, 500 MHz, 298 K) of the reaction between [(*o*-Tol$_3$P)$_2$Pd(H)(MeCN)][HCB$_{11}$H$_5$Br$_6$] (**11**·MeCN) and allyltrimethylsilane (**5b**).

7 Crystallographic Data

7.1 Molecular Structure of [*o*-Tol_3PSiMe_3][$HCB_{11}H_5Br_6$] (10b) [CCDC 2538954]

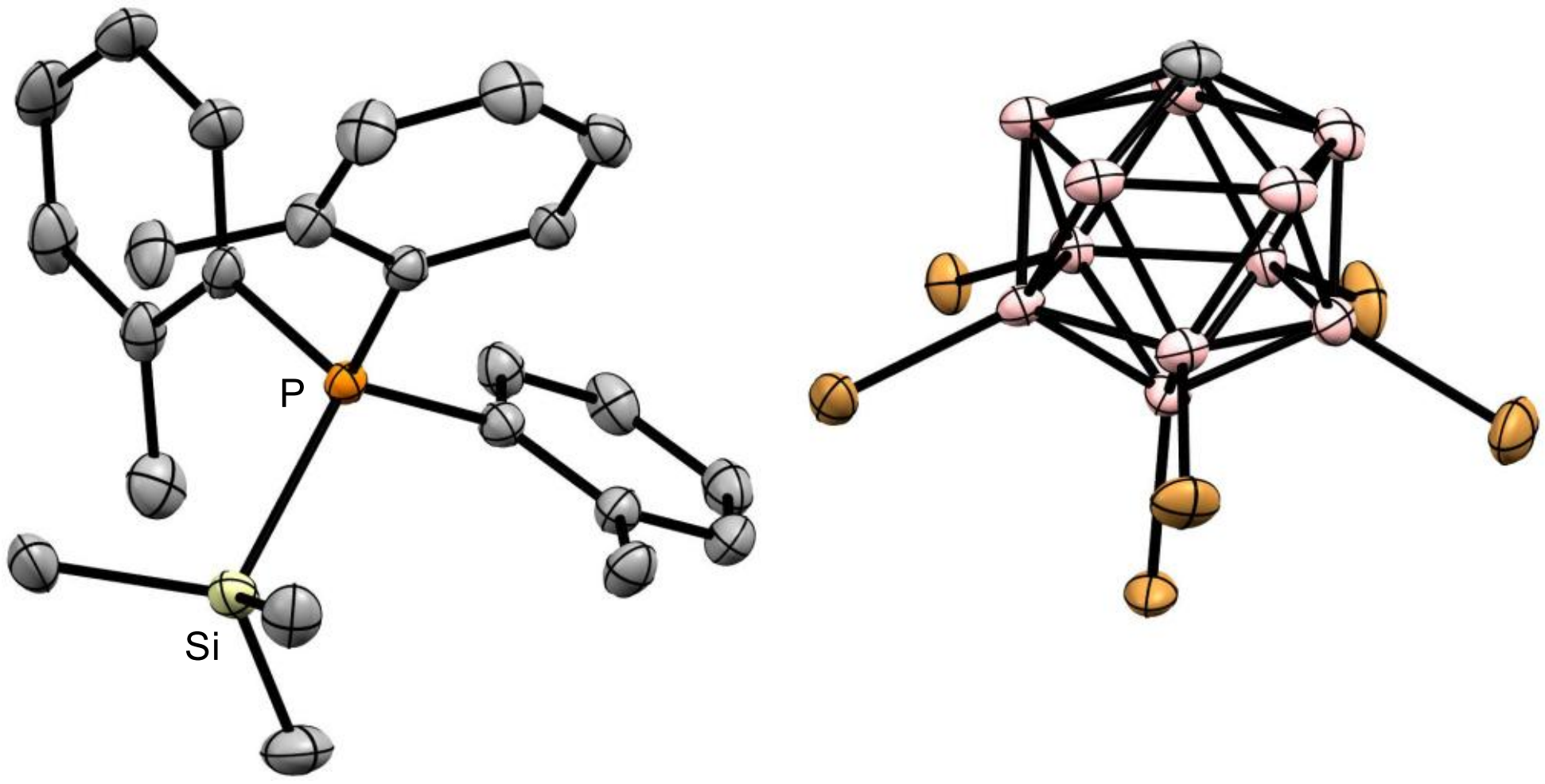

Figure S3. ORTEP view of the molecular structure of [*o*-Tol_3PSiMe_3][$HCB_{11}H_5Br_6$] (**10b**) with thermal ellipsoids shown at the 50% probability level. Hydrogen atoms are omitted for clarity.

Table S1. Crystal data and structure refinement for [*o*-Tol_3PSiMe_3][$HCB_{11}H_5Br_6$].

Empirical formula	$C_{25}H_{36}B_{11}Br_6PSi$	
Formula weight	993.97	
Temperature	150.01(10) K	
Wavelength	1.54184 Å	
Crystal system	Orthorhombic	
Space group	*Pbcn* (No. 60)	
Unit cell dimensions	a = 20.1781(3) Å	α = 90°
	b = 13.2442(2) Å	β = 90°
	c = 27.9981(3) Å	γ = 90°
Volume	7482.29(18) $Å^3$	
Z	8	
Density (calculated)	1.765 Mg/m^3	
Absorption coefficient	8.594 mm^{-1}	
F(000)	3840	
Crystal size	0.246 x 0.124 x 0.052 mm^3	
Theta range for data collection	3.157 to 72.690°	
Index ranges	-24<=h<=19, -16<=k<=12, -34<=l<=33	

Reflections collected	29929
Independent reflections	7327 [R(int) = 0.0279]
Completeness to theta = 67.684°	100.0%
Absorption correction	Semi-empirical from equivalents
Max. and min. transmission	1.00000 and 0.11500
Refinement method	Full-matrix least-squares on F^2
Data / restraints / parameters	7327 / 0 / 403
Goodness-of-fit on F^2	1.034
Final R indices [I>2sigma(I)]	R1 = 0.0239, wR2 = 0.0578
R indices (all data)	R1 = 0.0273, wR2 = 0.0598
Extinction coefficient	n/a
Largest diff. peak and hole	0.968 and -0.952 e.Å^{-3}

7.2 Molecular Structure of [(*o*-$Tol_3P)_2Pd(H)(MeCN)][HCB_{11}H_5Br_6]$ (11·MeCN)

[CCDC 2538955]

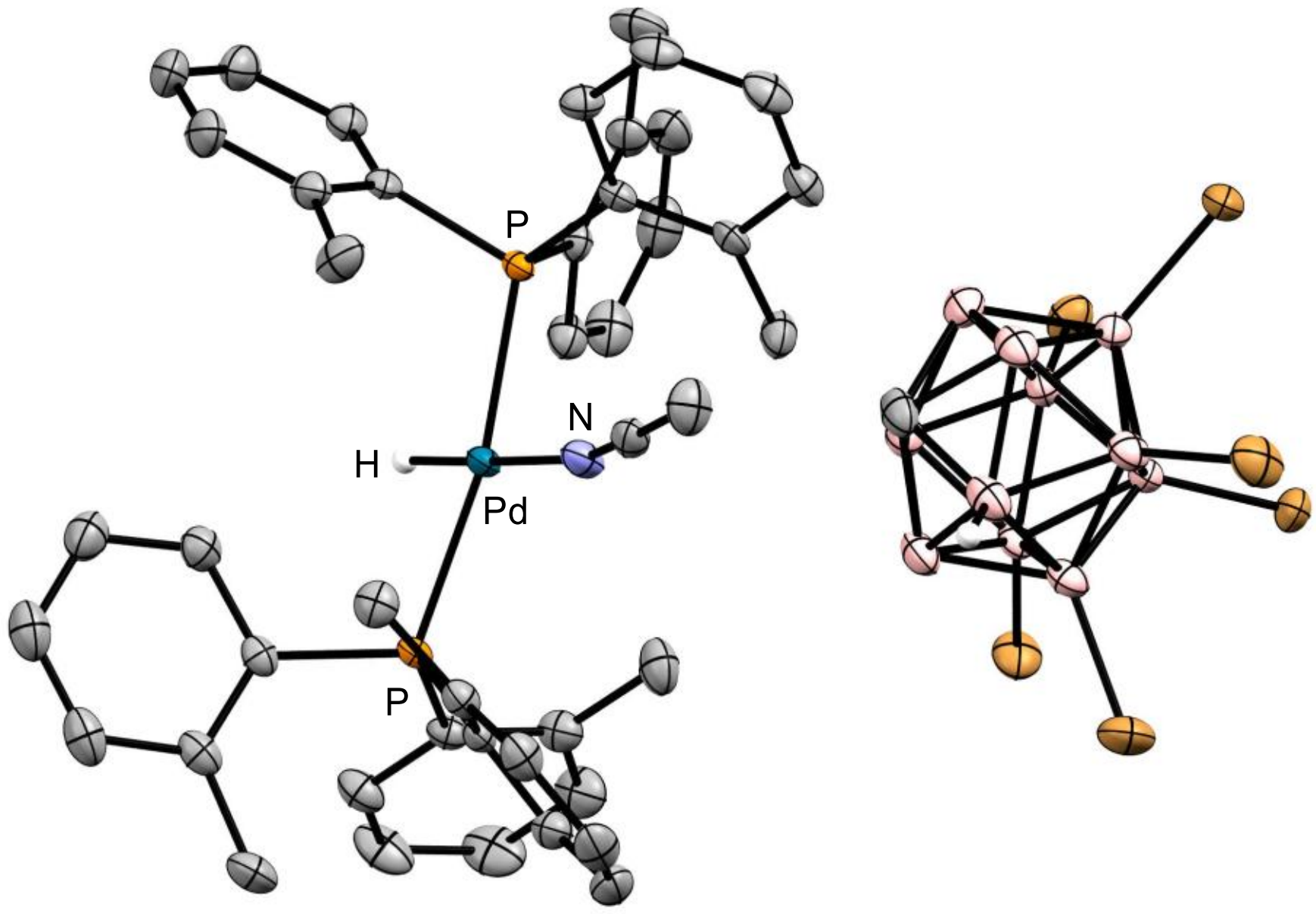

Figure S4. ORTEP view of the molecular structure of *trans*-[(*o*-$Tol_3P)_2Pd(H)(MeCN)][HCB_{11}H_5Br_6]$ (**11**·MeCN) with thermal ellipsoids shown at the 50% probability level. Hydrogen atoms except for Pd–H are omitted for clarity.

Table S2. Crystal data and structure refinement for *trans*-[(o-$Tol_3P)_2Pd(H)(MeCN)][HCB_{11}H_5Br_6]$.

Empirical formula	$C_{45}H_{52}B_{11}Br_6NP_2Pd$	
Formula weight	1373.58	
Temperature	150.01(10) K	
Wavelength	1.54184 Å	
Crystal system	Triclinic	
Space group	*P*-1 (No. 2)	
Unit cell dimensions	a = 12.5564(3) Å	α = 87.018(2)°
	b = 14.5458(4) Å	β = 84.814(2)°
	c = 14.9270(4) Å	γ = 80.548(2)°
Volume	2676.37(12) $Å^3$	
Z	2	
Density (calculated)	1.704 Mg/m^3	
Absorption coefficient	8.853 mm^{-1}	
F(000)	1340	

Crystal size	0.392 x 0.170 x 0.080 mm^3
Theta range for data collection	2.975 to 72.527°
Index ranges	-14<=h<=15, -17<=k<=17, -18<=l<=14
Reflections collected	19924
Independent reflections	10343 [R(int) = 0.0268]
Completeness to theta = 67.684°	99.9%
Absorption correction	Semi-empirical from equivalents
Max. and min. transmission	1.00000 and 0.35117
Refinement method	Full-matrix least-squares on F^2
Data / restraints / parameters	10343 / 0 / 606
Goodness-of-fit on F^2	1.049
Final R indices [I>2sigma(I)]	R1 = 0.0287, wR2 = 0.0725
R indices (all data)	R1 = 0.0310, wR2 = 0.0743
Extinction coefficient	n/a
Largest diff. peak and hole	0.696 and -0.998 e.$Å^{-3}$

7.3 Molecular Structure of [*o*-Tol$_3$PH][HCB$_{11}$H$_5$Br$_6$] (12) [CCDC 2538956]

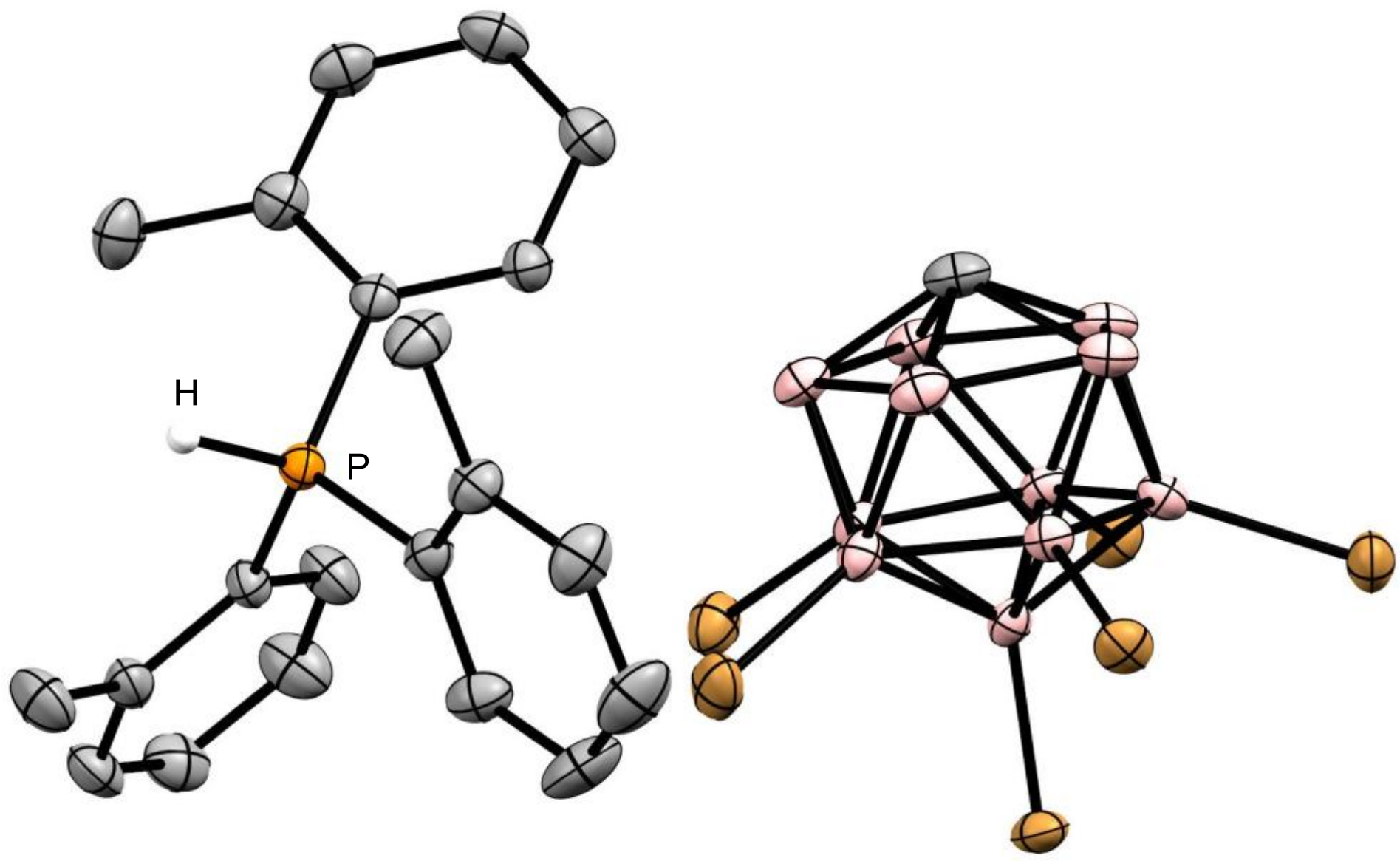

Figure S5. ORTEP view of the molecular structure of [*o*-Tol$_3$PH][HCB$_{11}$H$_5$Br$_6$] (**12**) with thermal ellipsoids shown at the 50% probability level. Hydrogen atoms except for P–H are omitted for clarity.

Table S3. Crystal data and structure refinement for [*o*-Tol$_3$PH][HCB$_{11}$H$_5$Br$_6$].

Empirical formula	$C_{22}H_{28}B_{11}Br_6P$	
Formula weight	921.78	
Temperature	150.01(10) K	
Wavelength	1.54184 Å	
Crystal system	Orthorhombic	
Space group	Pna2$_1$ (No. 33)	
Unit cell dimensions	a = 14.15550(10) Å	α = 90°
	b = 25.2612(3) Å	β = 90°
	c = 9.37890(10) Å	γ = 90°
Volume	3353.75(6) Å^3	
Z	4	
Density (calculated)	1.826 Mg/m^3	
Absorption coefficient	9.200 mm^{-1}	
F(000)	1760	
Crystal size	0.190 x 0.106 x 0.036 mm^3	
Theta range for data collection	3.499 to 72.651°	

Index ranges	-17<=h<=12, -28<=k<=31, -11<=l<=11
Reflections collected	23950
Independent reflections	6429 [R(int) = 0.0280]
Completeness to theta = 67.684°	100.0%
Absorption correction	Semi-empirical from equivalents
Max. and min. transmission	1.00000 and 0.21689
Refinement method	Full-matrix least-squares on F^2
Data / restraints / parameters	6429 / 1 / 368
Goodness-of-fit on F2	1.043
Final R indices [I>2sigma(I)]	R1 = 0.0189, wR2 = 0.0480
R indices (all data)	R1 = 0.0195, wR2 = 0.0484
Absolute structure parameter	-0.024(13)
Extinction coefficient	n/a
Largest diff. peak and hole	0.275 and -0.366 e.Å^{-3}

8 NMR Spectra

Figure S6. ^{1}H NMR spectrum (500 MHz, $CDCl_3$, 298 K) of Allyltriphenylsilane (5a)

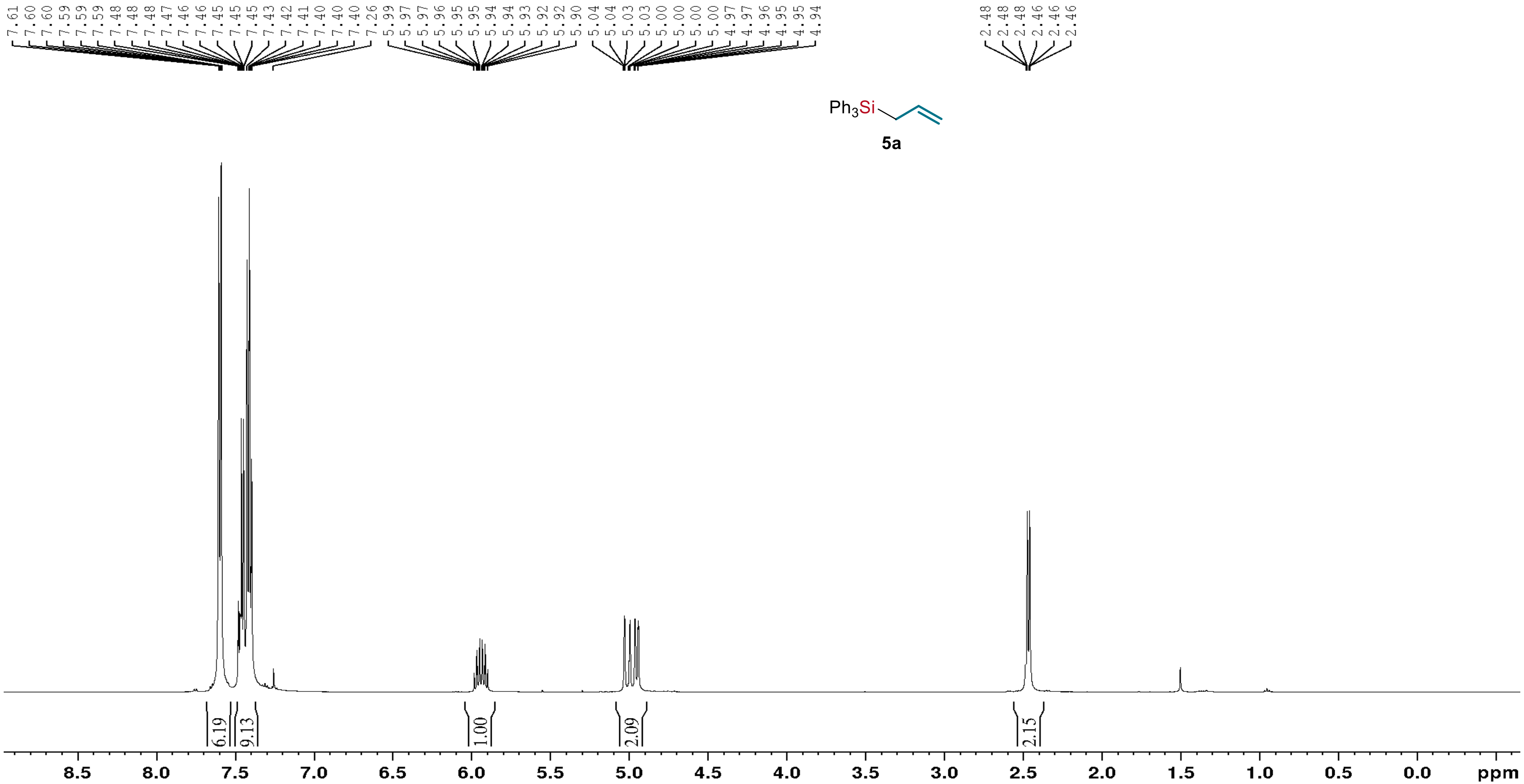

Figure S7. $^{13}C\{^1H\}$ **NMR spectrum (126 MHz,** $CDCl_3$**, 298 K) of Allyltriphenylsilane (5a)**

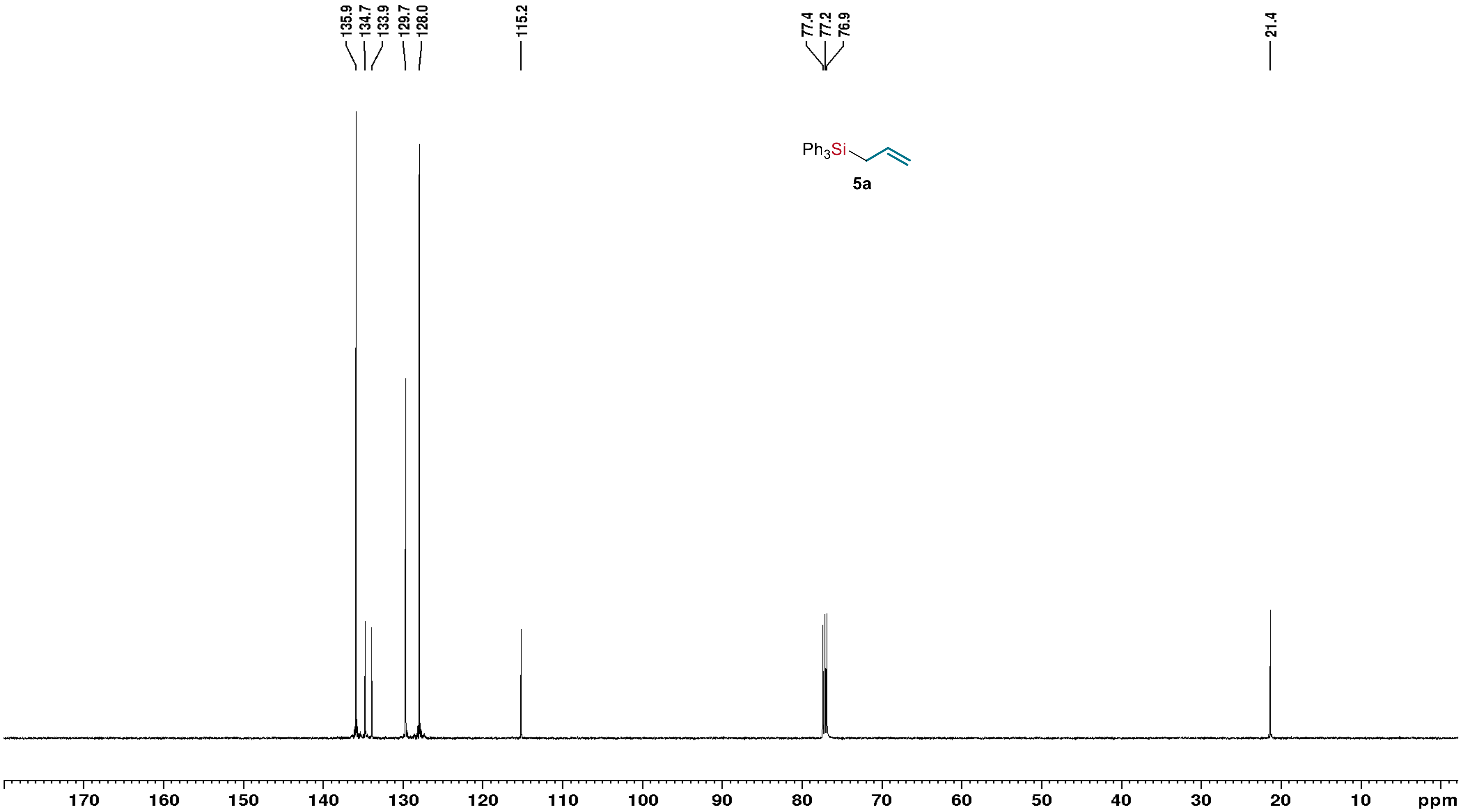

Figure S8. $^1H/^{29}Si$ HMQC NMR spectrum (500/99 MHz, $CDCl_3$, 298 K, optimized for J = 7 Hz) of 5a

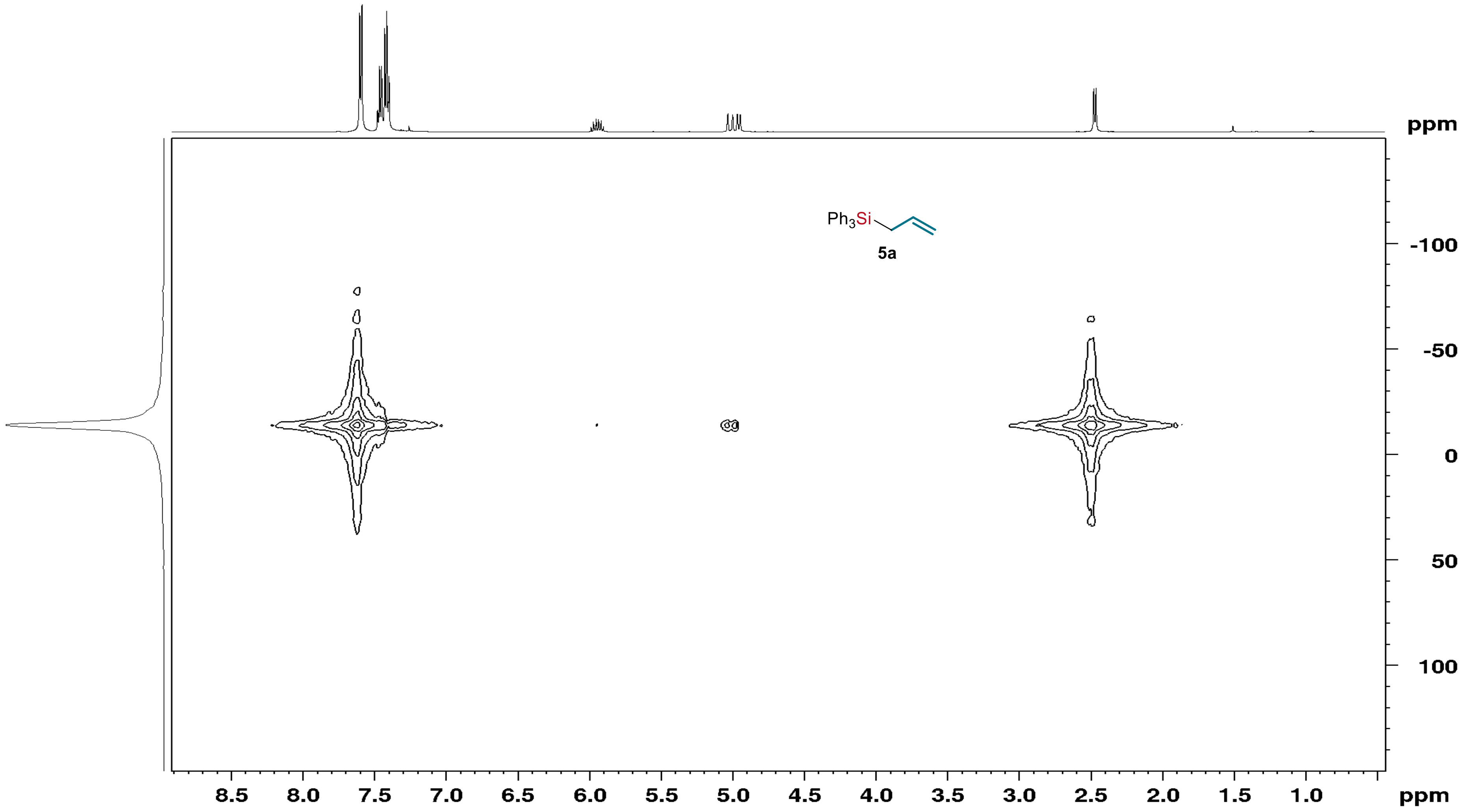

Figure S9. ^{1}H NMR spectrum (500 MHz, $CDCl_3$, 298 K) of Allyltri-*n*-butylsilane (5c)

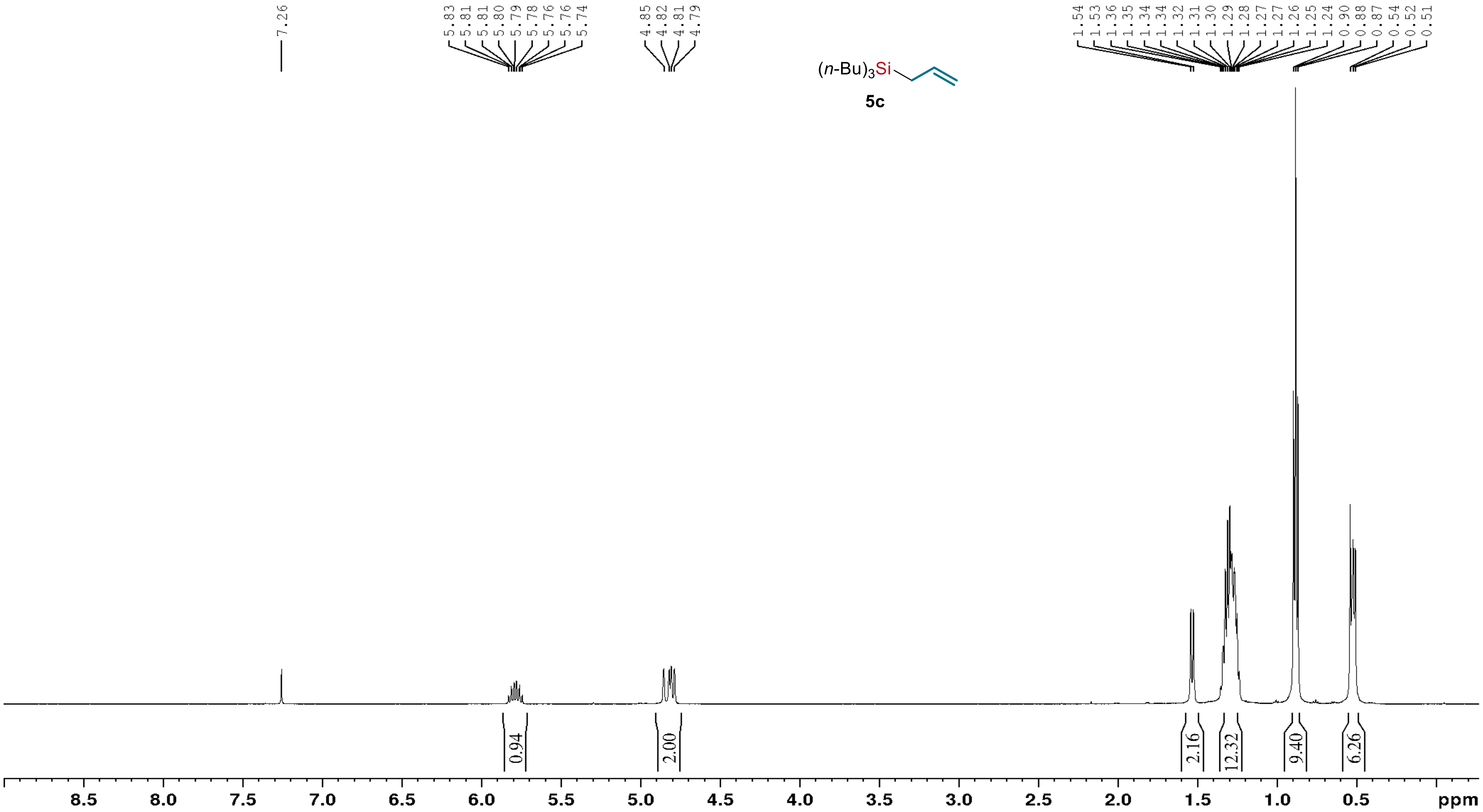

Figure S10. $^{13}C\{^{1}H\}$ **NMR spectrum (126 MHz, $CDCl_3$, 298 K) of Allyltri-*n*-butylsilane (5c)**

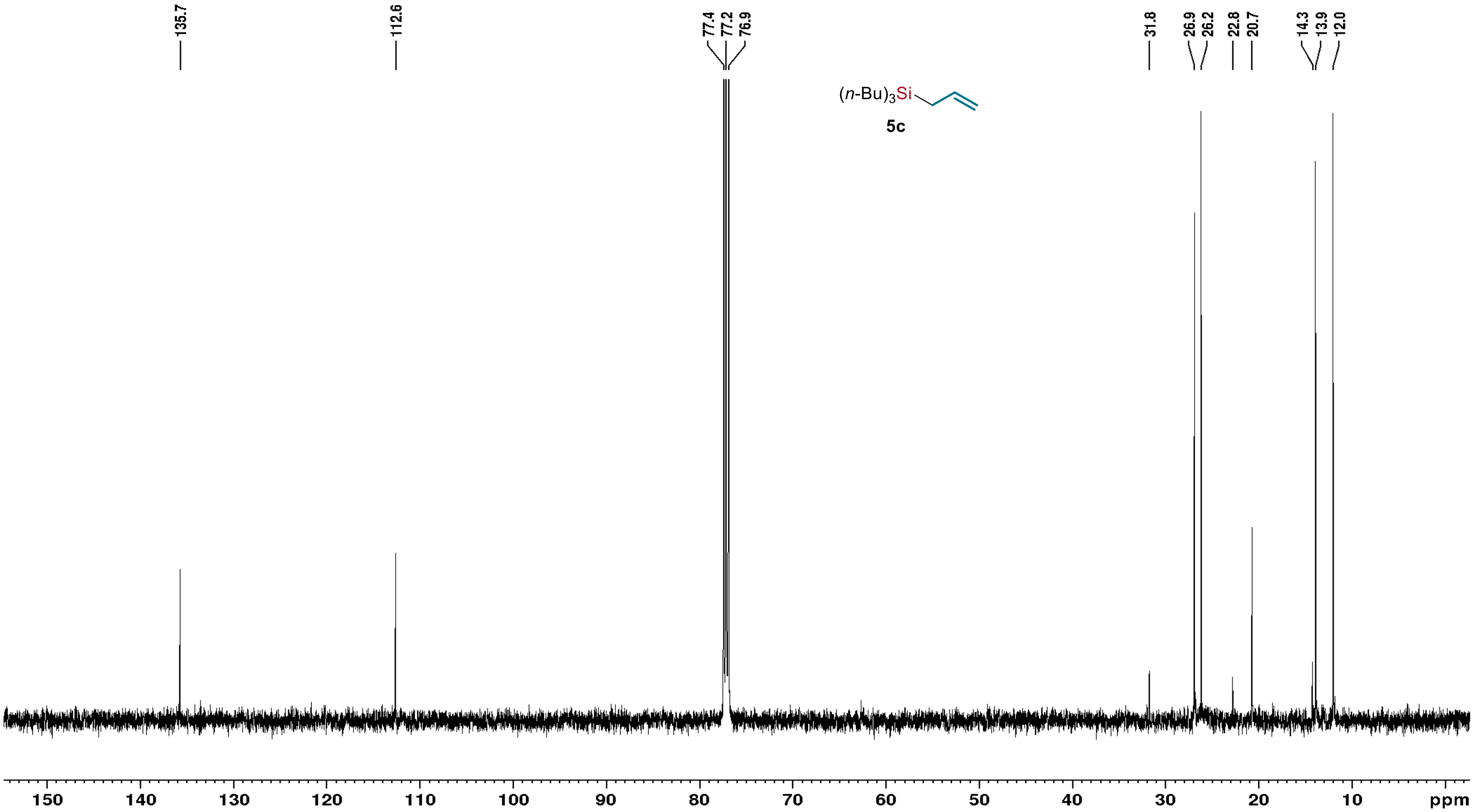

Figure S11. $^{1}H/^{29}Si$ **HMQC NMR spectrum (500/99 MHz, $CDCl_3$, 298 K, optimized for *J* = 7 Hz) of 5c**

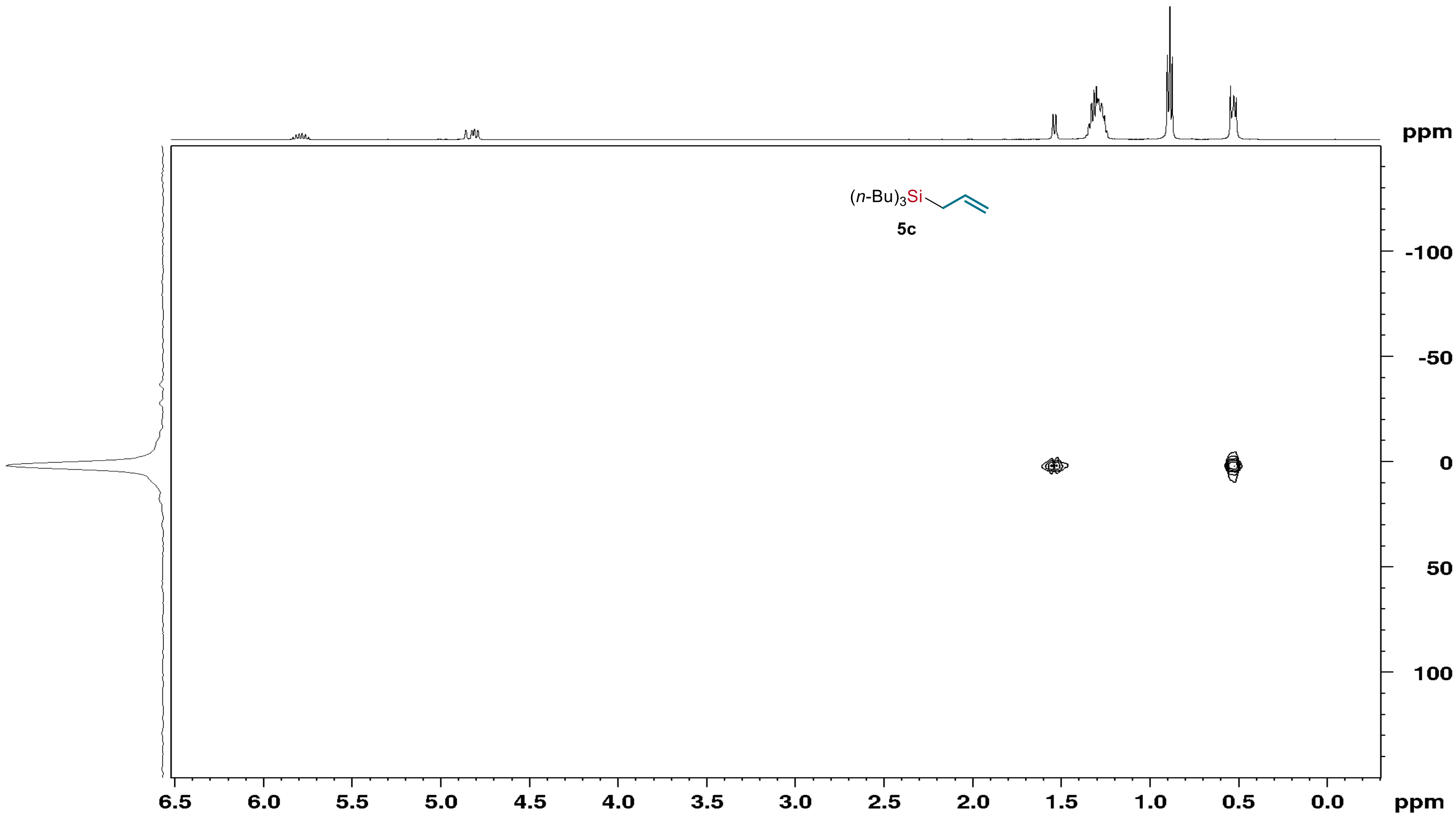

Figure S12. ^{1}H NMR spectrum (500 MHz, $CDCl_3$, 298 K) of Allyl(benzyl)dimethylsilane (5d)

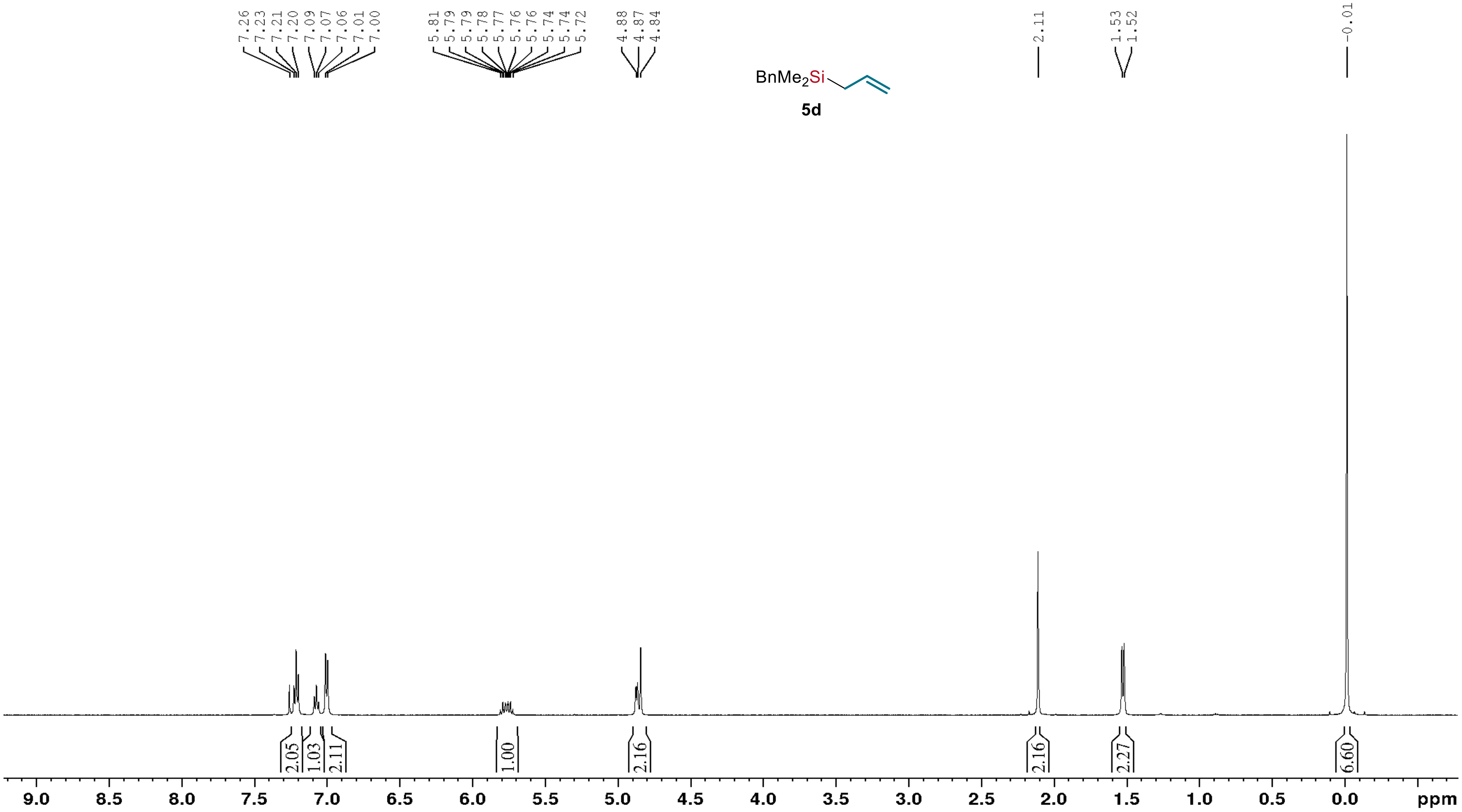

Figure S13. $^{13}C\{^1H\}$ NMR spectrum (126 MHz, $CDCl_3$, 298 K) of Allyl(benzyl)dimethylsilane (5d)

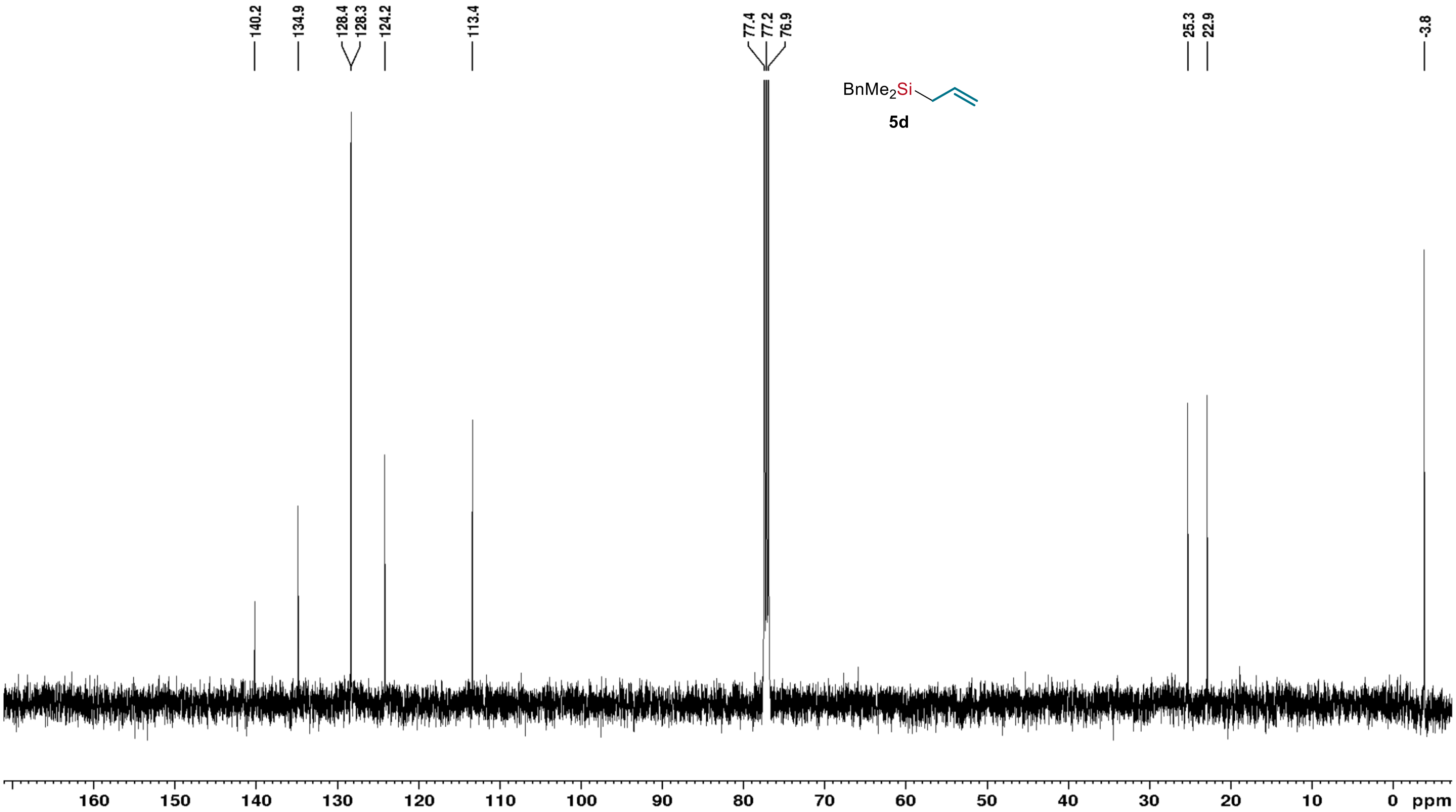

Figure S14. **$^1H/^{29}Si$ HMQC NMR spectrum (500/99 MHz, $CDCl_3$, 298 K, optimized for *J* = 7 Hz) of 5d**

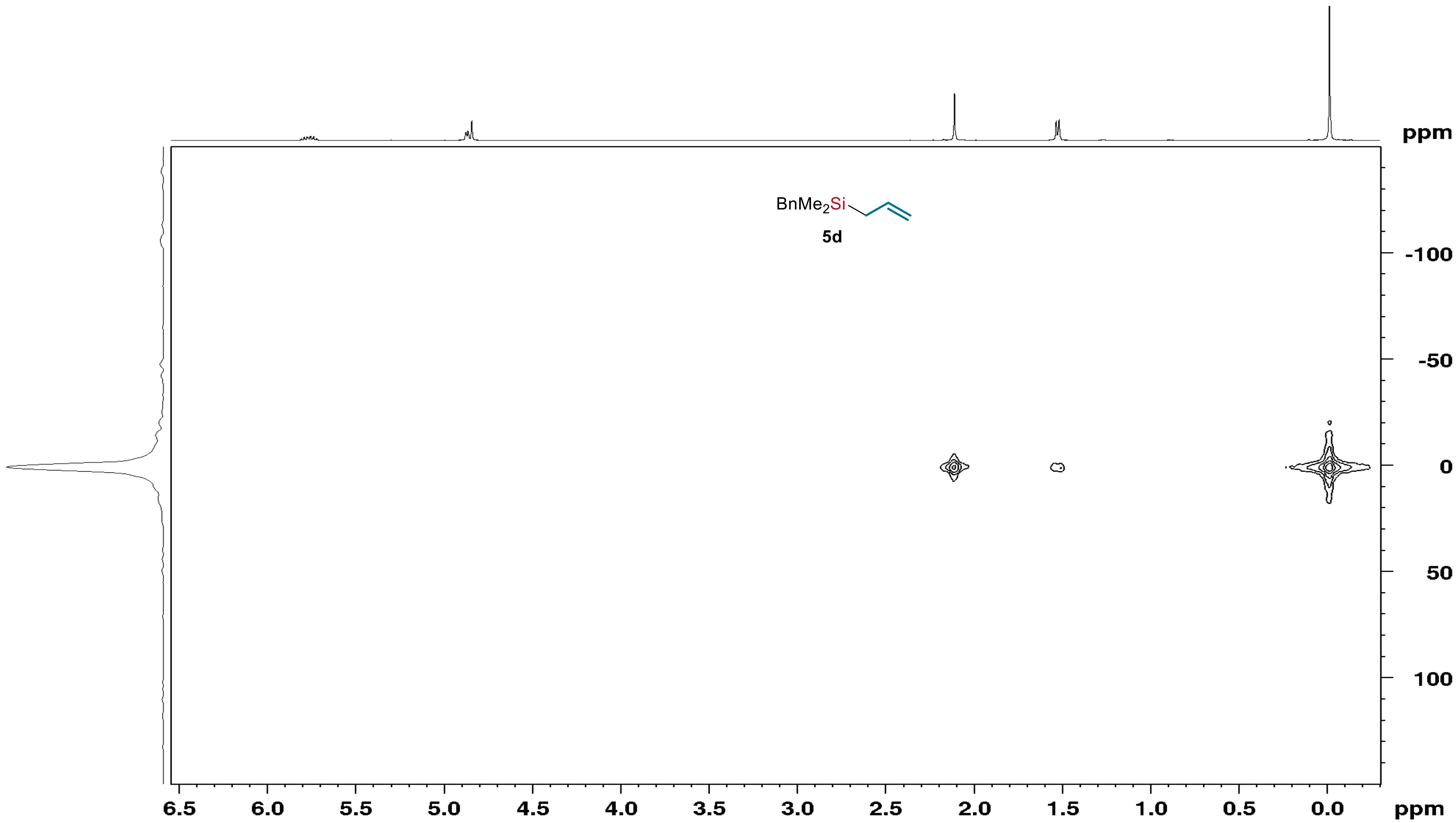

Figure S15. ^{1}H NMR spectrum (500 MHz, $CDCl_3$, 298 K) of Allyldimethyl(phenyl)silane (5e)

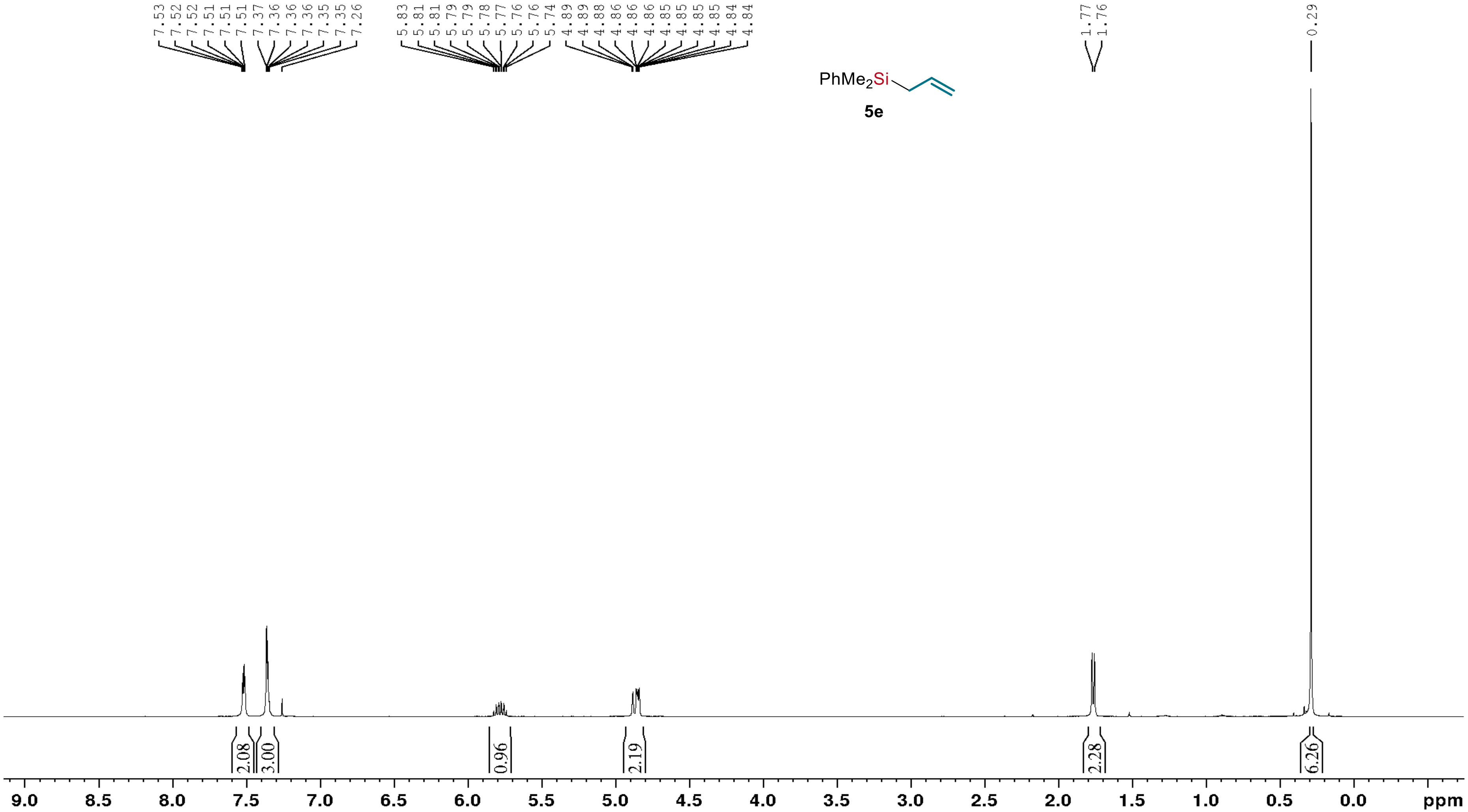

Figure S16. $^{13}C\{^{1}H\}$ NMR spectrum (126 MHz, $CDCl_3$, 298 K) of Allyldimethyl(phenyl)silane (5e)

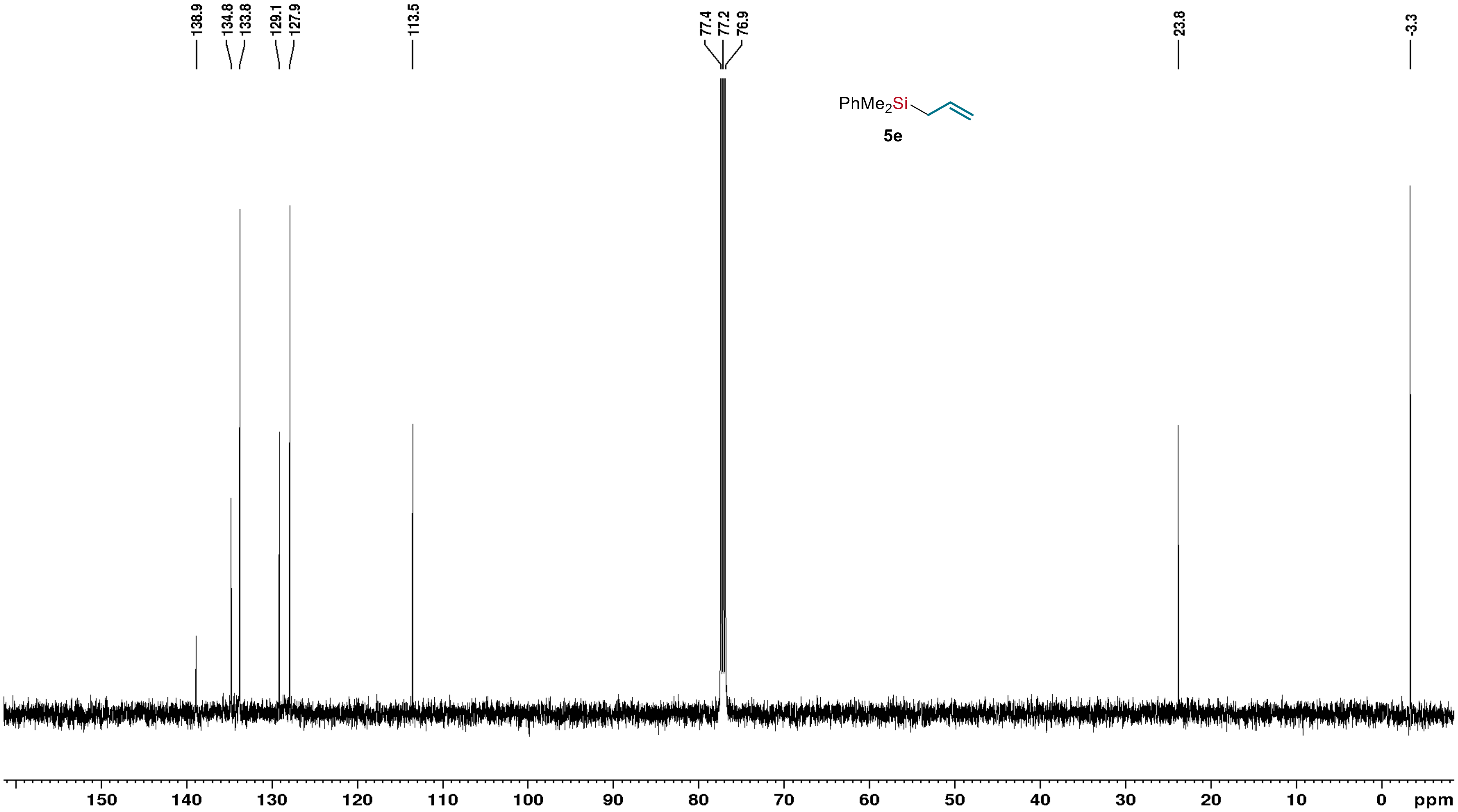

Figure S17. $^{1}H/^{29}Si$ **HMQC NMR spectrum (500/99 MHz, $CDCl_3$, 298 K, optimized for *J* = 7 Hz) of 5e**

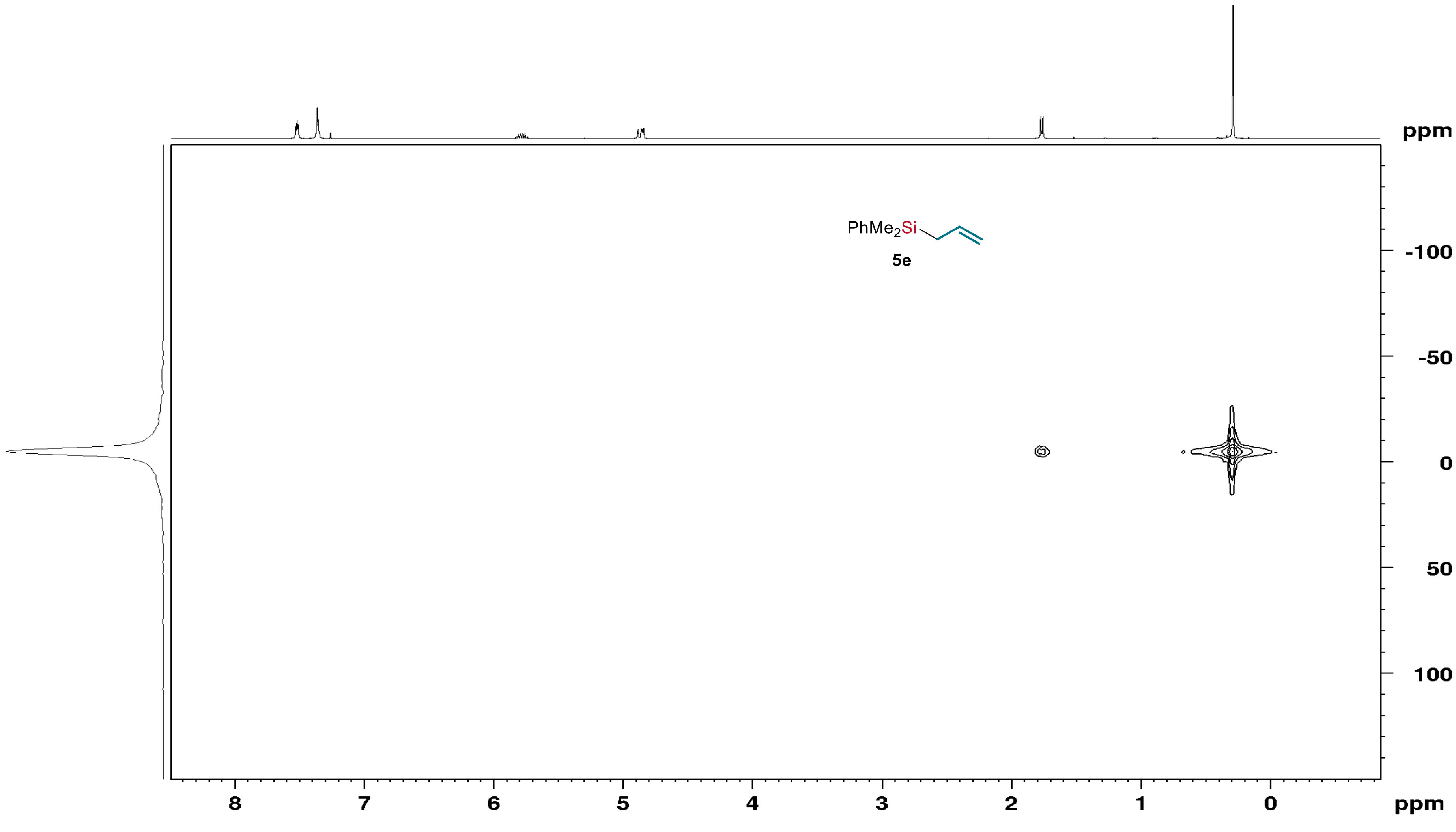

Figure S18. ^{1}H NMR spectrum (500 MHz, $CDCl_3$, 298 K) of Allyl(methyl)diphenylsilane (5f)

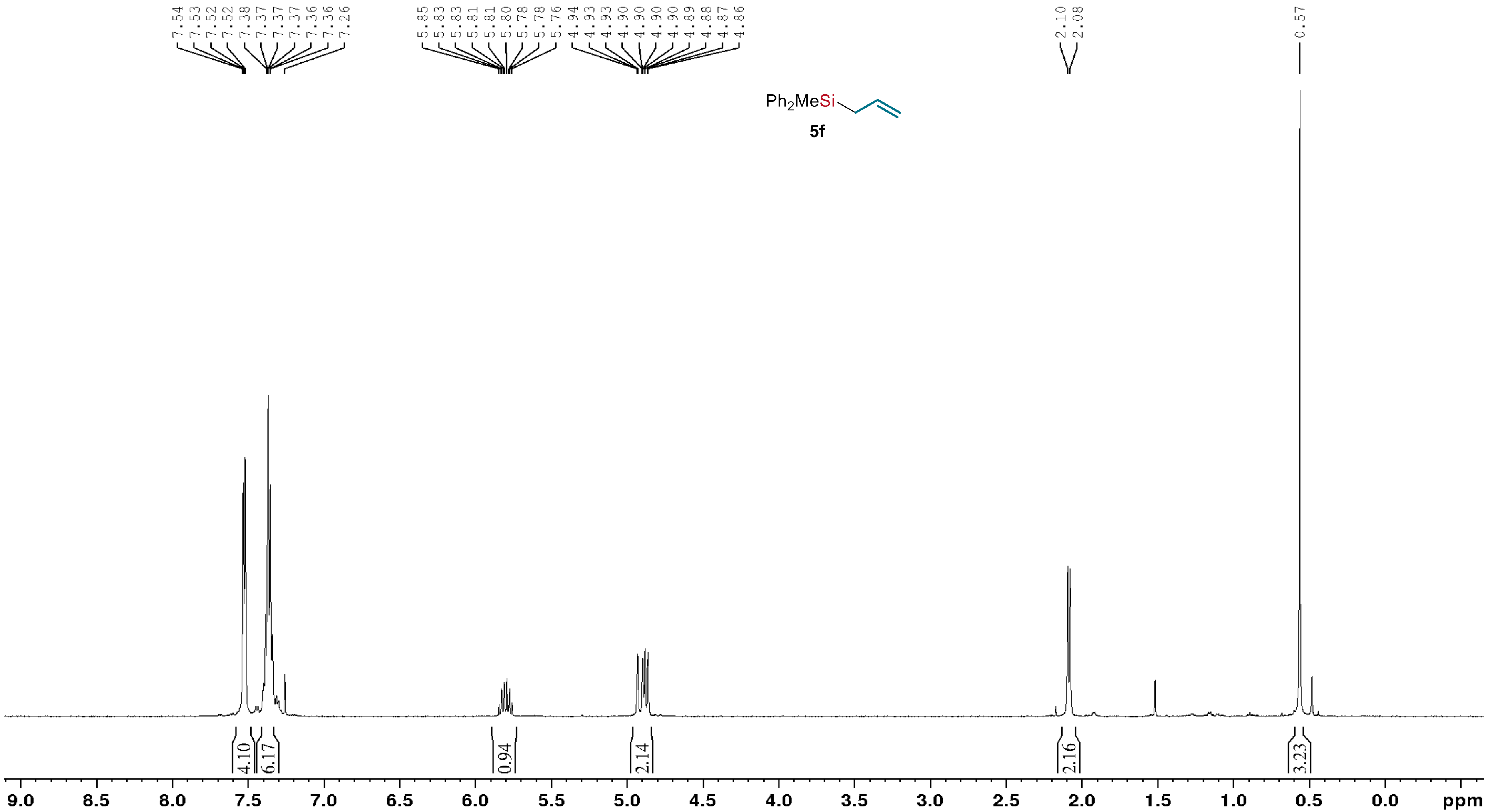

Figure S19. $^{13}C\{^1H\}$ **NMR spectrum (126 MHz, $CDCl_3$, 298 K) of Allyl(methyl)diphenylsilane (5f)**

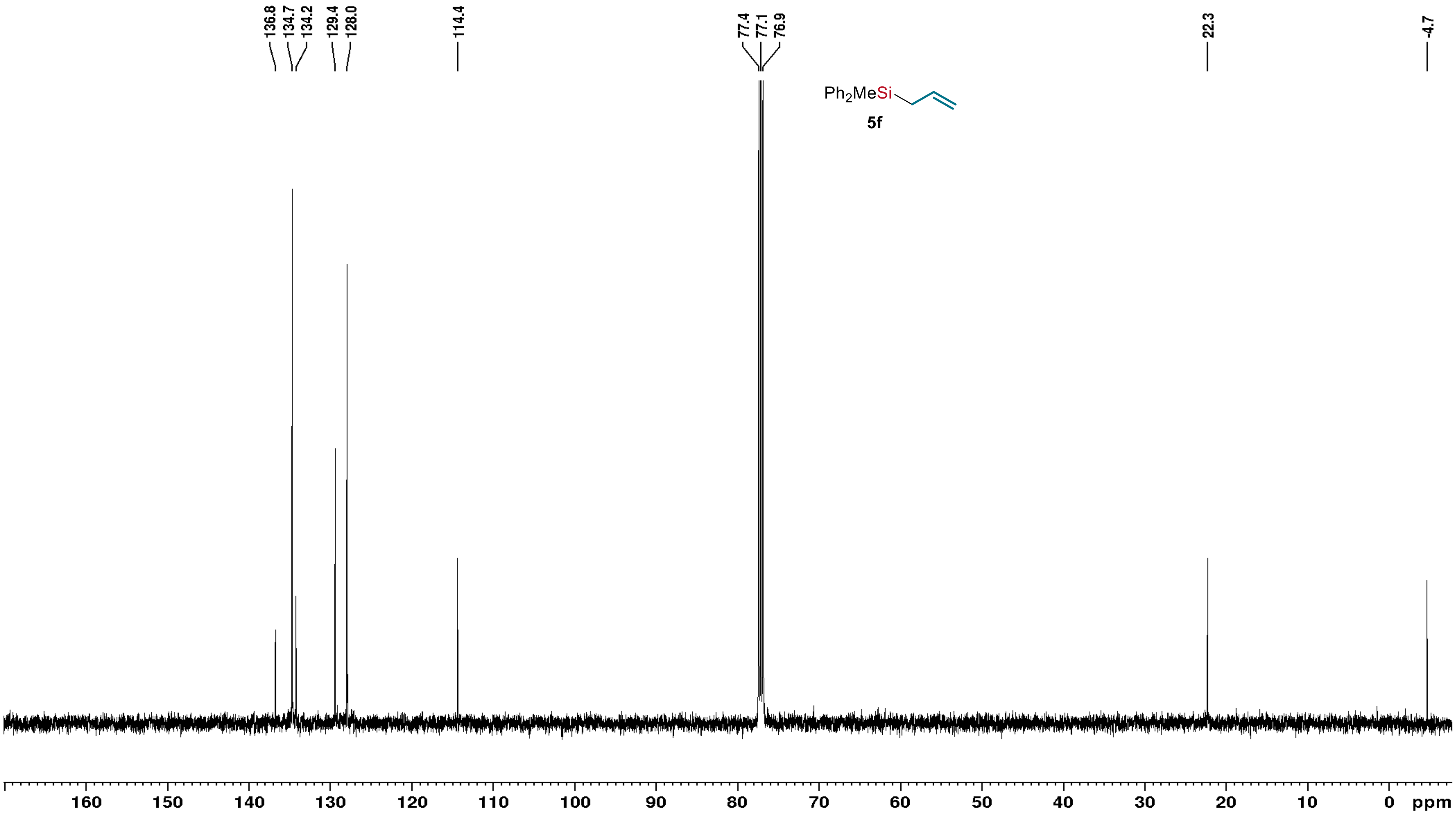

Figure S20. $^{1}H/^{29}Si$ **HMQC NMR spectrum (500/99 MHz, $CDCl_3$, 298 K, optimized for *J* = 7 Hz) of 5f**

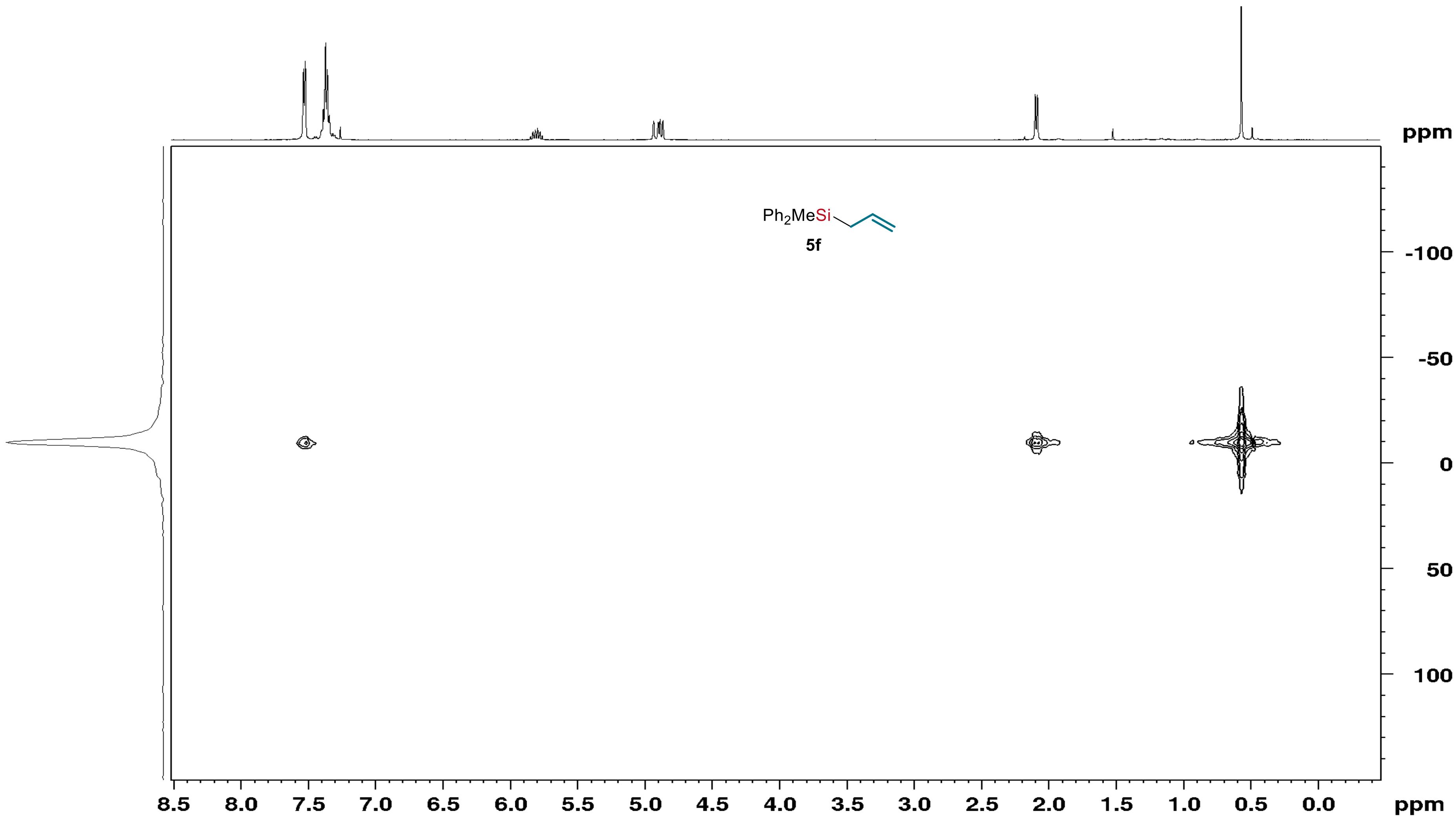

Figure S21. ^{1}H NMR spectrum (500 MHz, $CDCl_3$, 298 K) of Allyltriisopropylsilane (5g)

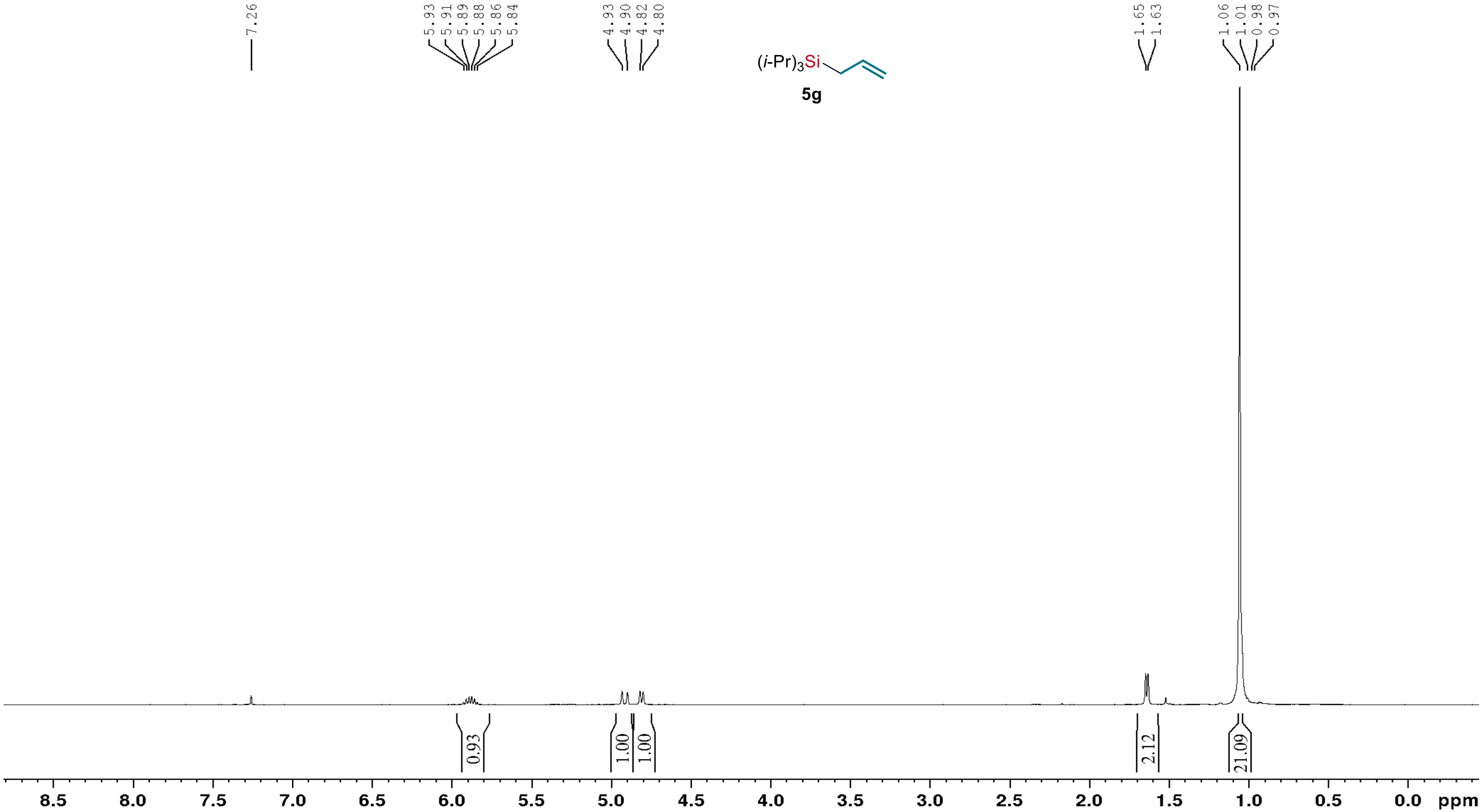

Figure S22. $^{13}C\{^1H\}$ **NMR spectrum (126 MHz, $CDCl_3$, 298 K) of Allyltriisopropylsilane (5g)**

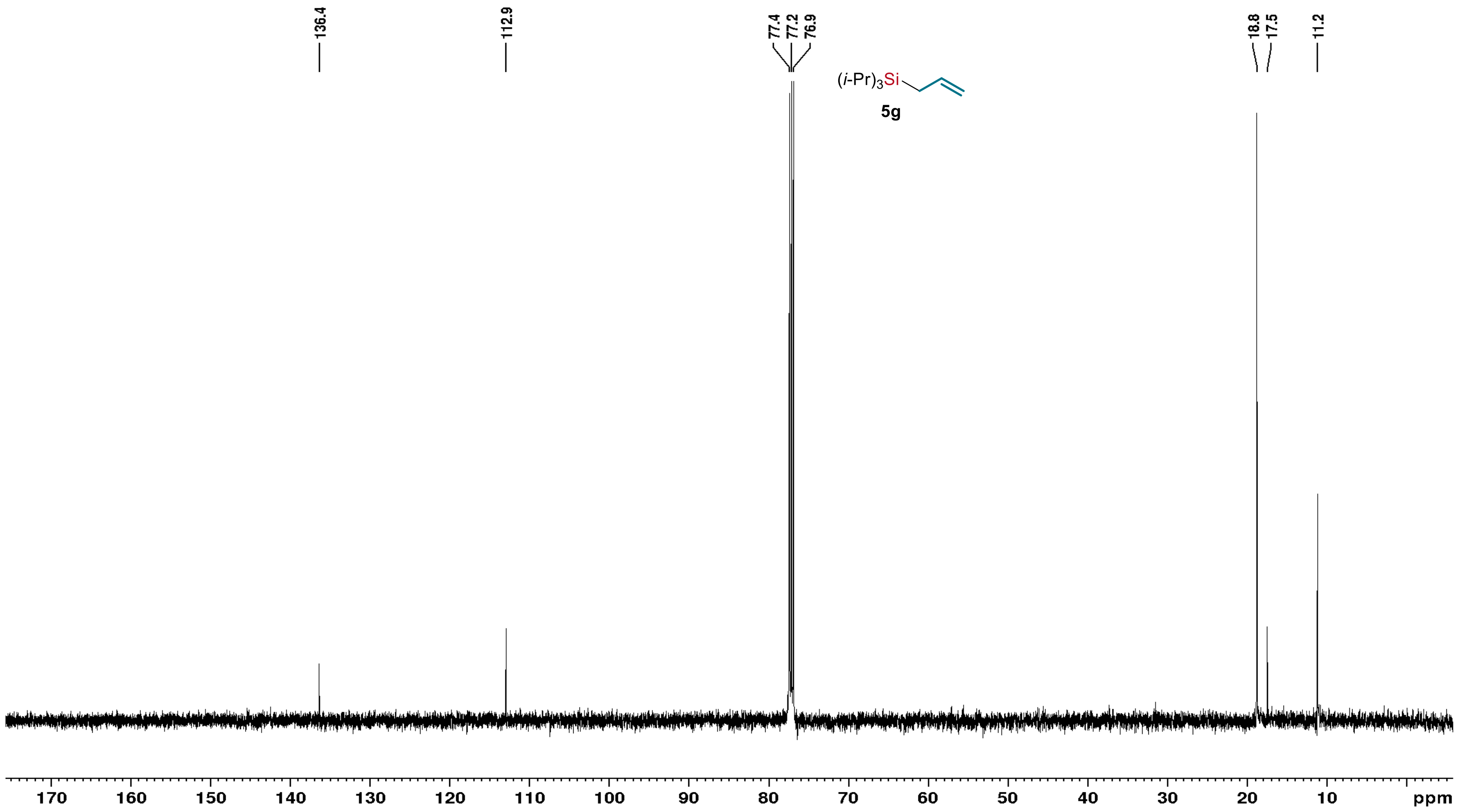

Figure S23. $^{1}H/^{29}Si$ **HMQC NMR spectrum (500/99 MHz, $CDCl_3$, 298 K, optimized for *J* = 7 Hz) of 5g**

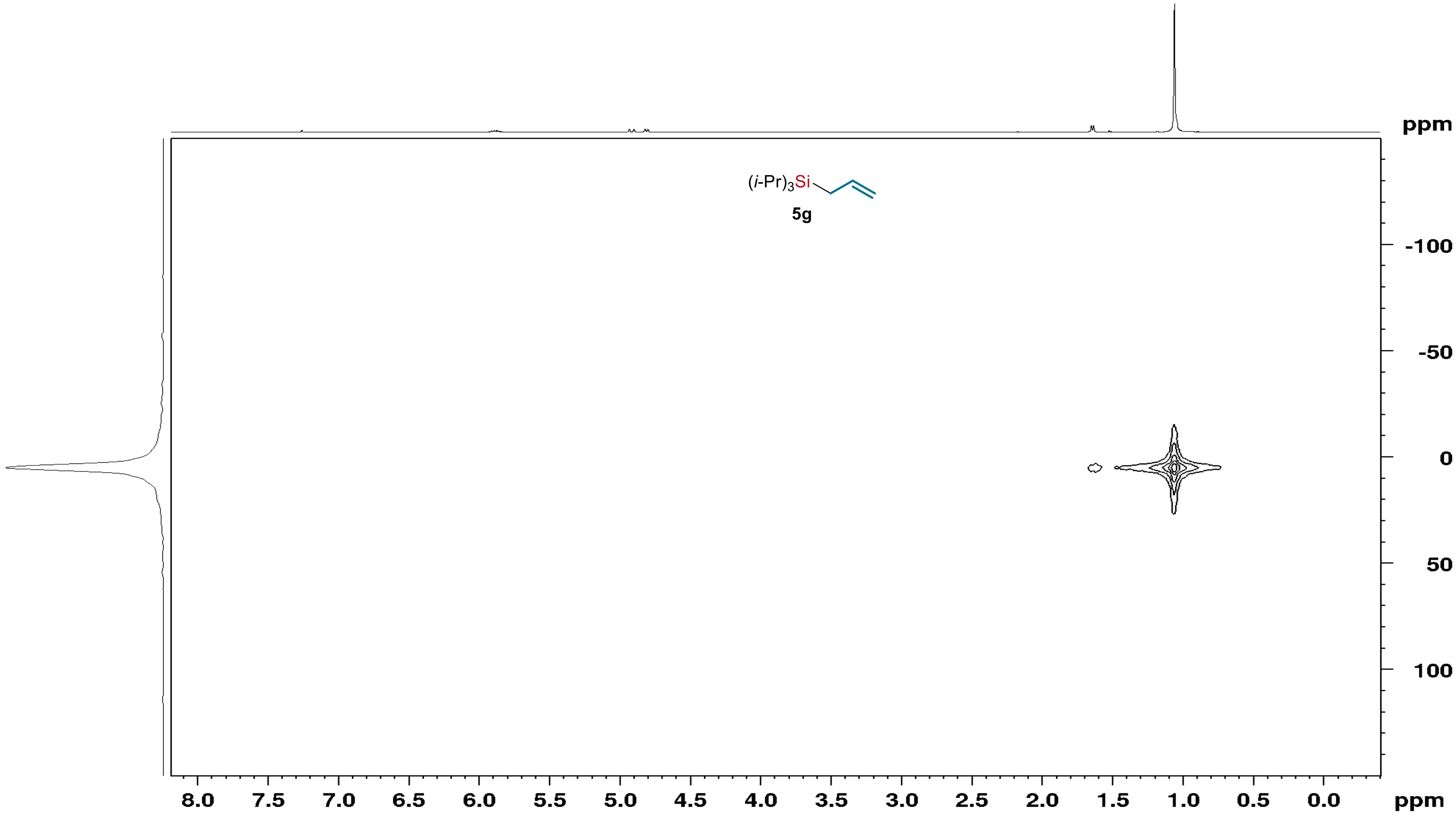

Figure S24. ^{1}H NMR spectrum (500 MHz, $CDCl_3$, 298 K) of (*E*)-triphenyl(3-phenylallyl)silane (3aa)

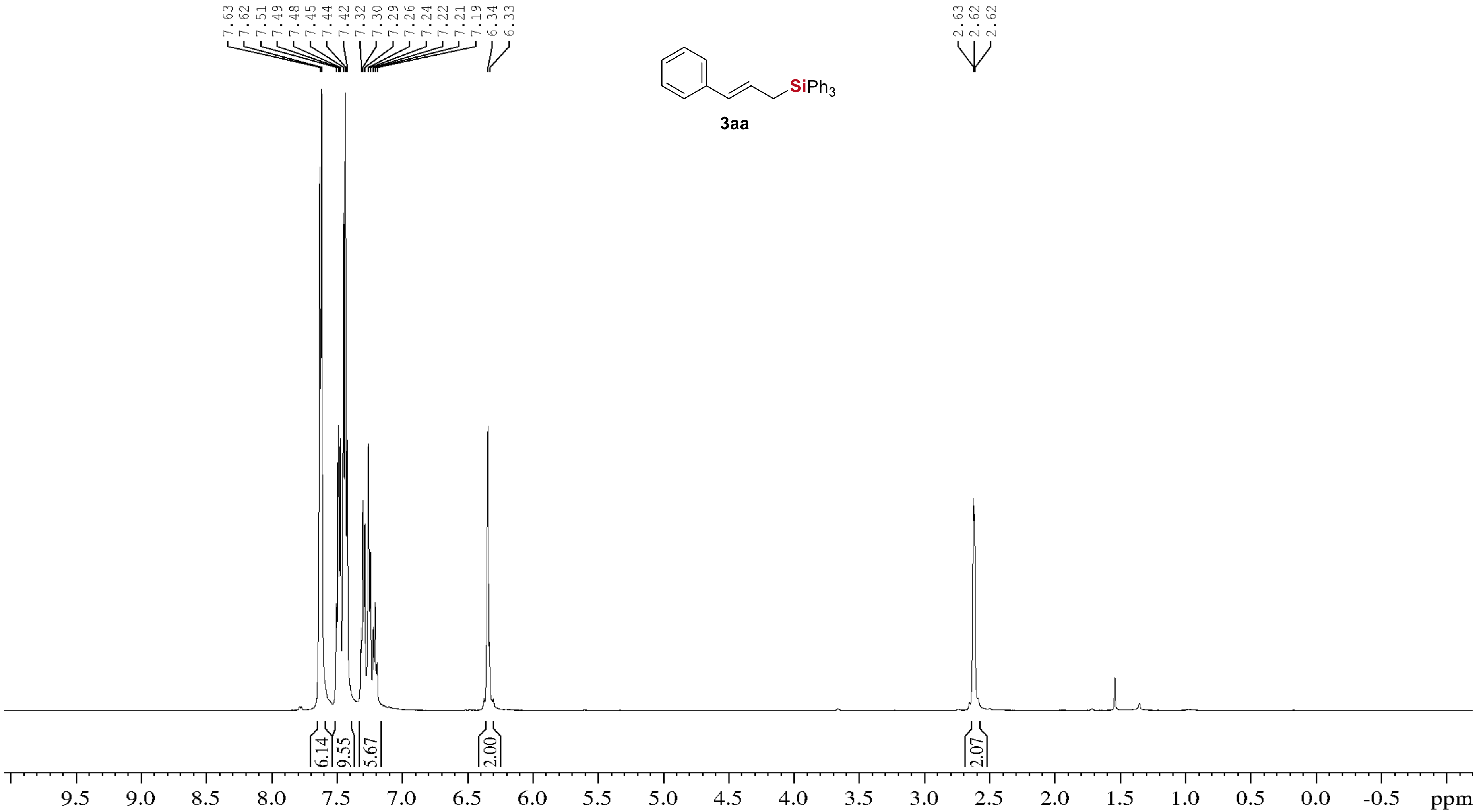

Figure S25. $^{13}C\{^1H\}$ NMR spectrum (126 MHz, $CDCl_3$, 298 K) of (*E*)-triphenyl(3-phenylallyl)silane (3aa)

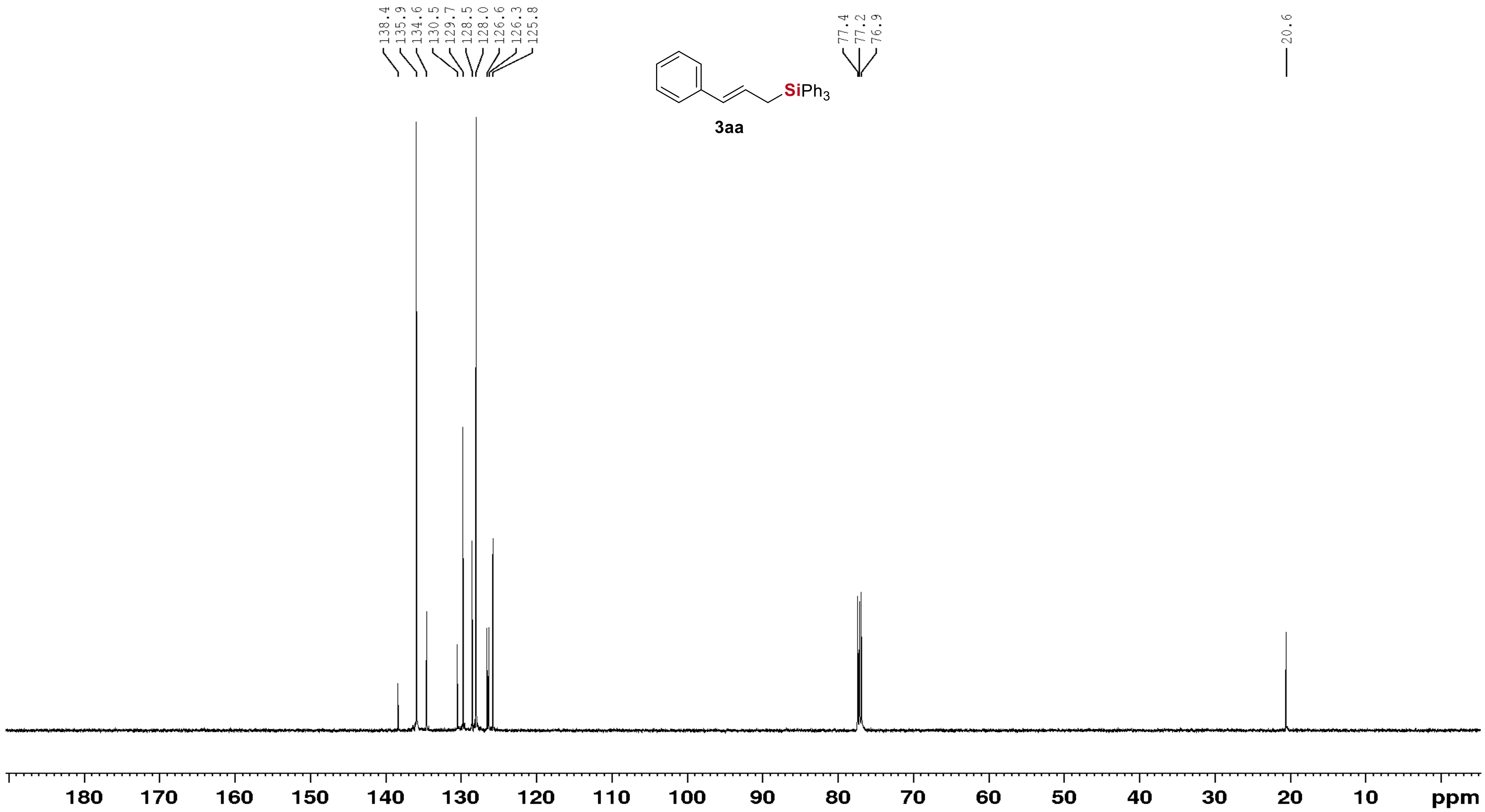

Figure S26. **$^{1}H/^{29}Si$ HMQC NMR spectrum (500/99 MHz, $CDCl_3$, 298 K, optimized for *J* = 7 Hz) of 3aa**

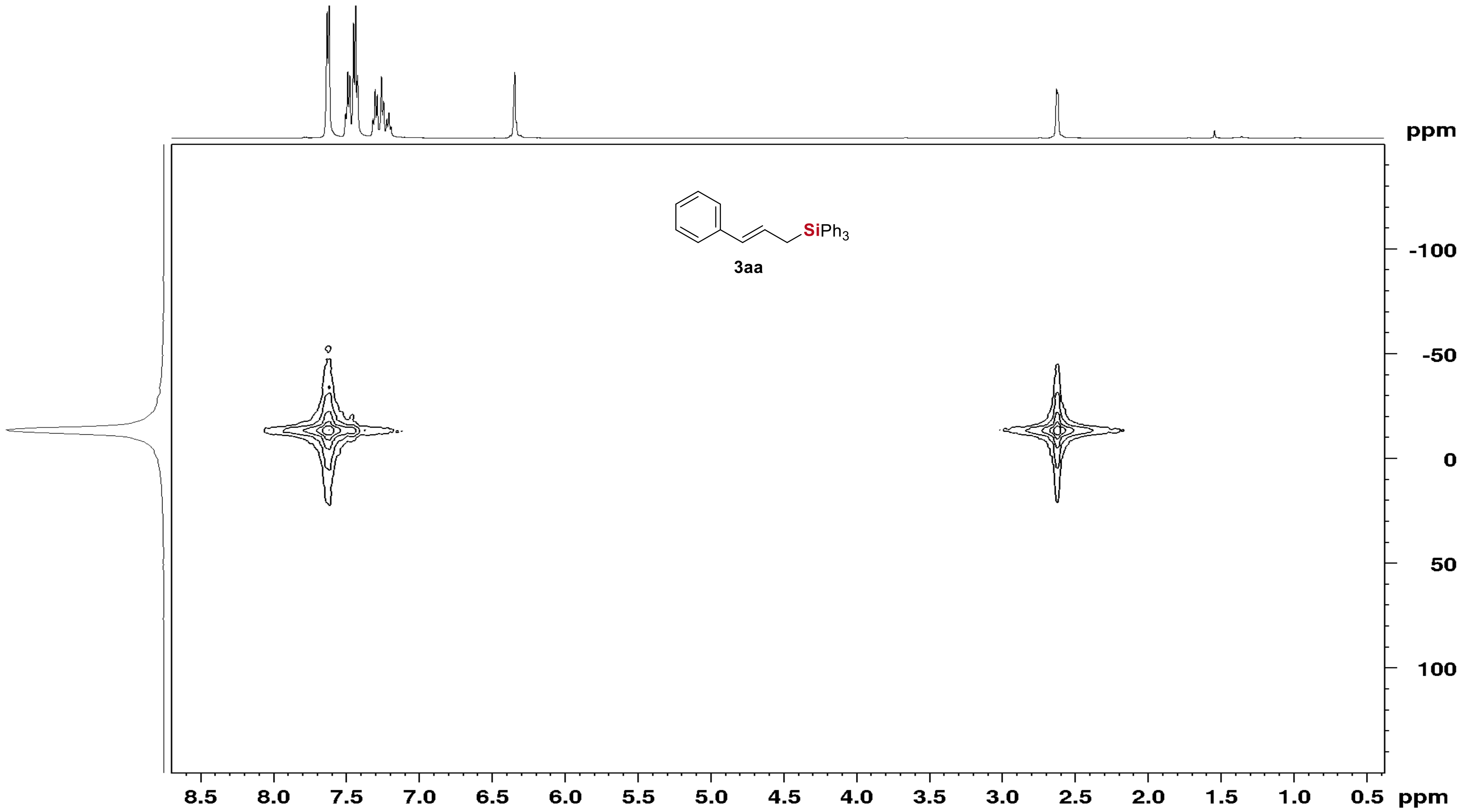

Figure S27. ^{1}H NMR spectrum (500 MHz, $CDCl_3$, 298 K) of (*E*)-triphenyl(3-(*p*-tolyl)allyl)silane (3ba)

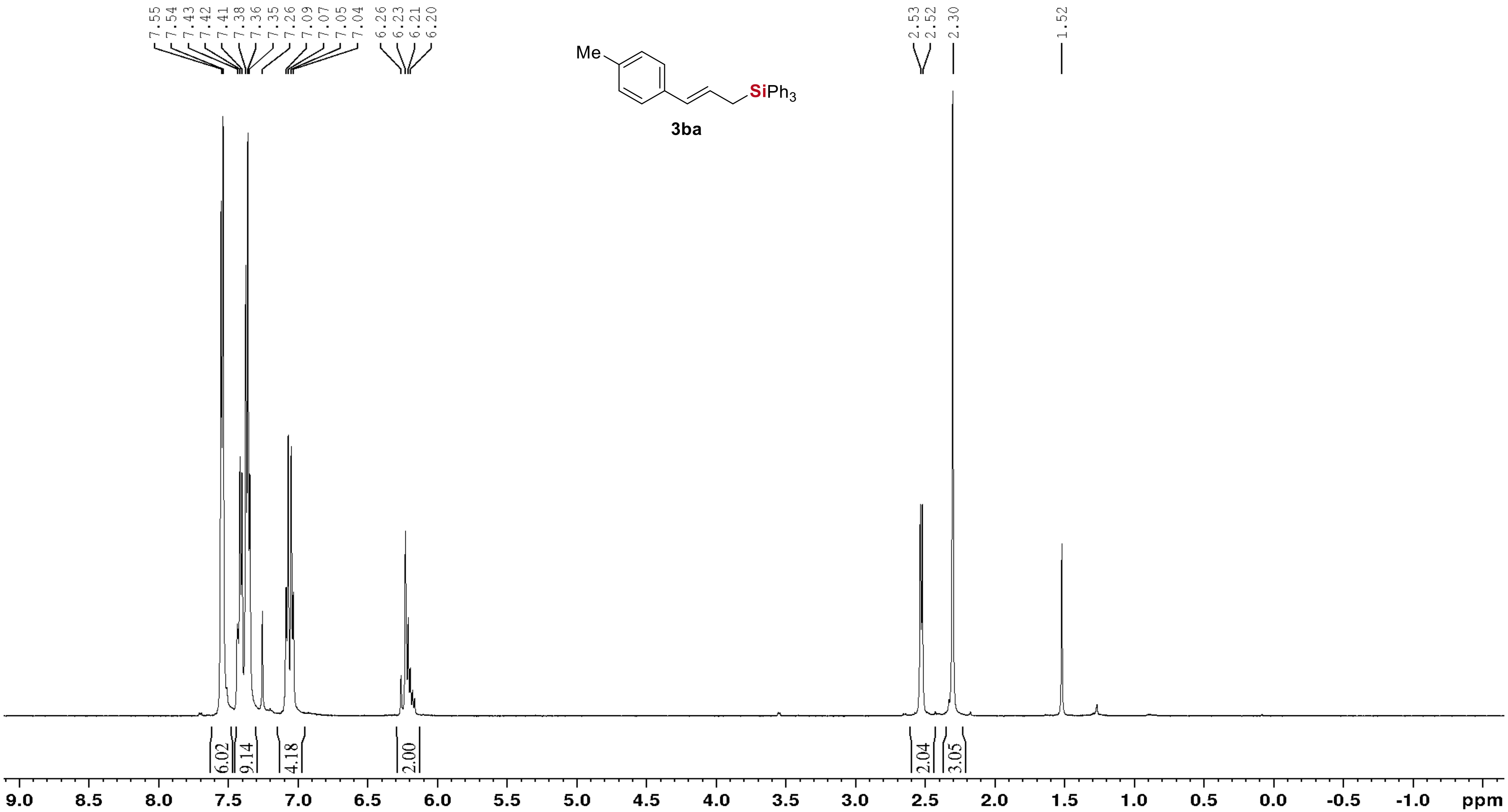

Figure S28. $^{13}C\{^1H\}$ **NMR spectrum (126 MHz, $CDCl_3$, 298 K) of (*E*)-triphenyl(3-(*p*-tolyl)allyl)silane (3ba)**

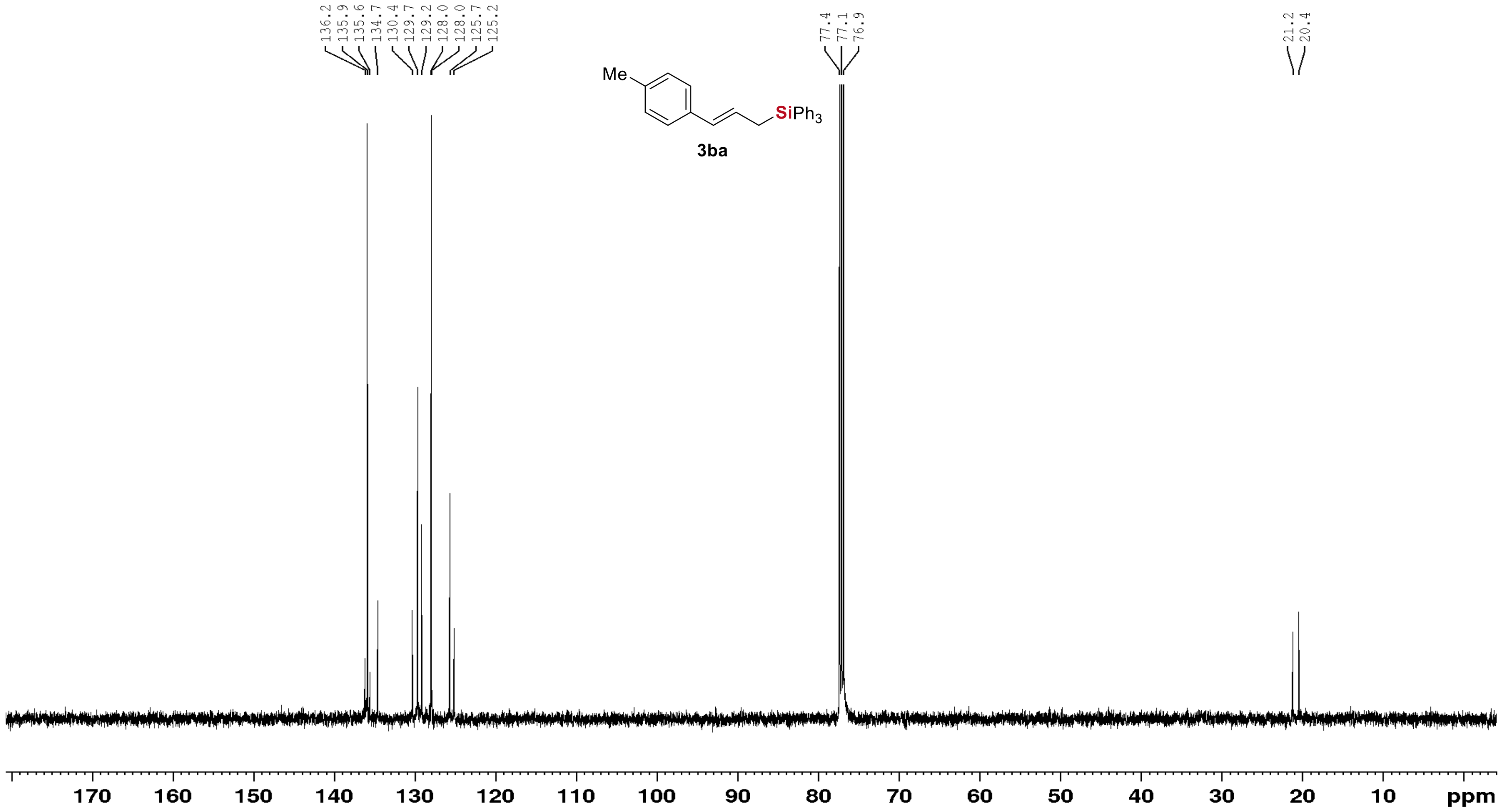

Figure S29. $^{1}H/^{29}Si$ **HMQC NMR spectrum (500/99 MHz,** $CDCl_3$**, 298 K, optimized for** J **= 7 Hz) of 3ba**

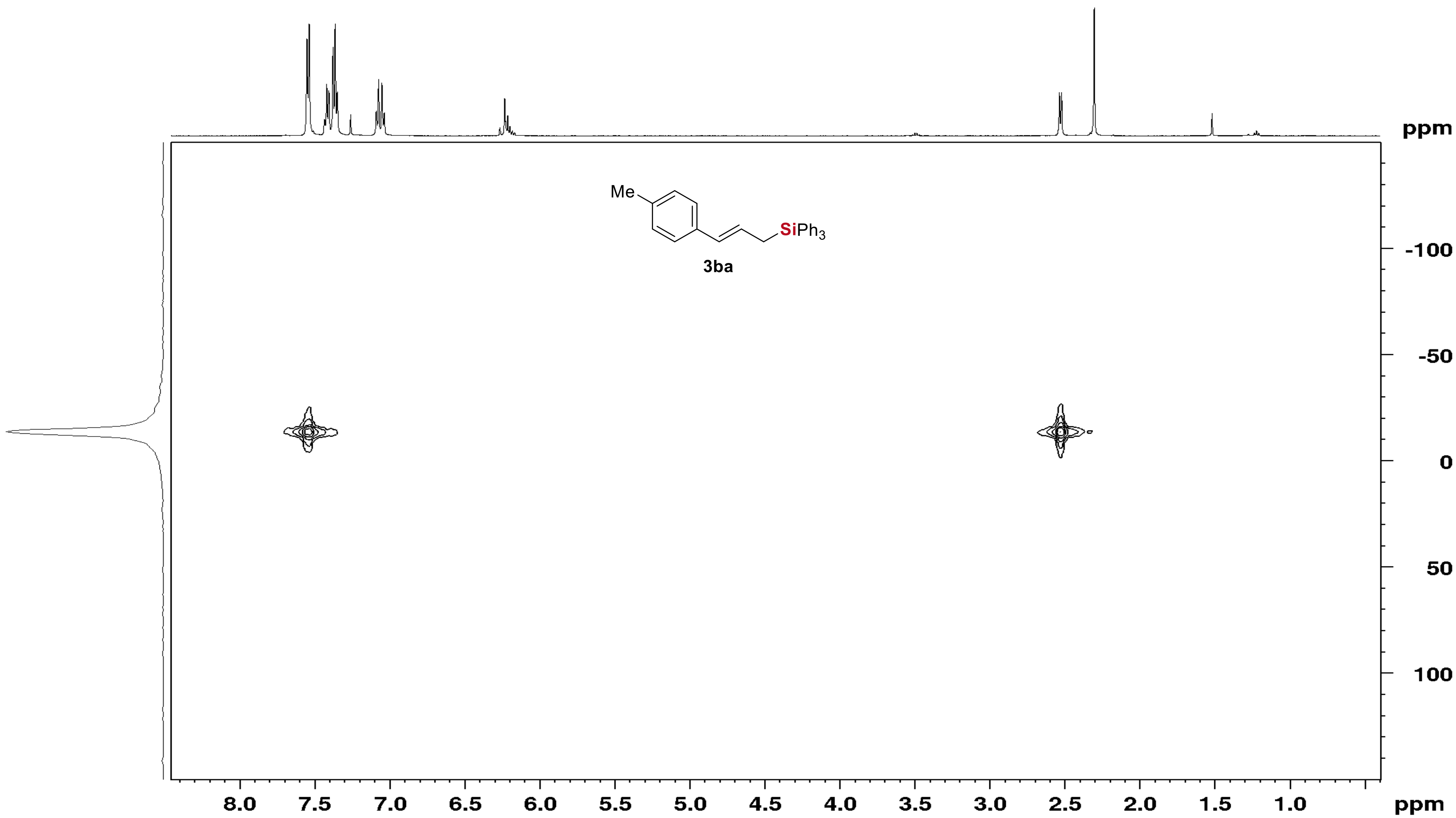

Figure S30. **^{1}H NMR spectrum (500 MHz, $CDCl_3$, 298 K) of (*E*)-(3-(4-(*tert*-butyl)phenyl)allyl)triphenylsilane (3ca)**

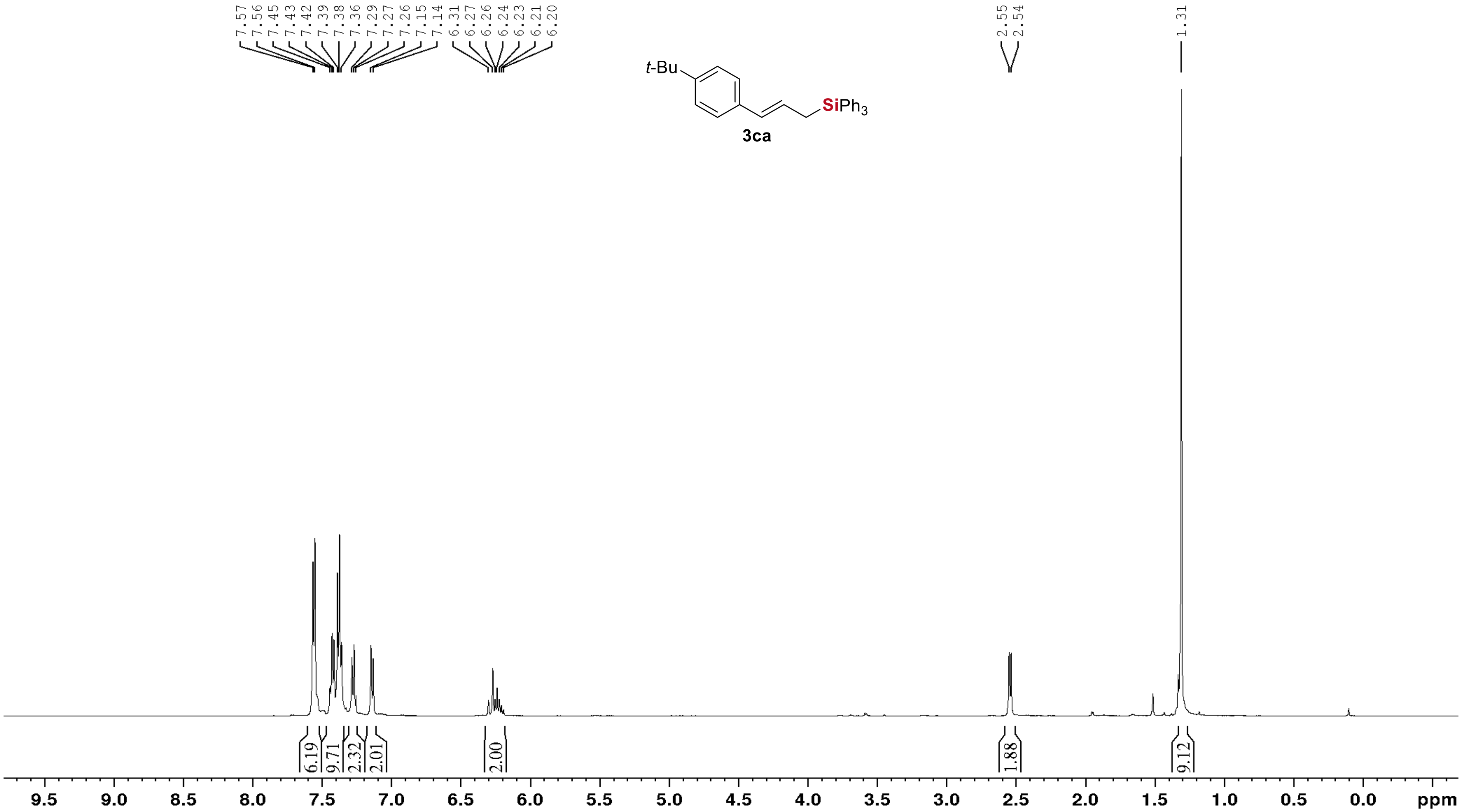

Figure S31. $^{13}C\{^{1}H\}$ NMR spectrum (126 MHz, $CDCl_3$, 298 K) of (*E*)-(3-(4-(*tert*-butyl)phenyl)allyl)triphenylsilane (3ca)

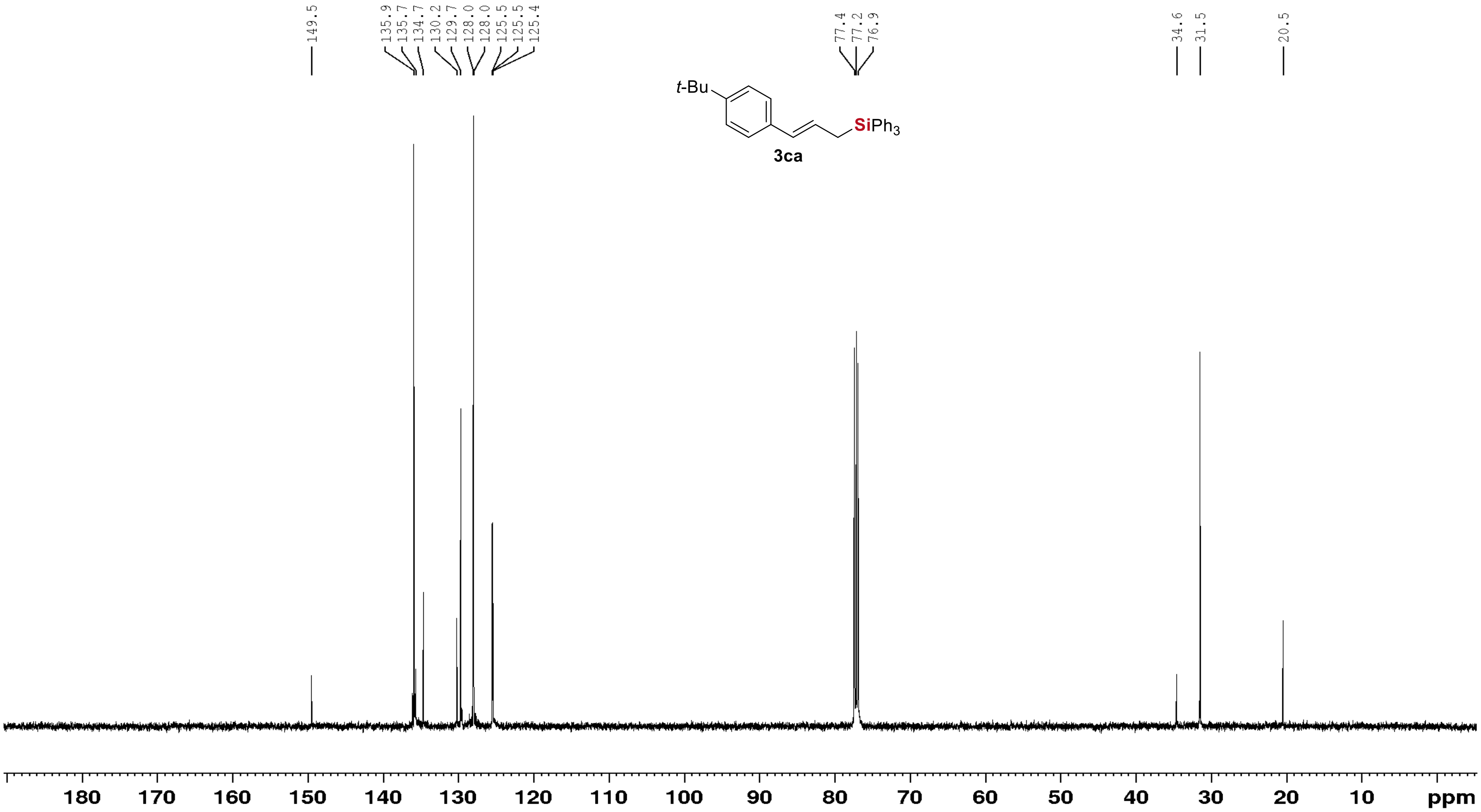

Figure S32. $^{1}H/^{29}Si$ HMQC NMR spectrum (500/99 MHz, $CDCl_3$, 298 K, optimized for *J* = 7 Hz) of 3ca

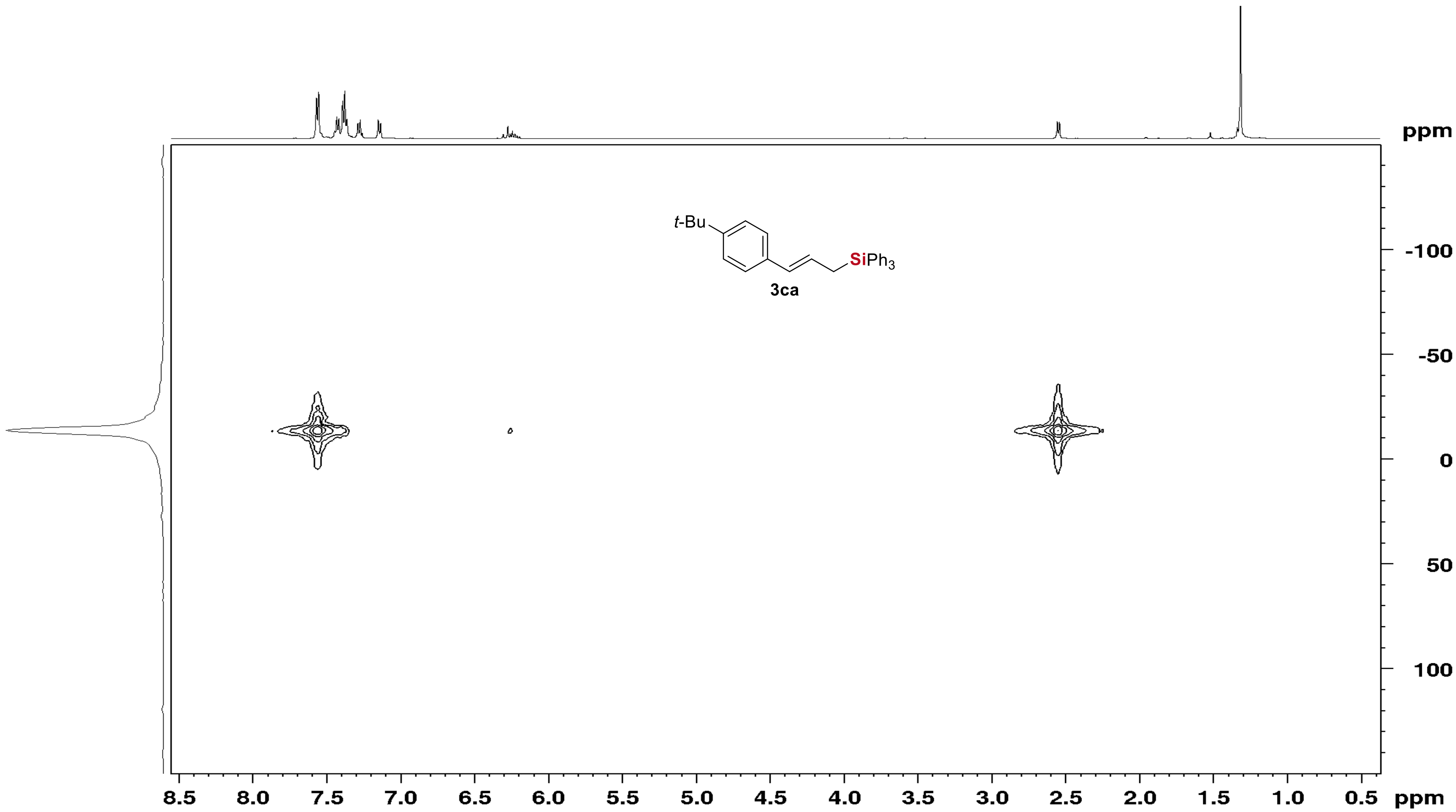

Figure S33. ^{1}H NMR spectrum (500 MHz, $CDCl_3$, 298 K) of (*E*)-trimethyl(4-(3-(triphenylsilyl)prop-1-en-1-yl)phenyl)silane (3da)

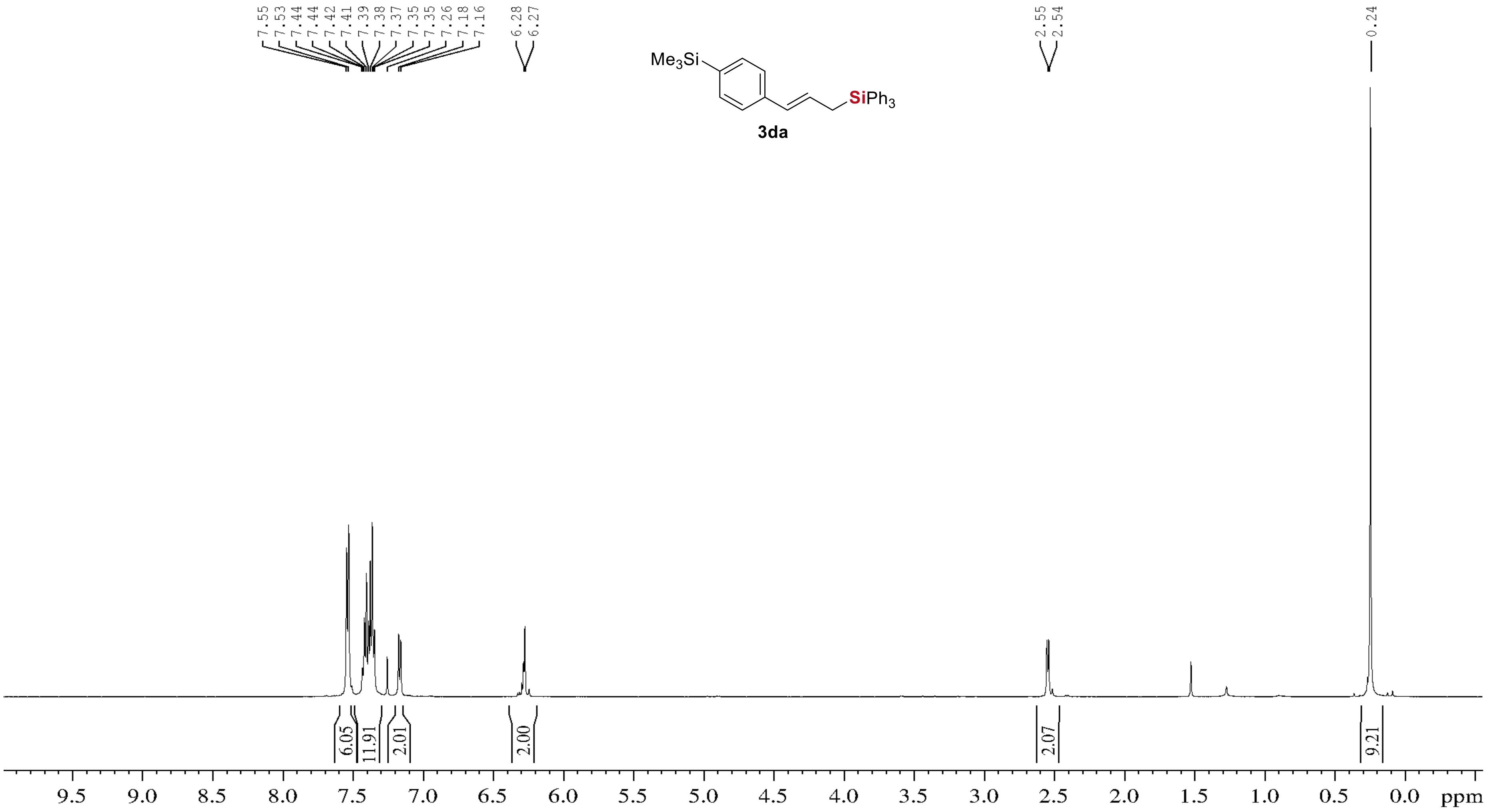

Figure S34. $^{13}C\{^1H\}$ **NMR spectrum (126 MHz, $CDCl_3$, 298 K) of (*E*)-trimethyl(4-(3-(triphenylsilyl)prop-1-en-1-yl)phenyl)silane (3da)**

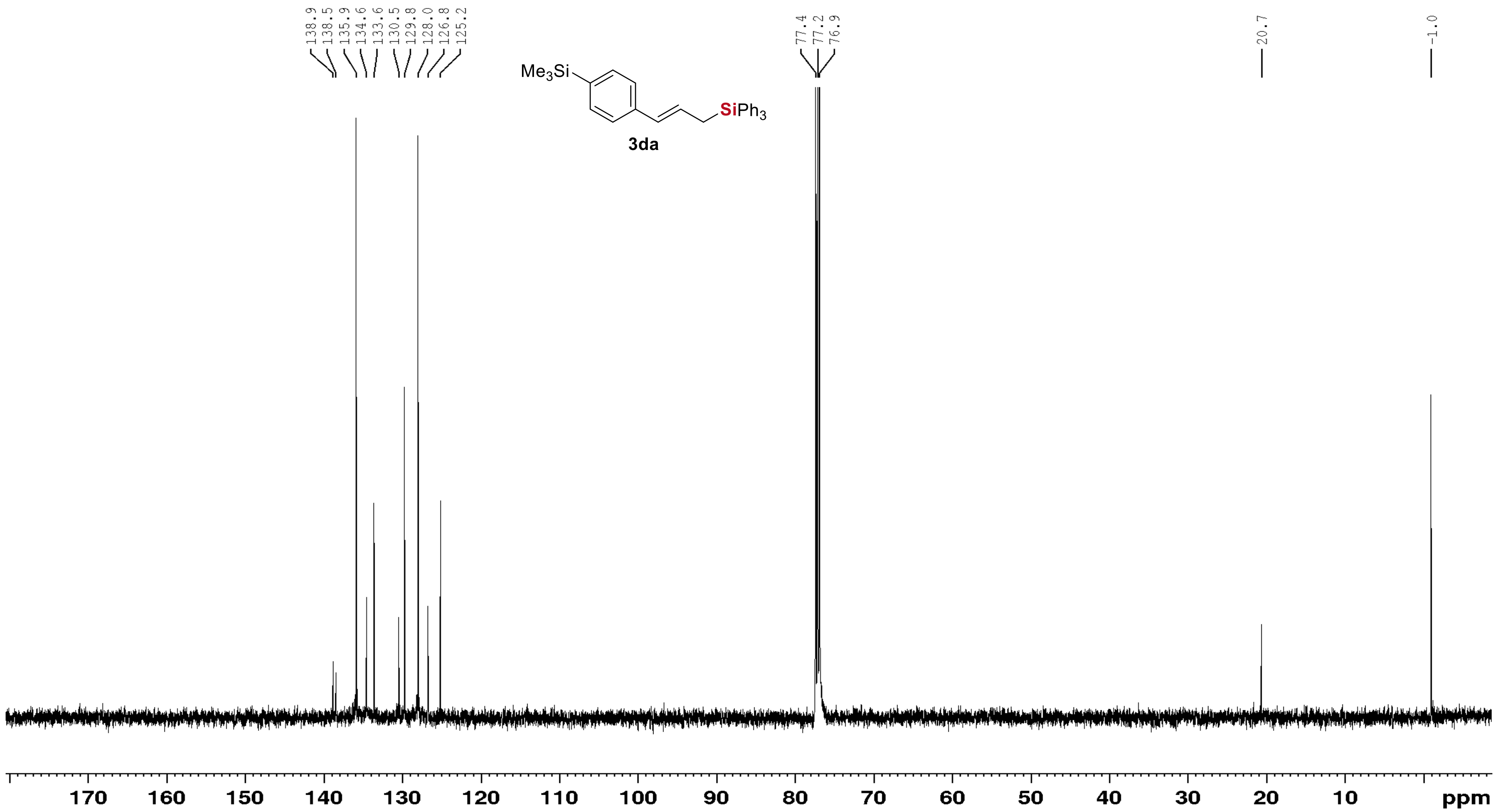

Figure S35. **$^{1}H/^{29}Si$ HMQC NMR spectrum (500/99 MHz, $CDCl_3$, 298 K, optimized for *J* = 7 Hz) of 3da**

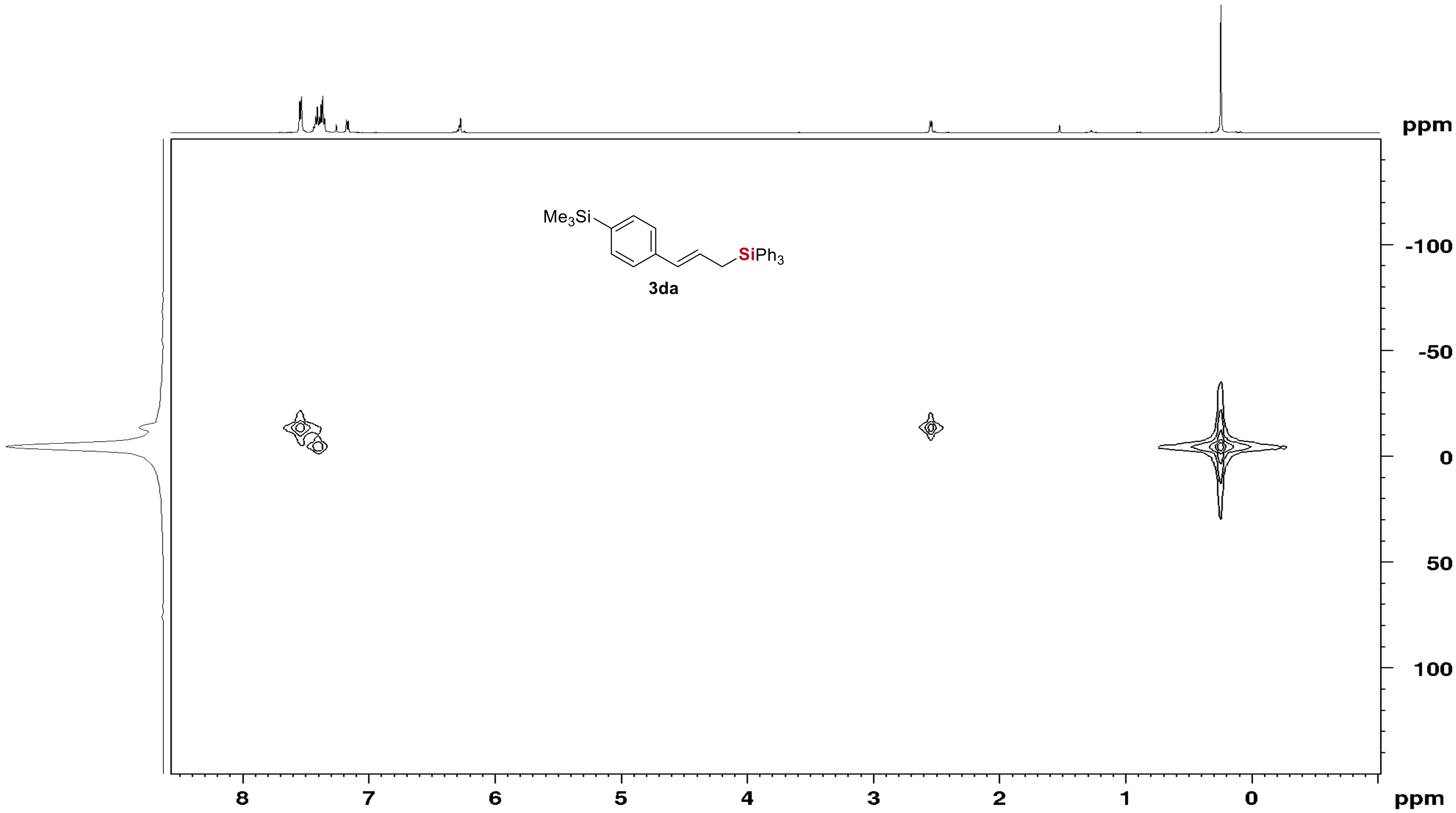

Figure S36. ^{1}H NMR spectrum (500 MHz, $CDCl_3$, 298 K) of (*E*)-(3-(4-CF_3phenyl)allyl)triphenylsilane (3ea)

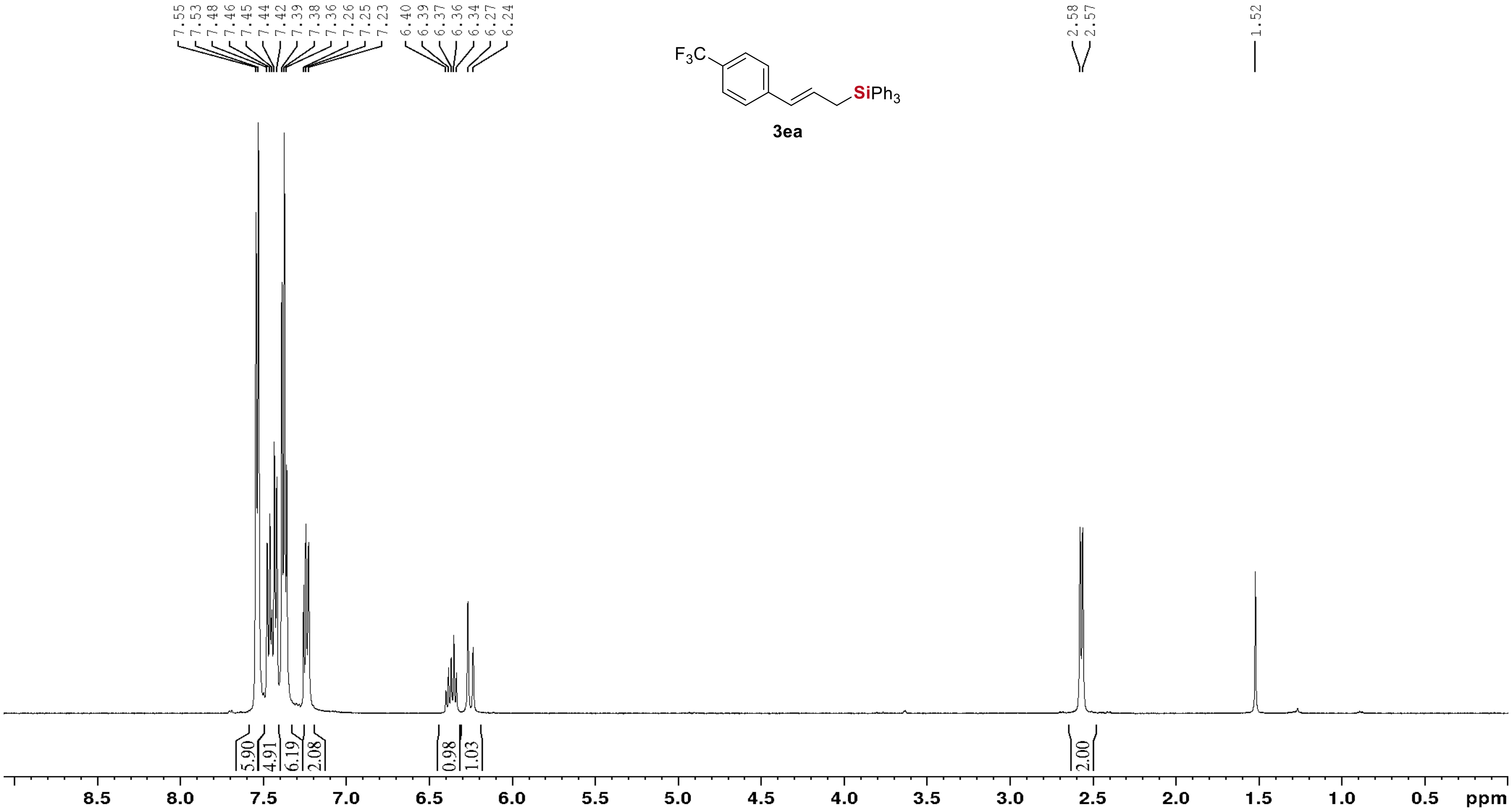

Figure S37. ^{19}F NMR (471 MHz, $CDCl_3$, 298 K) of (*E*)-(3-(4-CF_3phenyl)allyl)triphenylsilane (3ea)

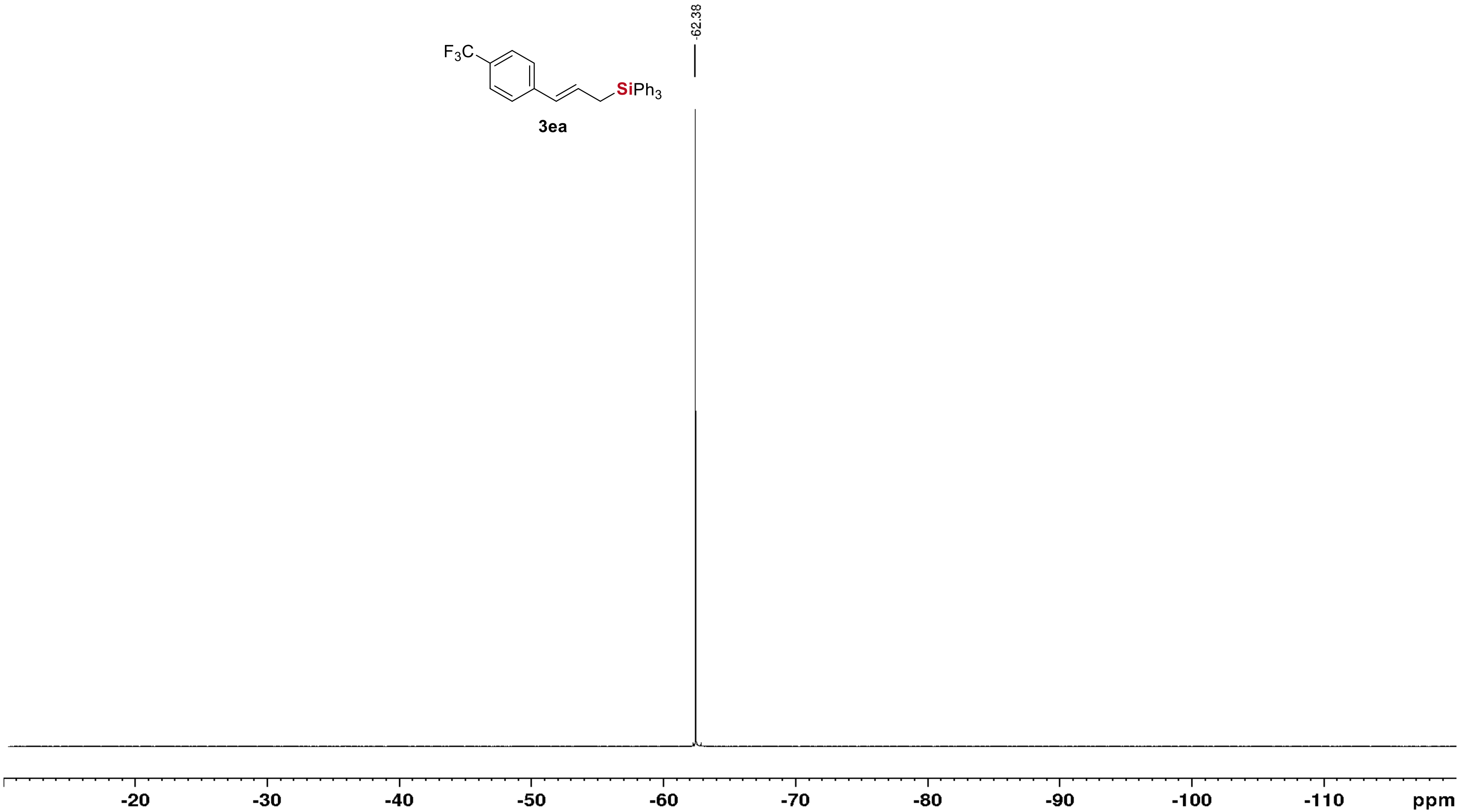

Figure S38. $^{13}C\{^{1}H\}$ **NMR spectrum (126 MHz, $CDCl_3$, 298 K) of (*E*)-(3-(4-CF_3phenyl)allyl)triphenylsilane (3ea)**

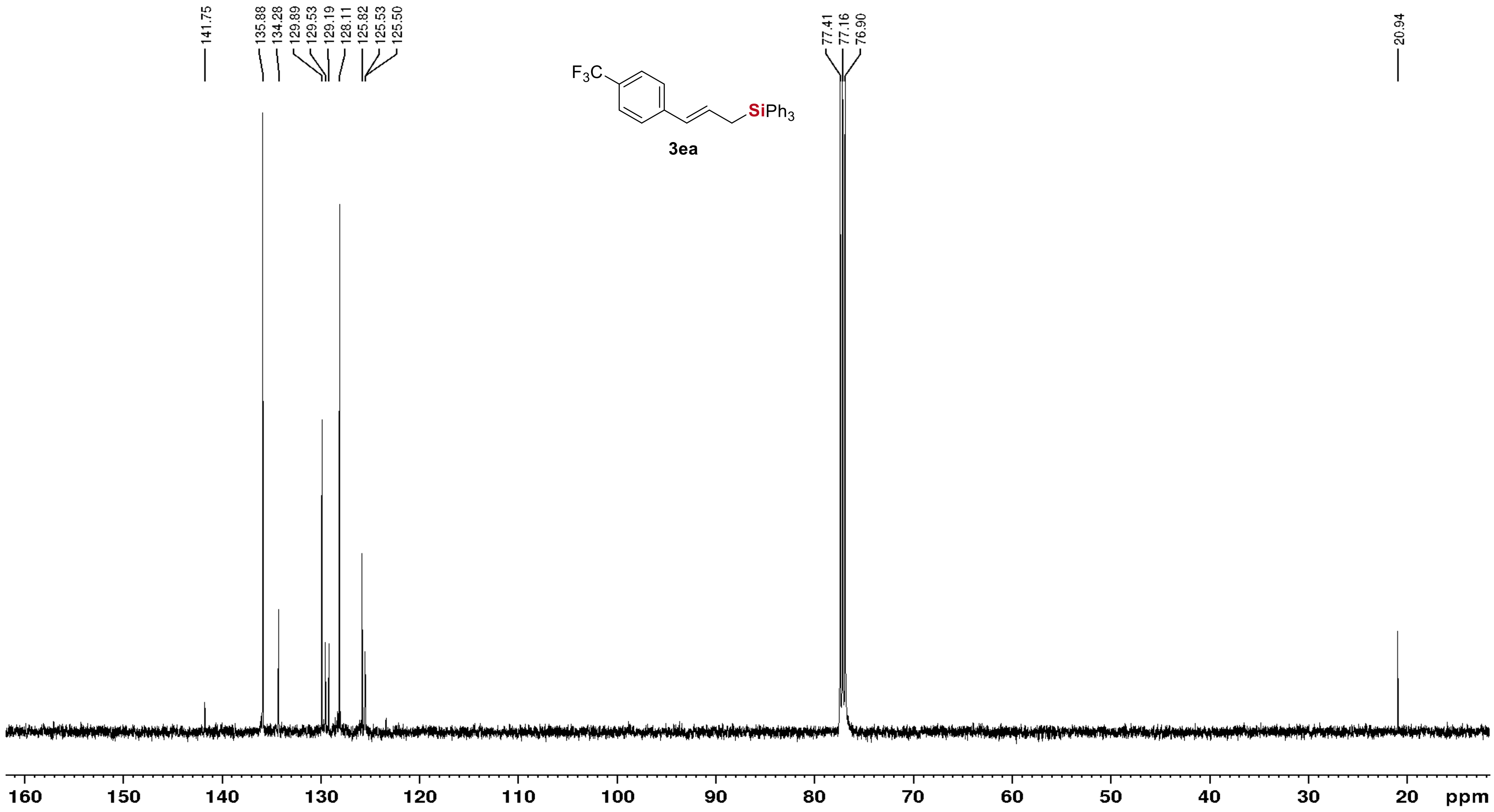

Figure S39. $^1H/^{29}Si$ HMQC NMR spectrum (500/99 MHz, $CDCl_3$, 298 K, optimized for *J* = 7 Hz) of 3ea

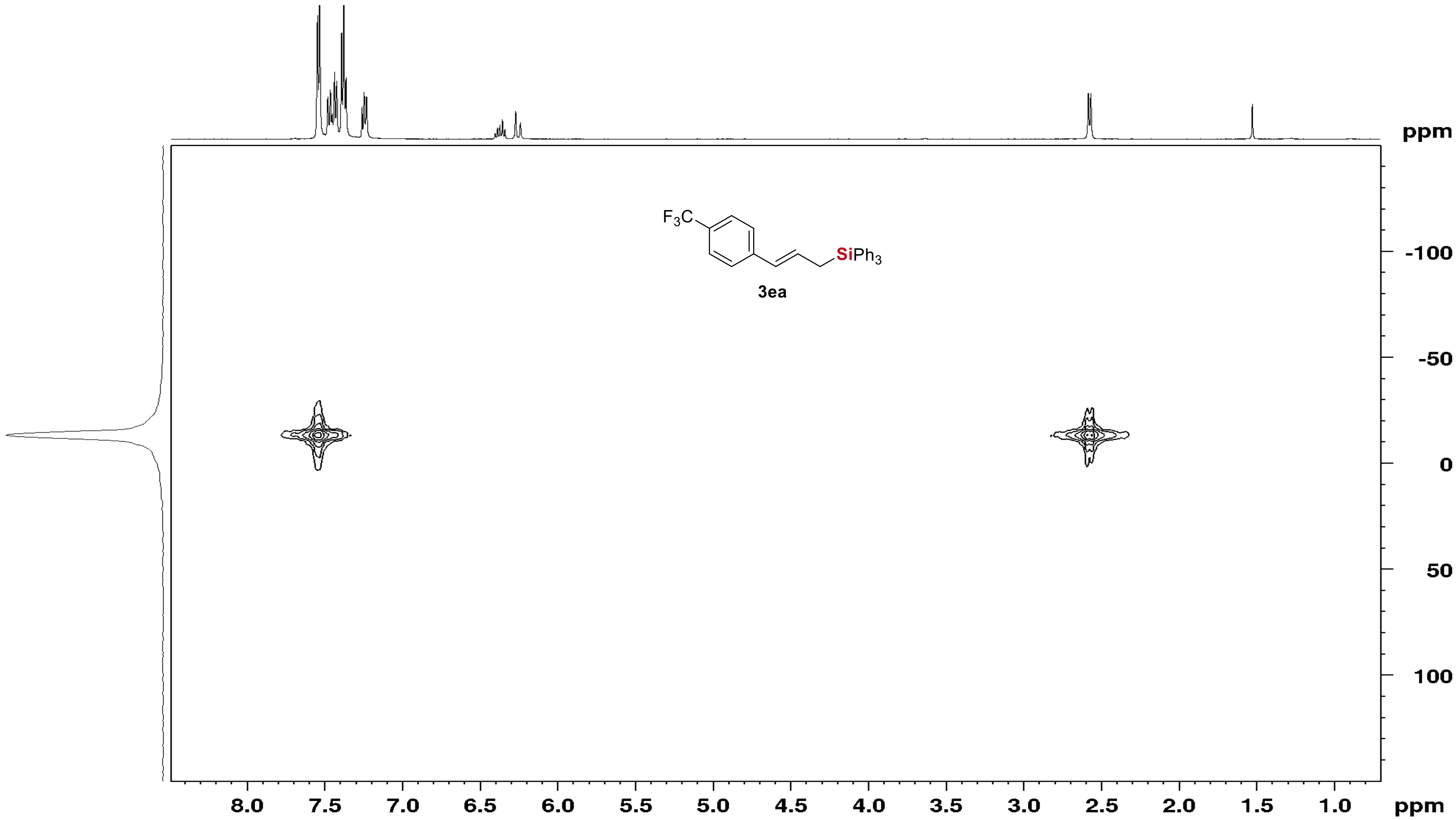

Figure S40. ¹H NMR spectrum (500 MHz, $CDCl_3$, 298 K) of (*E*)-(3-(4-fluorophenyl)allyl)triphenylsilane (3fa)

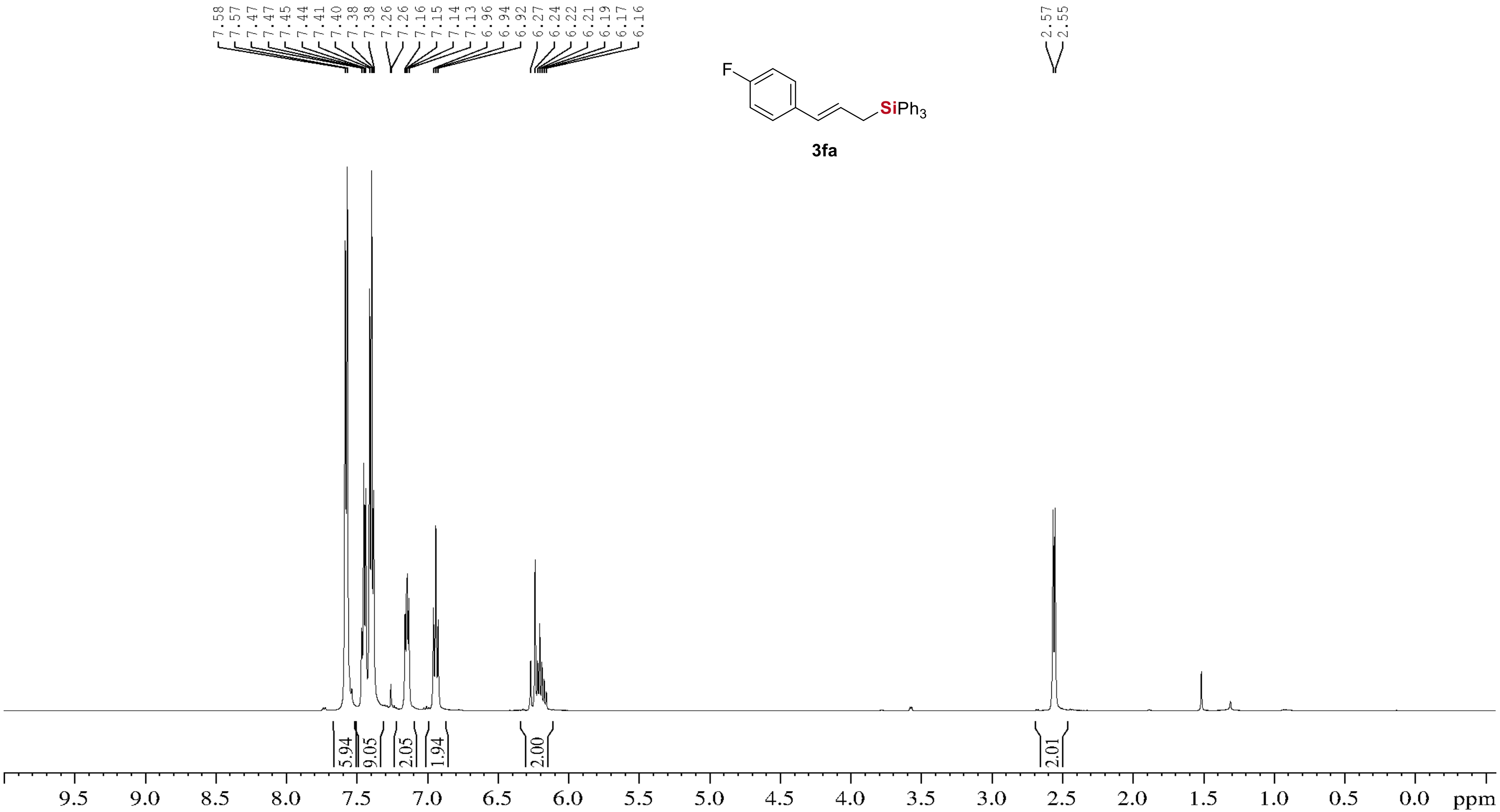

Figure S41. **^{19}F NMR (471 MHz, $CDCl_3$, 298 K) of (*E*)-(3-(4-fluorophenyl)allyl)triphenylsilane (3fa)**

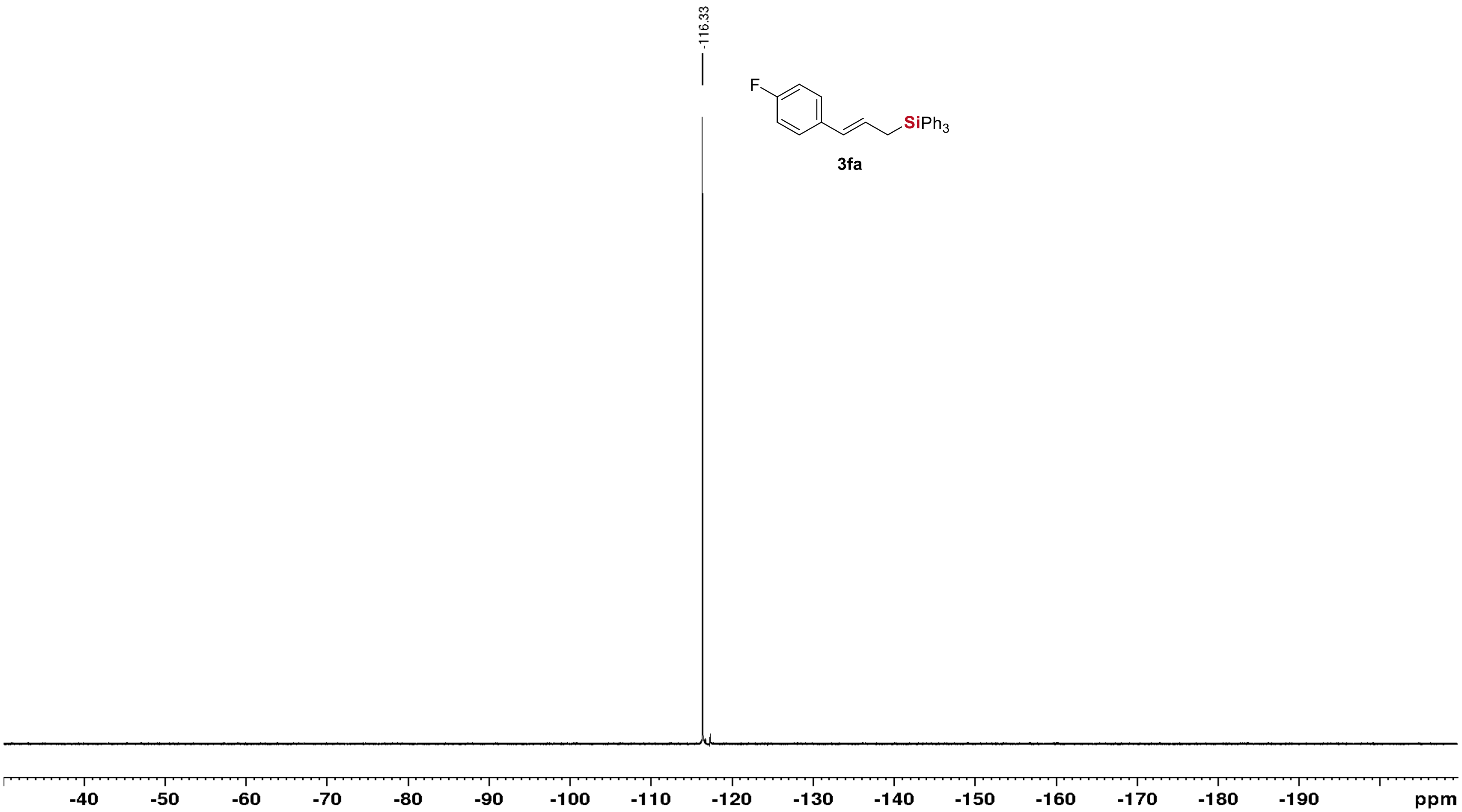

Figure S42. **$^{13}C\{^{1}H\}$ NMR spectrum (126 MHz, $CDCl_3$, 298 K) of (*E*)-(3-(4-fluorophenyl)allyl)triphenylsilane (3fa)**

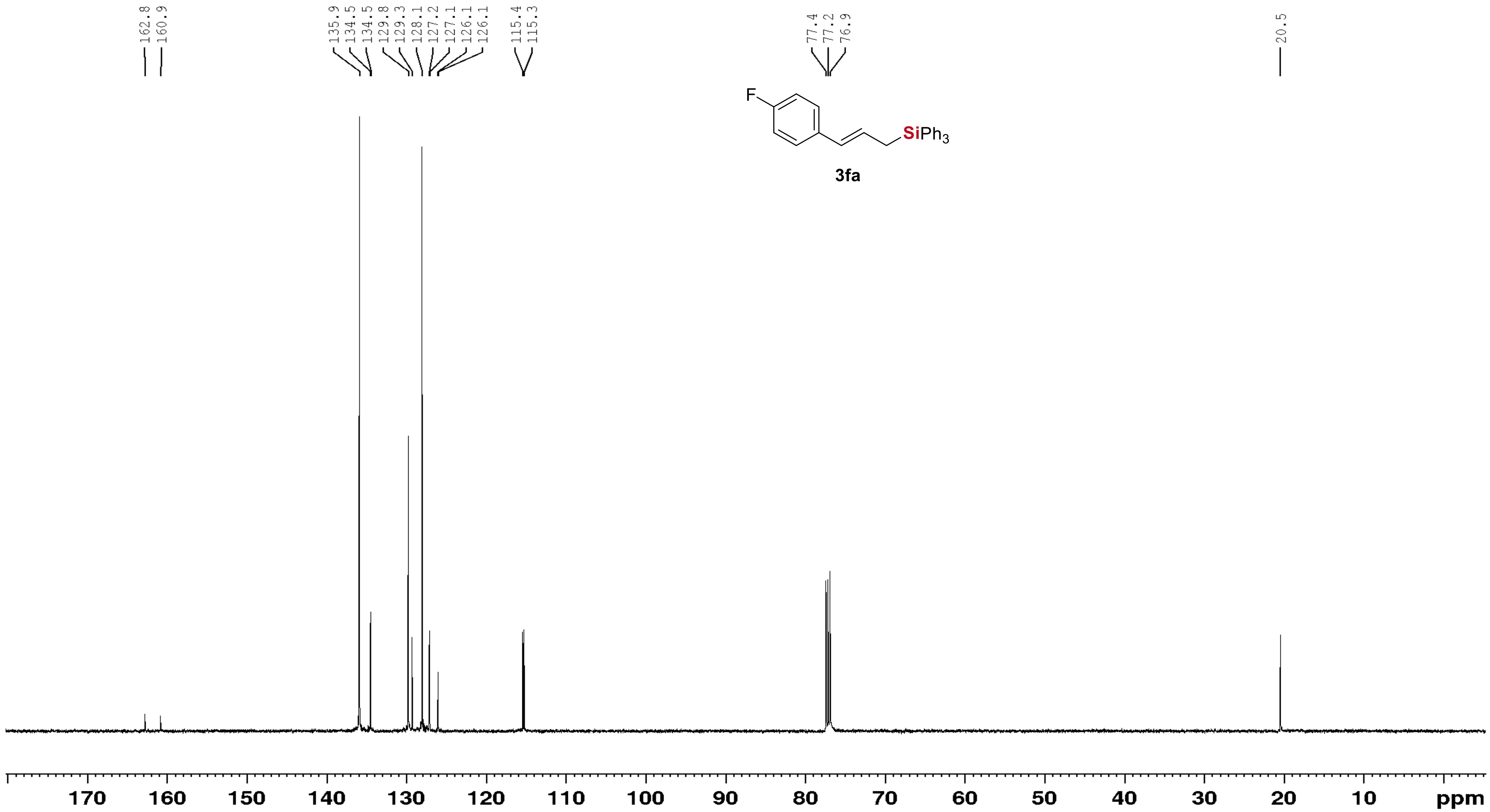

Figure S43. **$^{1}H/^{29}Si$ HMQC NMR spectrum (500/99 MHz, $CDCl_3$, 298 K, optimized for J = 7 Hz) of 3fa**

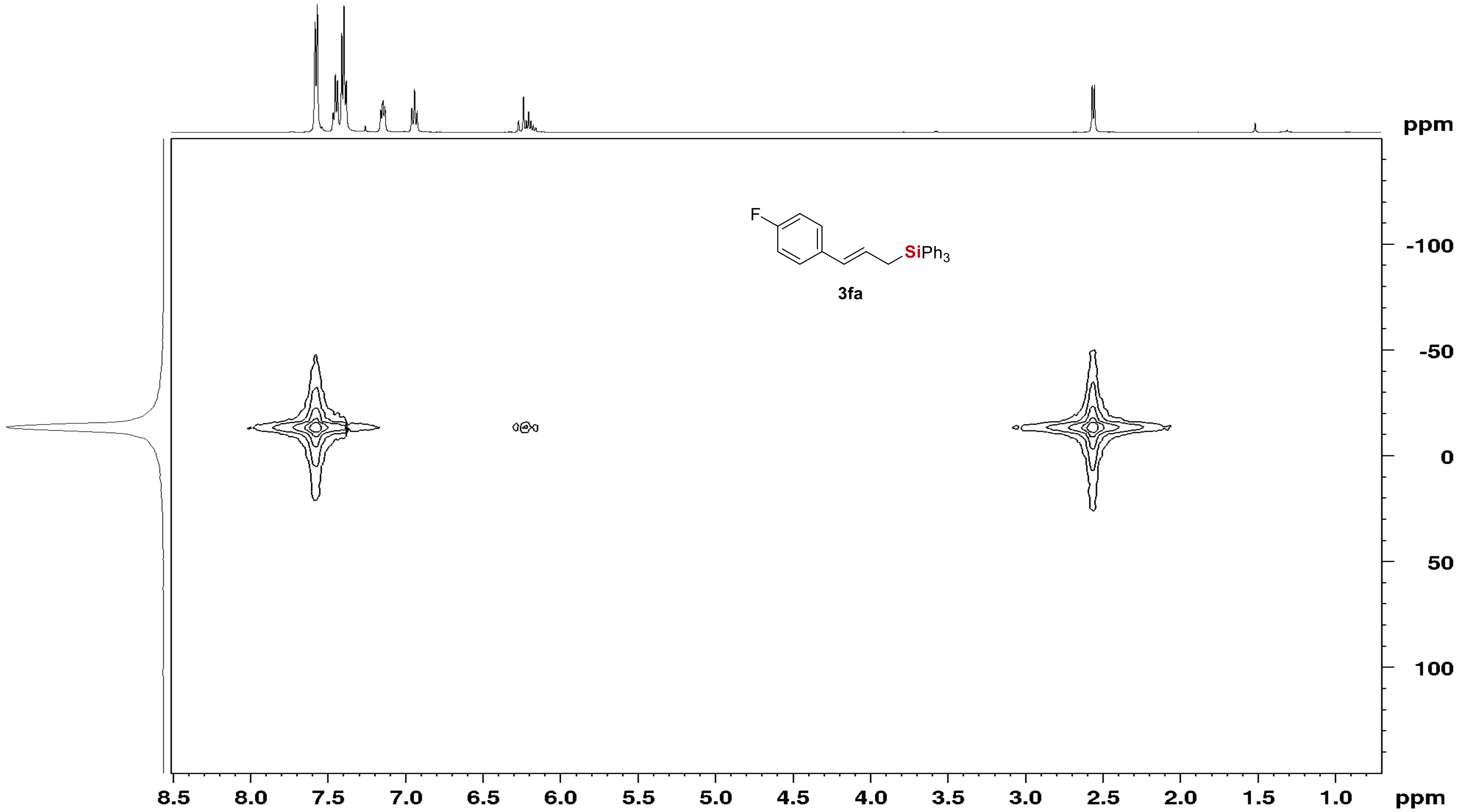

Figure S44. ^{1}H NMR spectrum (500 MHz, $CDCl_3$, 298 K) of (*E*)-(3-(4-chlorophenyl)allyl)triphenylsilane (3ga)

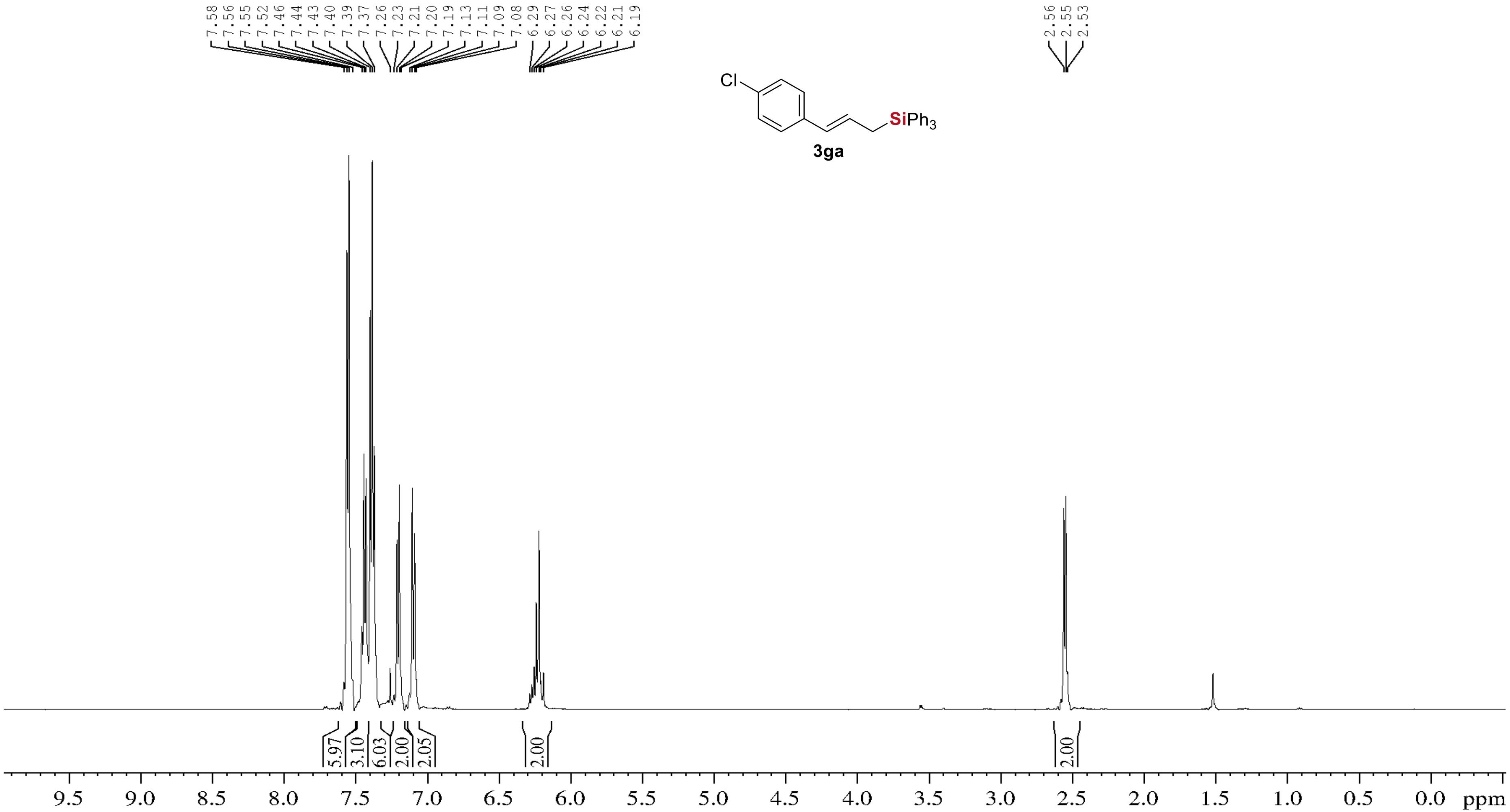

Figure S45. $^{13}C\{^1H\}$ NMR spectrum (126 MHz, $CDCl_3$, 298 K) of (*E*)-(3-(4-chlorophenyl)allyl)triphenylsilane (3ga)

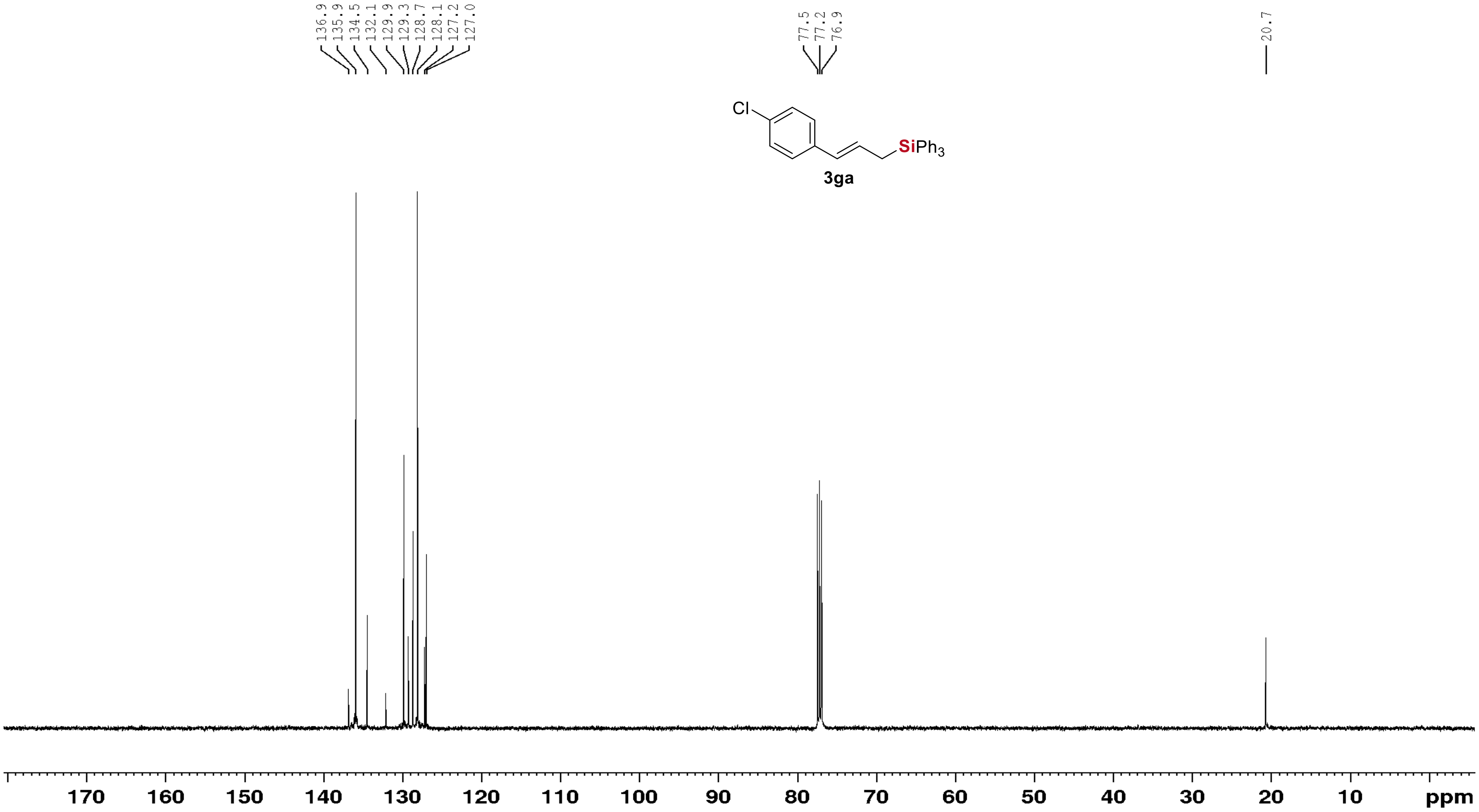

Figure S46. $^{1}H/^{29}Si$ **HMQC NMR spectrum (500/99 MHz, $CDCl_3$, 298 K, optimized for *J* = 7 Hz) of 3ga**

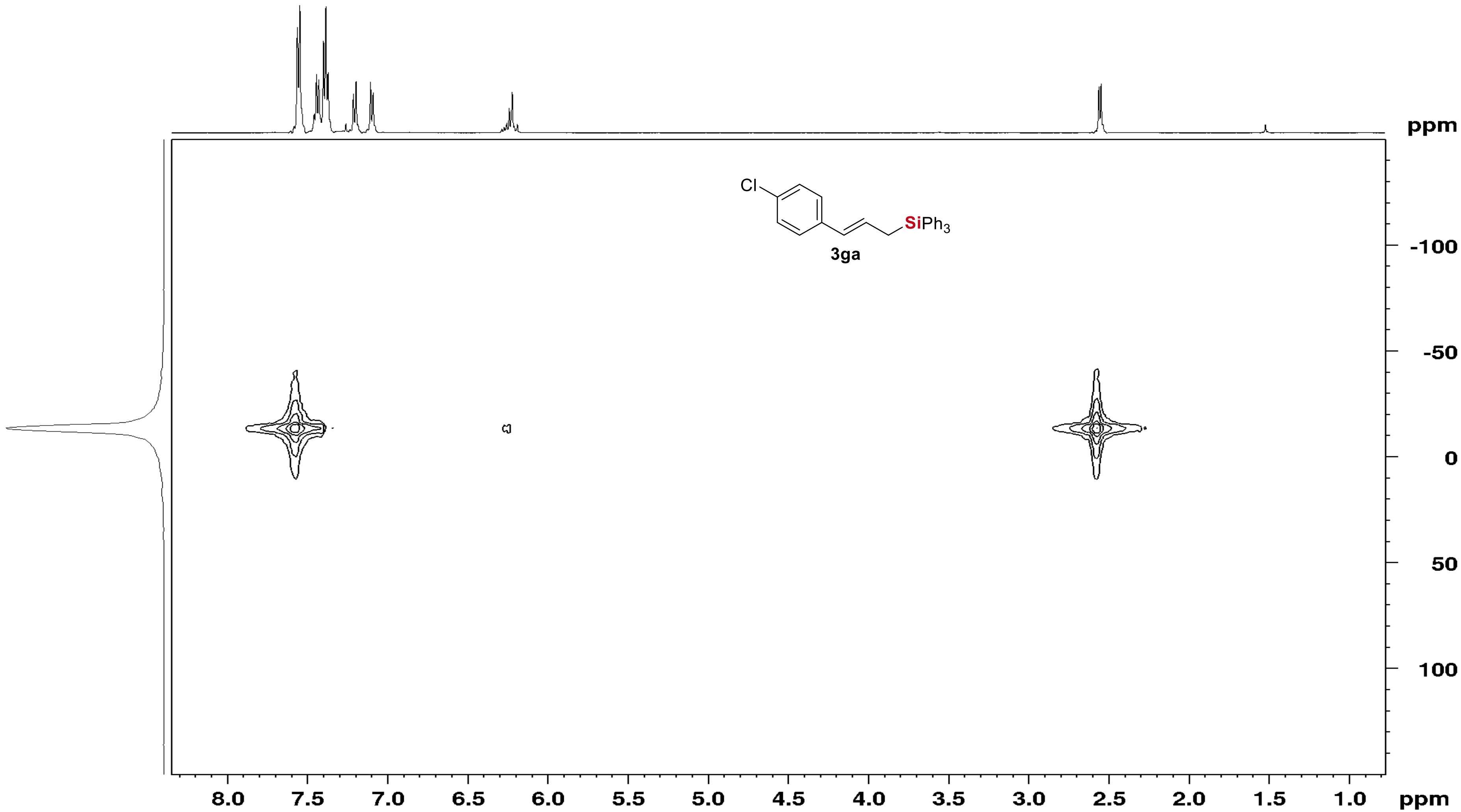

Figure S47. ^{1}H **NMR spectrum (500 MHz, $CDCl_3$, 298 K) of (*E*)-(3-(4-bromophenyl)allyl)triphenylsilane (3ha)**

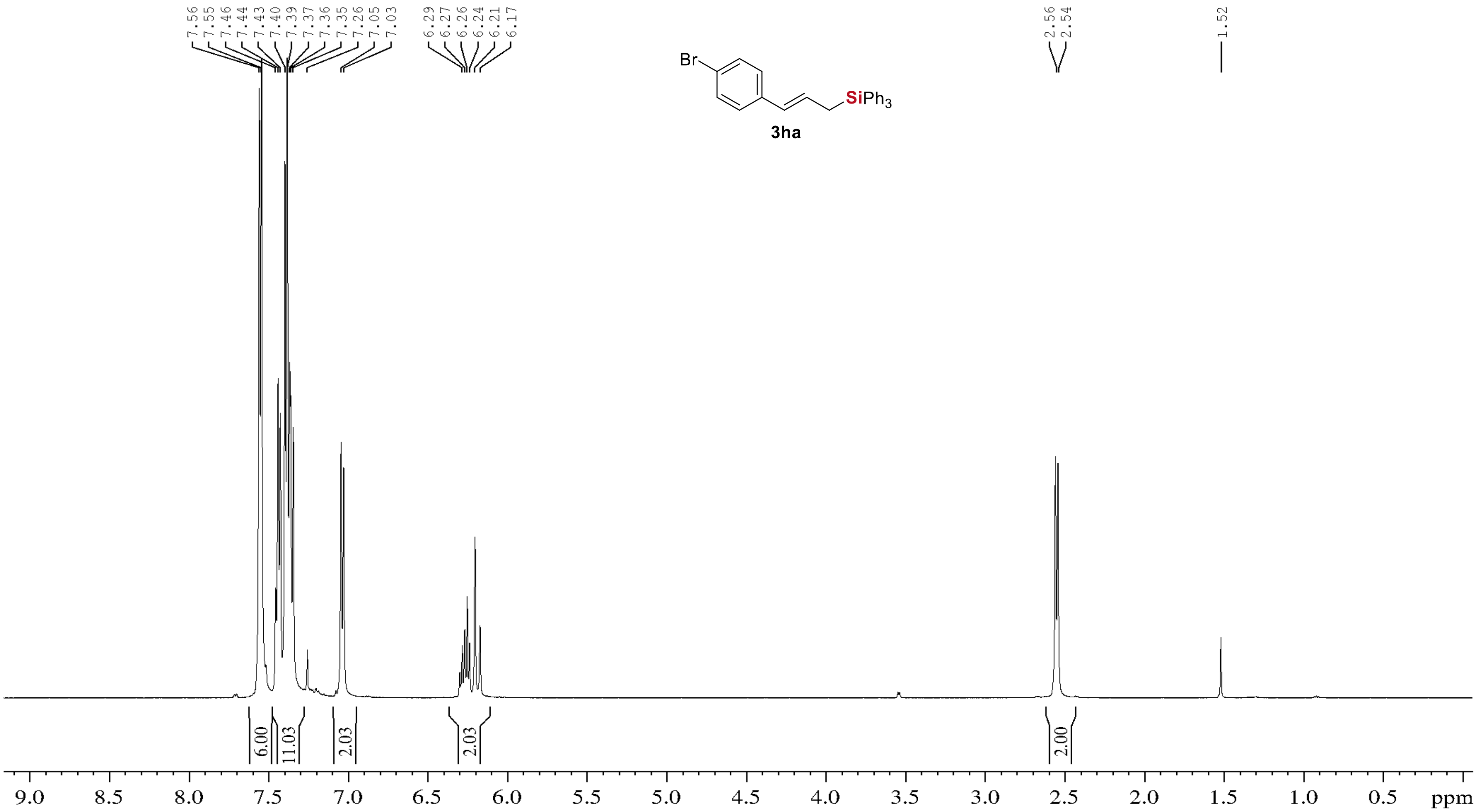

Figure S48. **$^{13}C\{^1H\}$ NMR spectrum (126 MHz, $CDCl_3$, 298 K) of (*E*)-(3-(4-bromophenyl)allyl)triphenylsilane (3ha)**

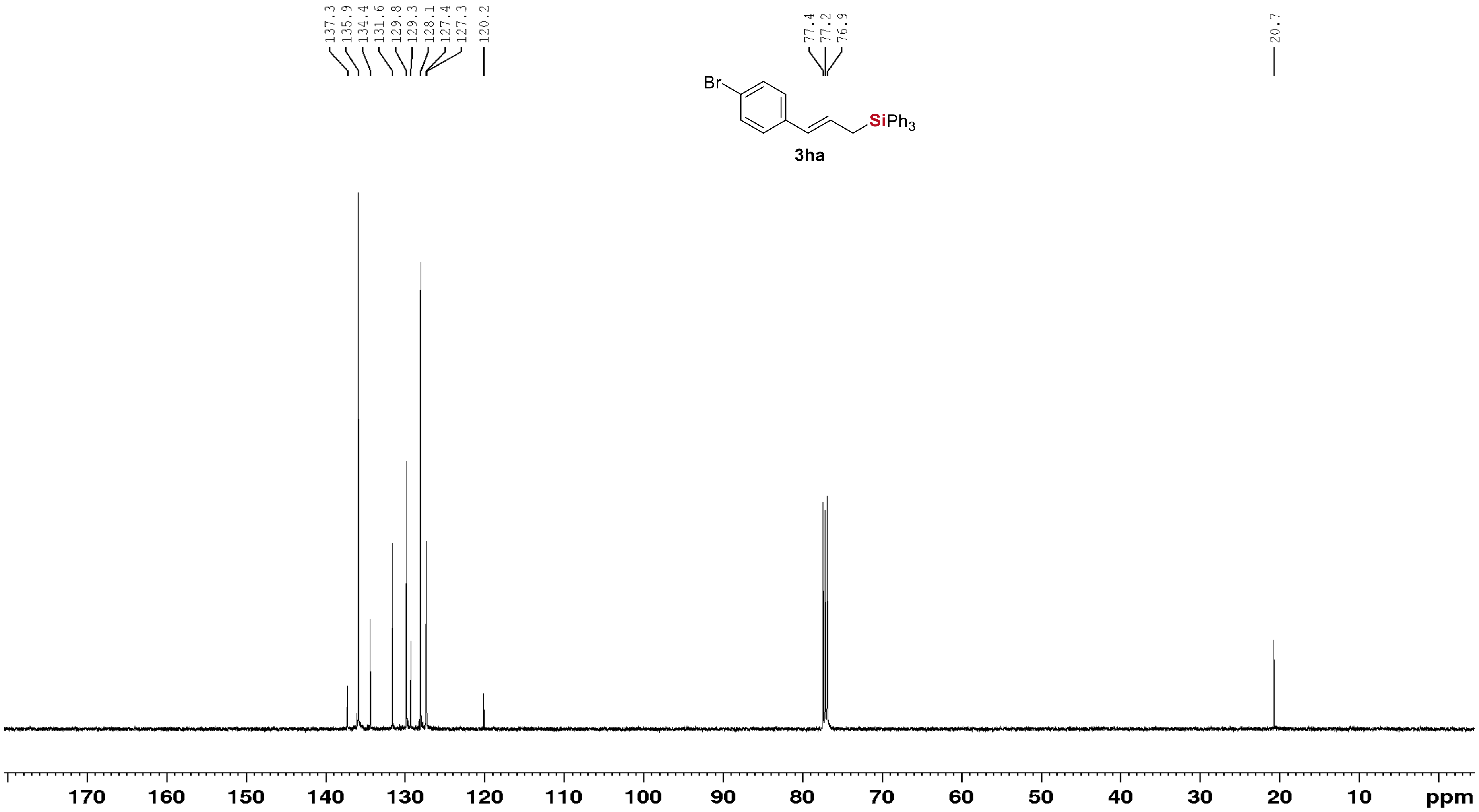

Figure S49. **$^1H/^{29}Si$ HMQC NMR spectrum (500/99 MHz, $CDCl_3$, 298 K, optimized for *J* = 7 Hz) of 3ha**

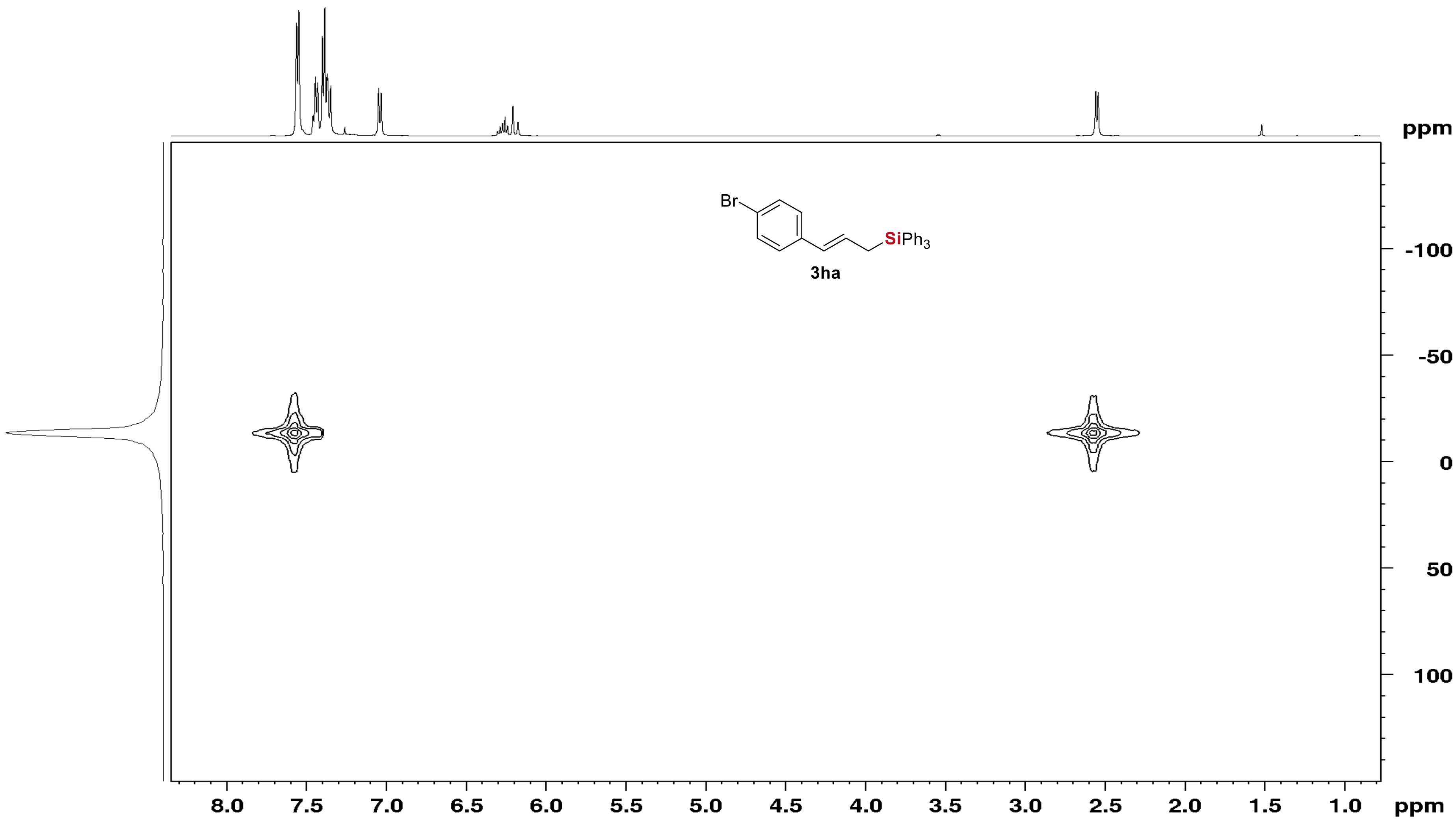

Figure S50. ¹H NMR spectrum (500 MHz, $CDCl_3$, 298 K) of (*E*)-triphenyl(3-(*m*-tolyl)allyl)silane (3ia)

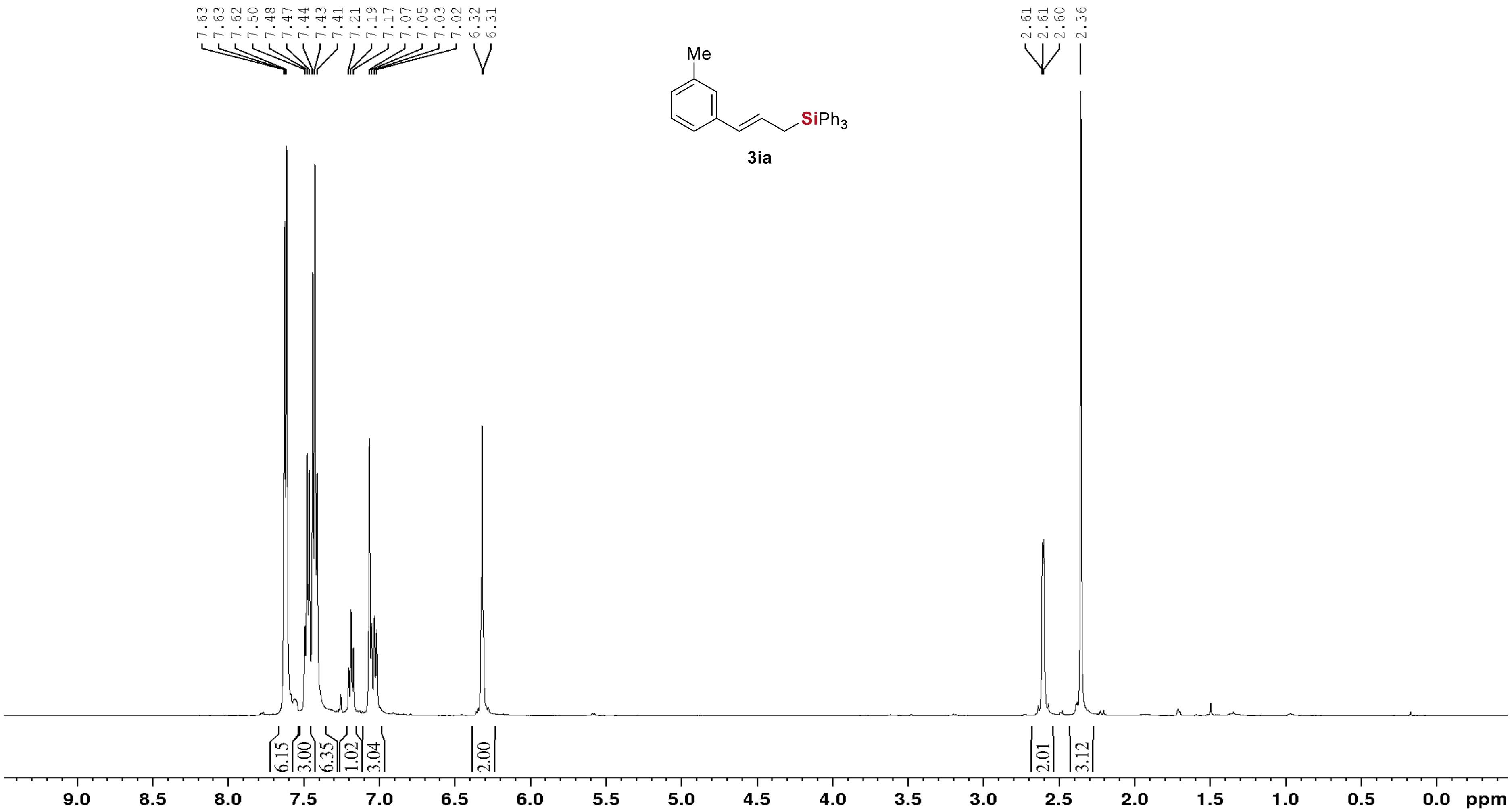

Figure S51. $^{13}C\{^1H\}$ NMR spectrum (126 MHz, $CDCl_3$, 298 K) of (*E*)-triphenyl(3-(*m*-tolyl)allyl)silane (3ia)

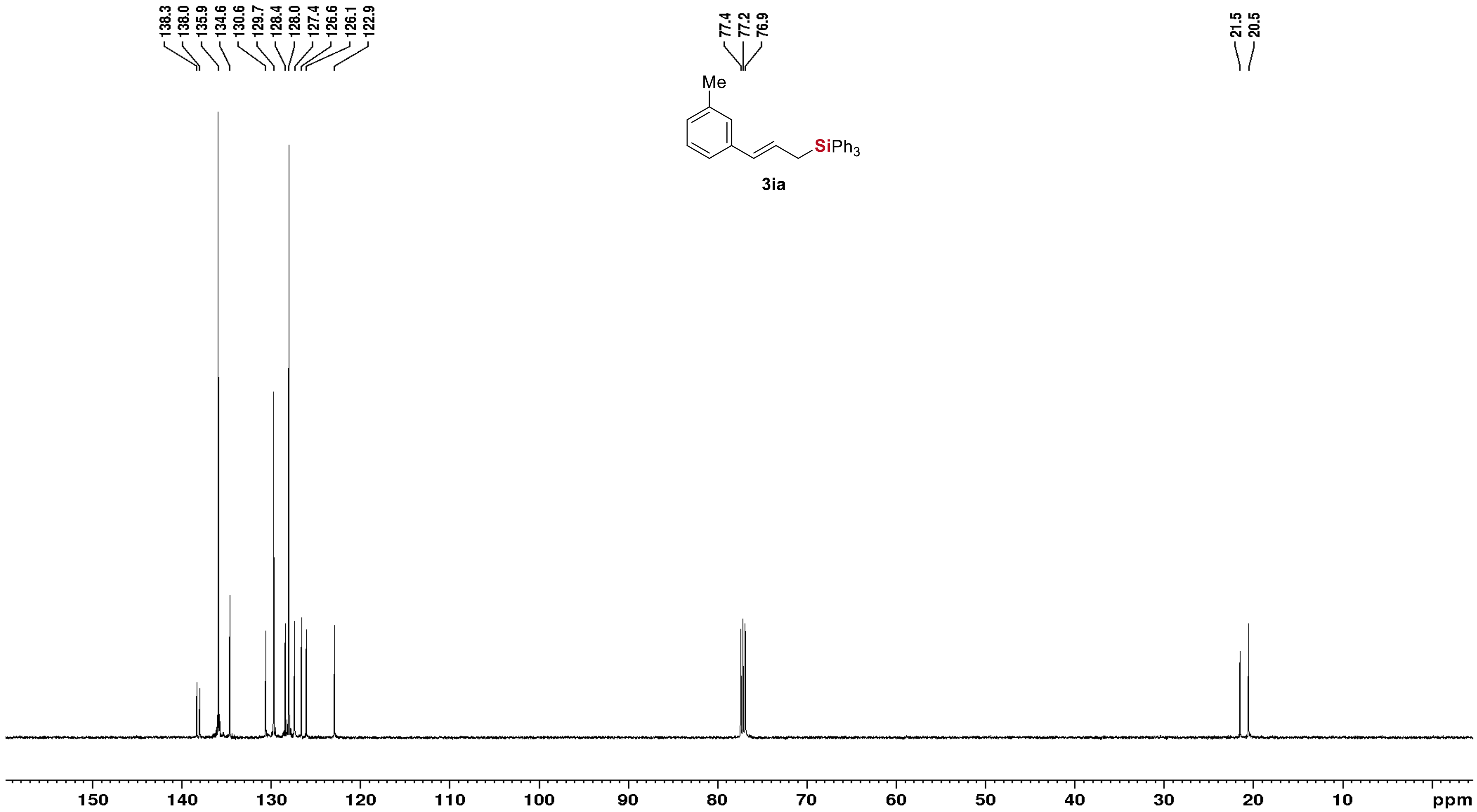

Figure S52. $^1H/^{29}Si$ HMQC NMR spectrum (500/99 MHz, $CDCl_3$, 298 K, optimized for J = 7 Hz) of 3ia

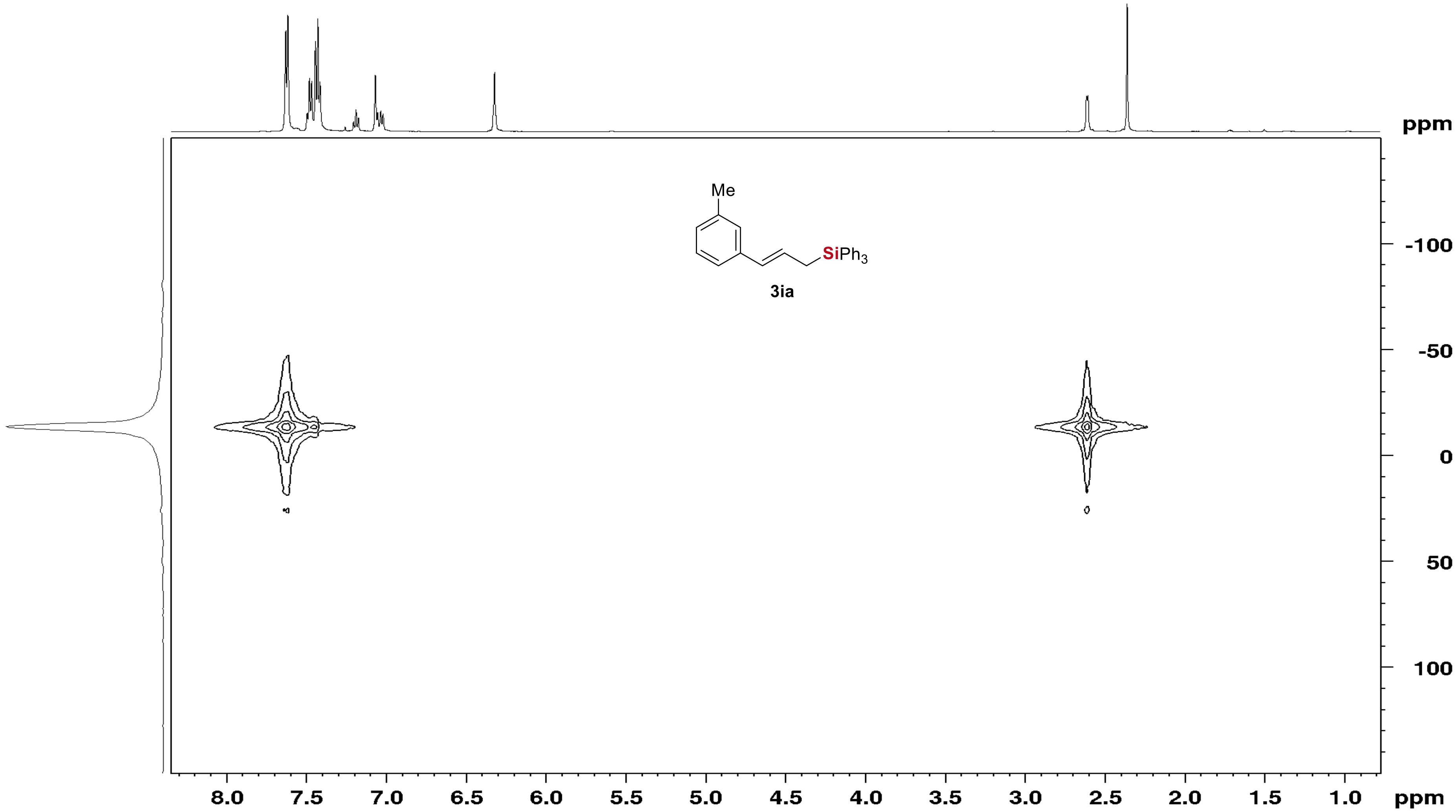

Figure S53. ^{1}H NMR spectrum (500 MHz, $CDCl_3$, 298 K) of (*E*)-(3-(3-fluorophenyl)allyl)triphenylsilane (3ja)

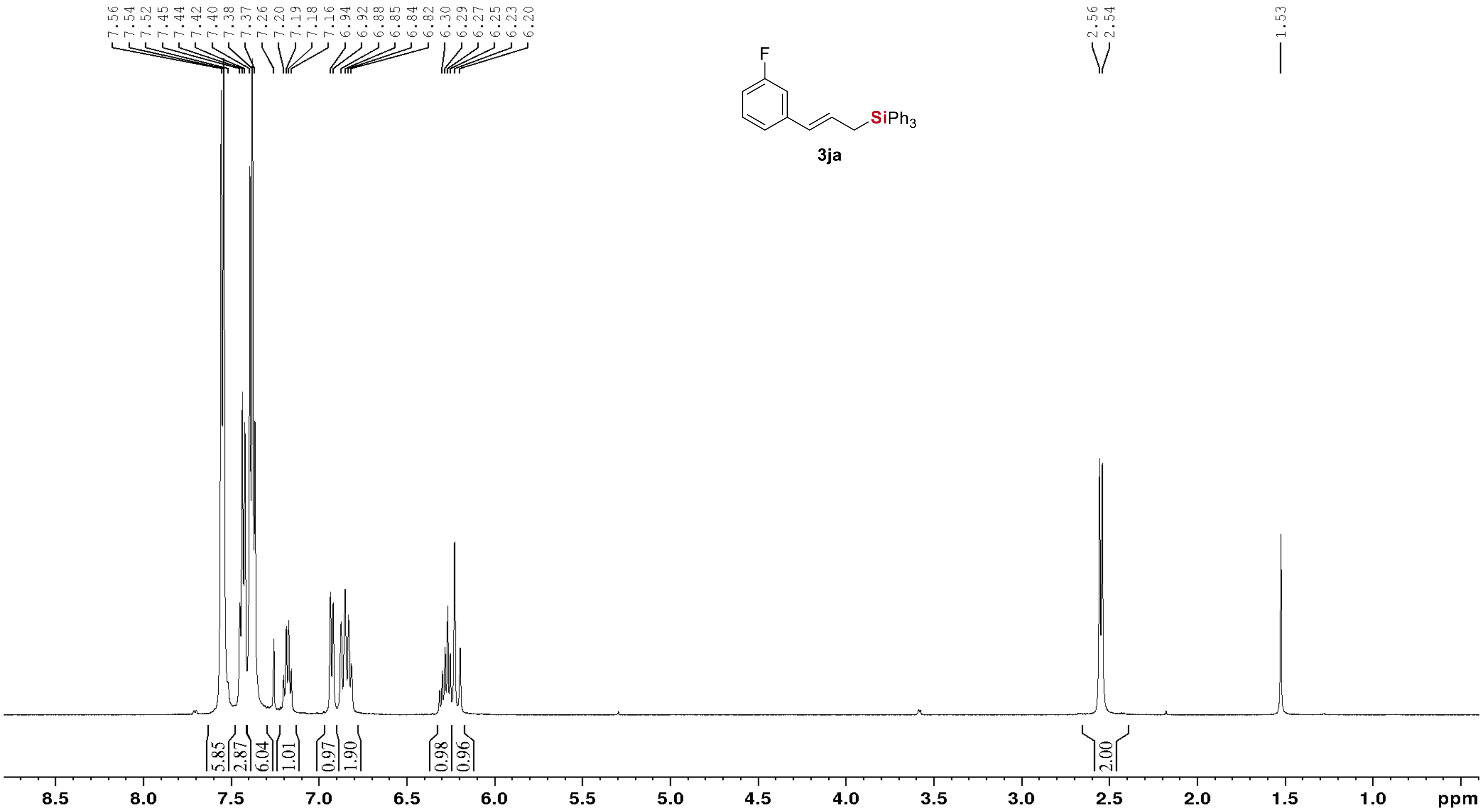

Figure S54. ^{19}F NMR (471 MHz, $CDCl_3$, 298 K) of (*E*)-(3-(3-fluorophenyl)allyl)triphenylsilane (3ja)

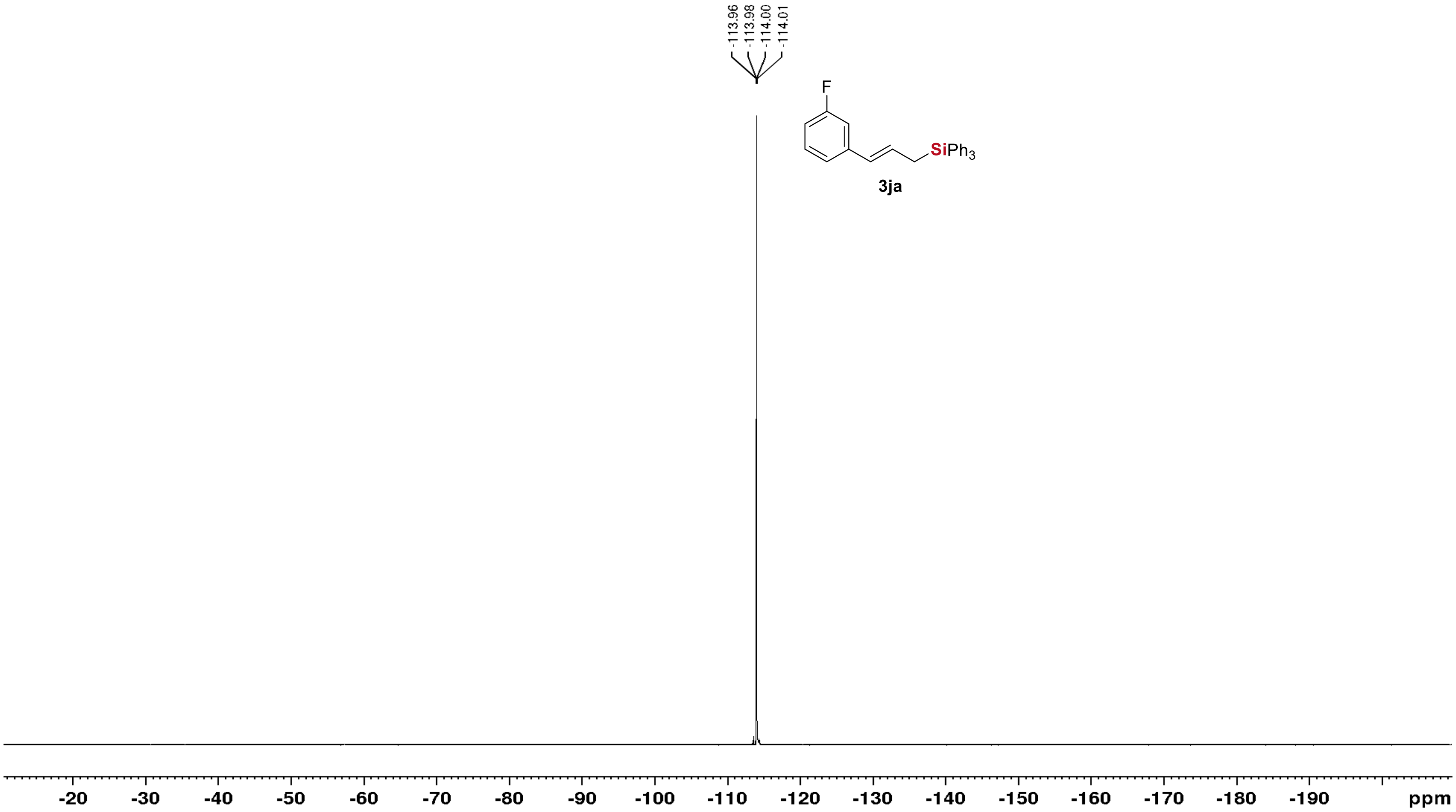

Figure S55. $^{13}C\{^{1}H\}$ **NMR spectrum (126 MHz,** $CDCl_3$**, 298 K) of (*E*)-(3-(3-fluorophenyl)allyl)triphenylsilane (3ja)**

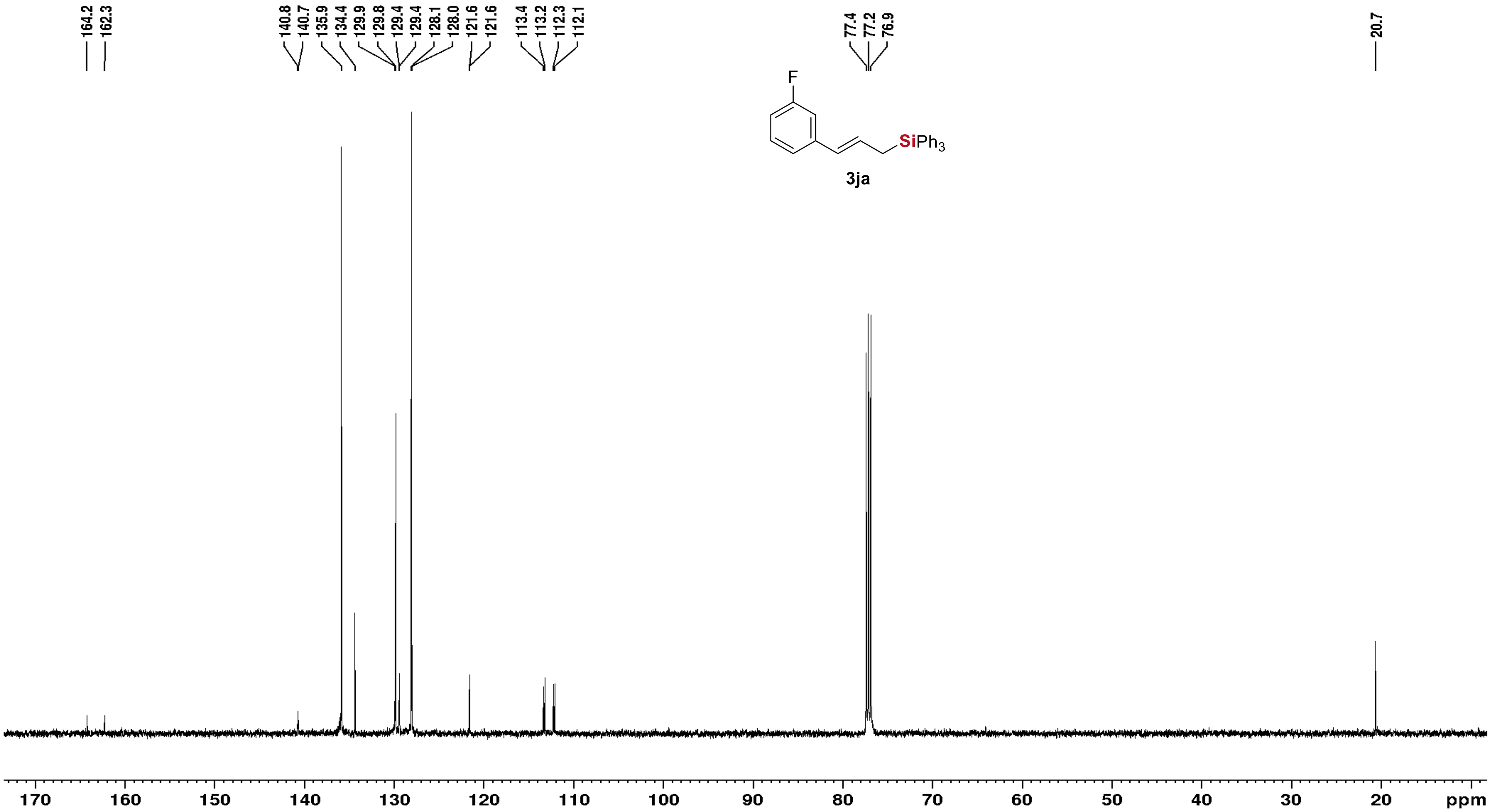

Figure S56. **$^{1}H/^{29}Si$ HMQC NMR spectrum (500/99 MHz, $CDCl_3$, 298 K, optimized for *J* = 7 Hz) of 3ja**

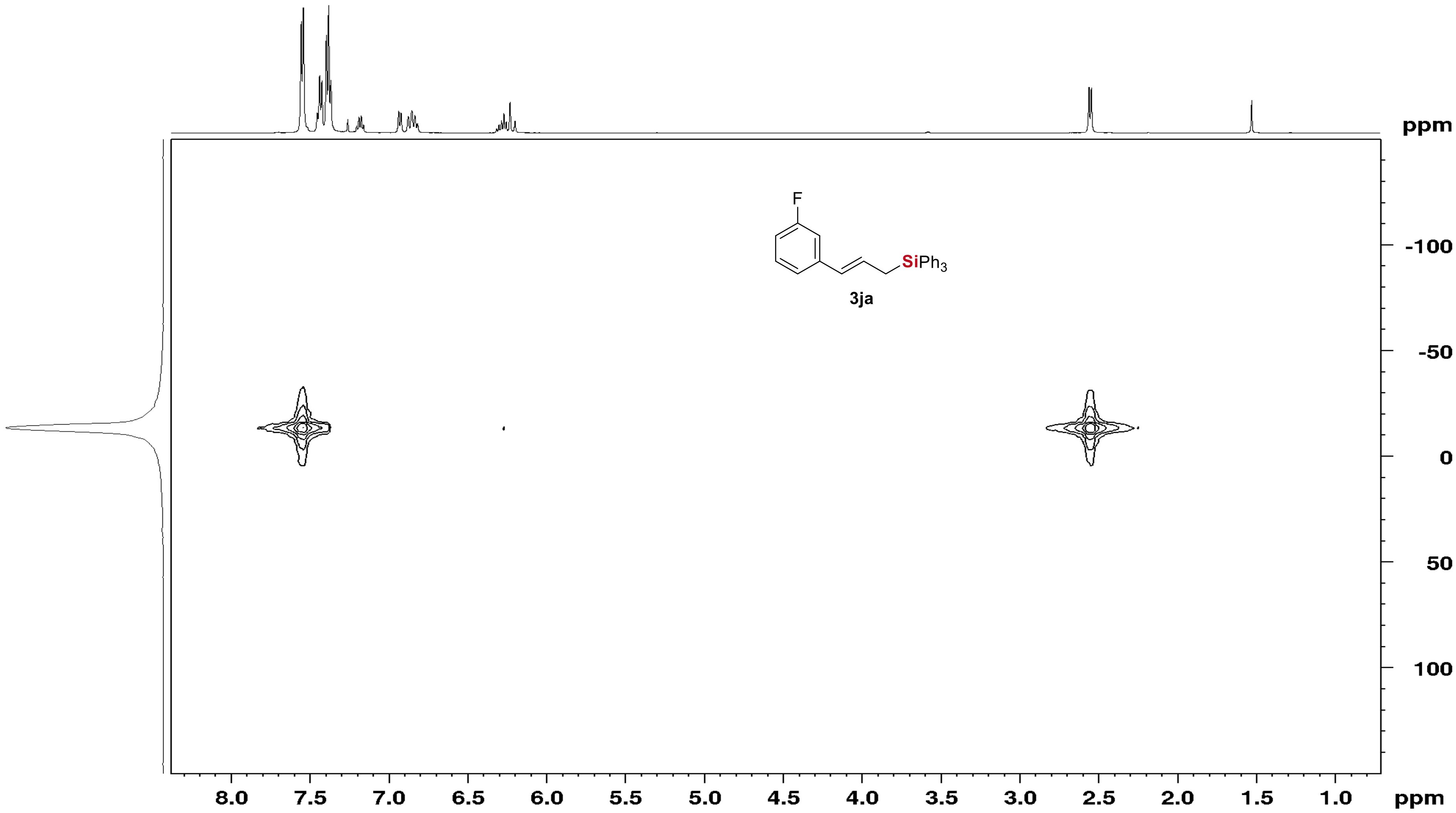

Figure S57. ^{1}H NMR spectrum (500 MHz, $CDCl_3$, 298 K) of (*E*)-(3-(3-chlorophenyl)allyl)triphenylsilane (3ka)

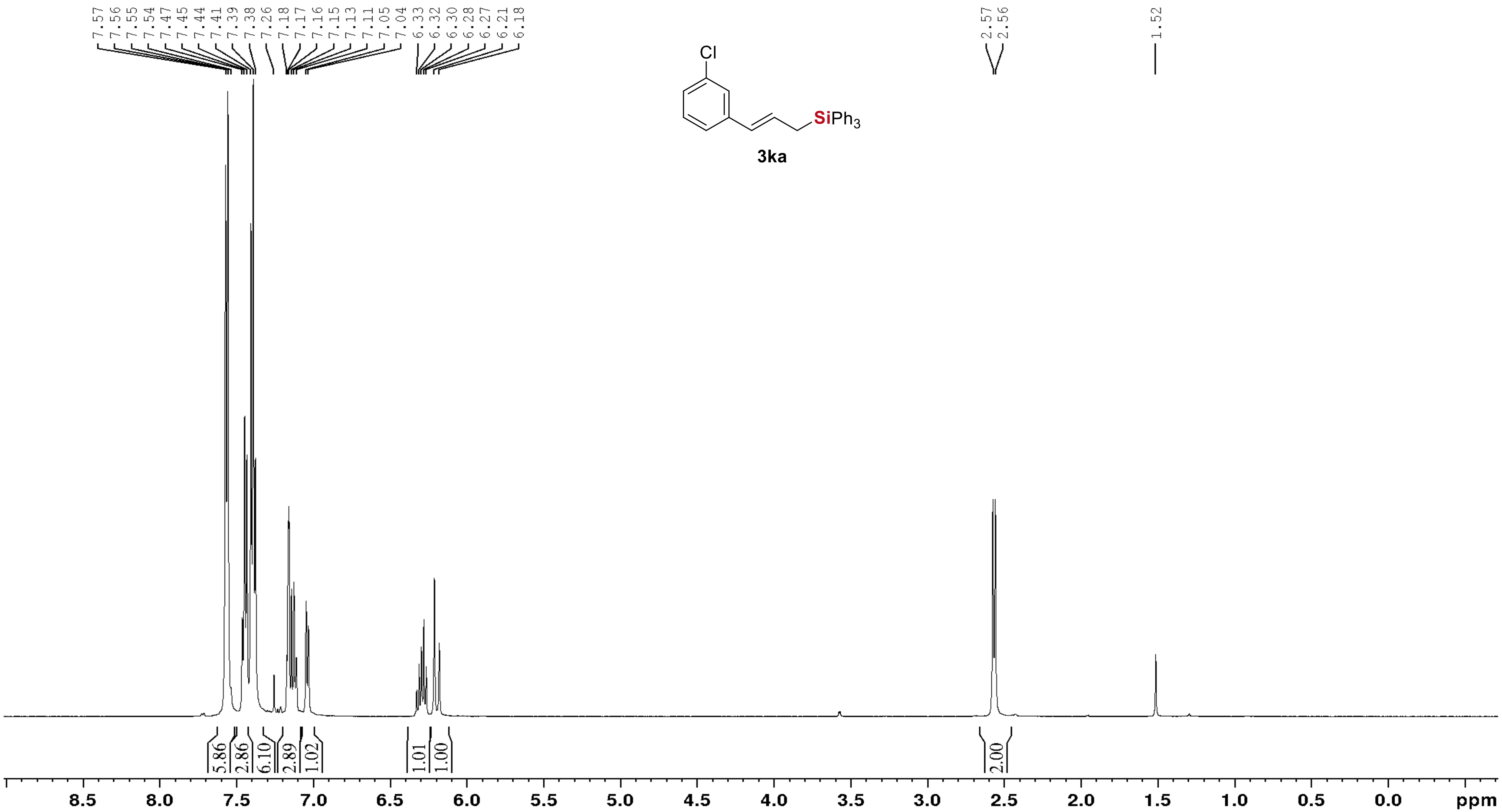

Figure S58. $^{13}C\{^1H\}$ **NMR spectrum (126 MHz, $CDCl_3$, 298 K) of (*E*)-(3-(3-chlorophenyl)allyl)triphenylsilane (3ka)**

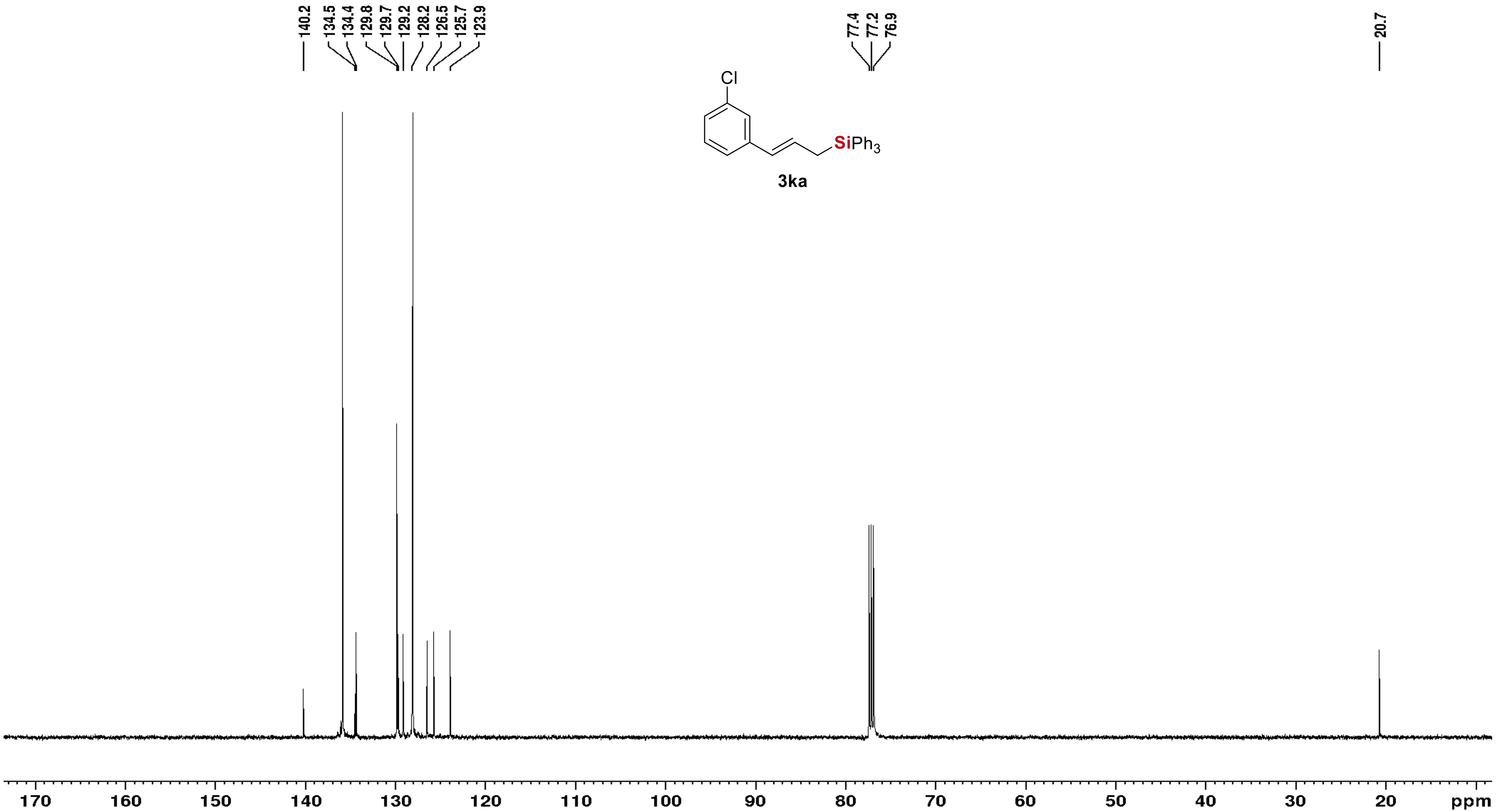

Figure S59. $^{1}H/^{29}Si$ HMQC NMR spectrum (500/99 MHz, $CDCl_3$, 298 K, optimized for *J* = 7 Hz) of 3ka

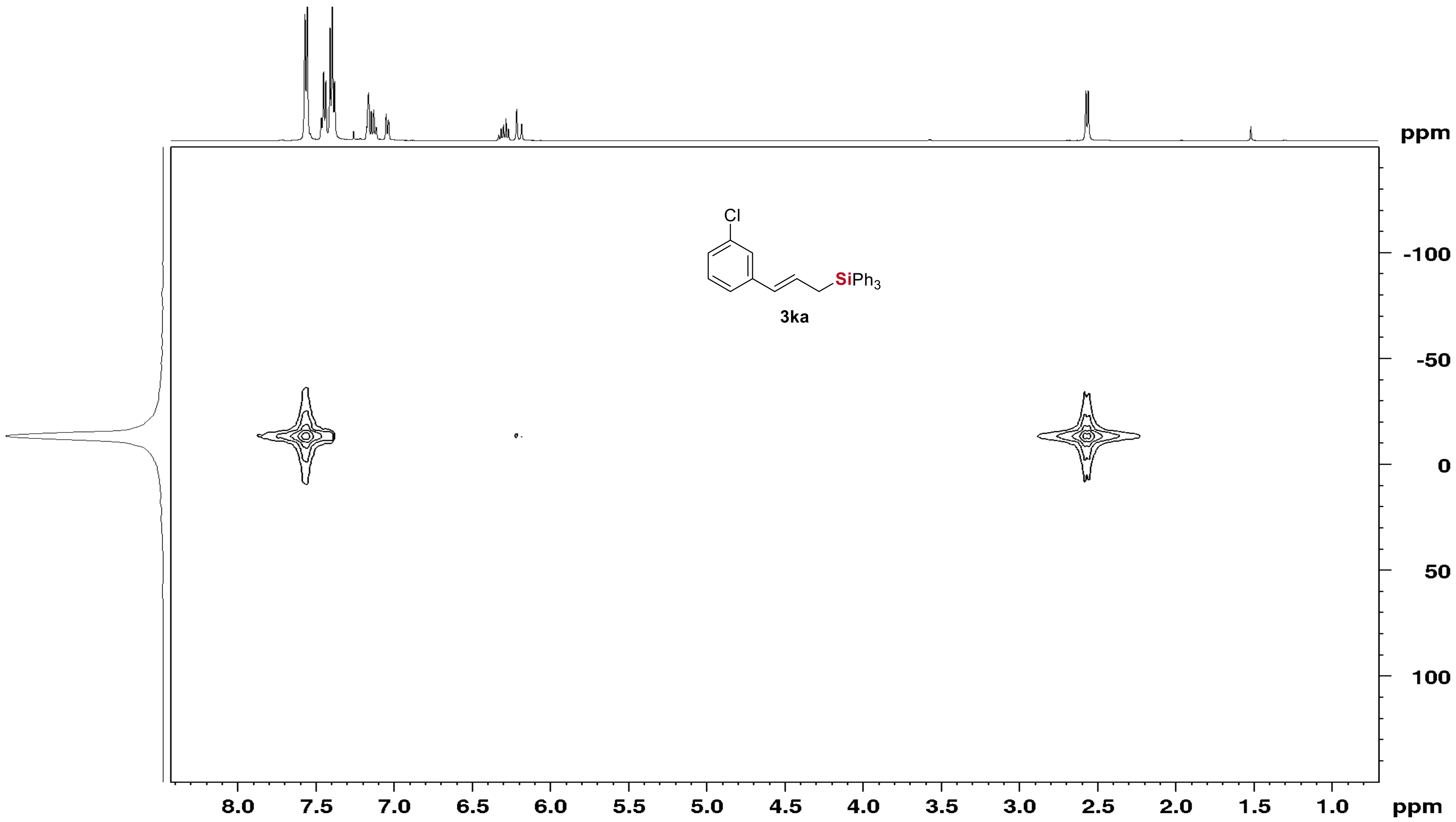

Figure S60. ^{1}H NMR spectrum (500 MHz, $CDCl_3$, 298 K) of (*E*)-triphenyl(3-(*o*-tolyl)allyl)silane (3la)

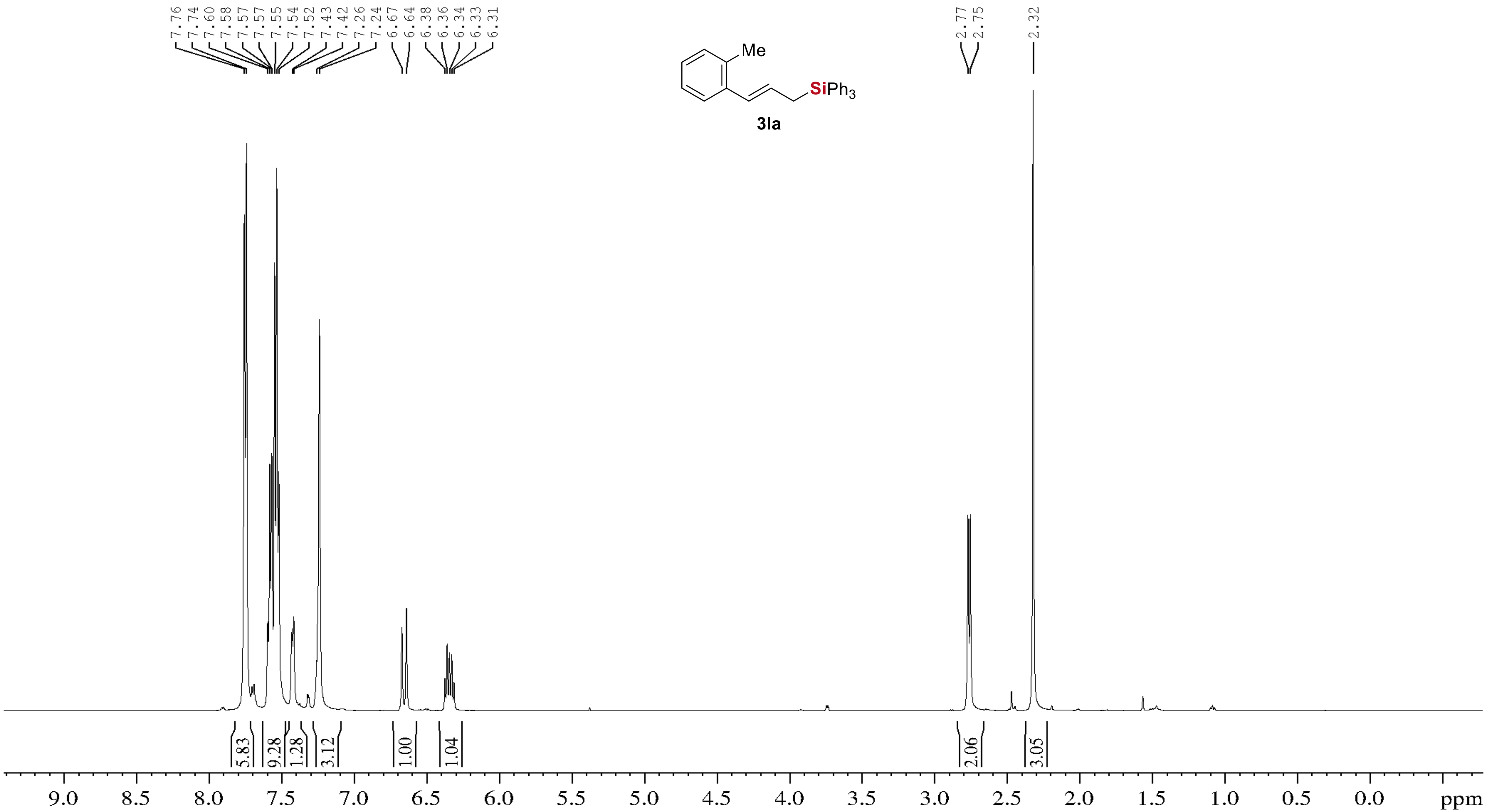

Figure S61. **$^{13}C\{^1H\}$ NMR spectrum (126 MHz, $CDCl_3$, 298 K) of (*E*)-triphenyl(3-(*o*-tolyl)allyl)silane (3la)**

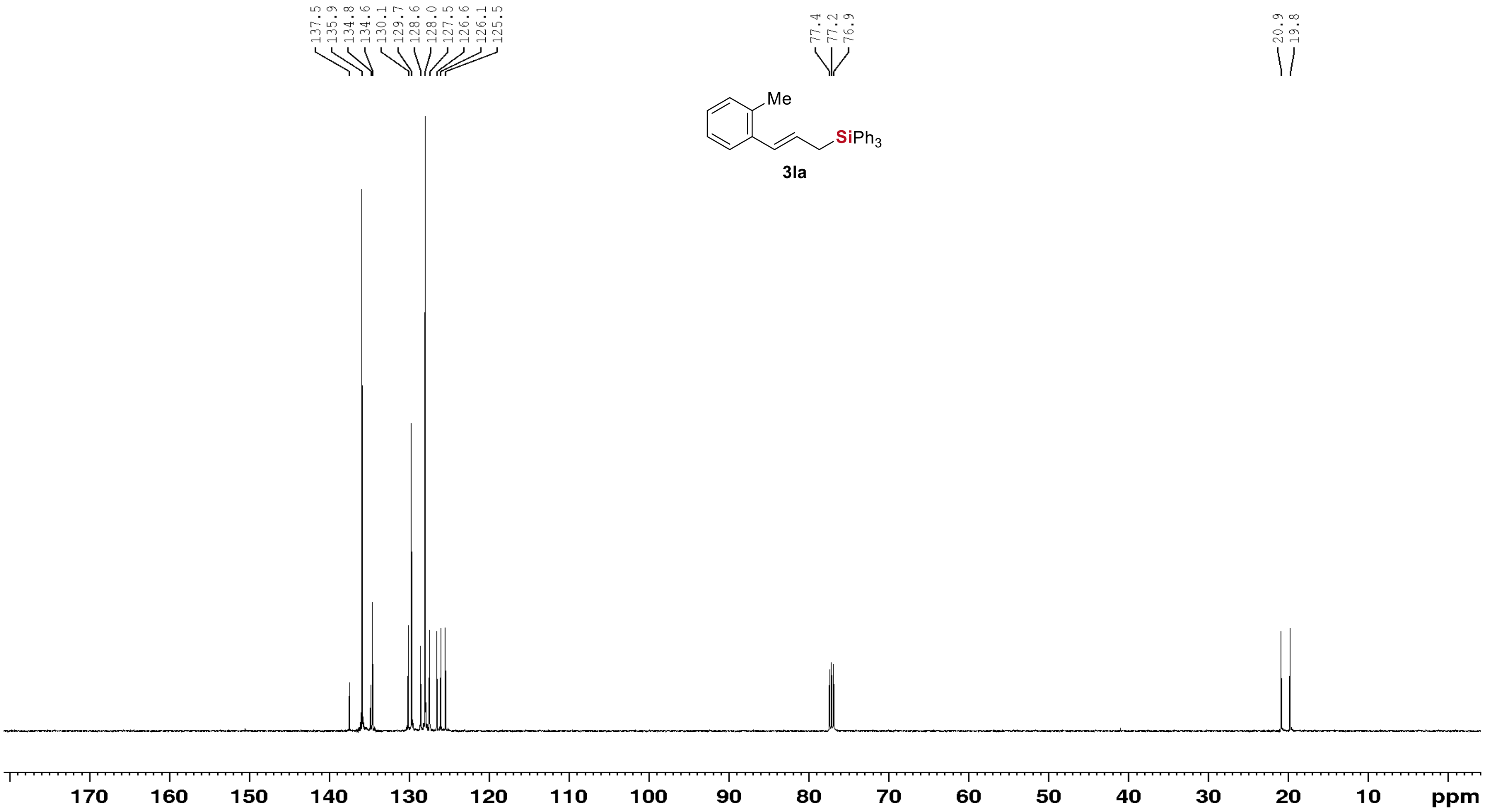

Figure S62. $^{1}H/^{29}Si$ HMQC NMR spectrum (500/99 MHz, $CDCl_3$, 298 K, optimized for *J* = 7 Hz) of 3Ia

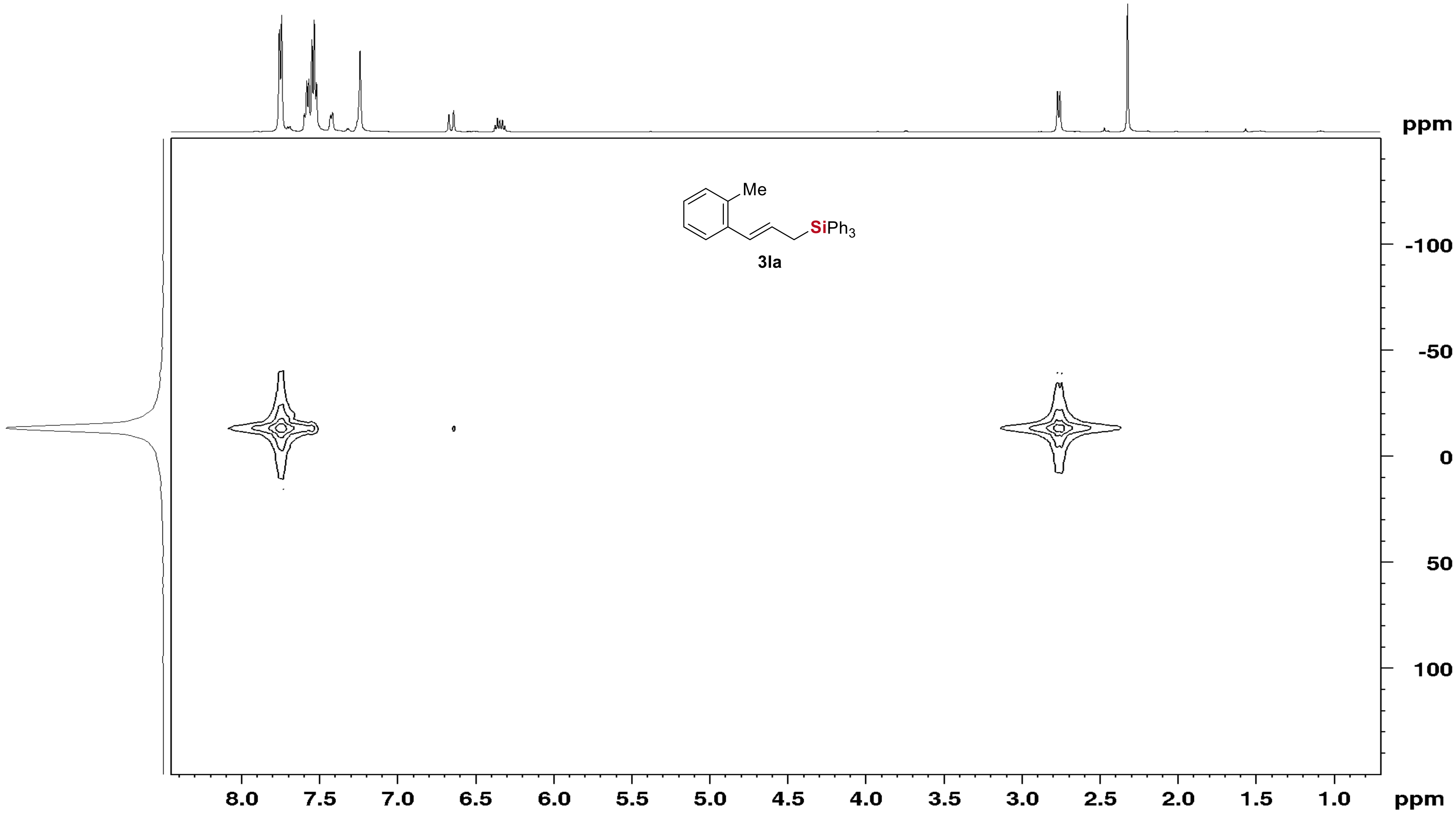

Figure S63. **^{1}H NMR spectrum (500 MHz, CDCl$_3$, 298 K) of (*E*)-triphenyl(2-(*florophenyl*)allyl)silane (3ma)**

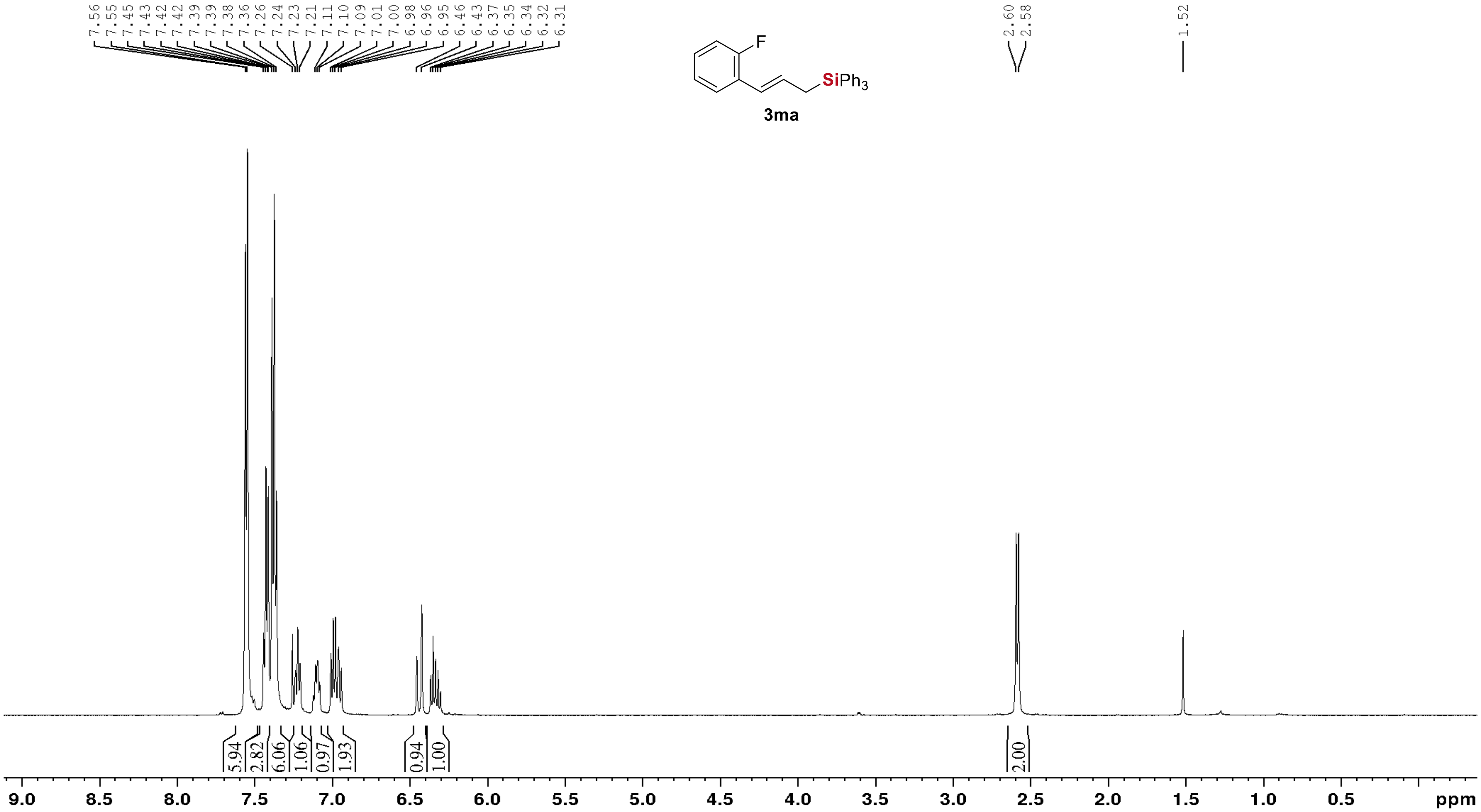

3ma

Figure S64. ^{19}F NMR (471 MHz, $CDCl_3$, 298 K) of (*E*)-triphenyl(2-(*florophenyl*)allyl)silane (3ma)

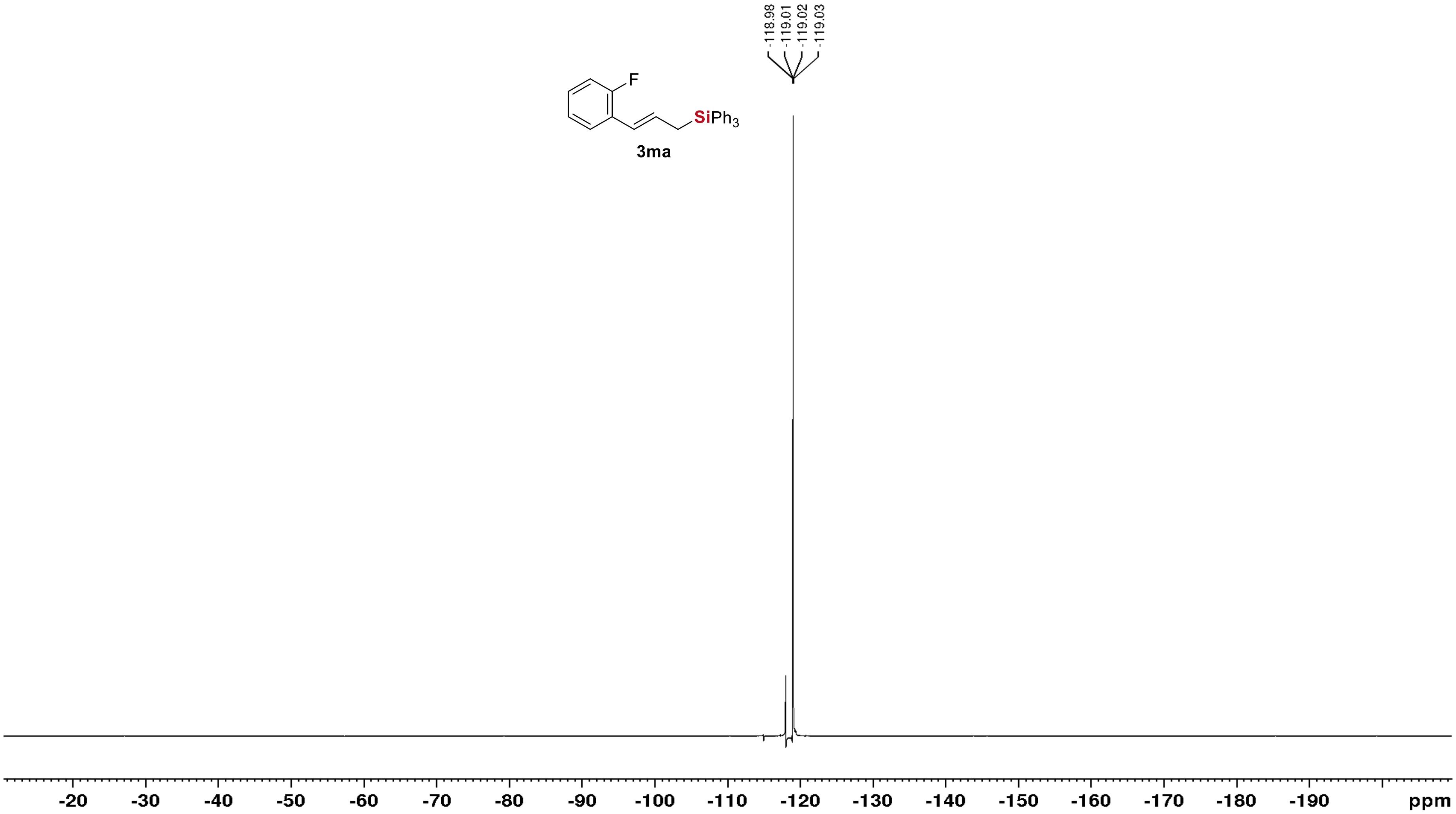

Figure S65. $^{13}C\{^1H\}$ **NMR spectrum (126 MHz, $CDCl_3$, 298 K) of *E*)-triphenyl(2-(*florophenyl*)allyl)silane (3ma)**

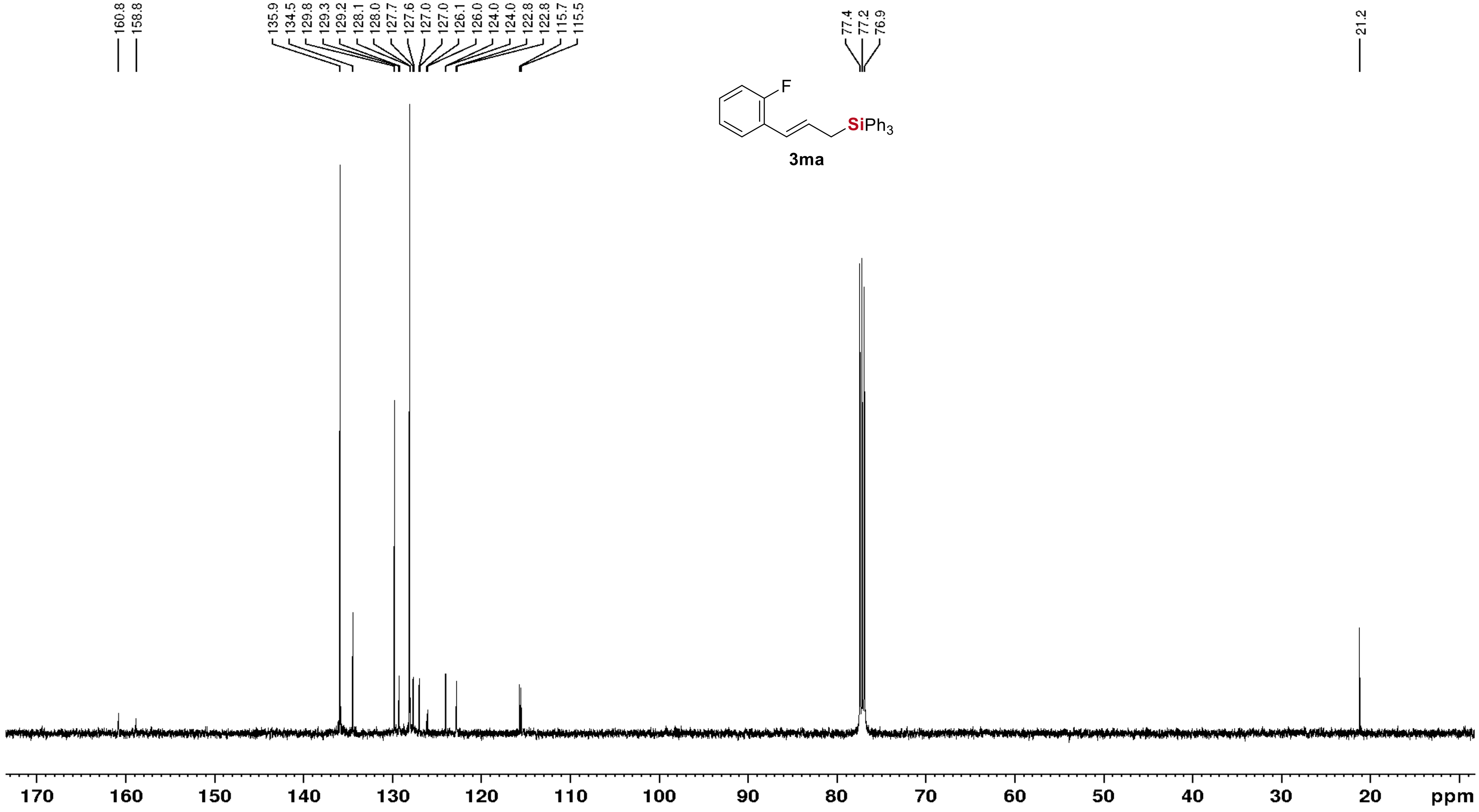

Figure S66. $^{1}H/^{29}Si$ HMQC NMR spectrum (500/99 MHz, $CDCl_3$, 298 K, optimized for J = 7 Hz) of 3ma

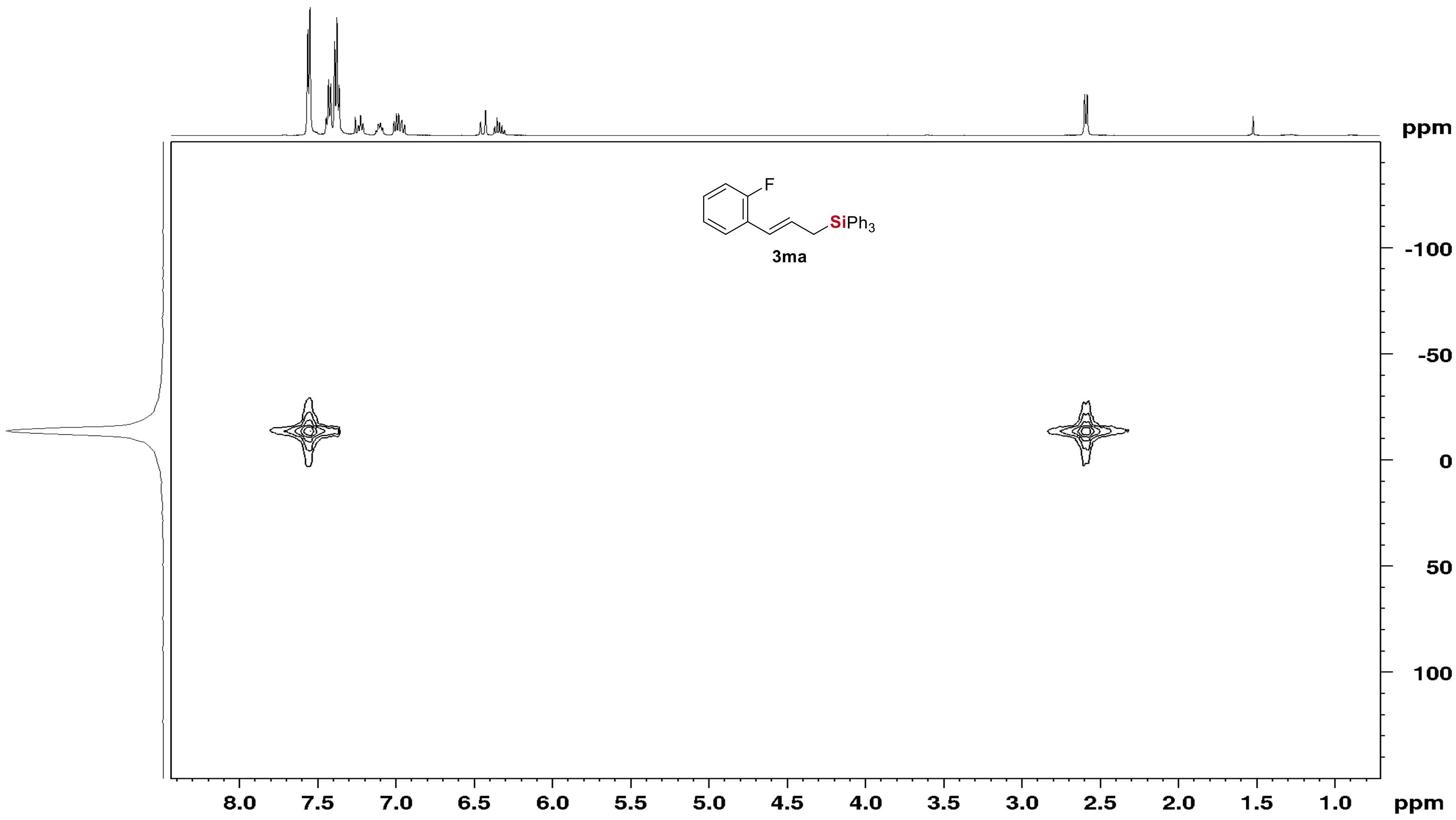

Figure S67. ^{1}H NMR spectrum (500 MHz, $CDCl_3$, 298 K) of (*E*)-(3-(2-bromophenyl)allyl)triphenylsilane (3na)

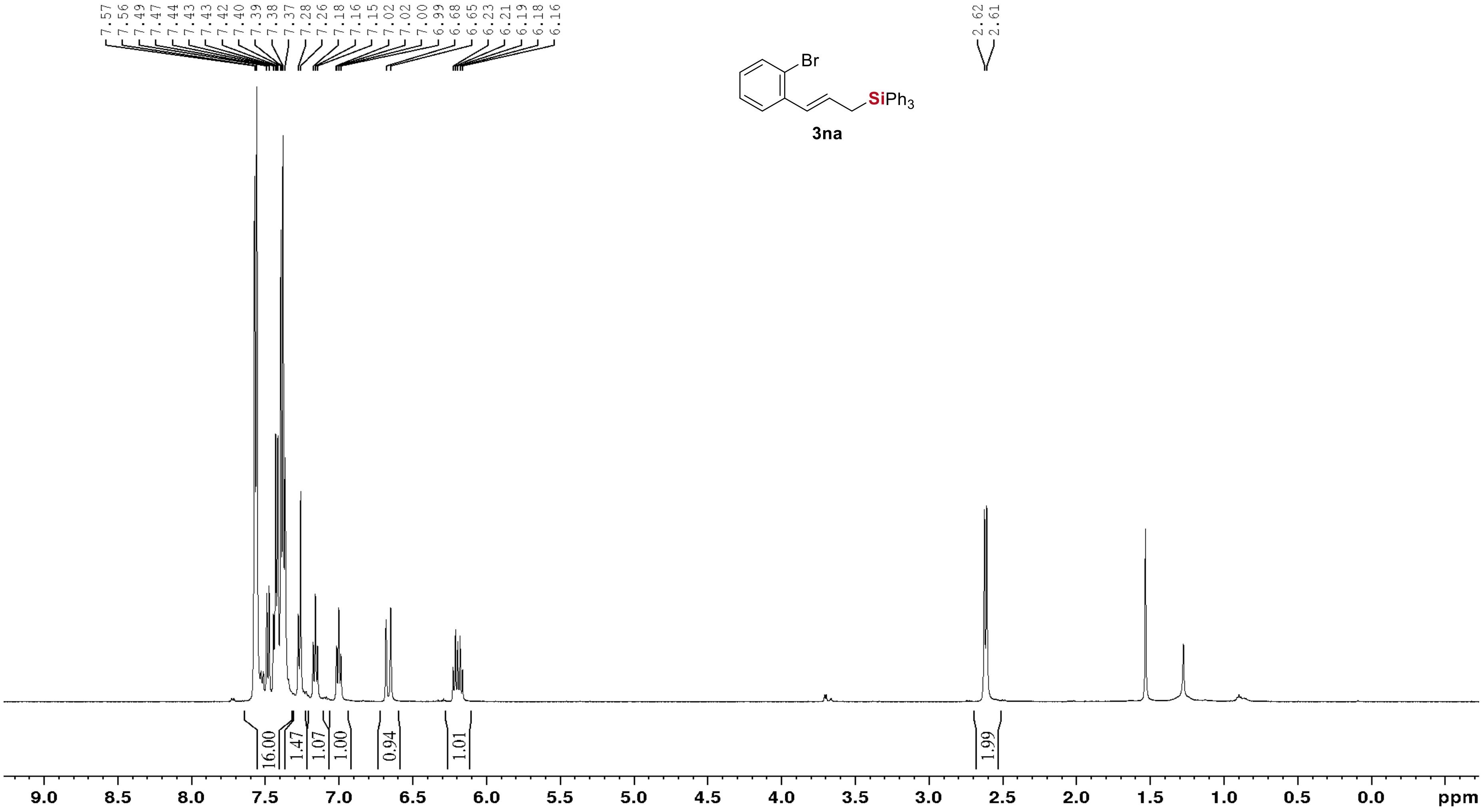

Figure S68. $^{13}C\{^1H\}$ **NMR spectrum (126 MHz, $CDCl_3$, 298 K) of (*E*)-(3-(2-bromophenyl)allyl)triphenylsilane (3na)**

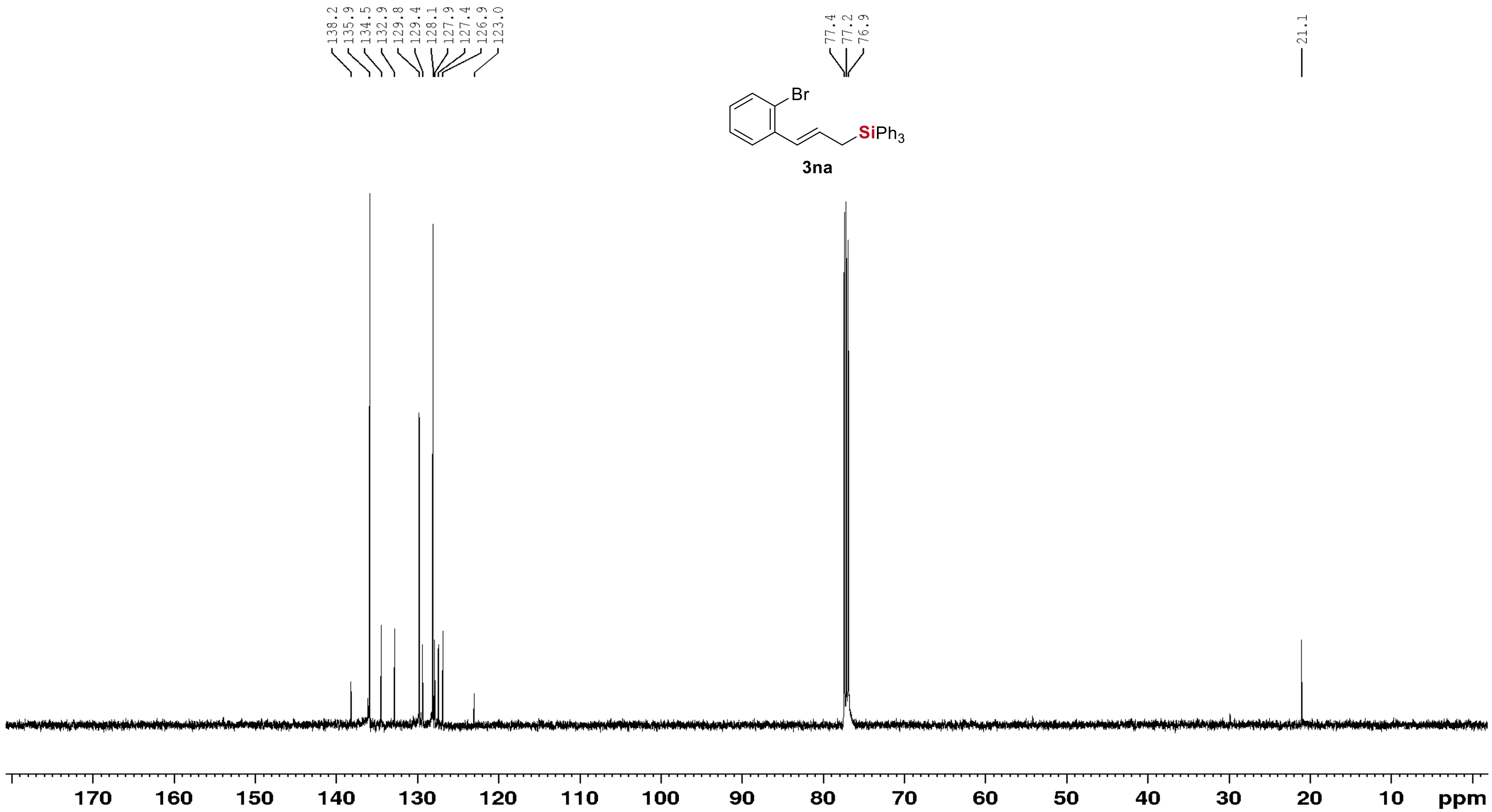

Figure S69. $^{1}H/^{29}Si$ HMQC NMR spectrum (500/99 MHz, $CDCl_3$, 298 K, optimized for J = 7 Hz) of 3na

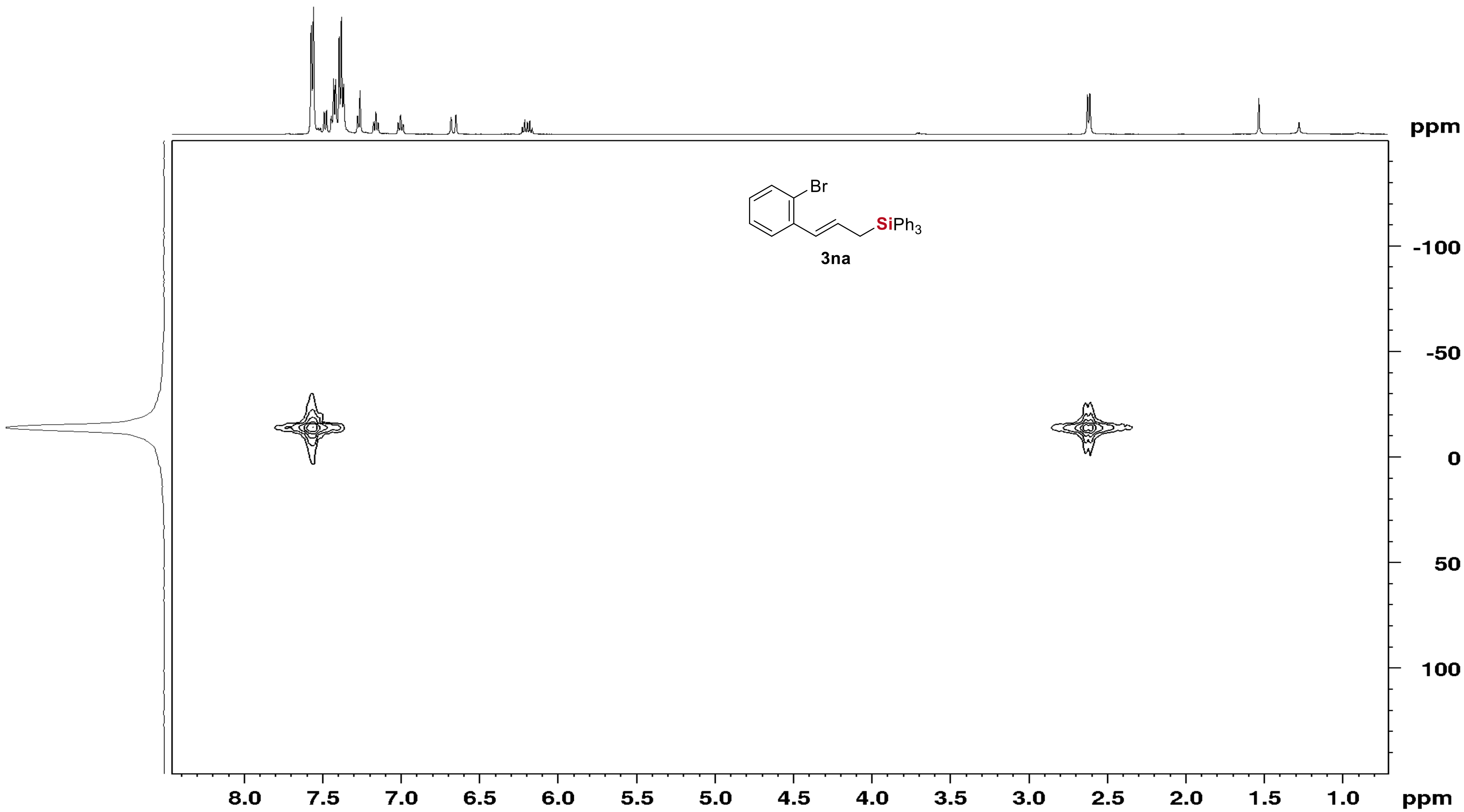

Figure S70. ¹H NMR spectrum (500 MHz, $CDCl_3$, 298 K) of (3oa + 4oa)

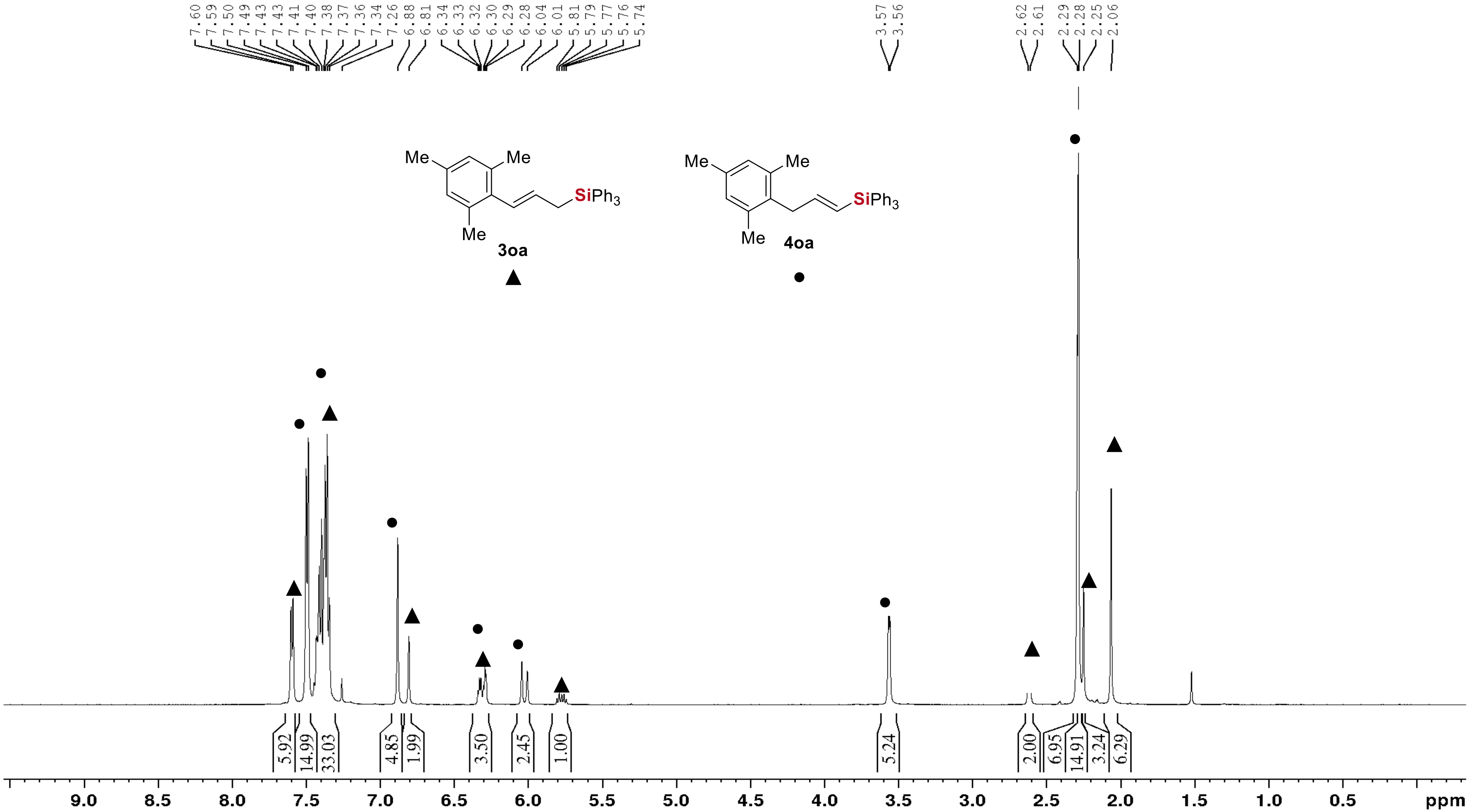

Figure S71. $^{1}H/^{29}Si$ **HMQC NMR spectrum (500/99 MHz, $CDCl_3$, 298 K, optimized for *J* = 7 Hz) of (3oa + 4oa)**

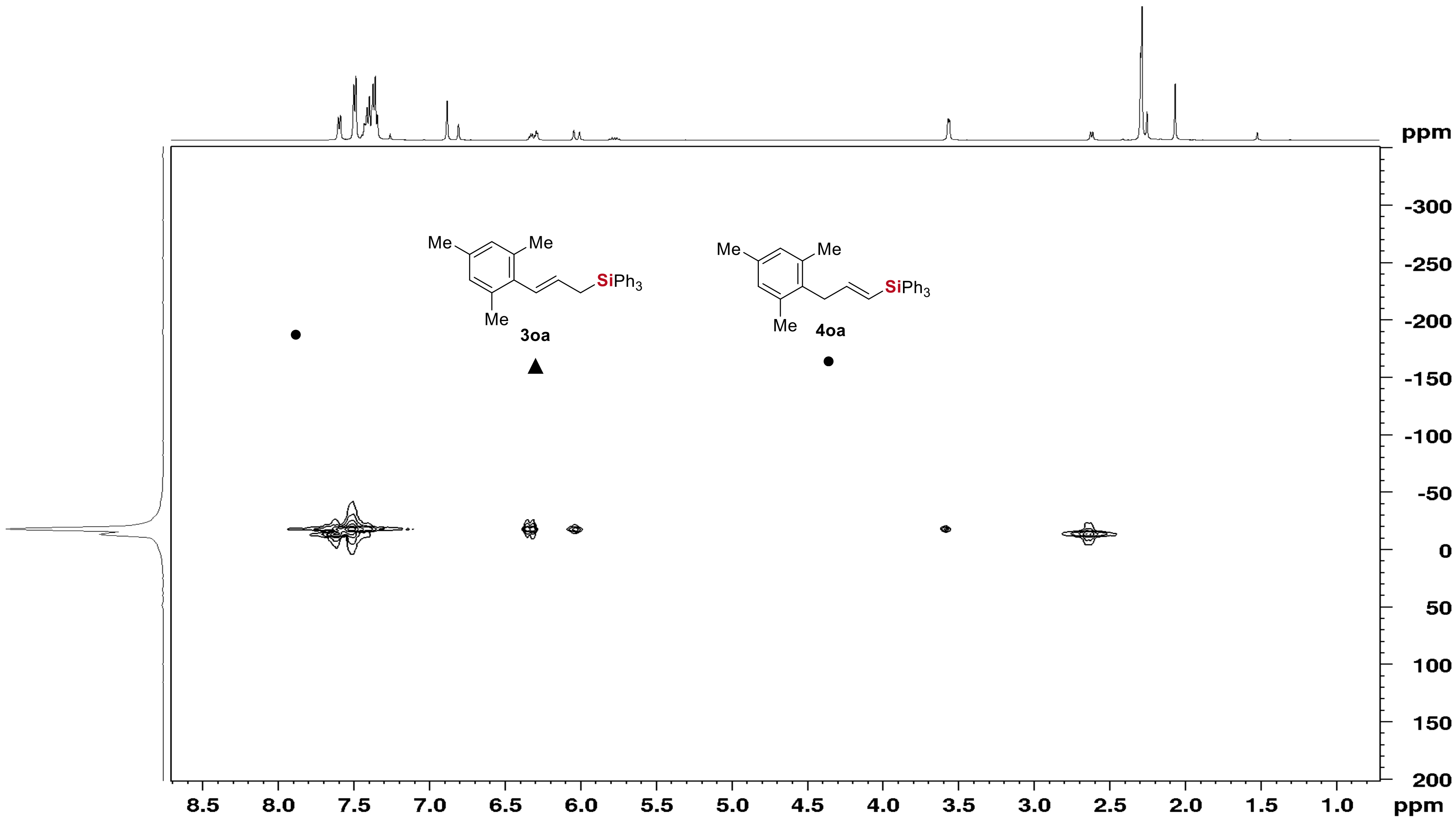

Figure S72. H-H COSY NMR spectrum (500 MHz, $CDCl_3$, 298 K) of (3oa + 4oa)

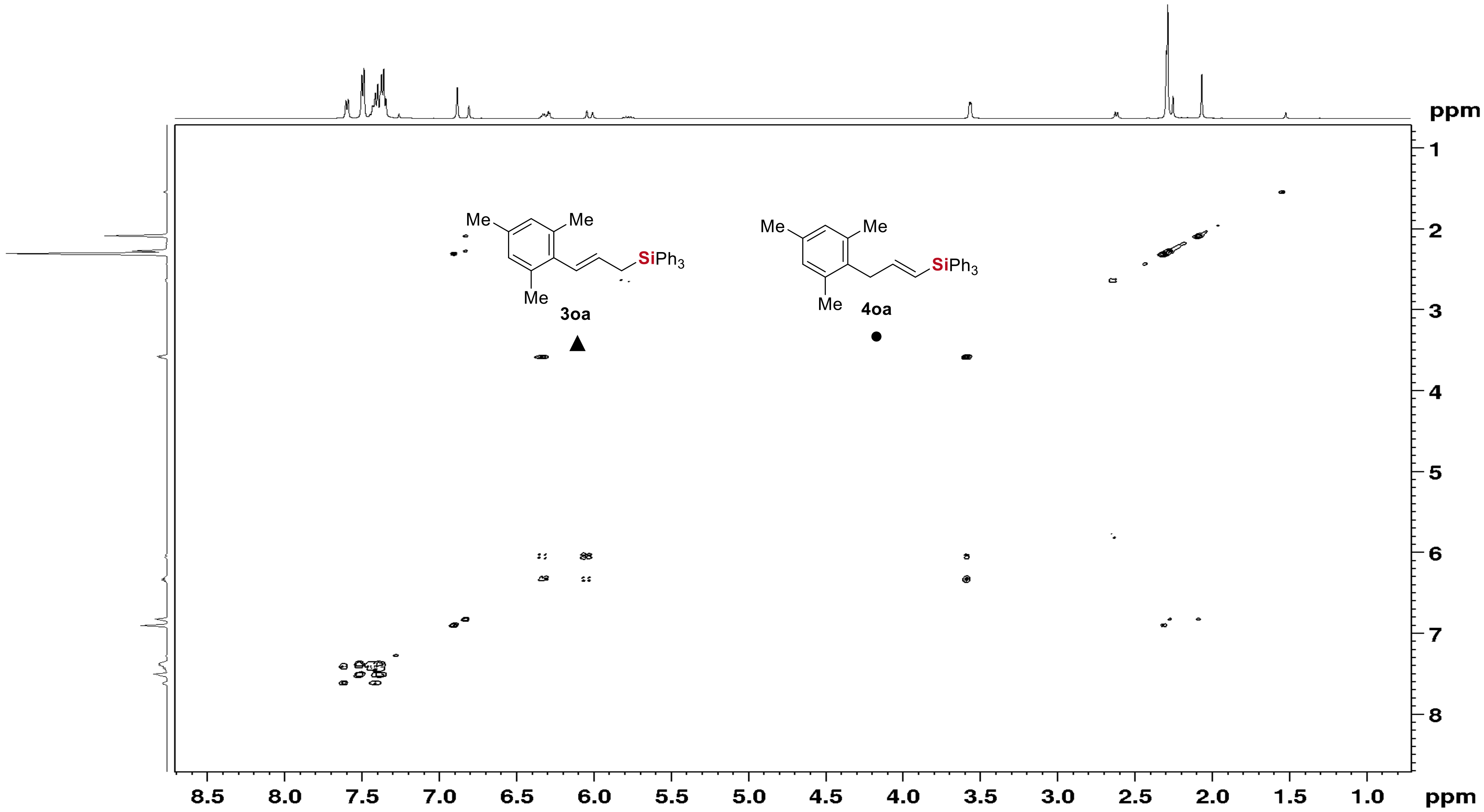

Figure S73. ¹H NMR spectrum (500 MHz, $CDCl_3$, 298 K) of (*E*)-(3-([1,1'-biphenyl]-4-yl)allyl)triphenylsilane (3pa)

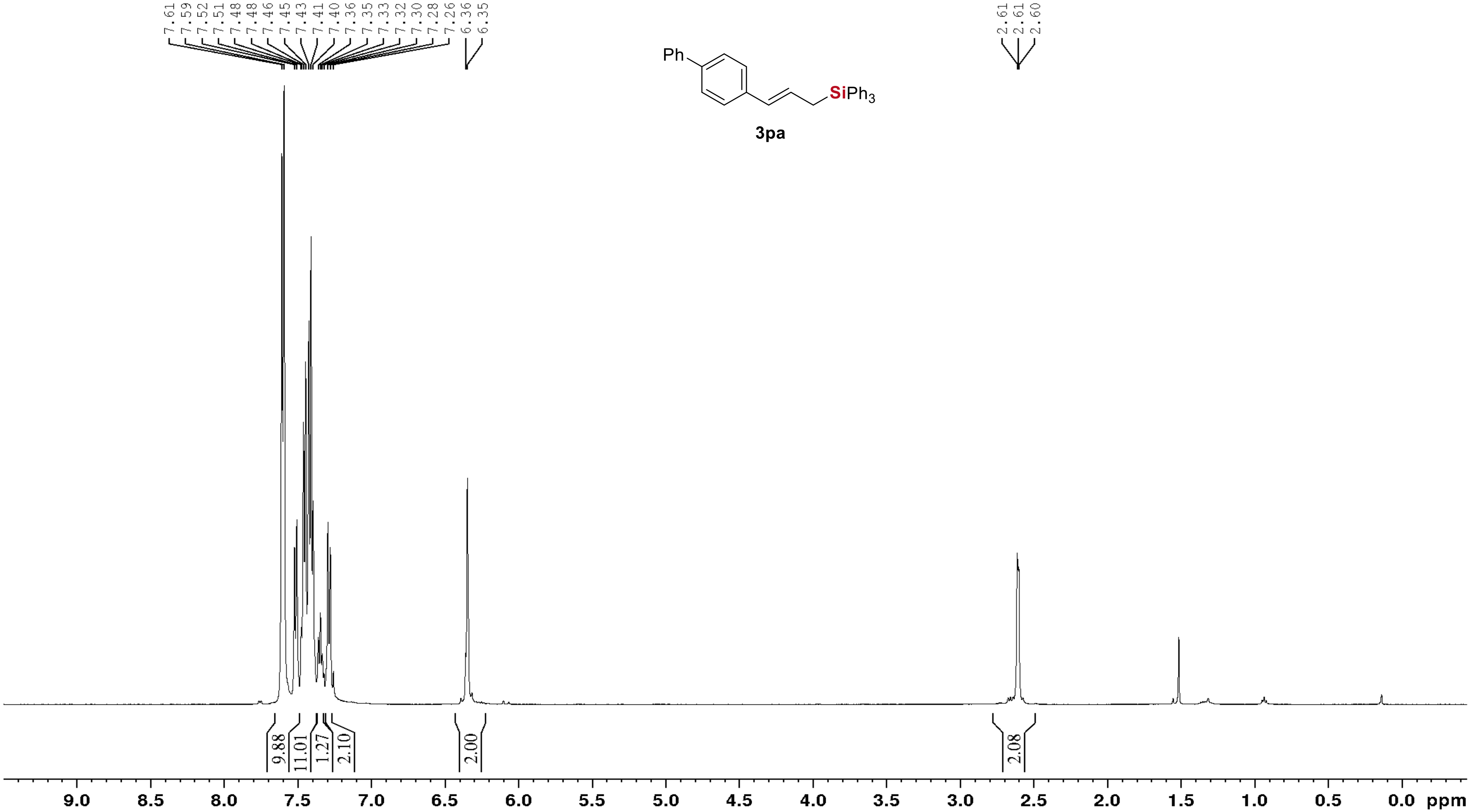

Figure S74. $^{13}C\{^{1}H\}$ NMR spectrum (126 MHz, $CDCl_3$, 298 K) of (*E*)-(3-([1,1'-biphenyl]-4-yl)allyl)triphenylsilane (3pa)

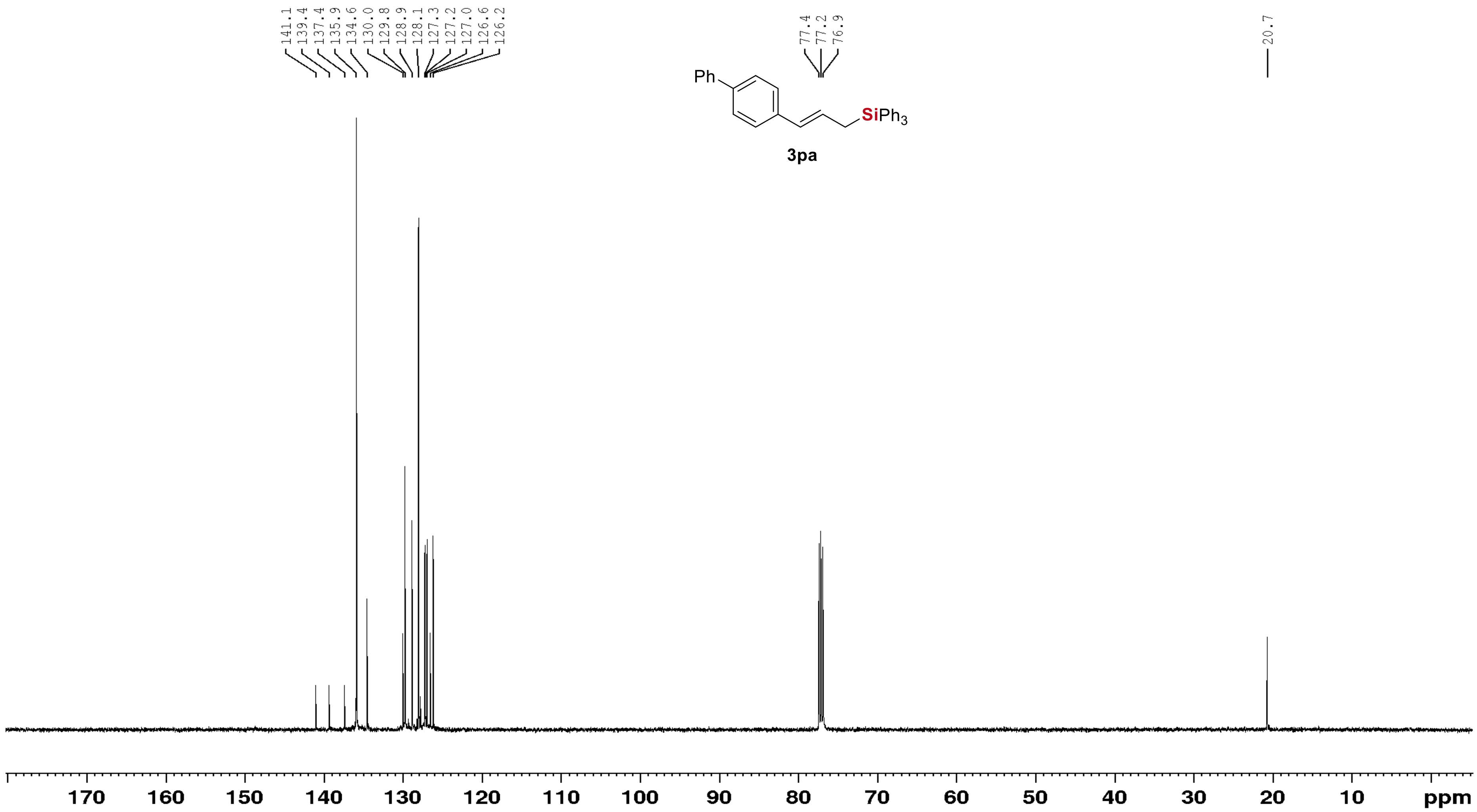

Figure S75. $^{1}H/^{29}Si$ **HMQC NMR spectrum (500/99 MHz, $CDCl_3$, 298 K, optimized for *J* = 7 Hz) of 3pa**

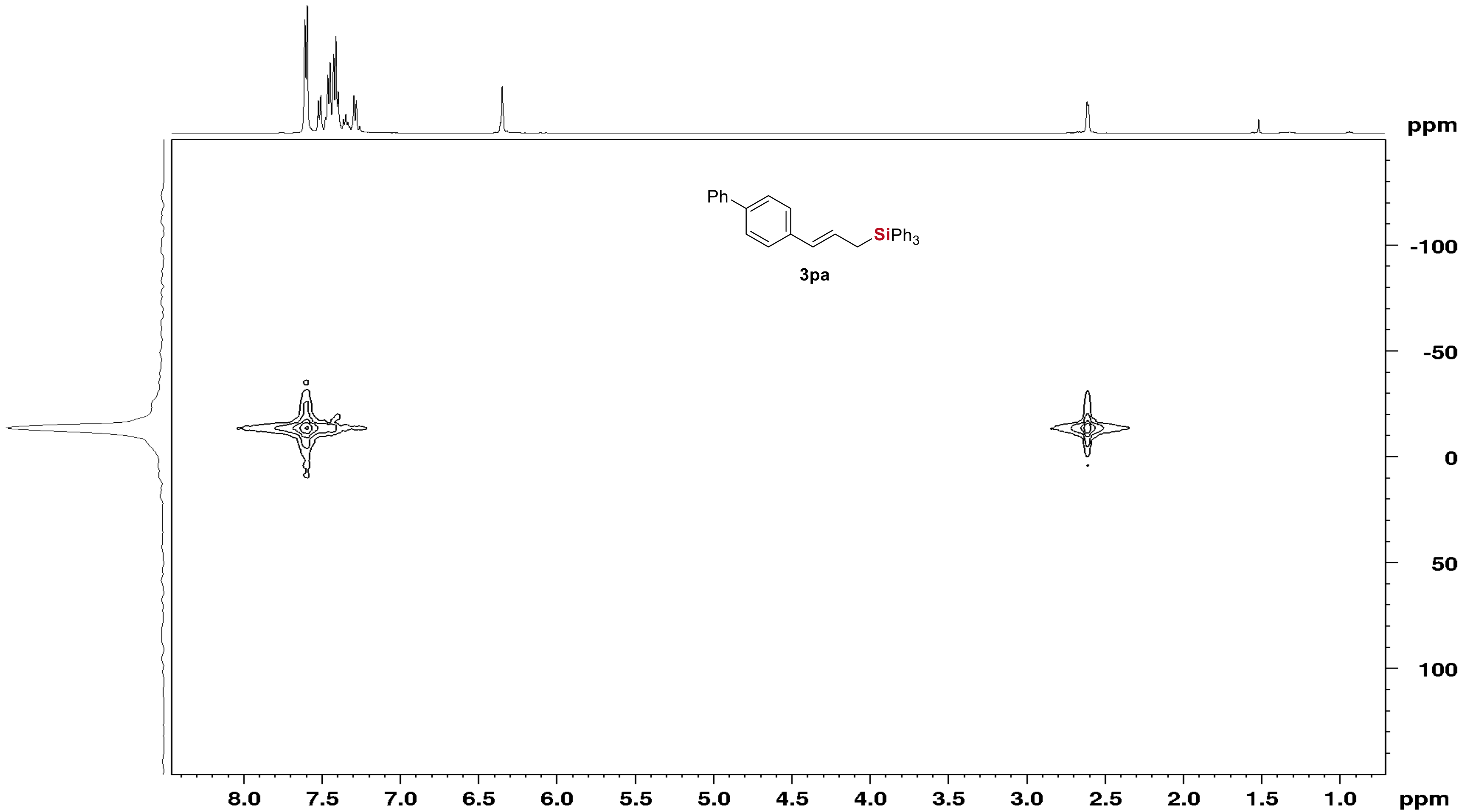

Figure S76. ^{1}H **NMR spectrum (500 MHz,** $CDCl_3$**, 298 K) of (*E*)-(3-(naphthalen-1-yl)allyl)triphenylsilane (3qa)**

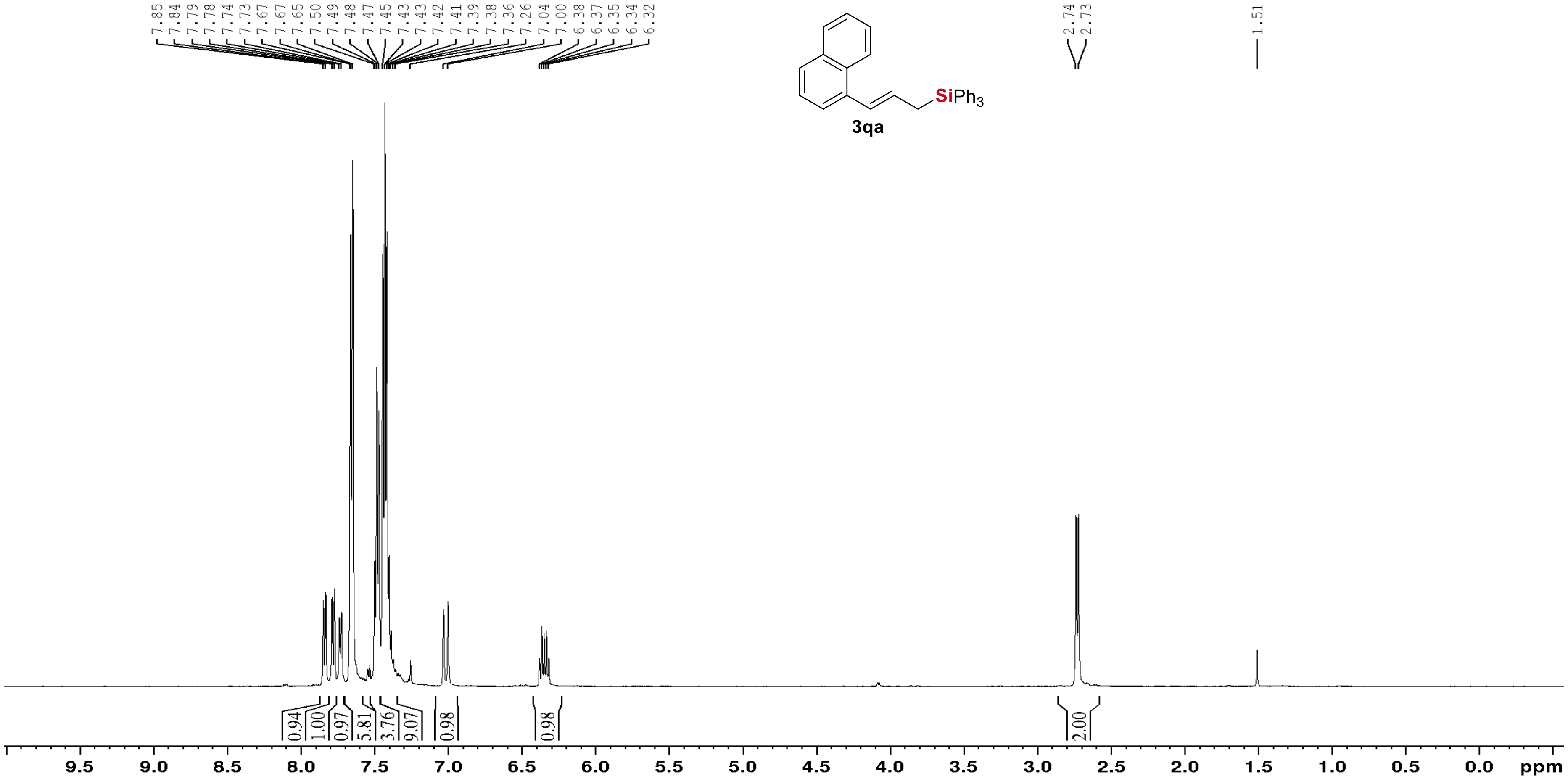

Figure S77. $^{13}C\{^{1}H\}$ NMR spectrum (126 MHz, $CDCl_3$, 298 K) of (*E*)-(3-(naphthalen-1-yl)allyl)triphenylsilane (3qa)

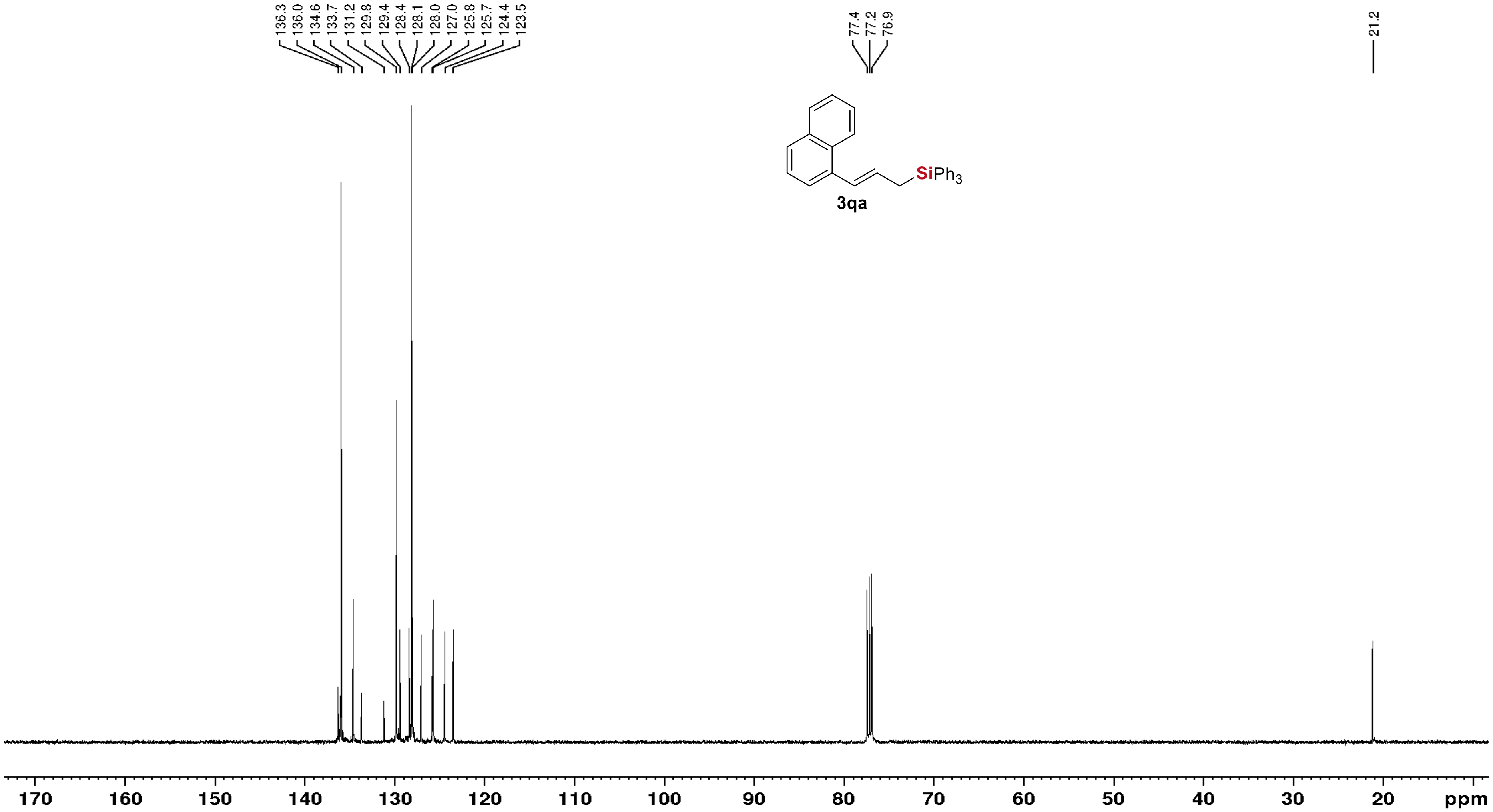

Figure S78. $^1H/^{29}Si$ **HMQC NMR spectrum (500/99 MHz, CDCl$_3$, 298 K, optimized for J = 7 Hz) of 3qa**

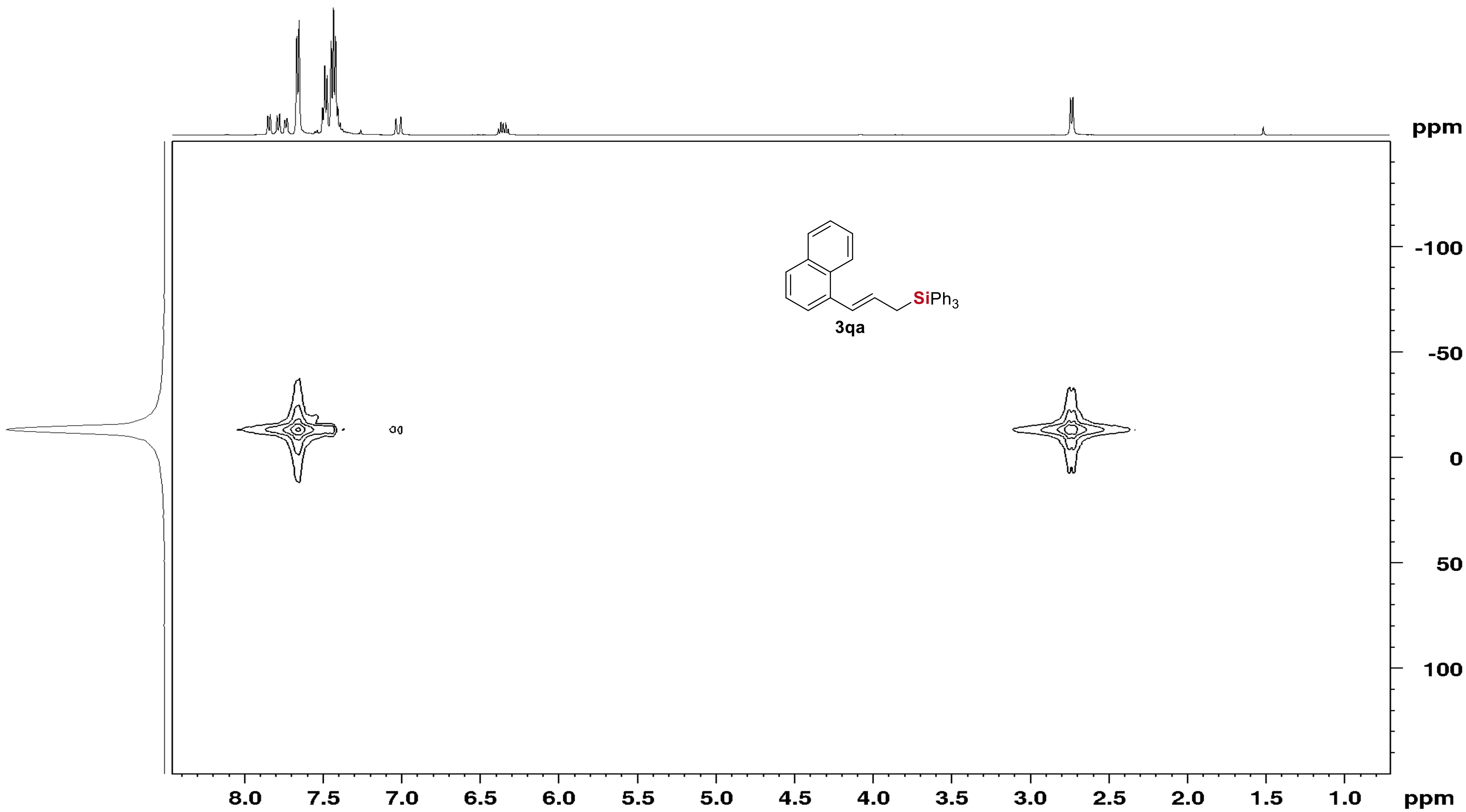

Figure S79. ^{1}H NMR spectrum (500 MHz, $CDCl_3$, 298 K) of (*E*)-(3-(naphthalen-2-yl)allyl)triphenylsilane (3ra)

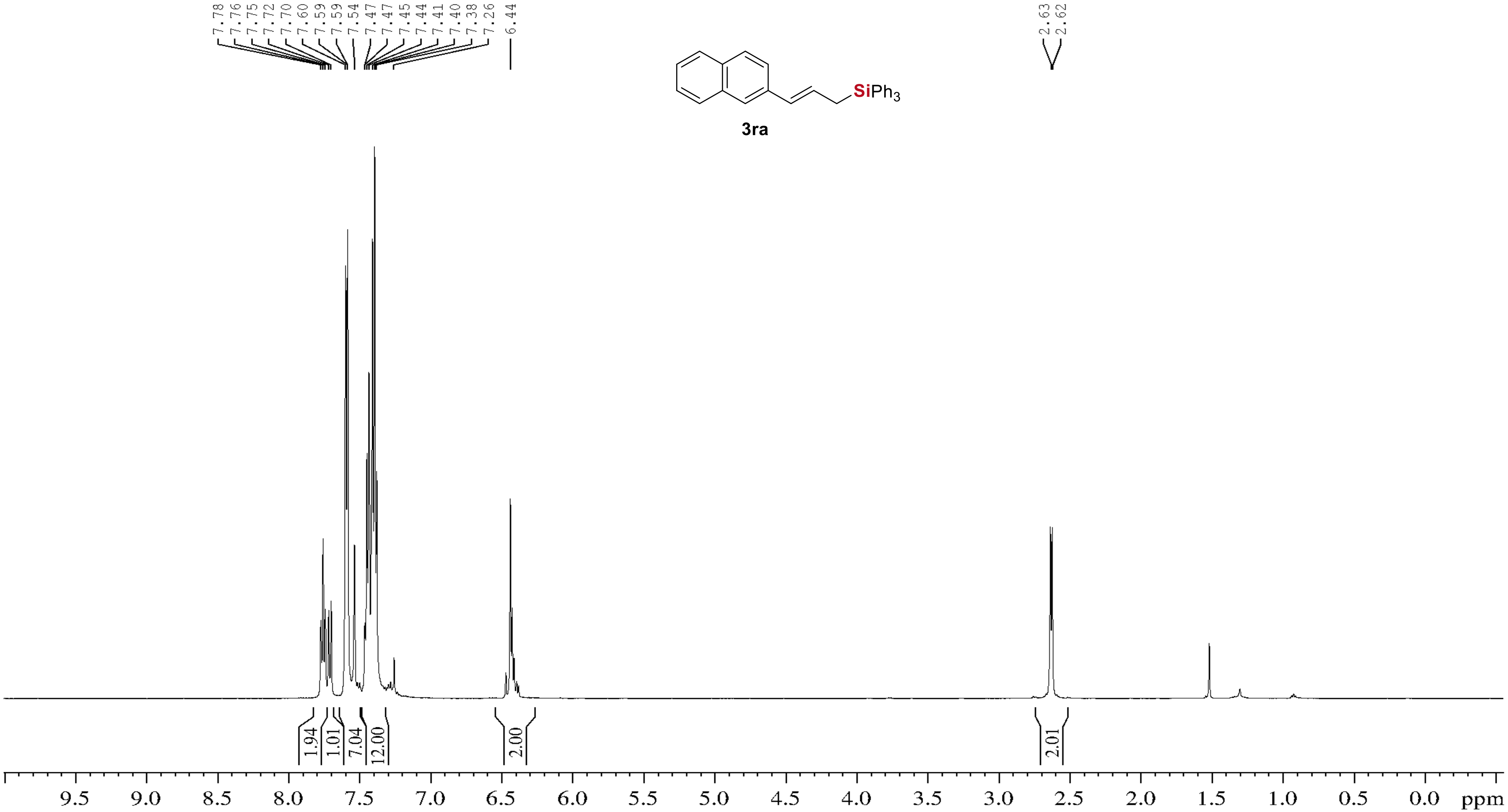

Figure S80. $^{13}C\{^1H\}$ **NMR spectrum (126 MHz, $CDCl_3$, 298 K) of (*E*)-(3-(naphthalen-2-yl)allyl)triphenylsilane (3ra)**

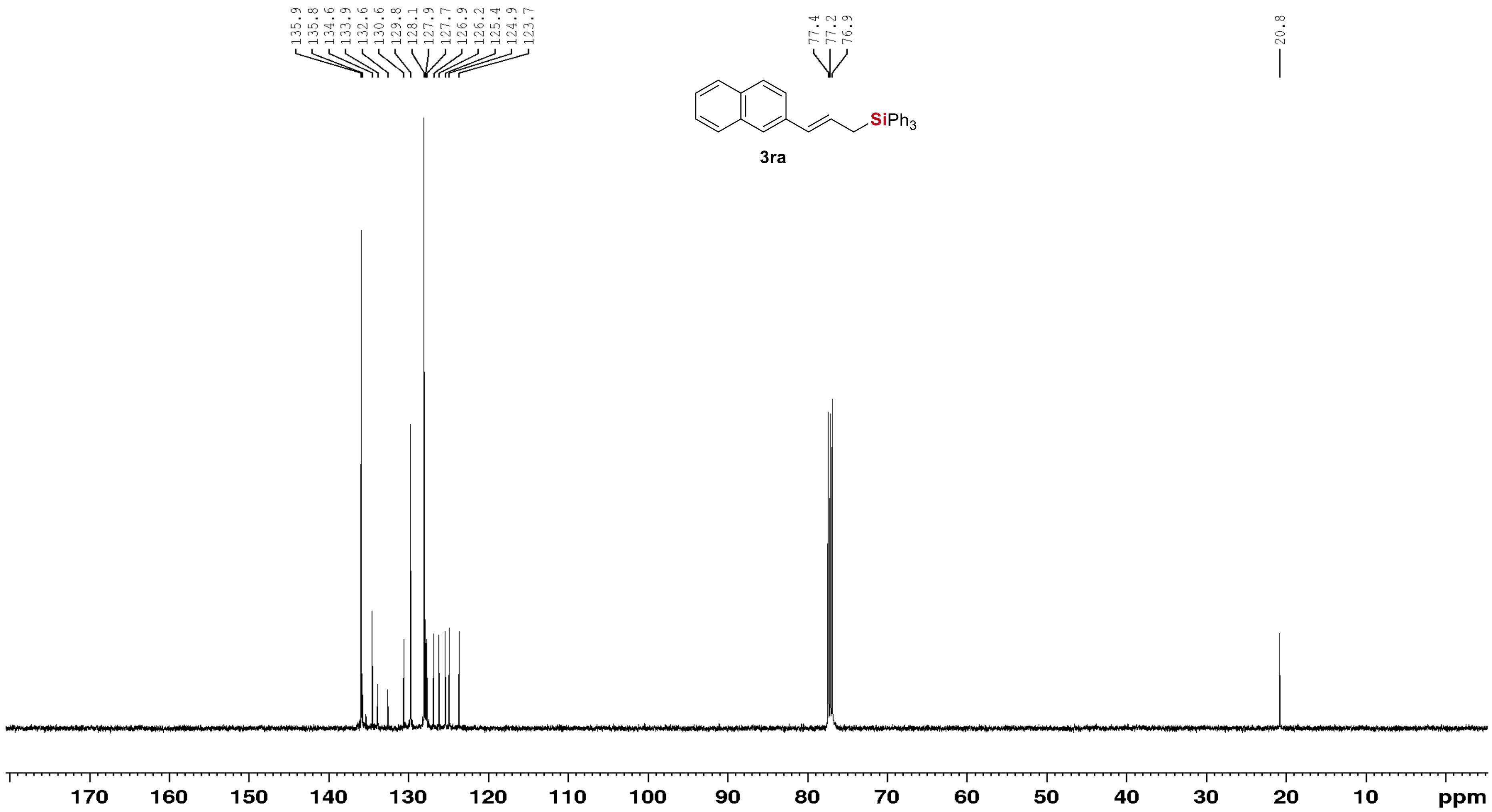

Figure S81. $^{1}H/^{29}Si$ **HMQC NMR spectrum (500/99 MHz, $CDCl_3$, 298 K, optimized for *J* = 7 Hz) of 3ra**

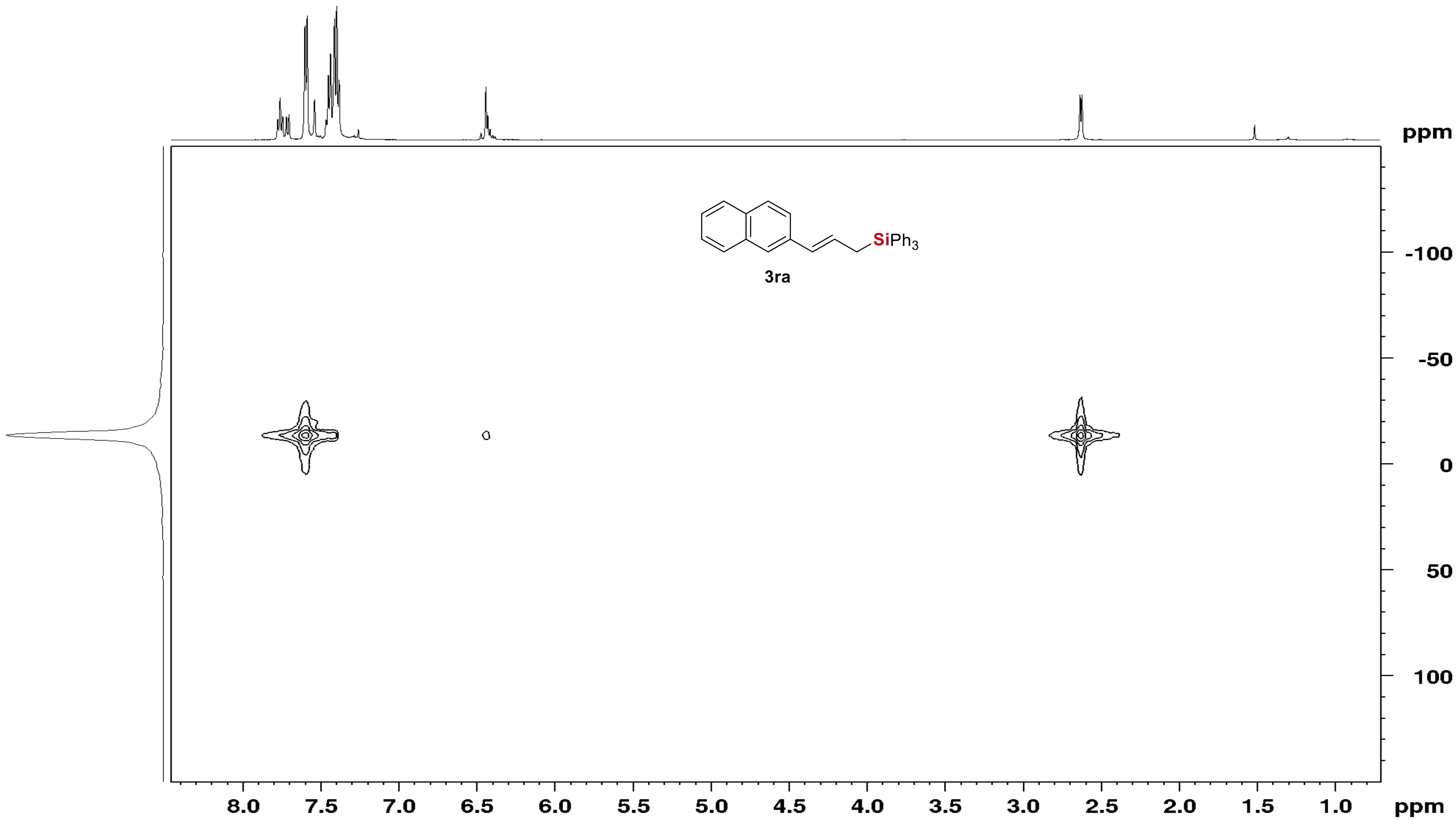

Figure S82. **^{1}H NMR spectrum (500 MHz, $CDCl_3$, 298 K) of (*E*)-triphenyl(3-(thiophen-2-yl)allyl)silane (3sa)**

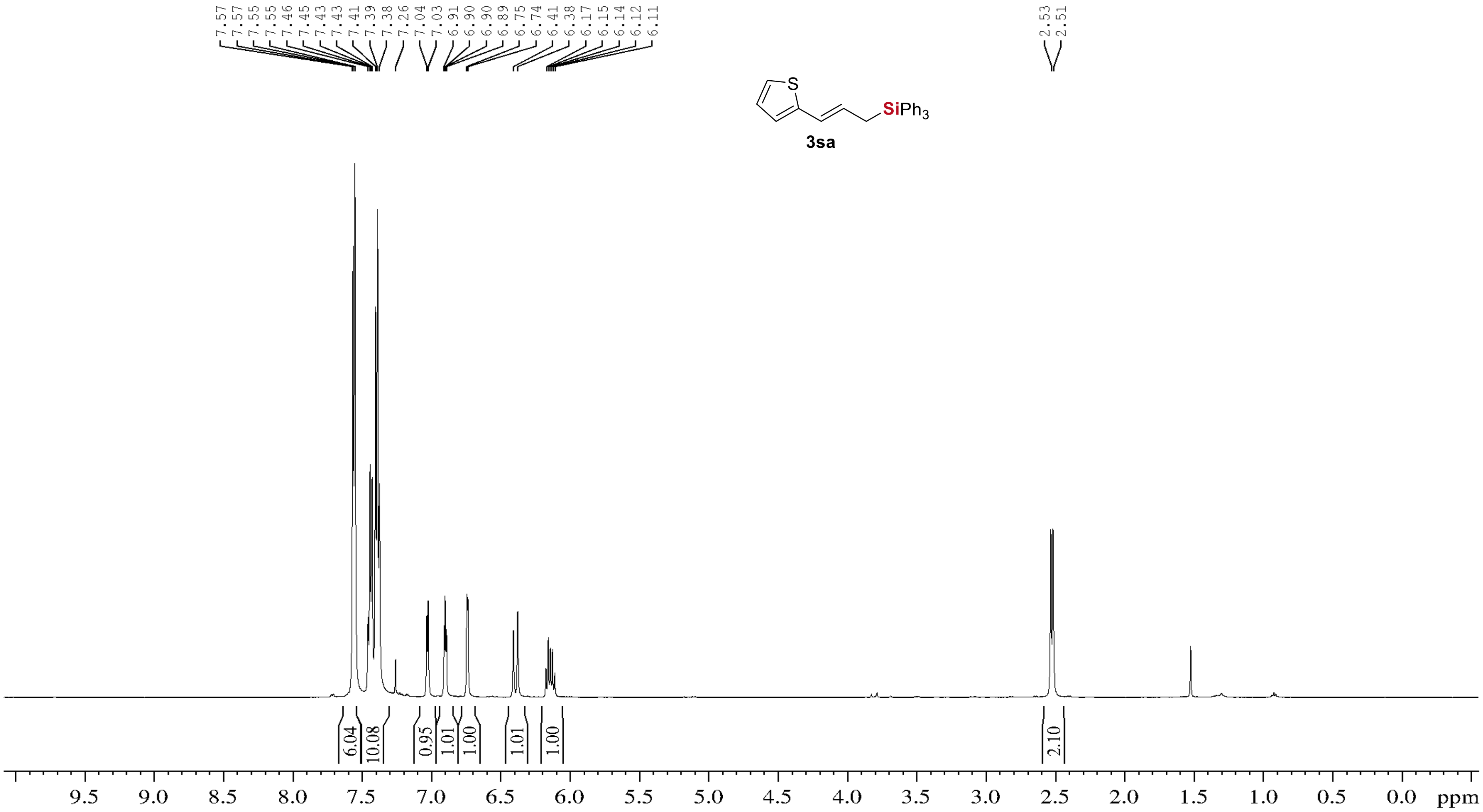

Figure S83. $^{13}C\{^{1}H\}$ NMR spectrum (126 MHz, $CDCl_3$, 298 K) of (*E*)-triphenyl(3-(thiophen-2-yl)allyl)silane (3sa)

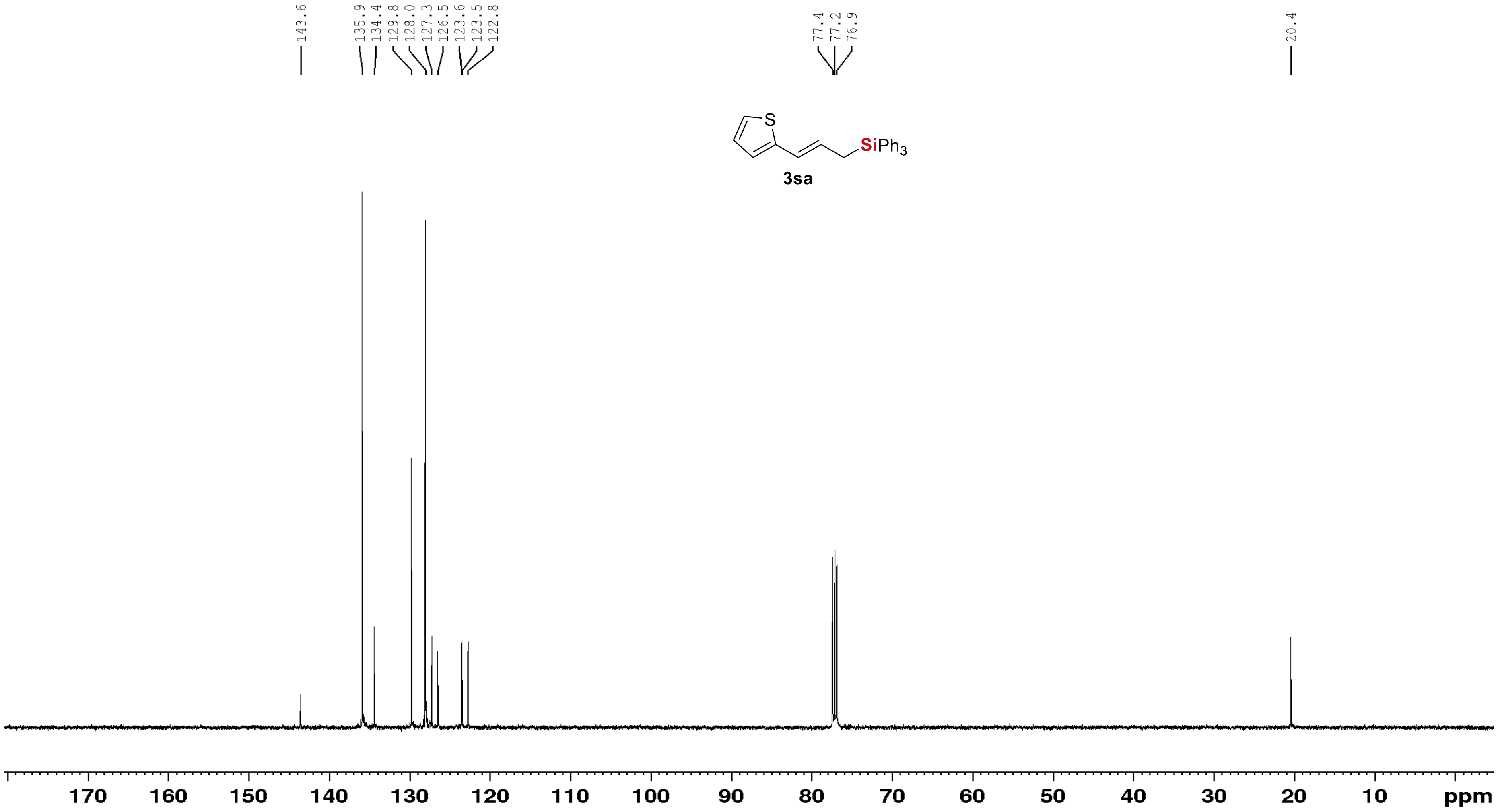

Figure S84. ^{1}**H/**^{29}**Si HMQC NMR spectrum (500/99 MHz, $CDCl_3$, 298 K, optimized for *J* = 7 Hz) of 3sa**

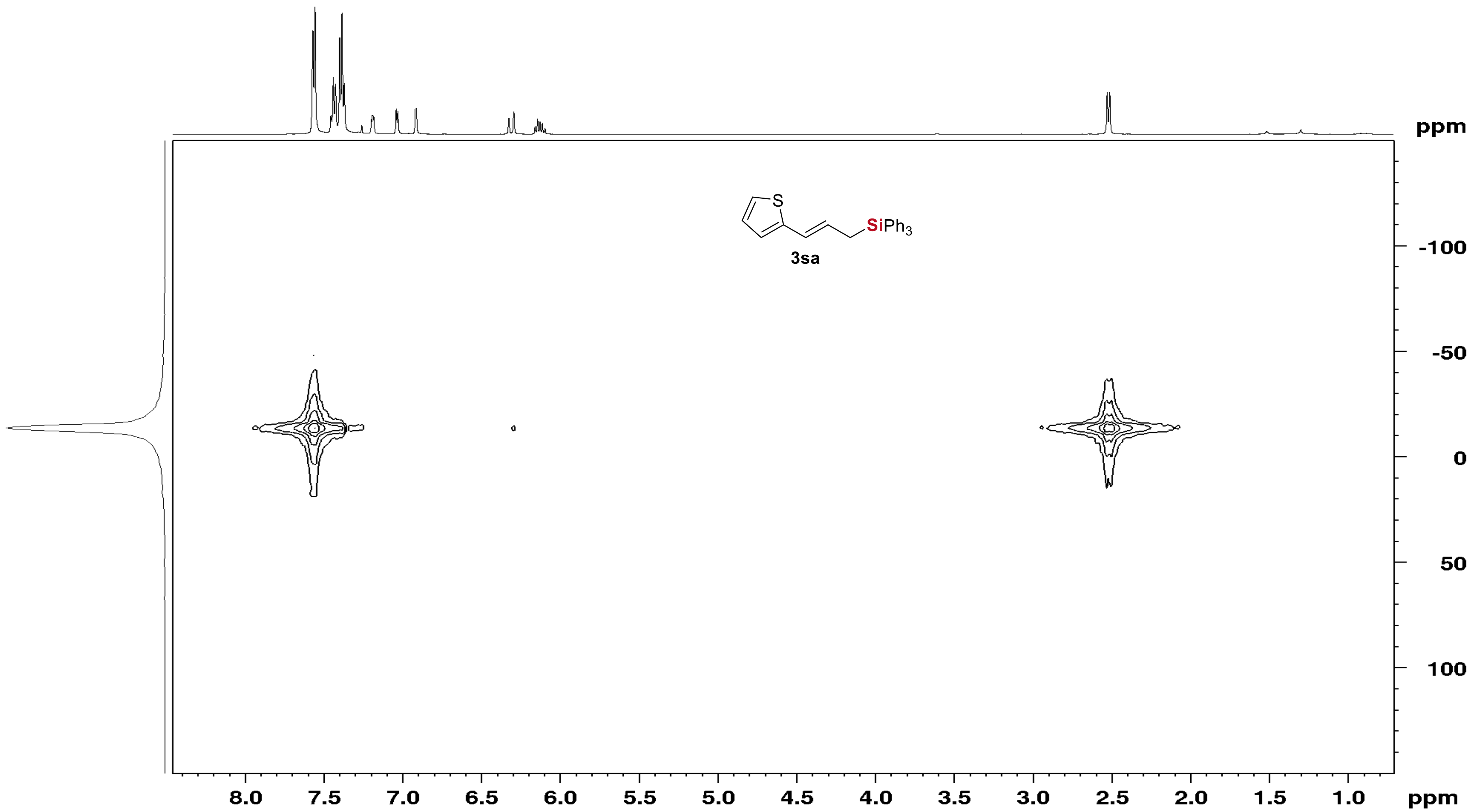

Figure S85. ^{1}H NMR spectrum (500 MHz, $CDCl_3$, 298 K) of (*E*)-triphenyl(3-(thiophen-3-yl)allyl)silane (3ta)

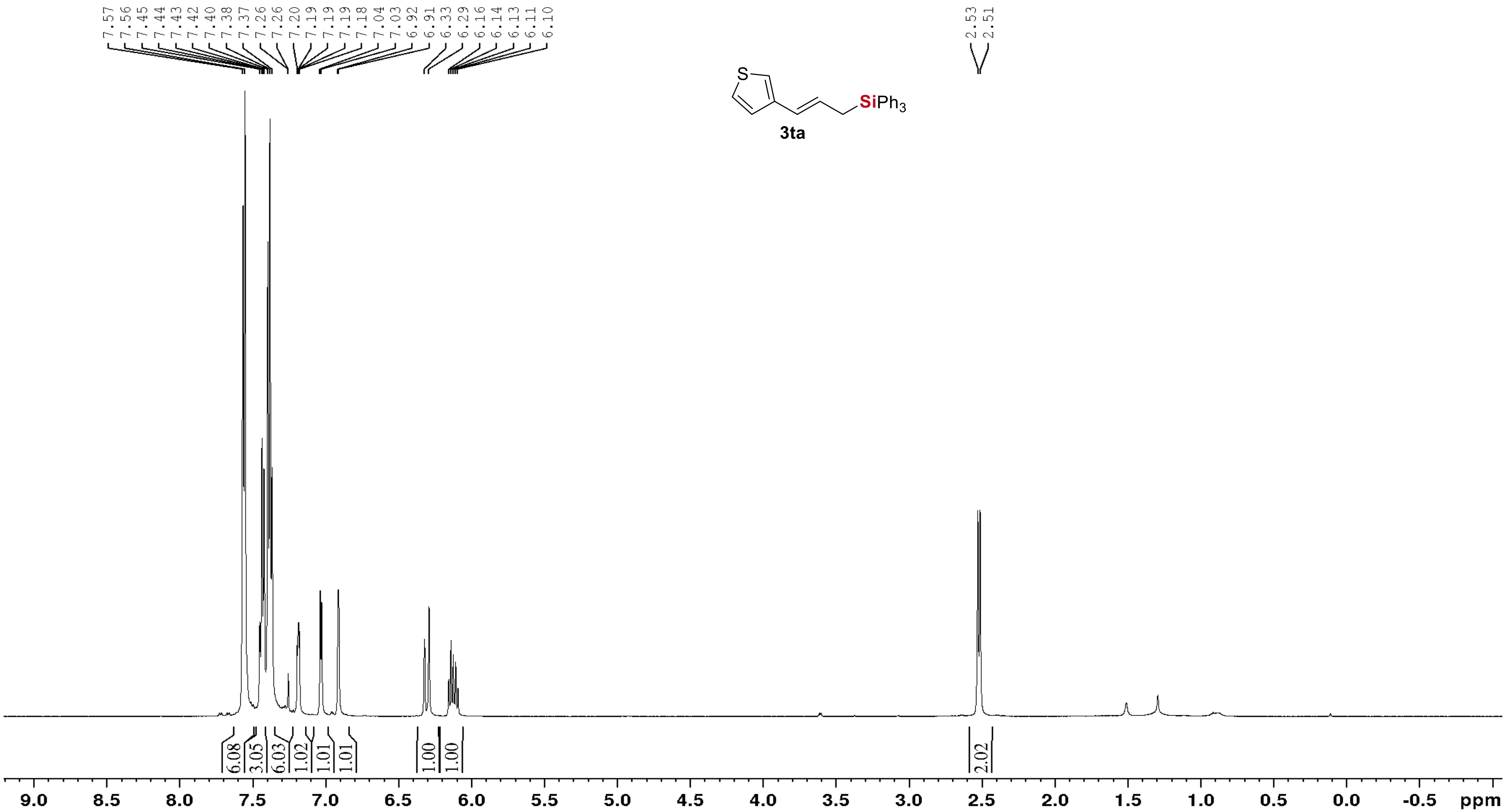

Figure S86. $^{13}C\{^1H\}$ NMR spectrum (126 MHz, $CDCl_3$, 298 K) of (*E*)-triphenyl(3-(thiophen-3-yl)allyl)silane (3ta)

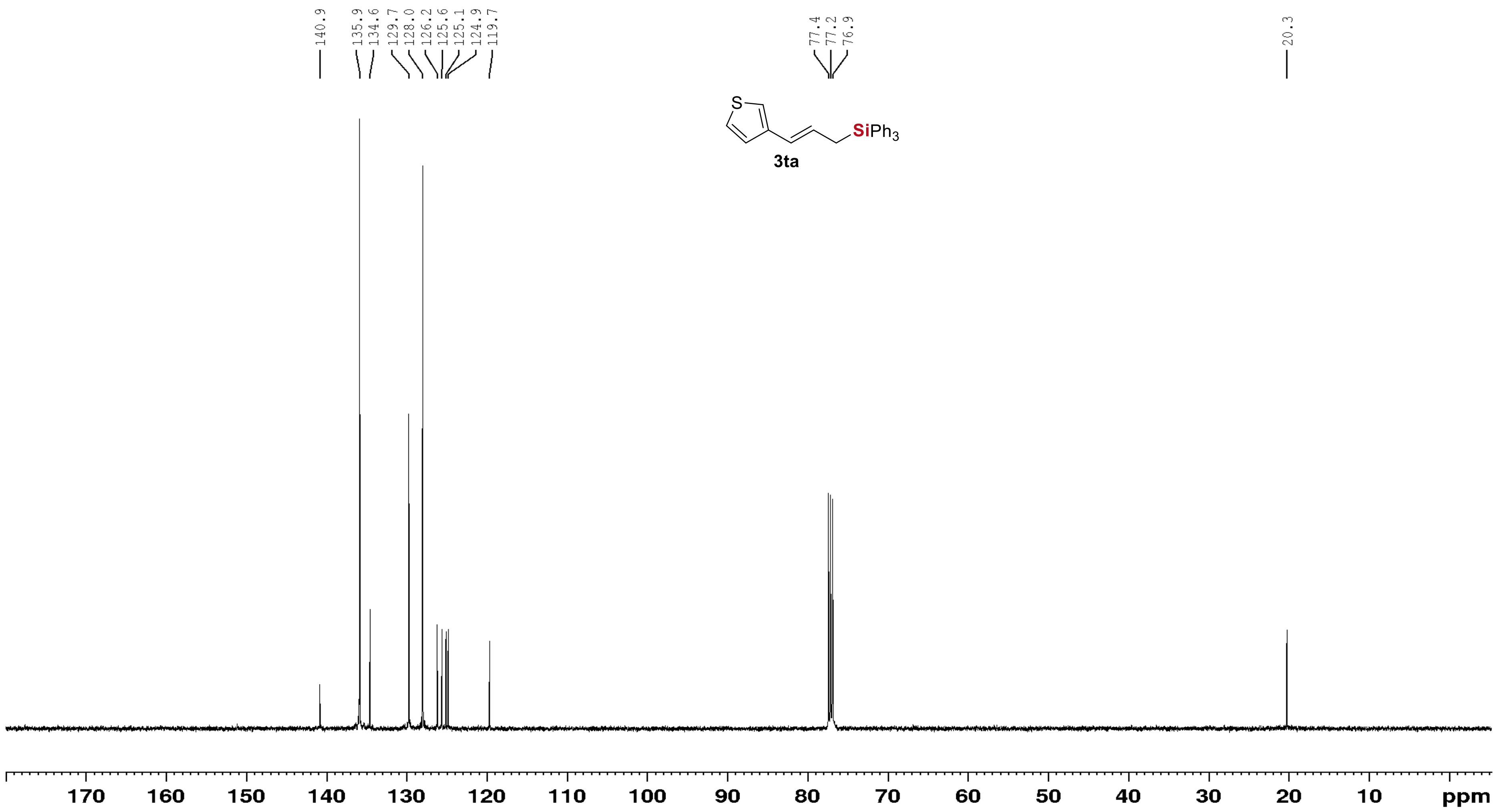

Figure S87. **$^{1}H/^{29}Si$ HMQC NMR spectrum (500/99 MHz, $CDCl_3$, 298 K, optimized for *J* = 7 Hz) of 3ta**

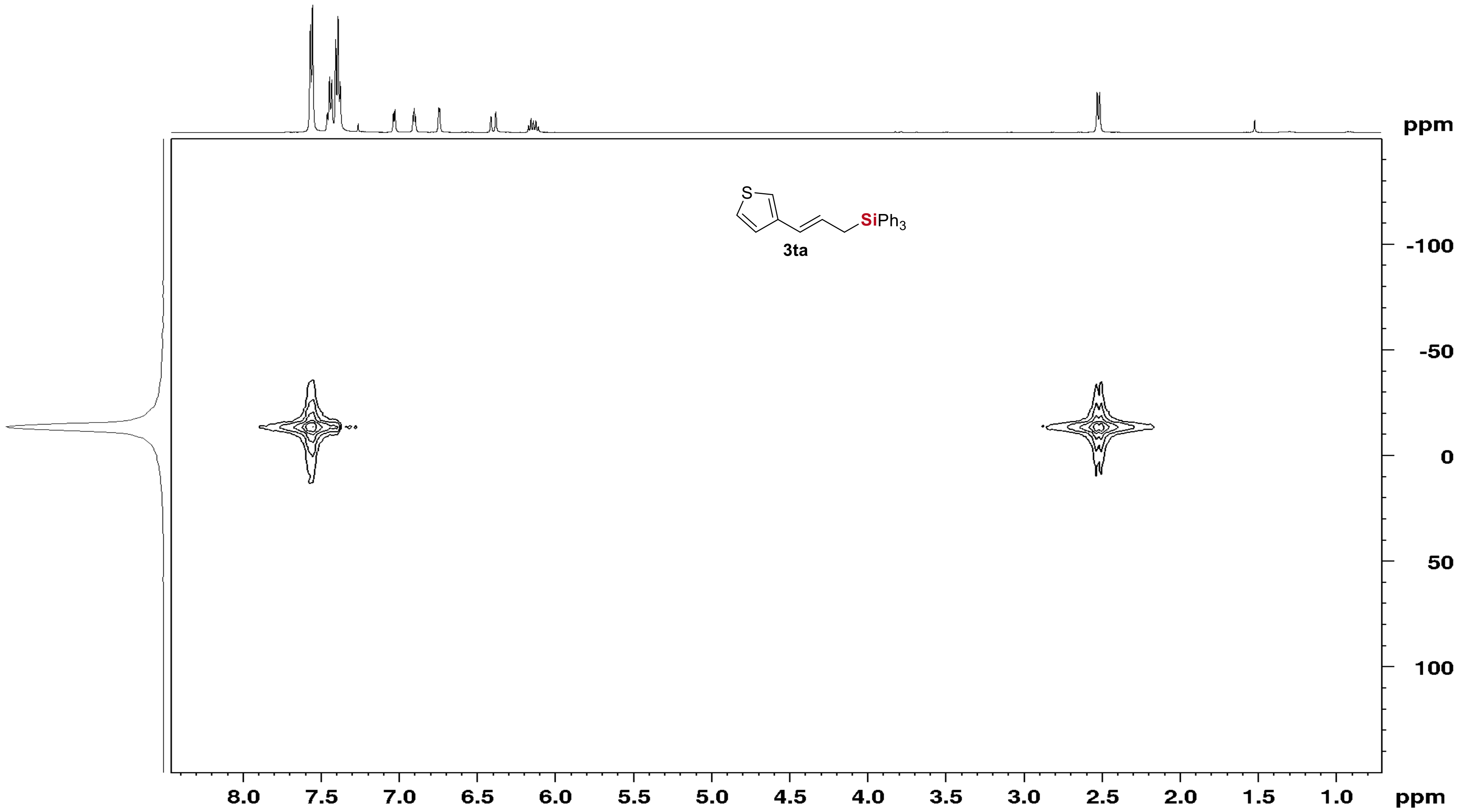

Figure S88. ¹H NMR spectrum (500 MHz, $CDCl_3$, 298 K) of (*E*)-cinnamyltrimethylsilane (3ab)

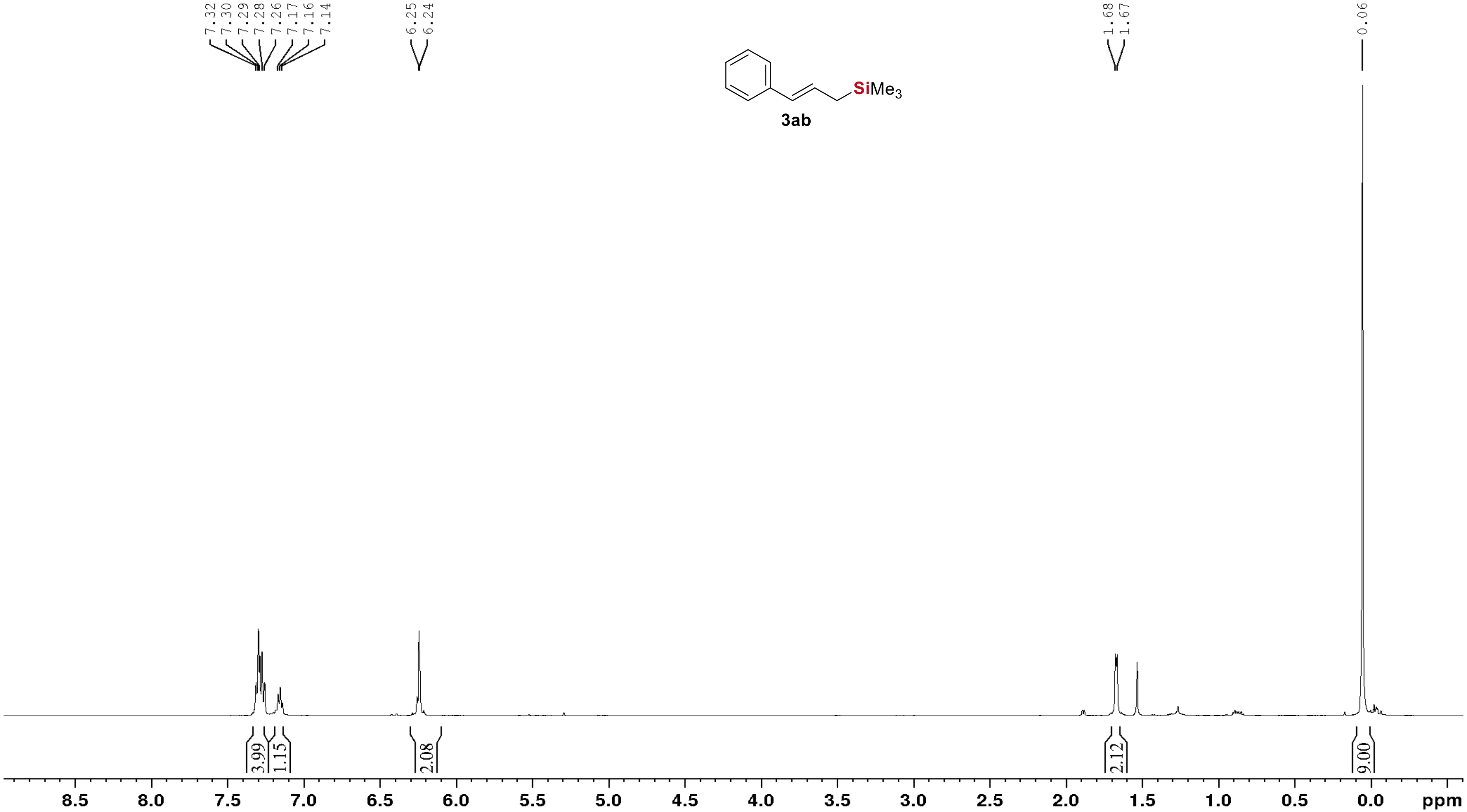

Figure S89. $^{13}C\{^{1}H\}$ **NMR spectrum (126 MHz, $CDCl_3$, 298 K) of (*E*)-cinnamyltrimethylsilane (3ab)**

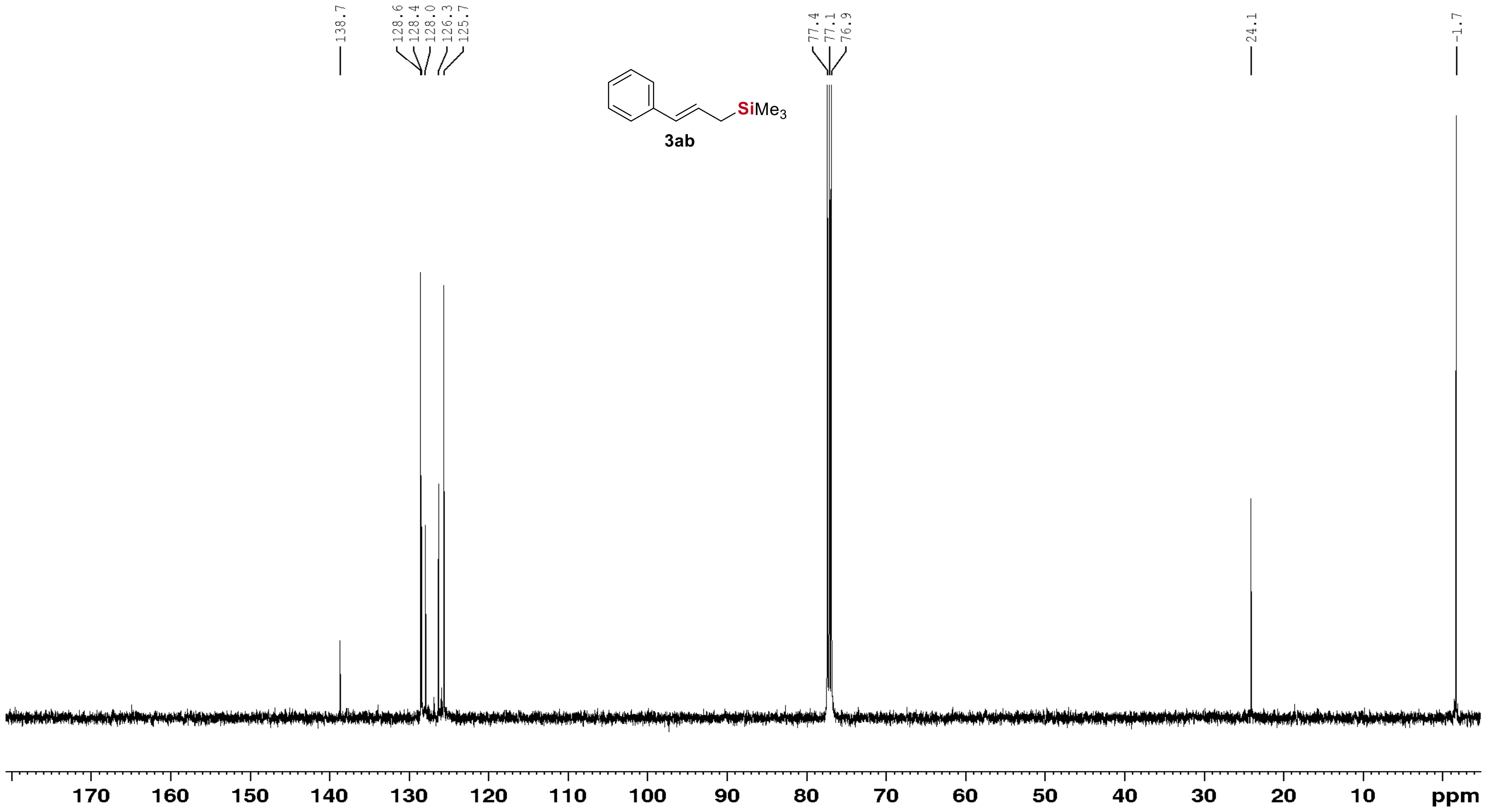

Figure S90. $^{1}H/^{29}Si$ HMQC NMR spectrum (500/99 MHz, $CDCl_3$, 298 K, optimized for J = 7 Hz) of 3ab

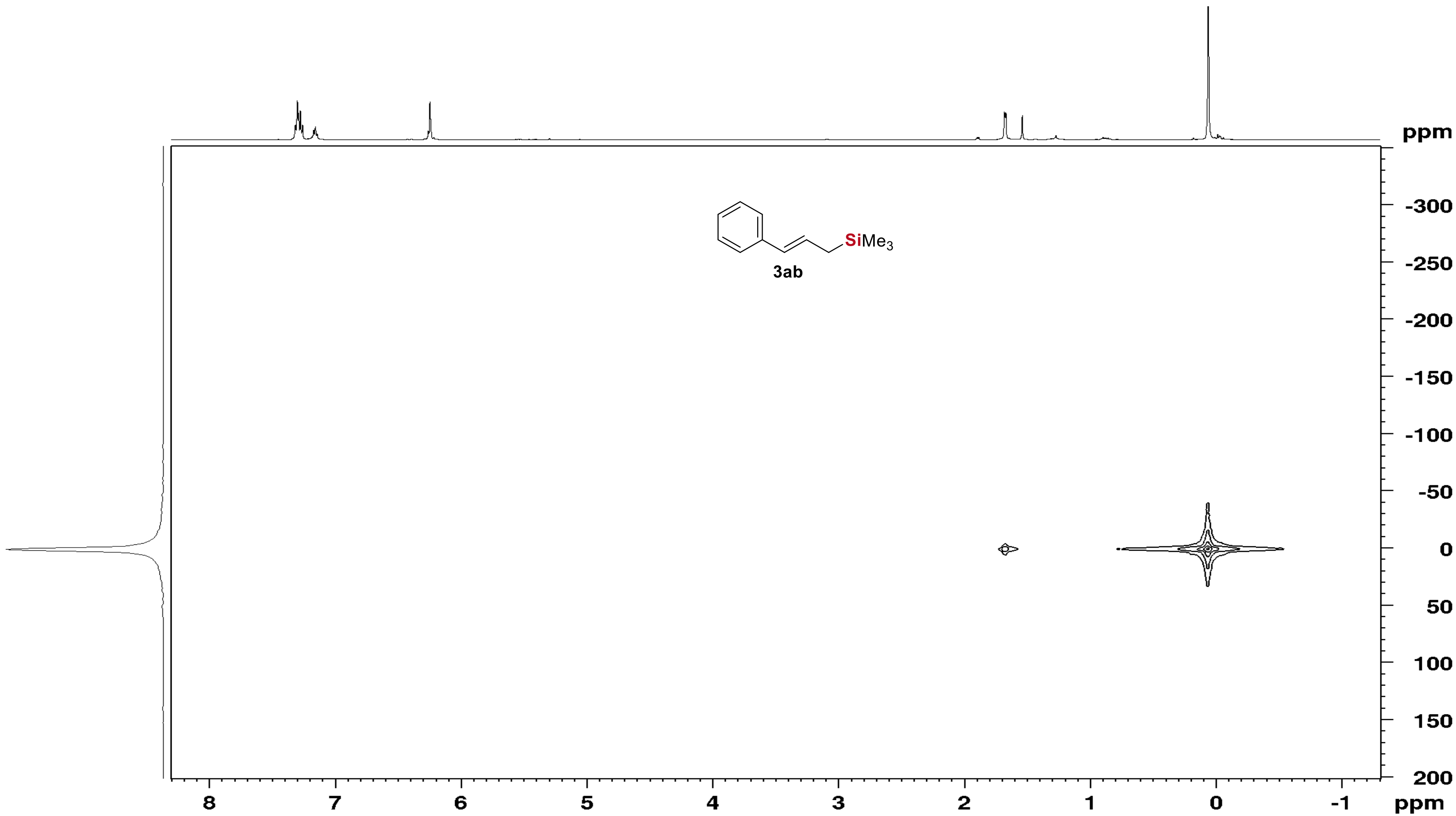

Figure S91. ^{1}H NMR spectrum (500 MHz, $CDCl_3$, 298 K) of (*E*)-(3-(4-fluorophenyl)allyl)trimethylsilane (3gb)

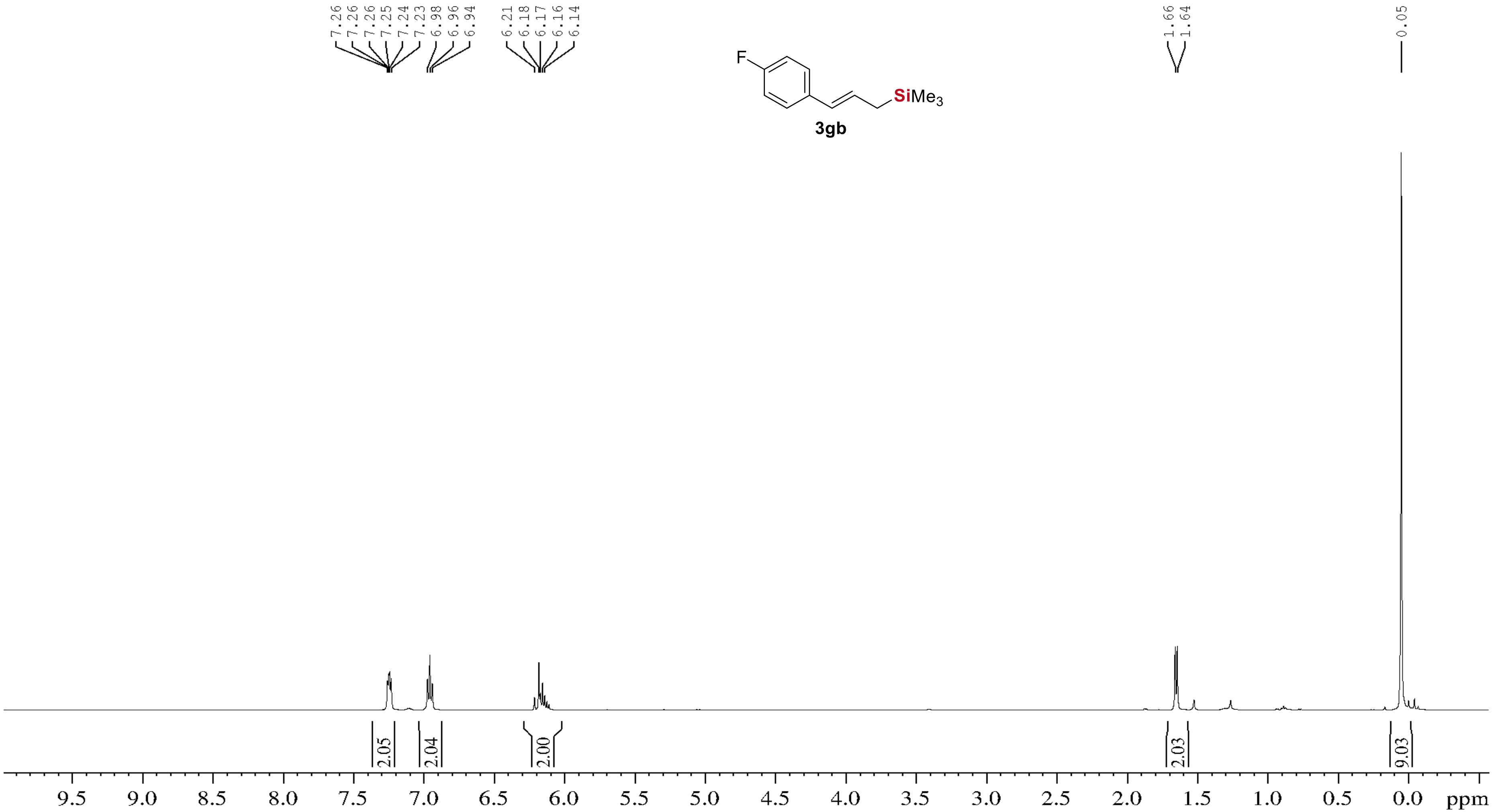

Figure S92. ^{19}F NMR spectrum (471 MHz, $CDCl_3$, 298 K) of (*E*)-(3-(4-fluorophenyl)allyl)trimethylsilane (3gb)

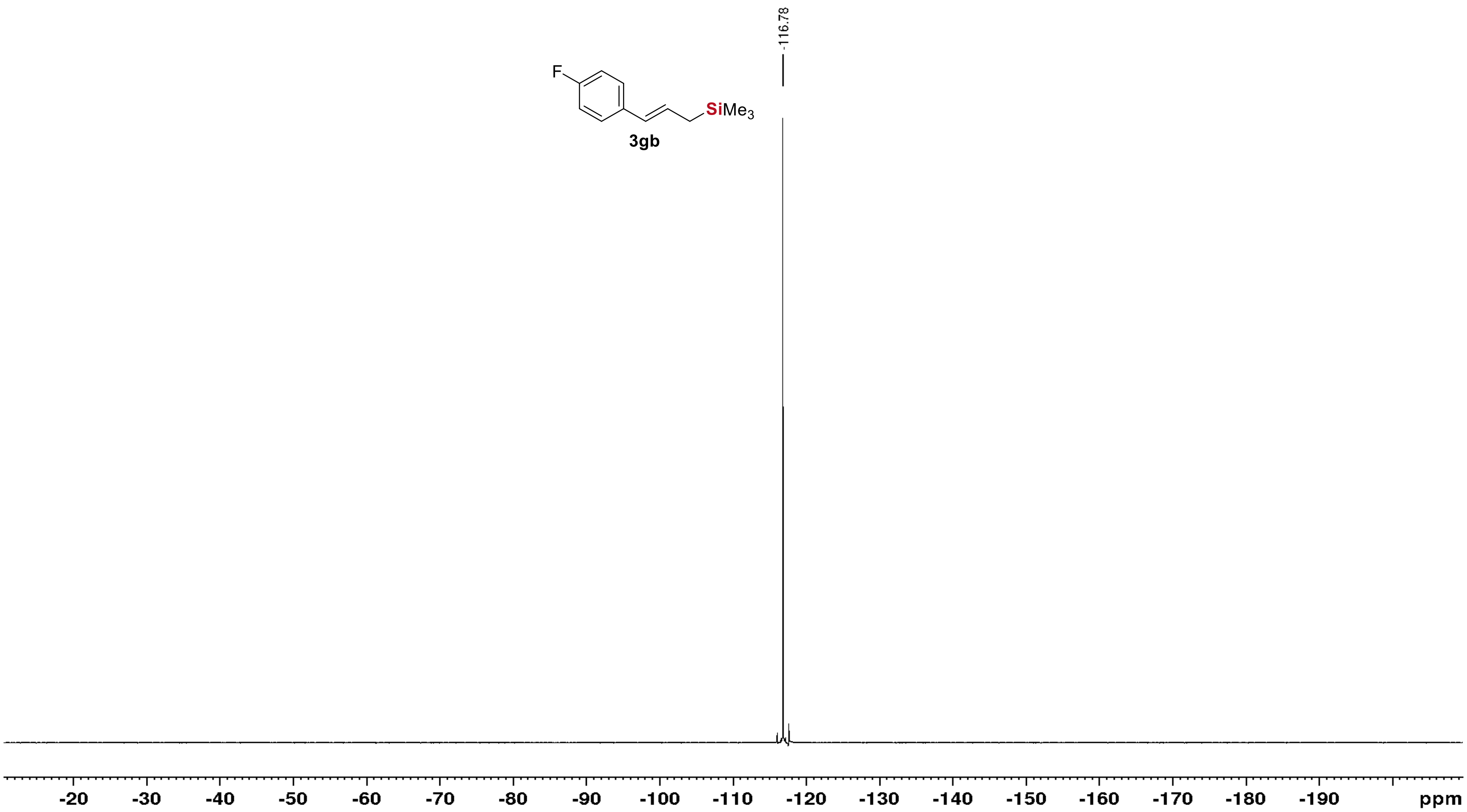

Figure S93. $^{13}C\{^{1}H\}$ NMR spectrum (126 MHz, $CDCl_3$, 298 K) of (*E*)-(3-(4-fluorophenyl)allyl)trimethylsilane (3gb)

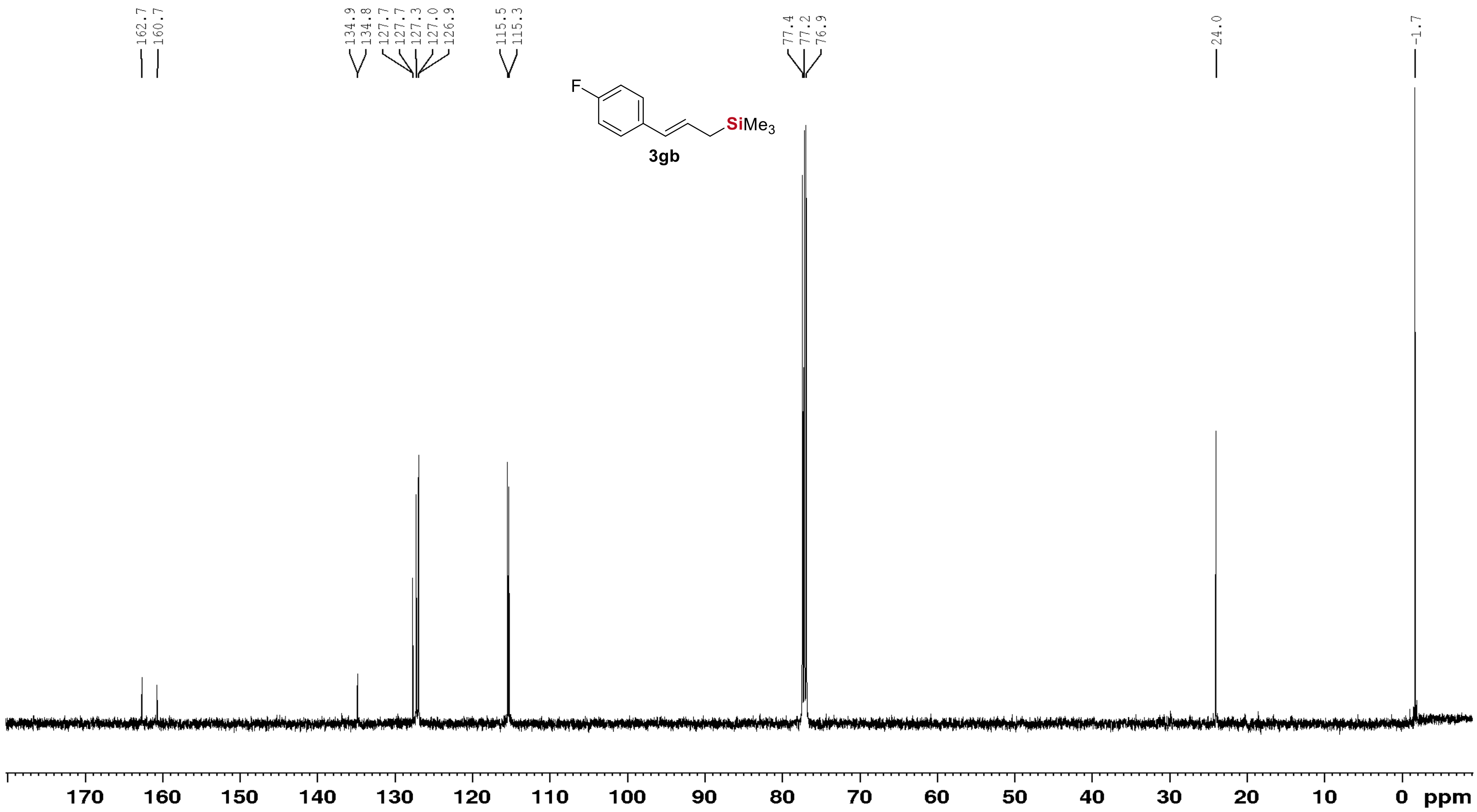

Figure S94. $^{1}H/^{29}Si$ **HMQC NMR spectrum (500/99 MHz, $CDCl_3$, 298 K, optimized for *J* = 7 Hz) of 3gb**

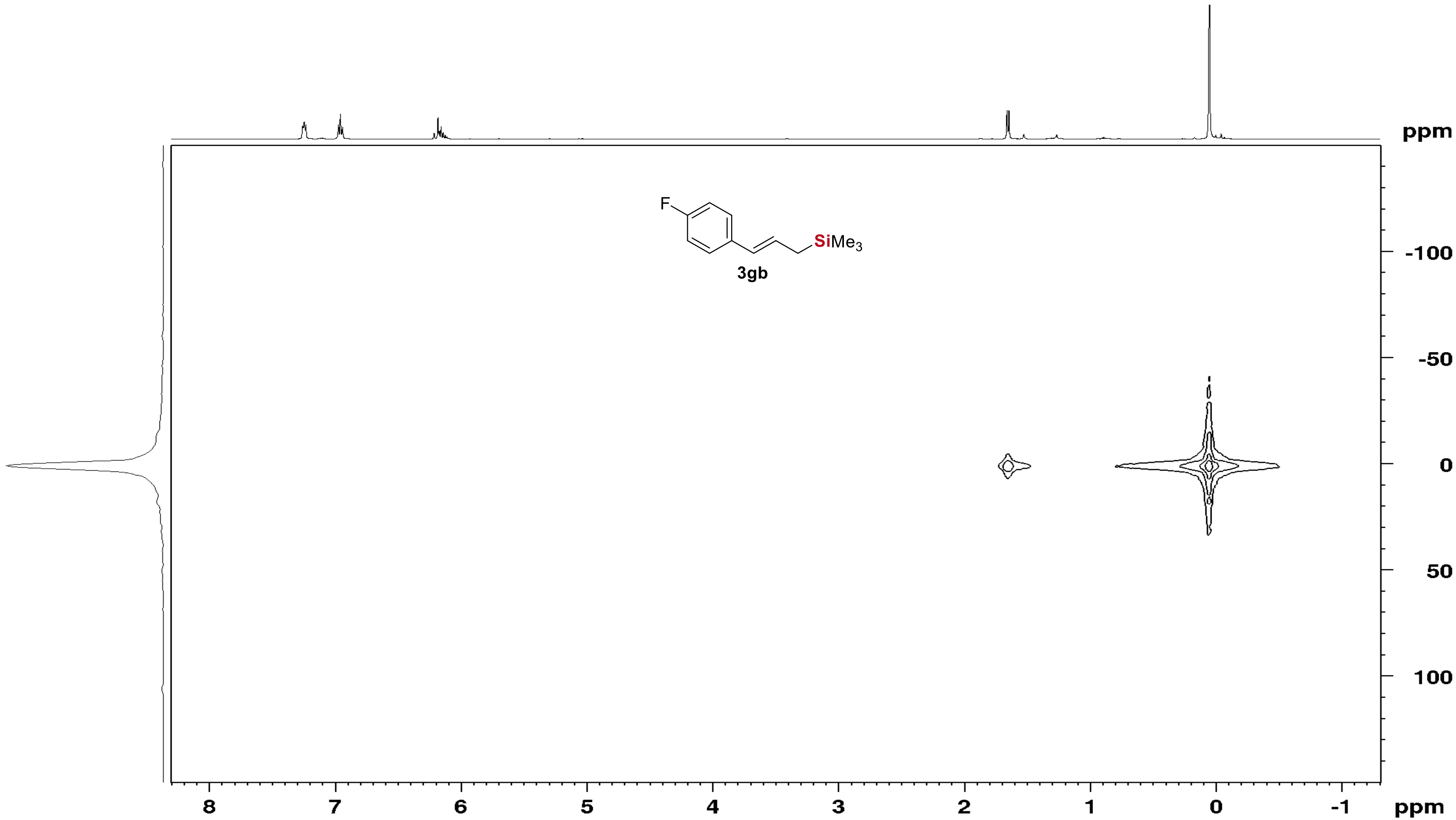

Figure S95. ^{1}**H NMR spectrum (500 MHz, CDCl$_3$, 298 K) of (*E*)-tributyl(cinnamyl)silane (3ac)**

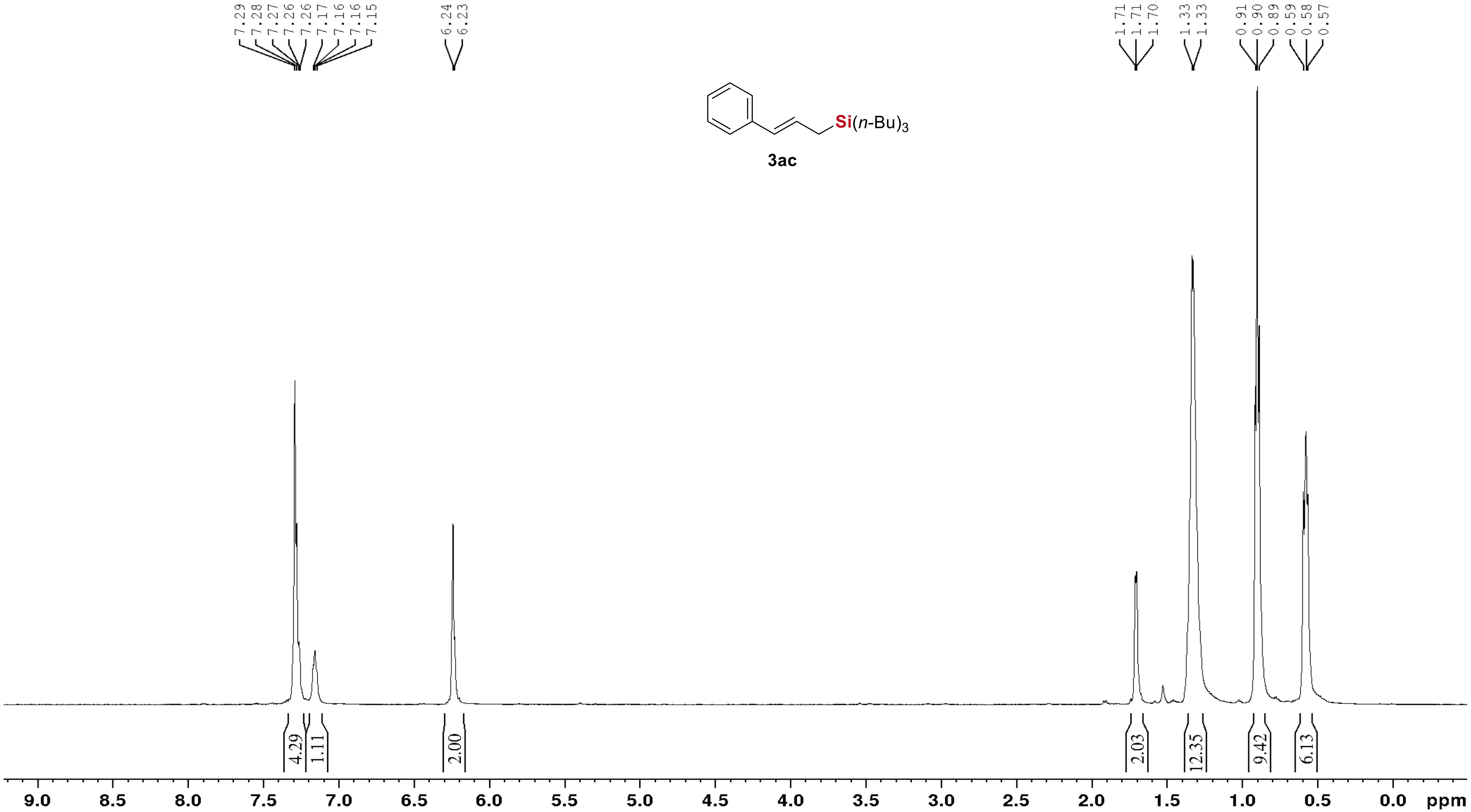

Figure S96. $^{13}C\{^1H\}$ **NMR spectrum (126 MHz, $CDCl_3$, 298 K) of (*E*)- tributyl(cinnamyl)silane (3ac)**

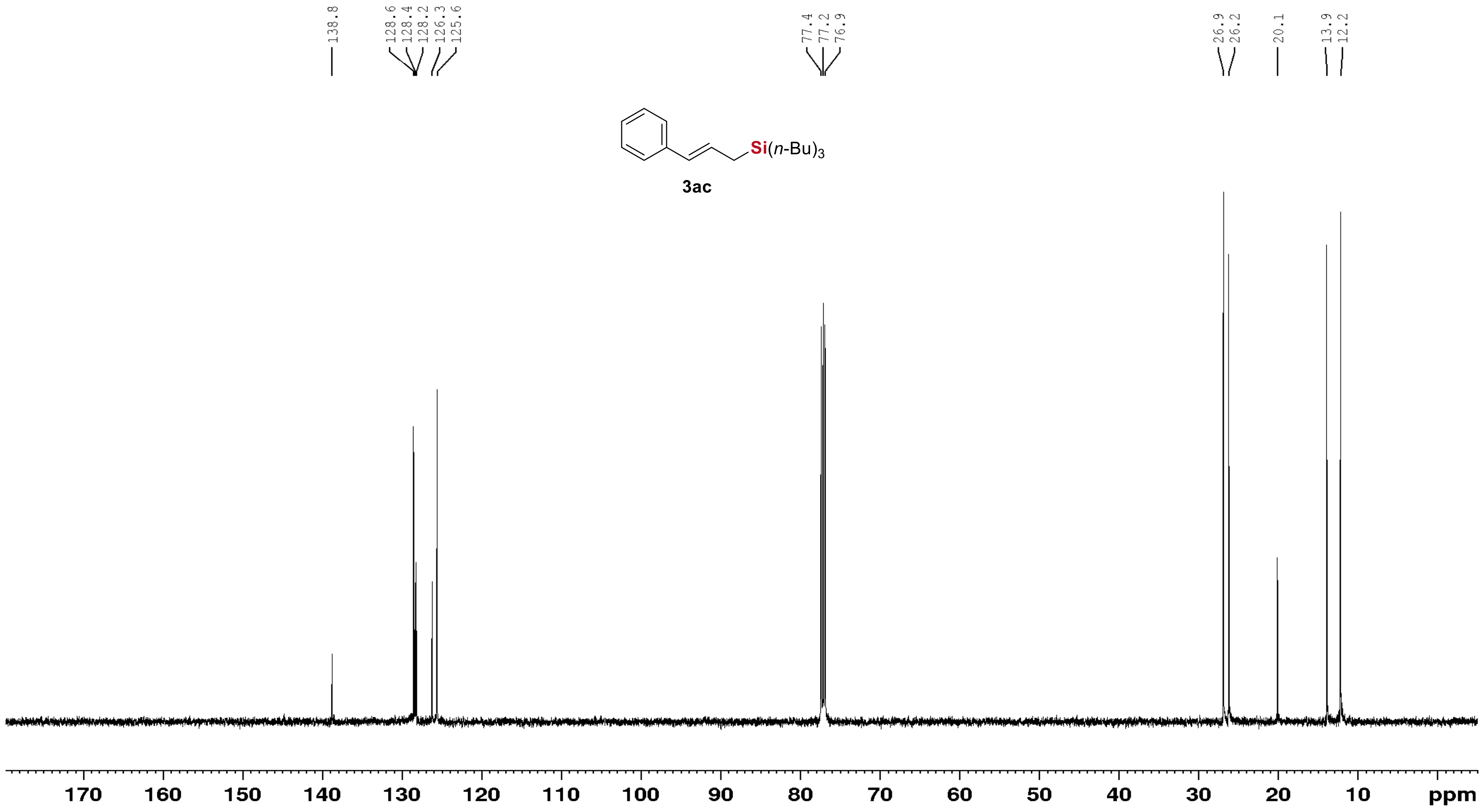

Figure S97. $^1H/^{29}Si$ **HMQC NMR spectrum (500/99 MHz, $CDCl_3$, 298 K, optimized for *J* = 7 Hz) of 3ac**

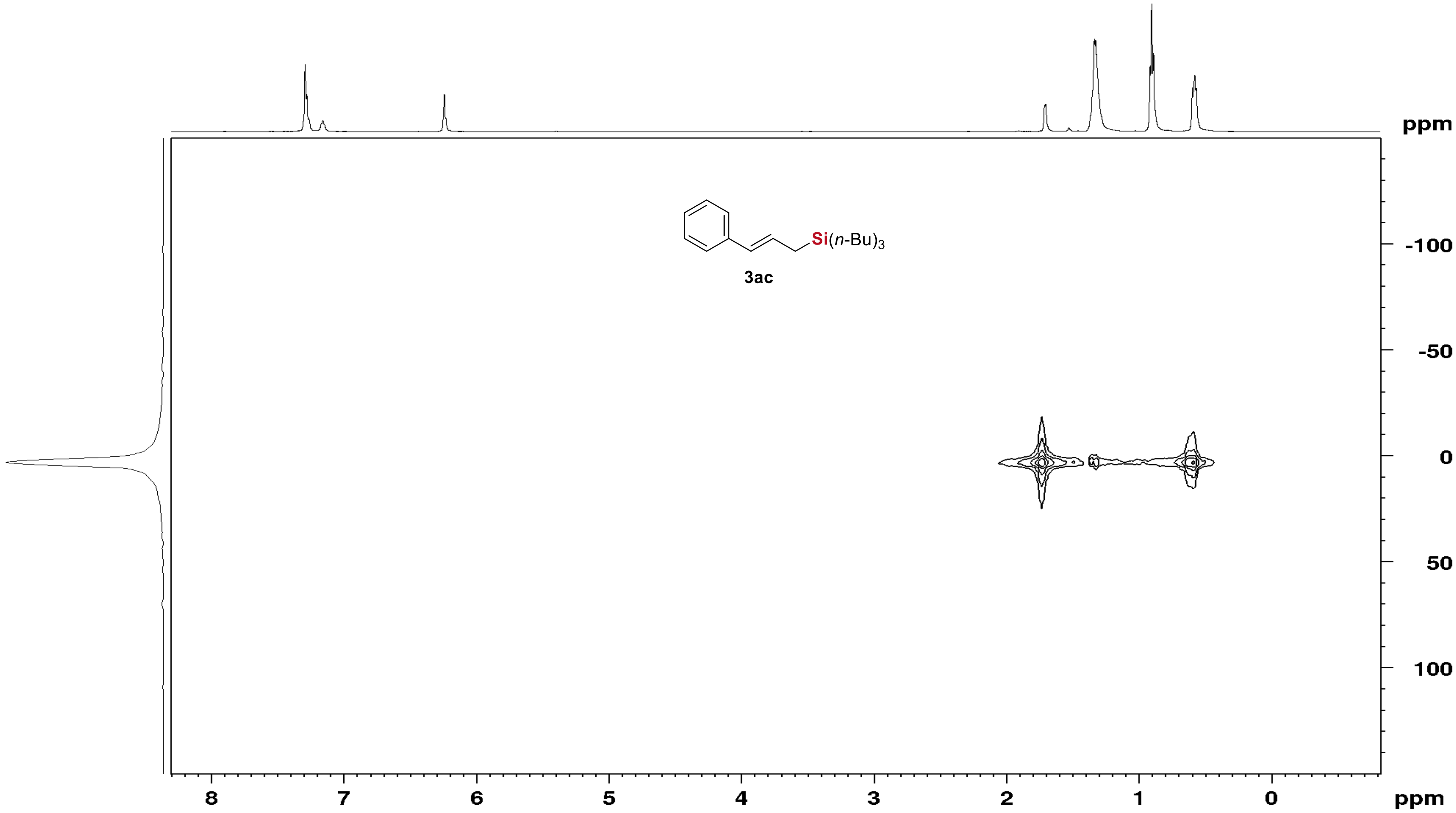

Figure S98. ¹H NMR spectrum (500 MHz, $CDCl_3$, 298 K) of (*E*)-tributyl(3-(4-(*tert*-butyl)phenyl)allyl)silane (3cc)

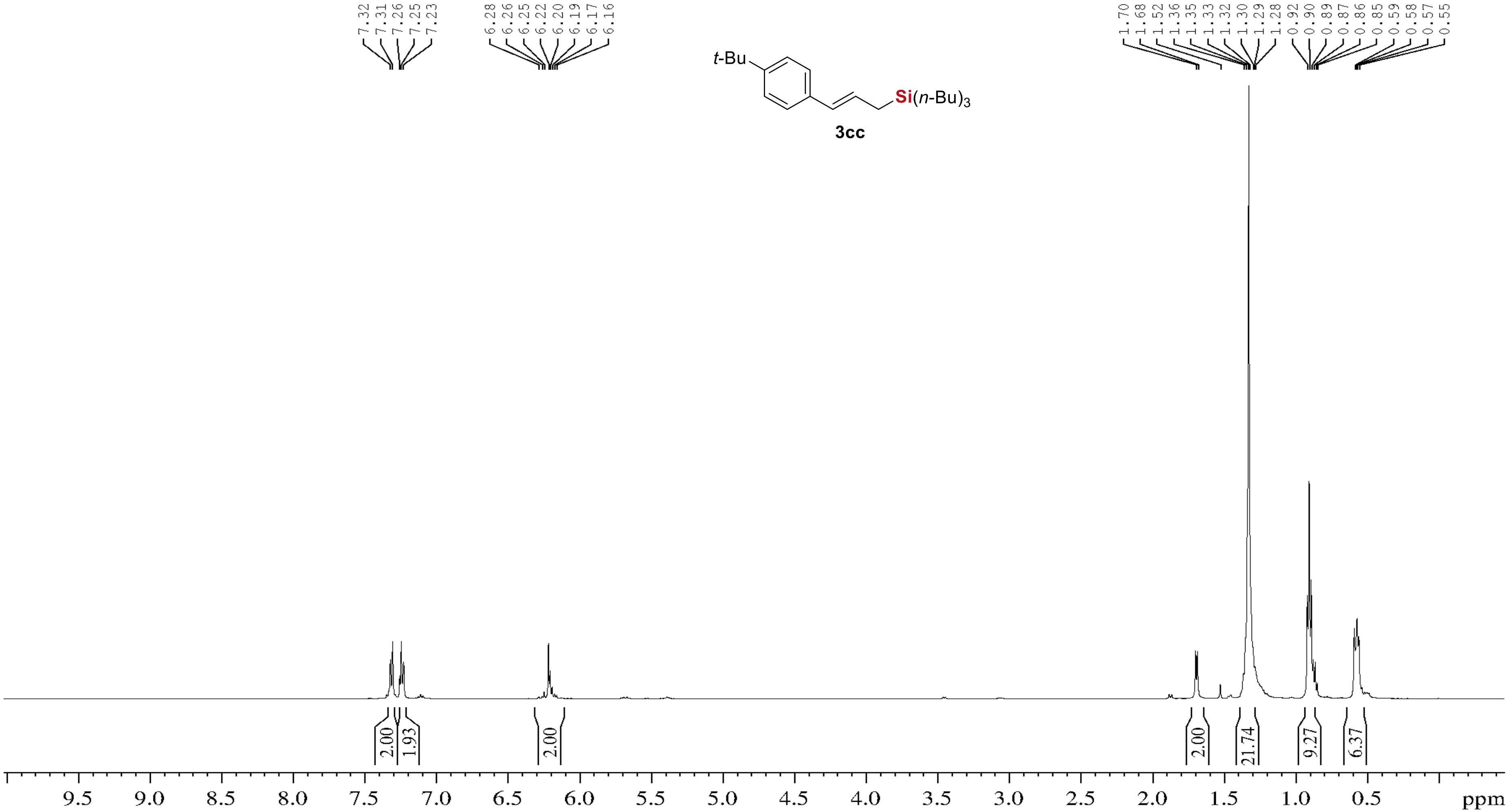

Figure S99. $^{13}C\{^{1}H\}$ NMR spectrum (126 MHz, $CDCl_3$, 298 K) of (*E*)-tributyl(3-(4-(*tert*-butyl)phenyl)allyl)silane (3cc)

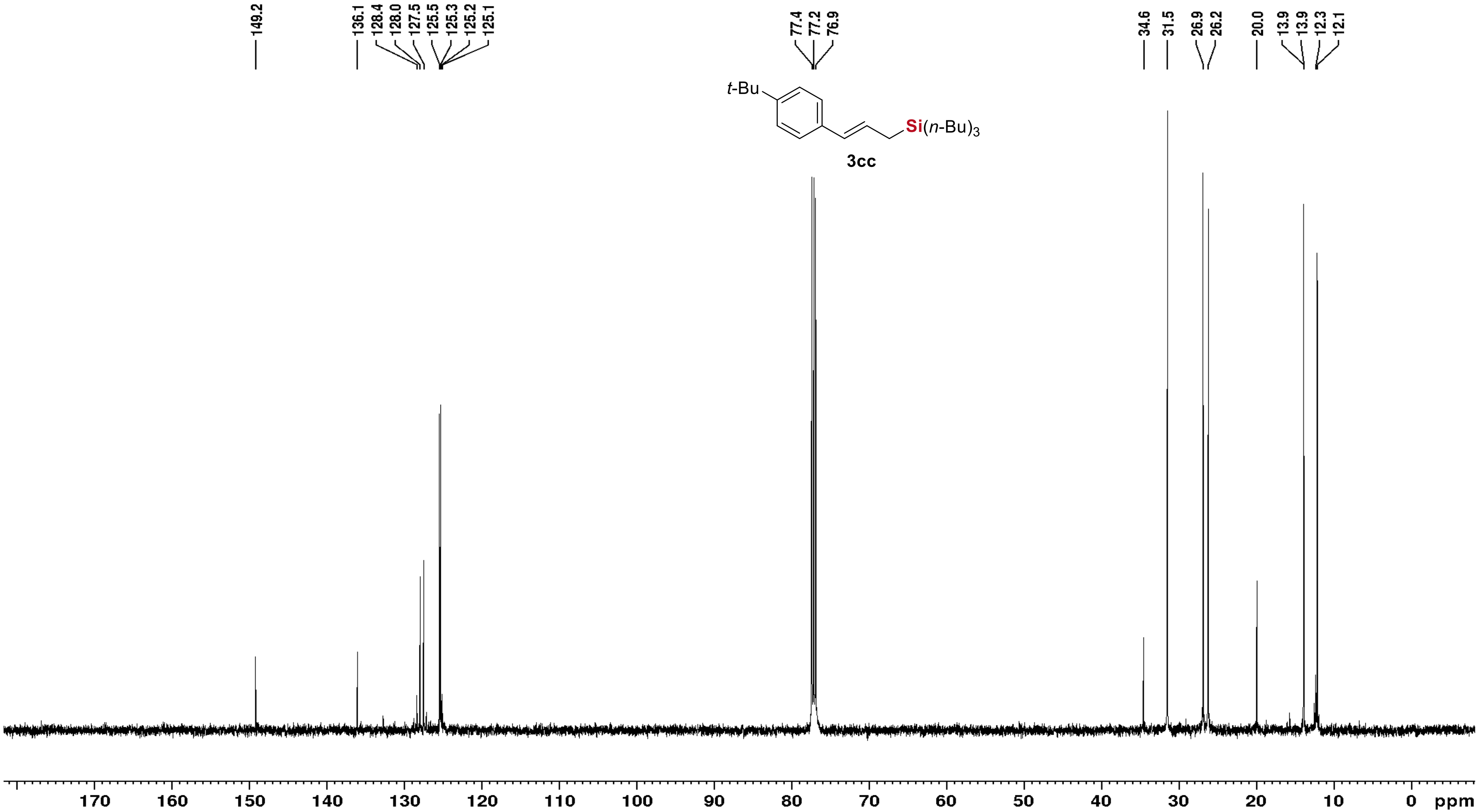

Figure S100. $^{1}H/^{29}Si$ **HMQC NMR spectrum (500/99 MHz,** $CDCl_3$**, 298 K, optimized for** J **= 7 Hz) of 3cc**

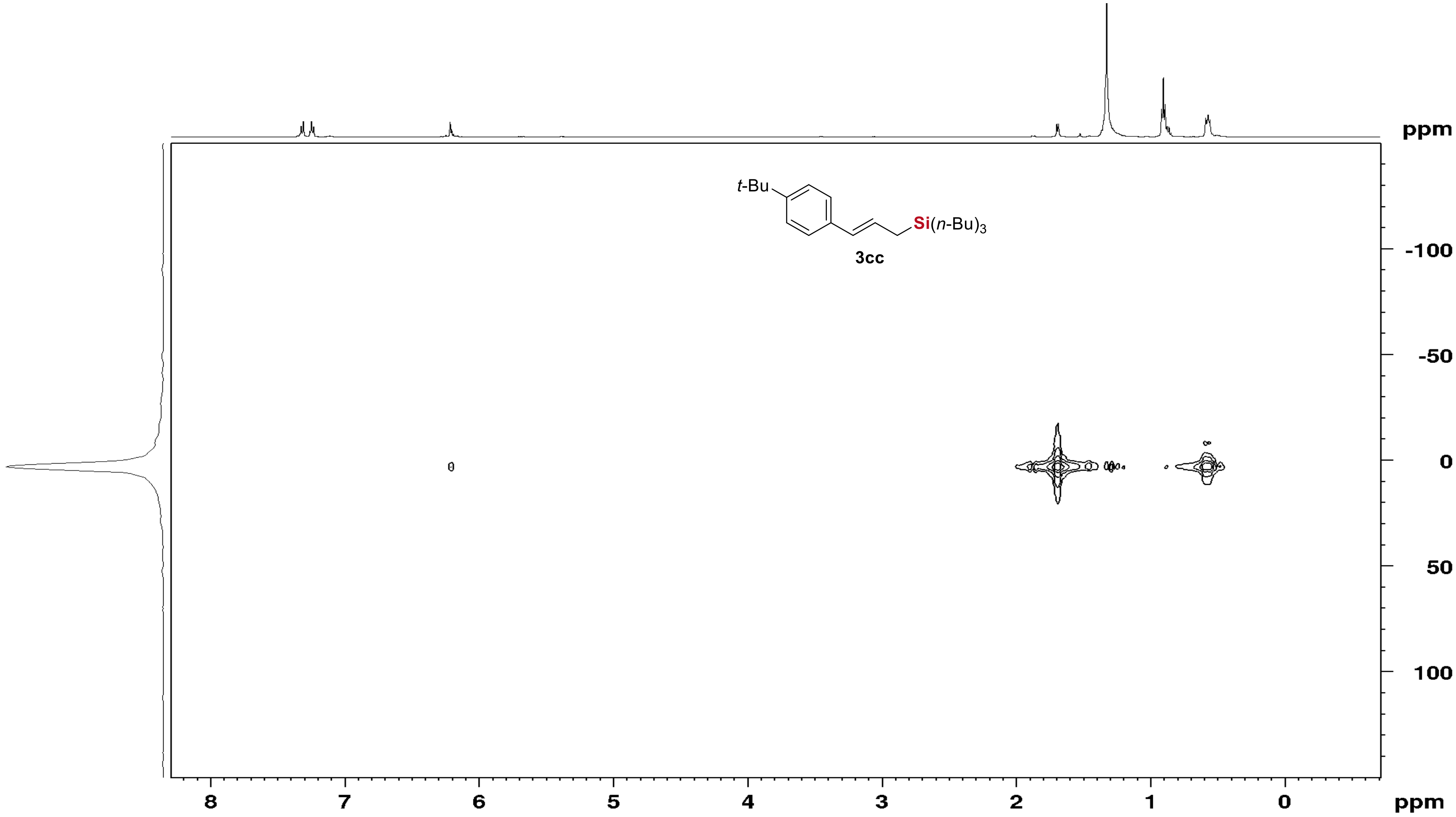

Figure S101. ^1H NMR spectrum (500 MHz, $CDCl_3$, 298 K) of (*E*)-benzyl(cinnamyl)dimethylsilane (3ad)

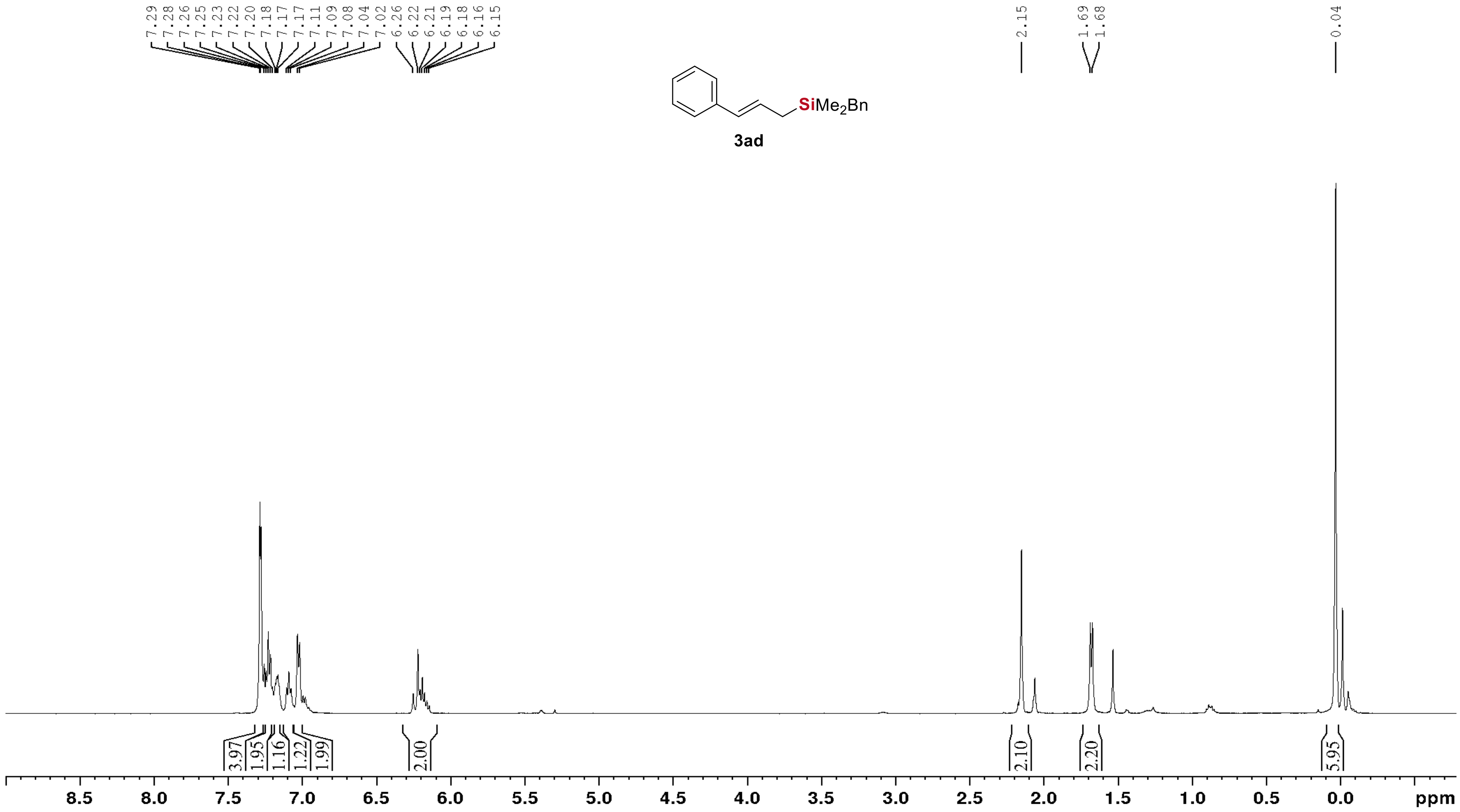

Figure S102. $^{13}C\{^{1}H\}$ NMR spectrum (126 MHz, $CDCl_3$, 298 K) of (*E*)- cinnamyl(methyl)diphenylsilane (3ad)

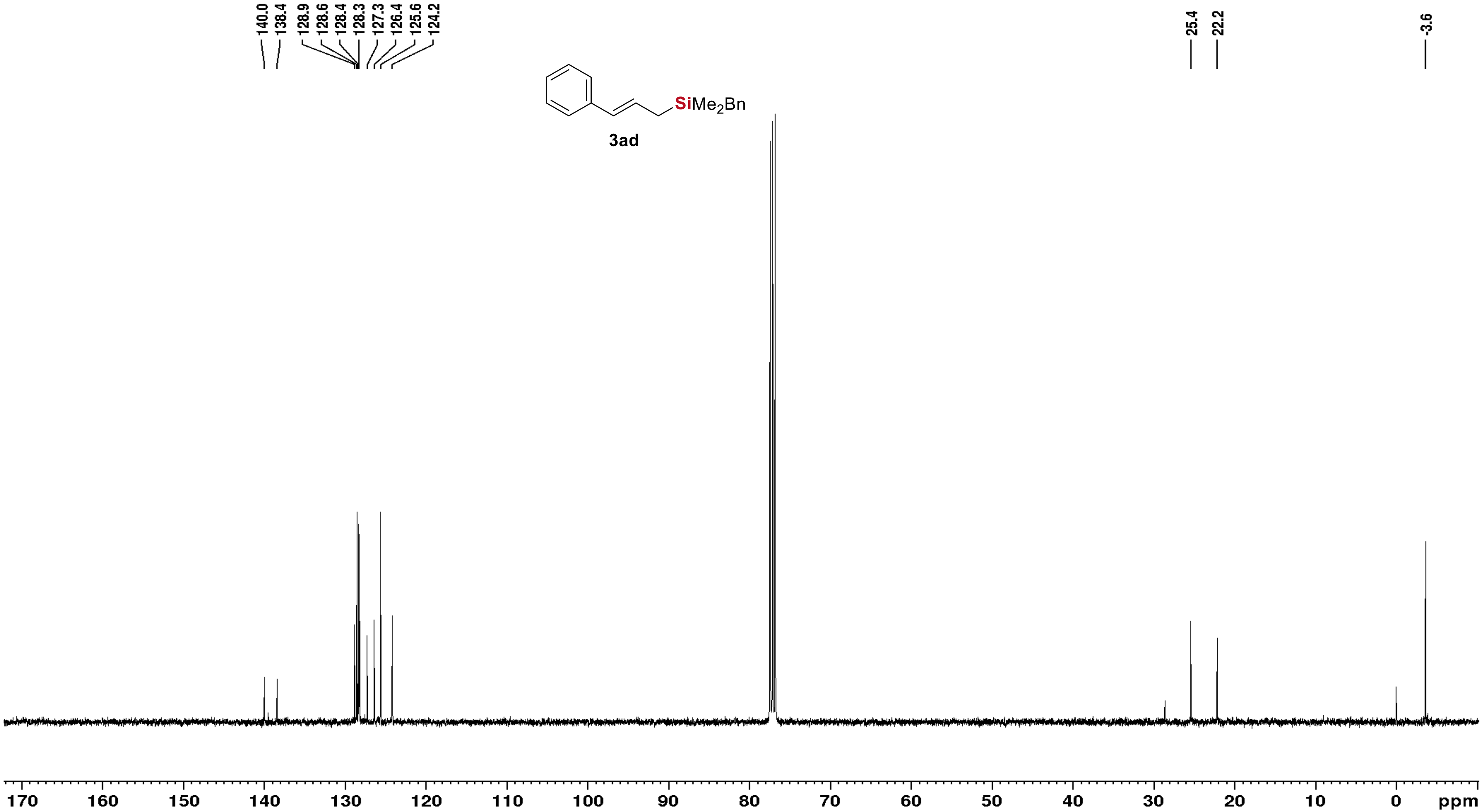

Figure S103. $^{1}H/^{29}Si$ **HMQC NMR spectrum (500/99 MHz, $CDCl_3$, 298 K, optimized for J = 7 Hz) of 3ad**

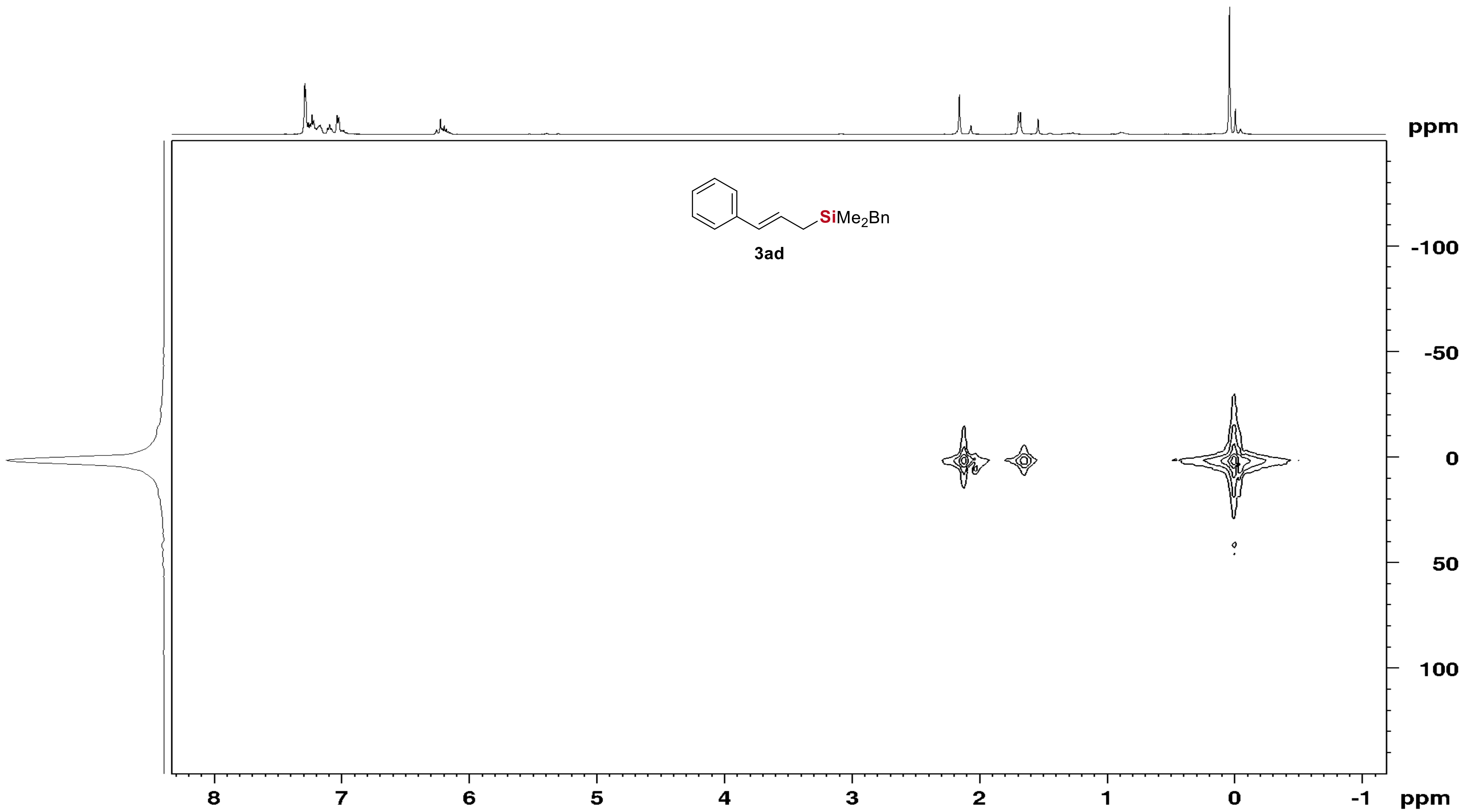

**Figure S104. **

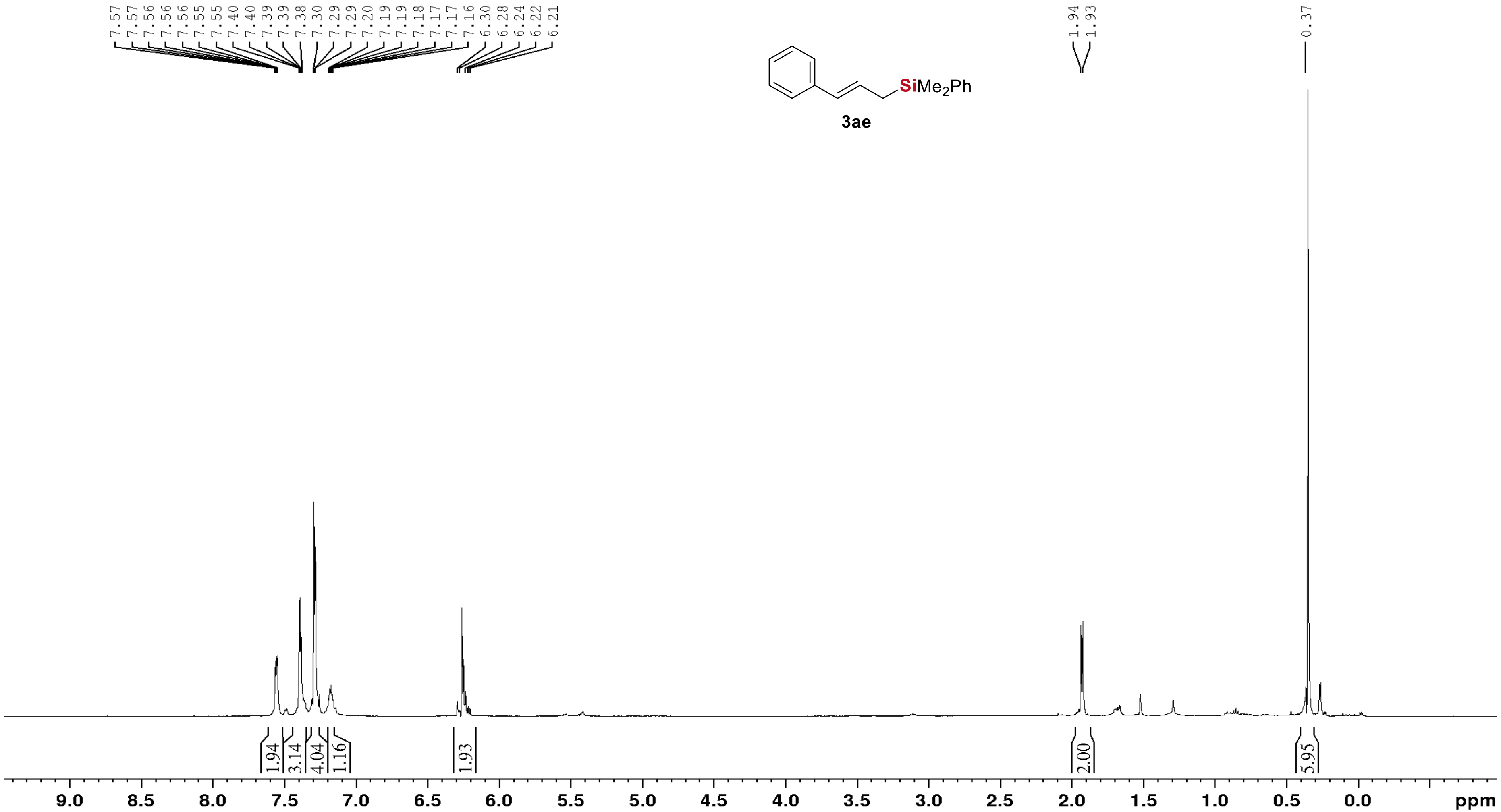

Figure S105. $^{13}C\{^1H\}$ **NMR spectrum (126 MHz, CDCl$_3$, 298 K) of (*E*)-cinnamyldimethyl(phenyl)silane (3ae)**

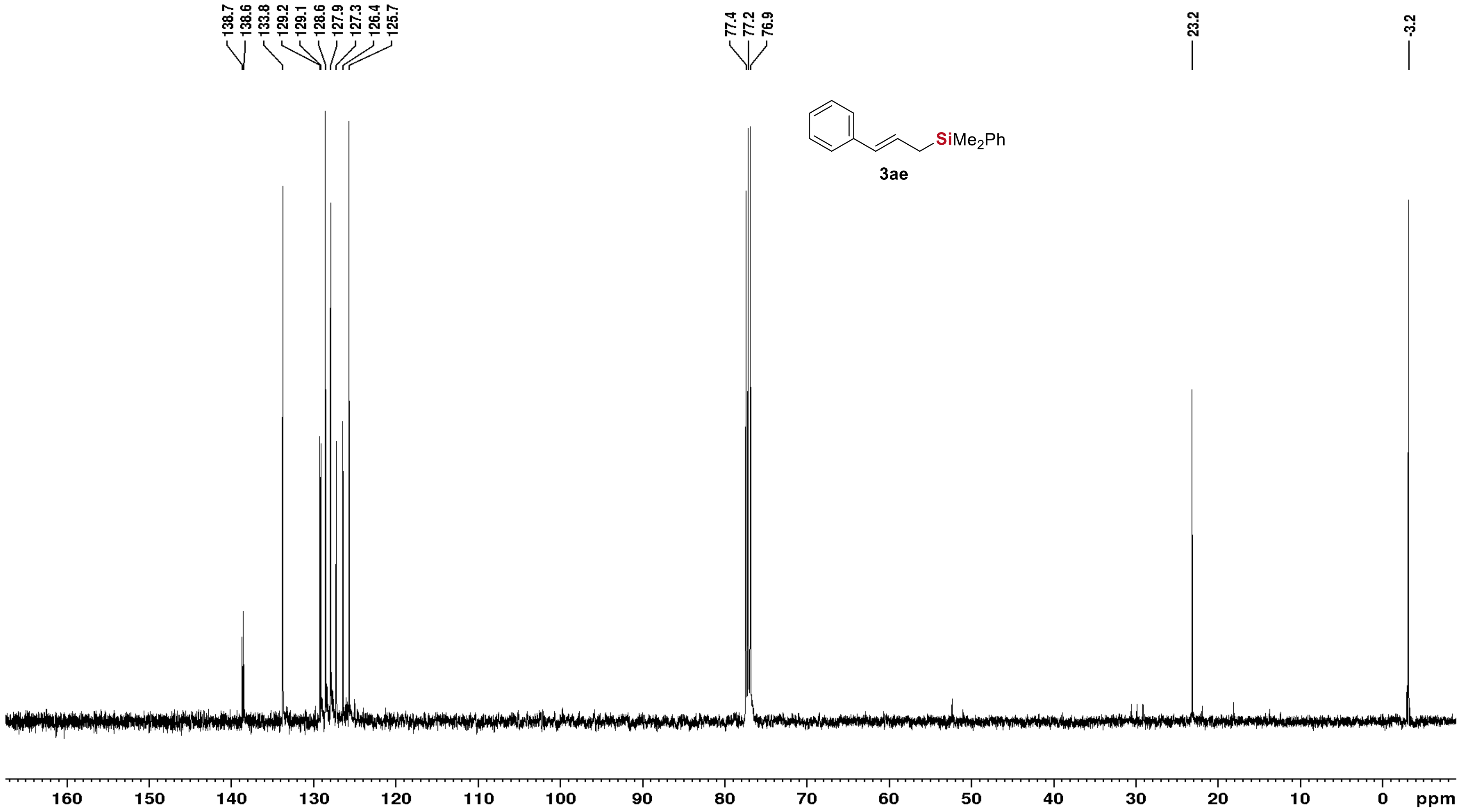

Figure S106. **$^{1}H/^{29}Si$ HMQC NMR spectrum (500/99 MHz, $CDCl_3$, 298 K, optimized for *J* = 7 Hz) of 3ae**

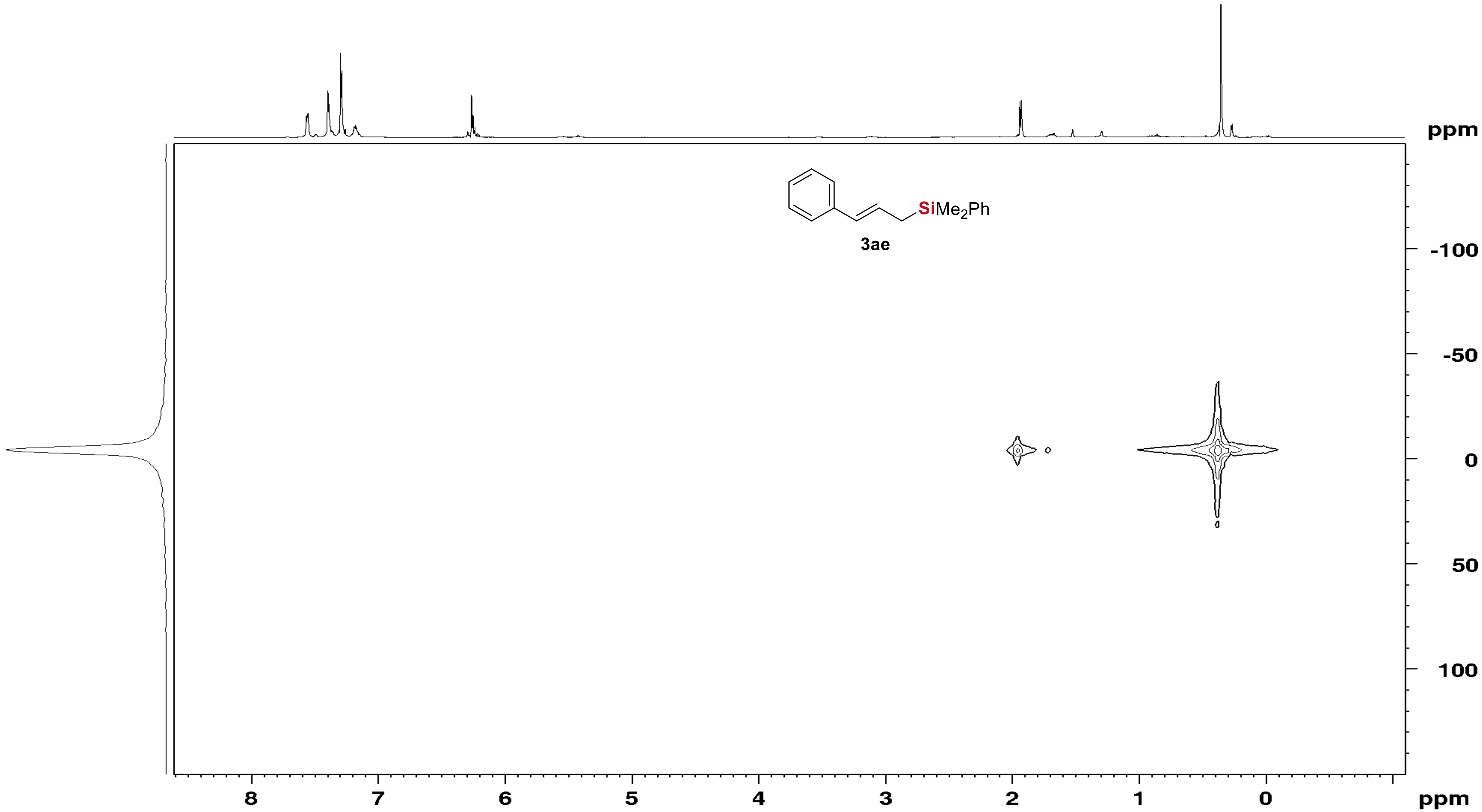

Figure S107. ¹H NMR spectrum (500 MHz, $CDCl_3$, 298 K) of cinnamyl(methyl)diphenylsilane (3af)

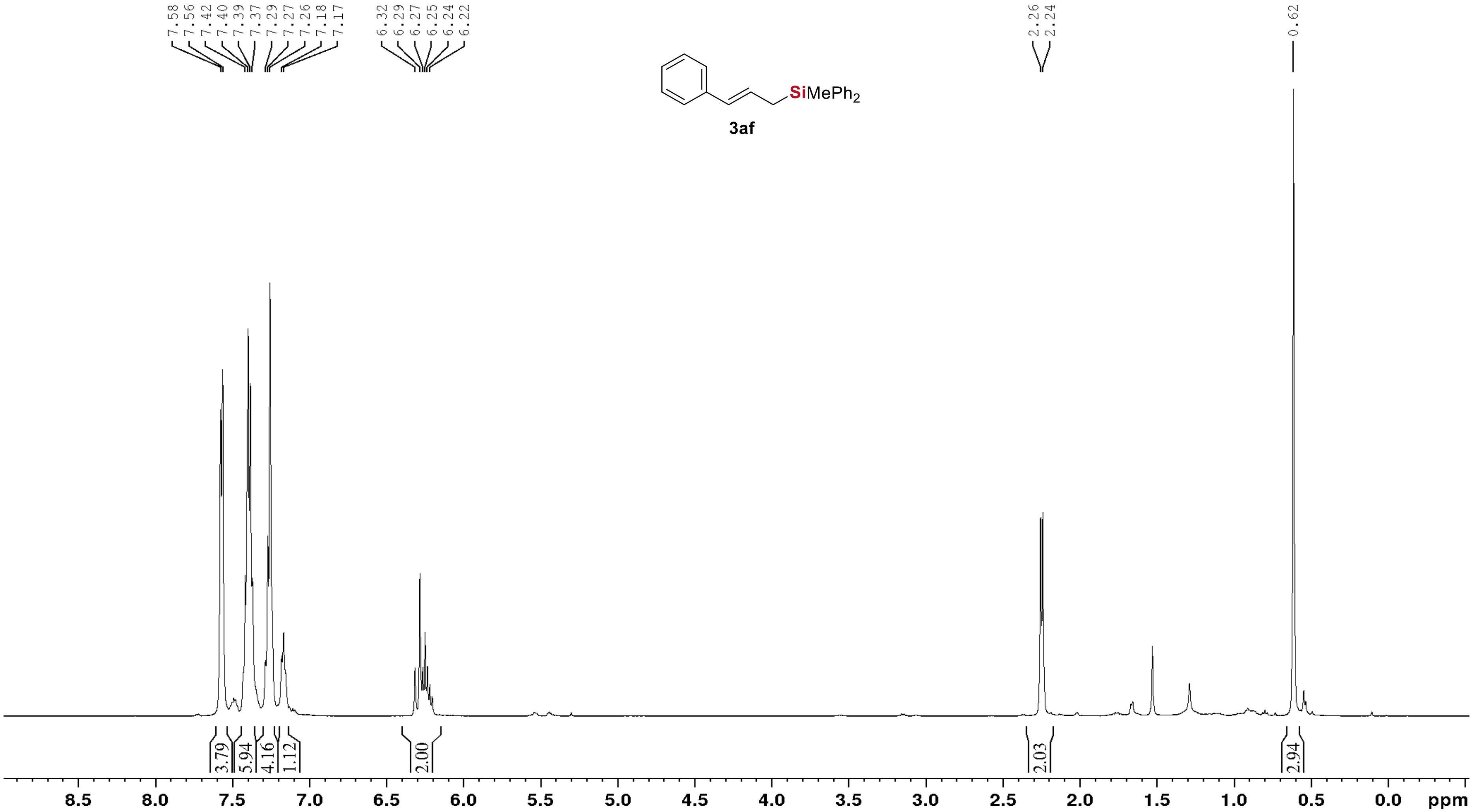

Figure S108. $^{13}C\{^1H\}$ **NMR spectrum (126 MHz, $CDCl_3$, 298 K) of (*E*)-cinnamyl(methyl)diphenylsilane (3af)**

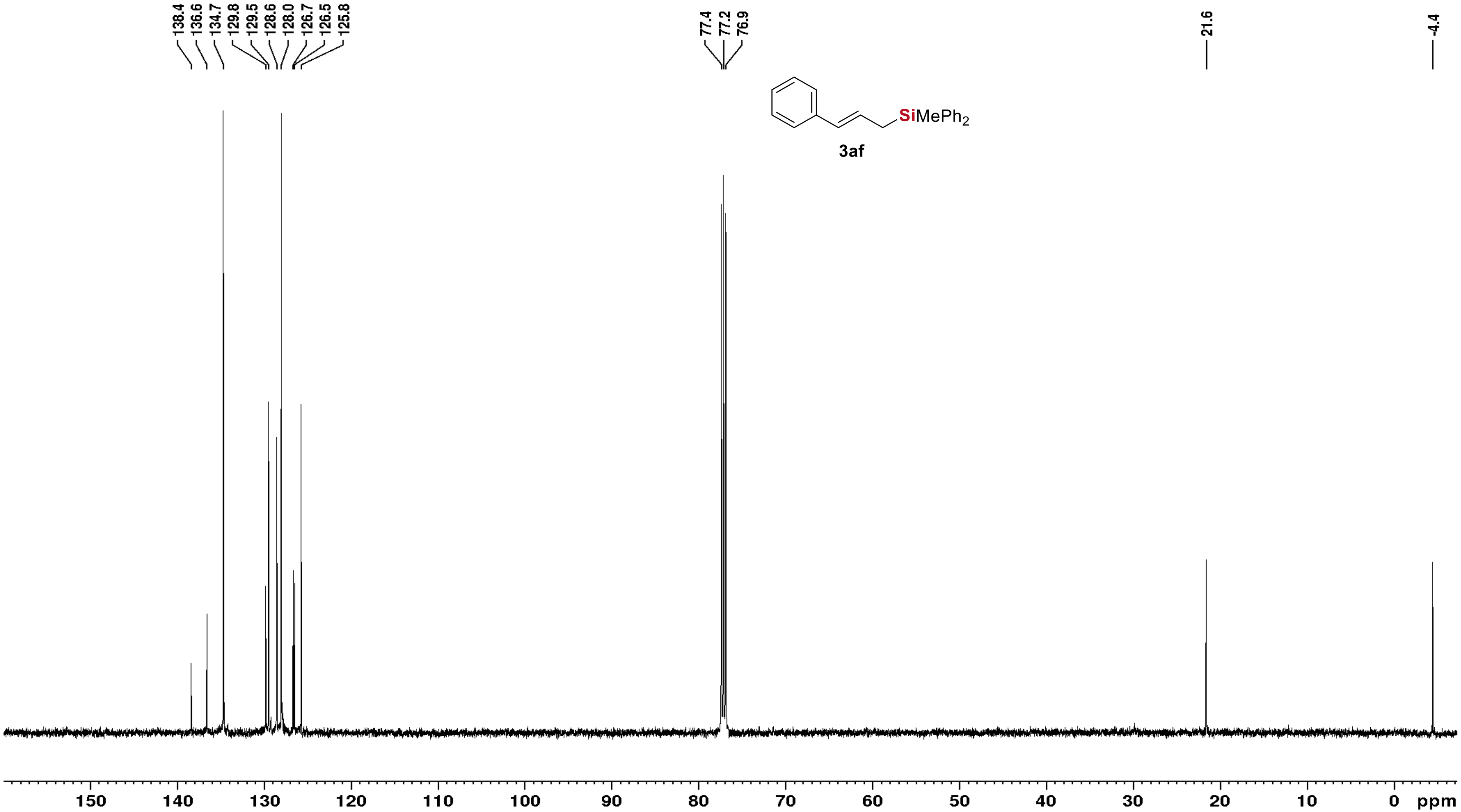

Figure S109. $^{1}H/^{29}Si$ **HMQC NMR spectrum (500/99 MHz, $CDCl_3$, 298 K, optimized for *J* = 7 Hz) of 3af**

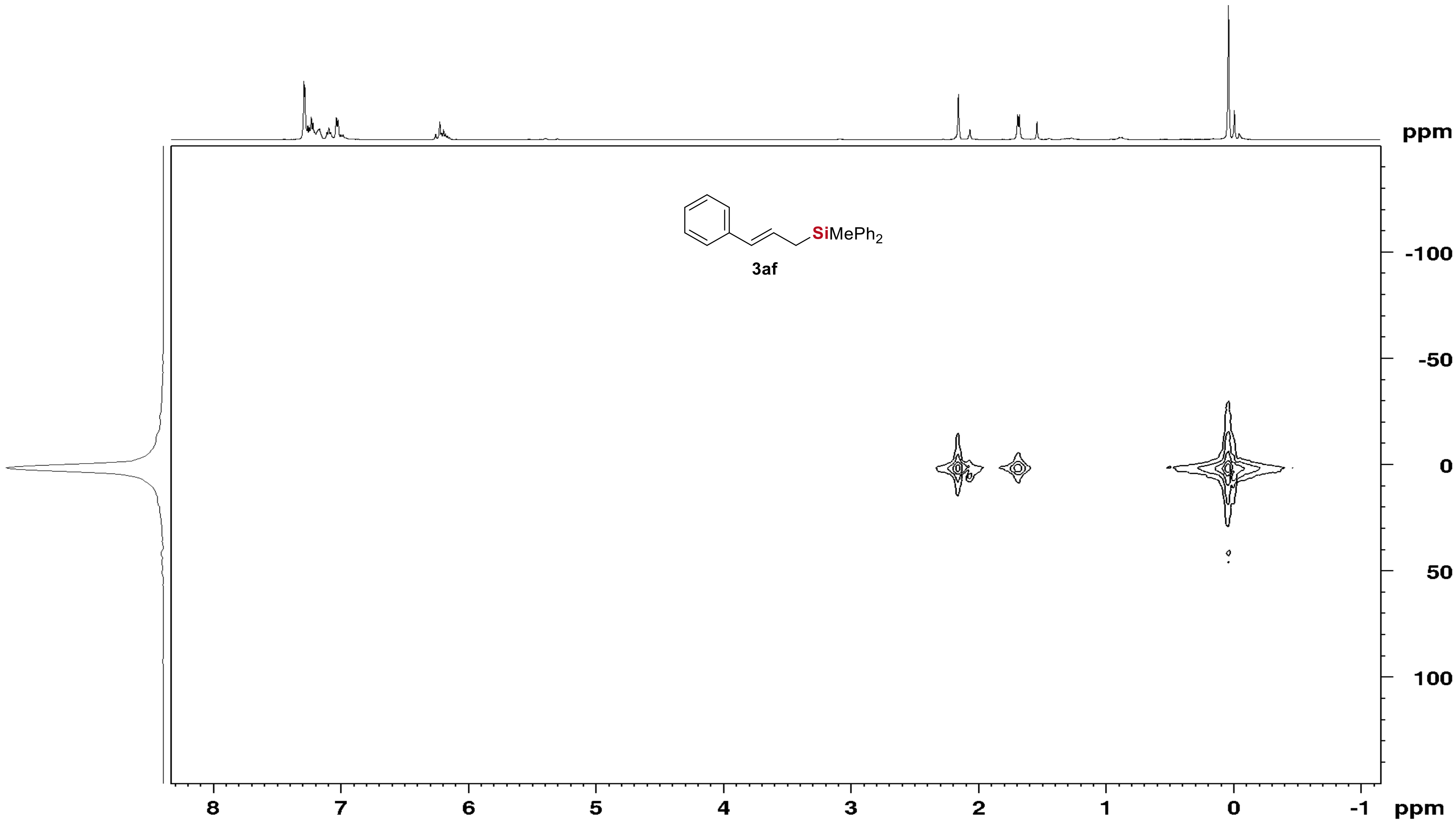

Figure S110. ^{1}H NMR spectrum (500 MHz, $CDCl_3$, 298 K) of (*E, E*)-dicinnamyldimethylsilane (3ah)

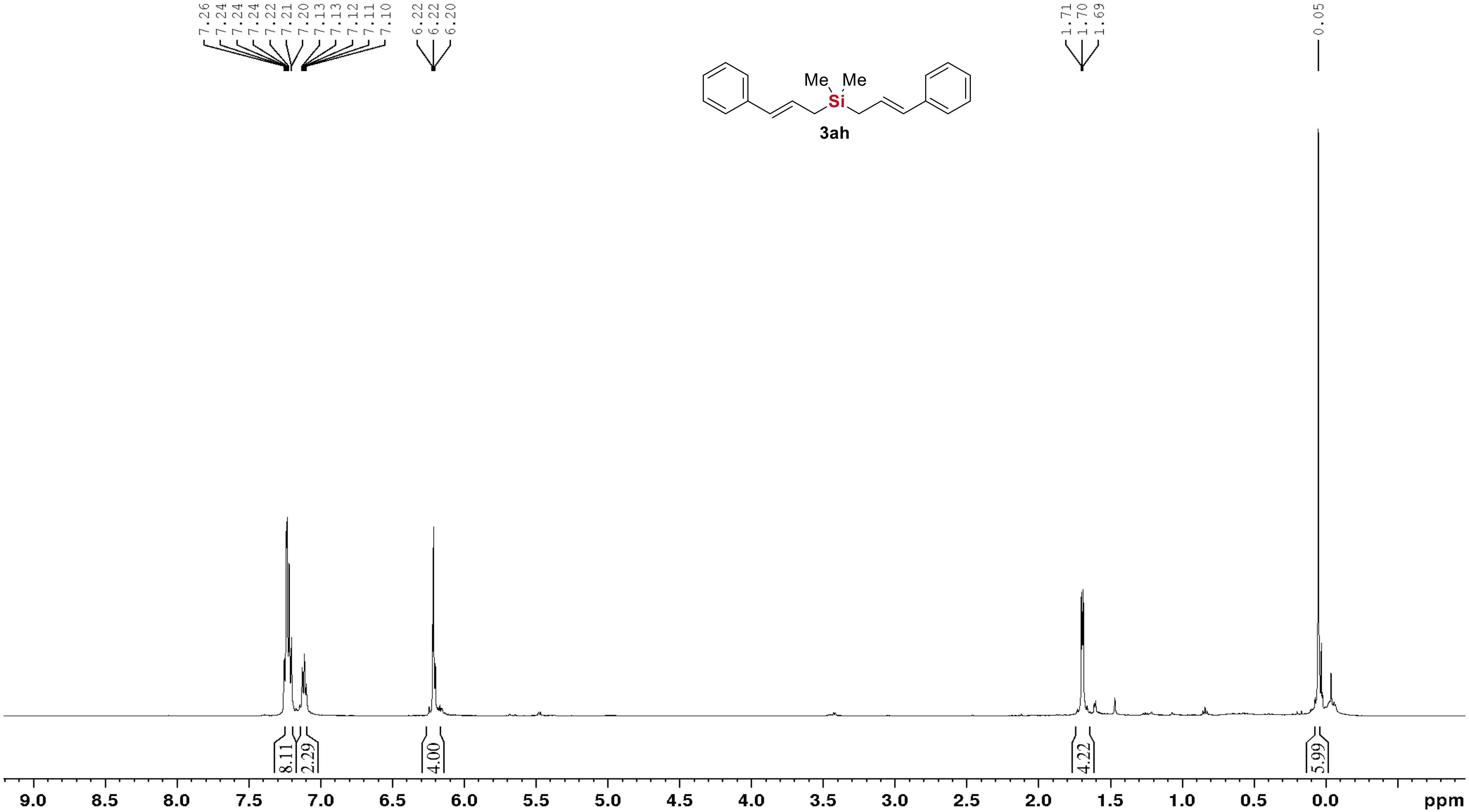

Figure S111. $^{13}C\{^1H\}$ **NMR spectrum (126 MHz, $CDCl_3$, 298 K) of (*E, E*)-dicinnamyldimethylsilane (3ah)**

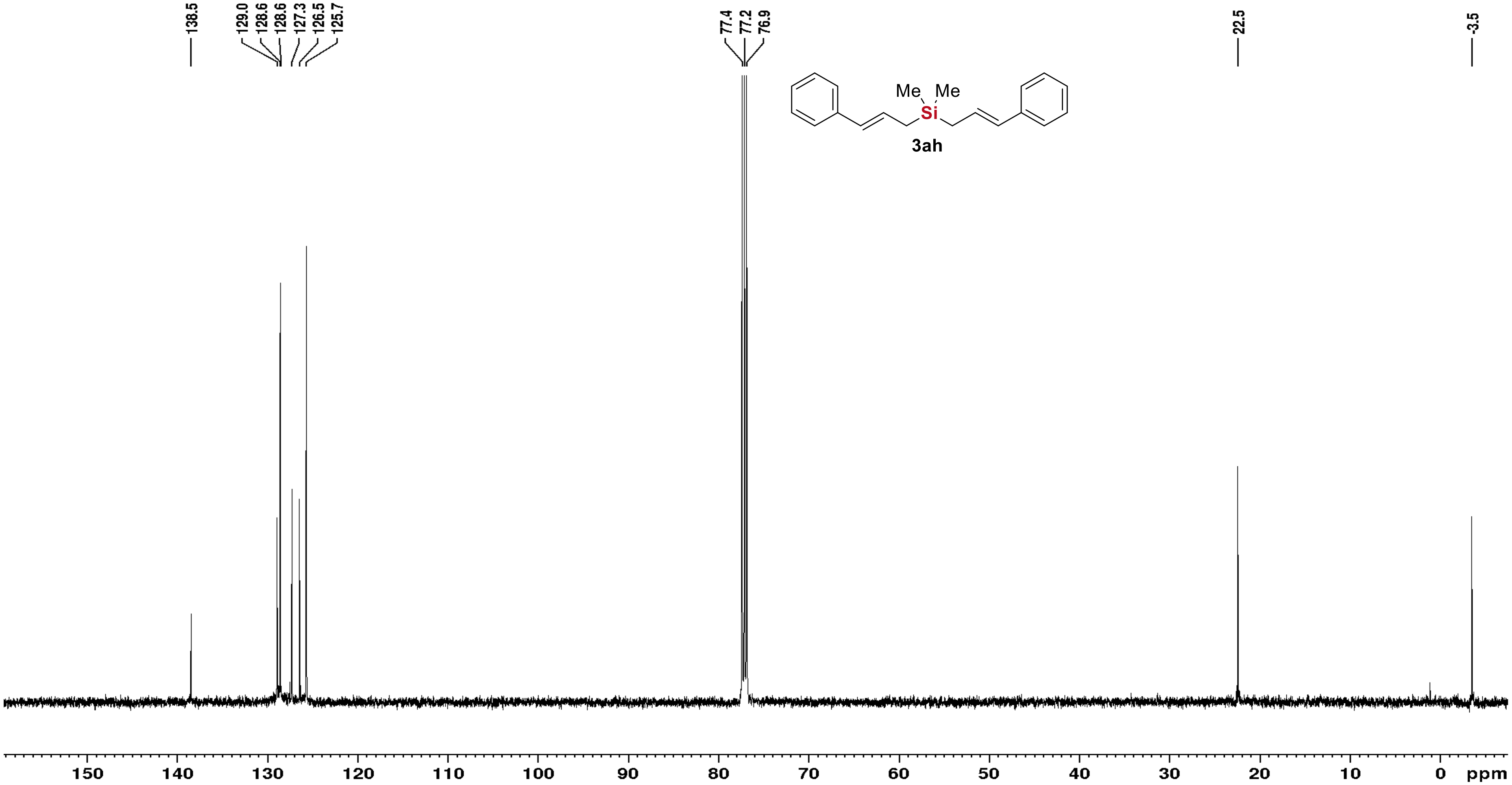

Figure S112. **^{1}H/^{29}Si HMQC NMR spectrum (500/99 MHz, $CDCl_3$, 298 K, optimized for *J* = 7 Hz) of 3ah**

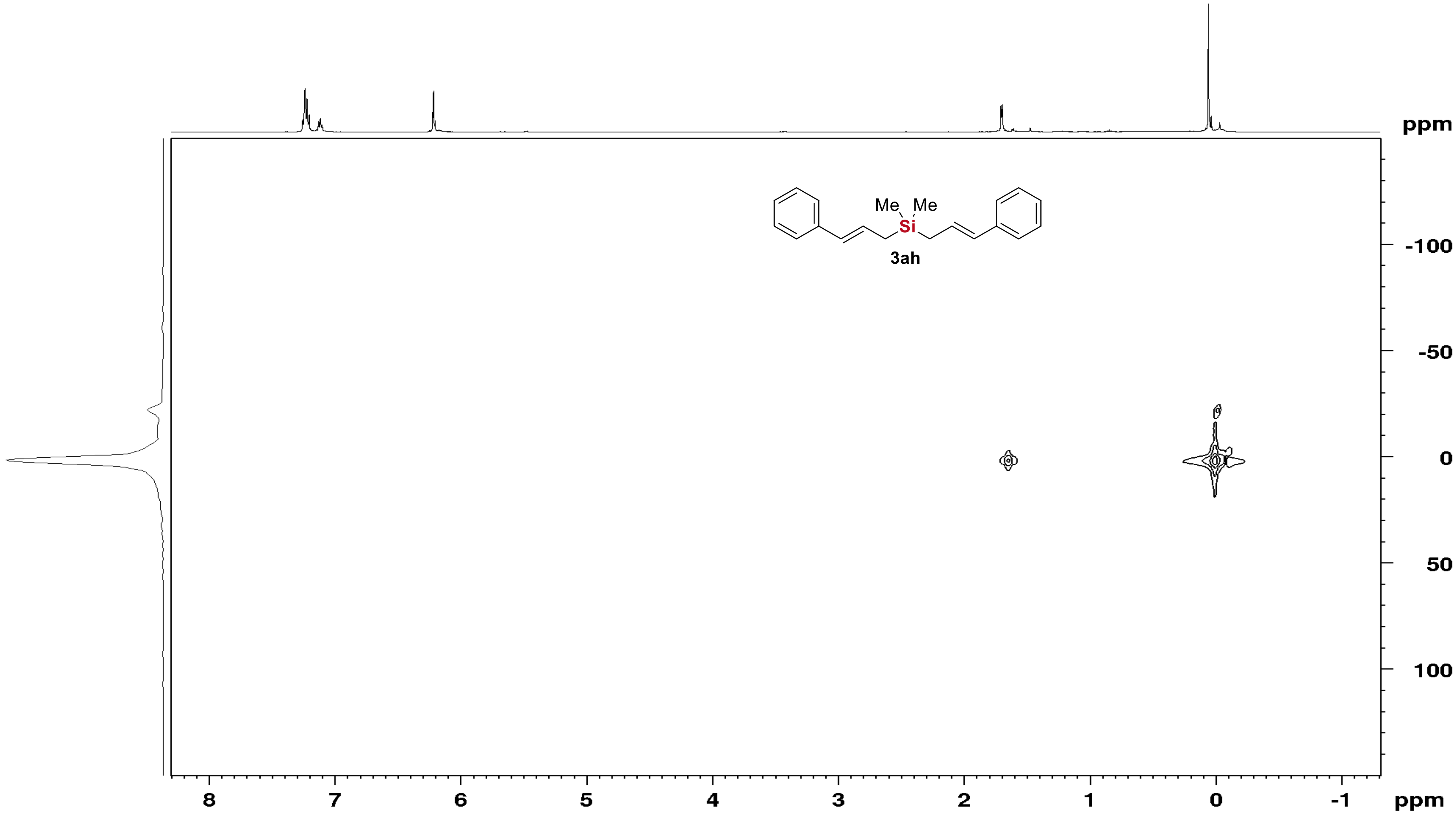

Figure S113. ^{1}H NMR spectrum (500 MHz, C_6D_6, 298 K) of (COD)Pd(CH_2SiMe_3)$_2$

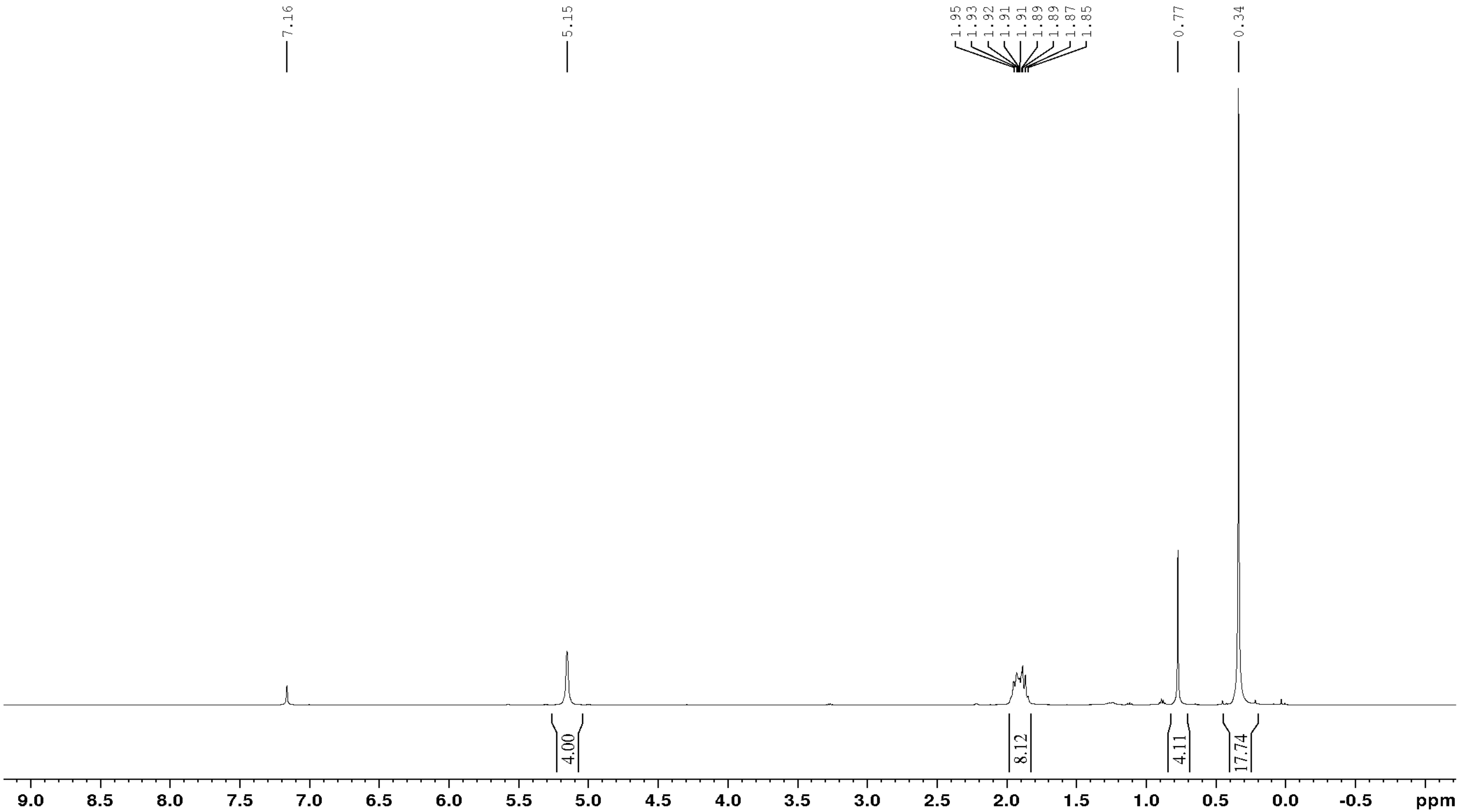

Figure S114. $^{1}H/^{29}Si$ **HMQC NMR spectrum (500/99 MHz,** C_6D_6**, 298 K, optimized for** J **= 7 Hz) of (COD)Pd(**CH_2SiMe_3**)**$_2$

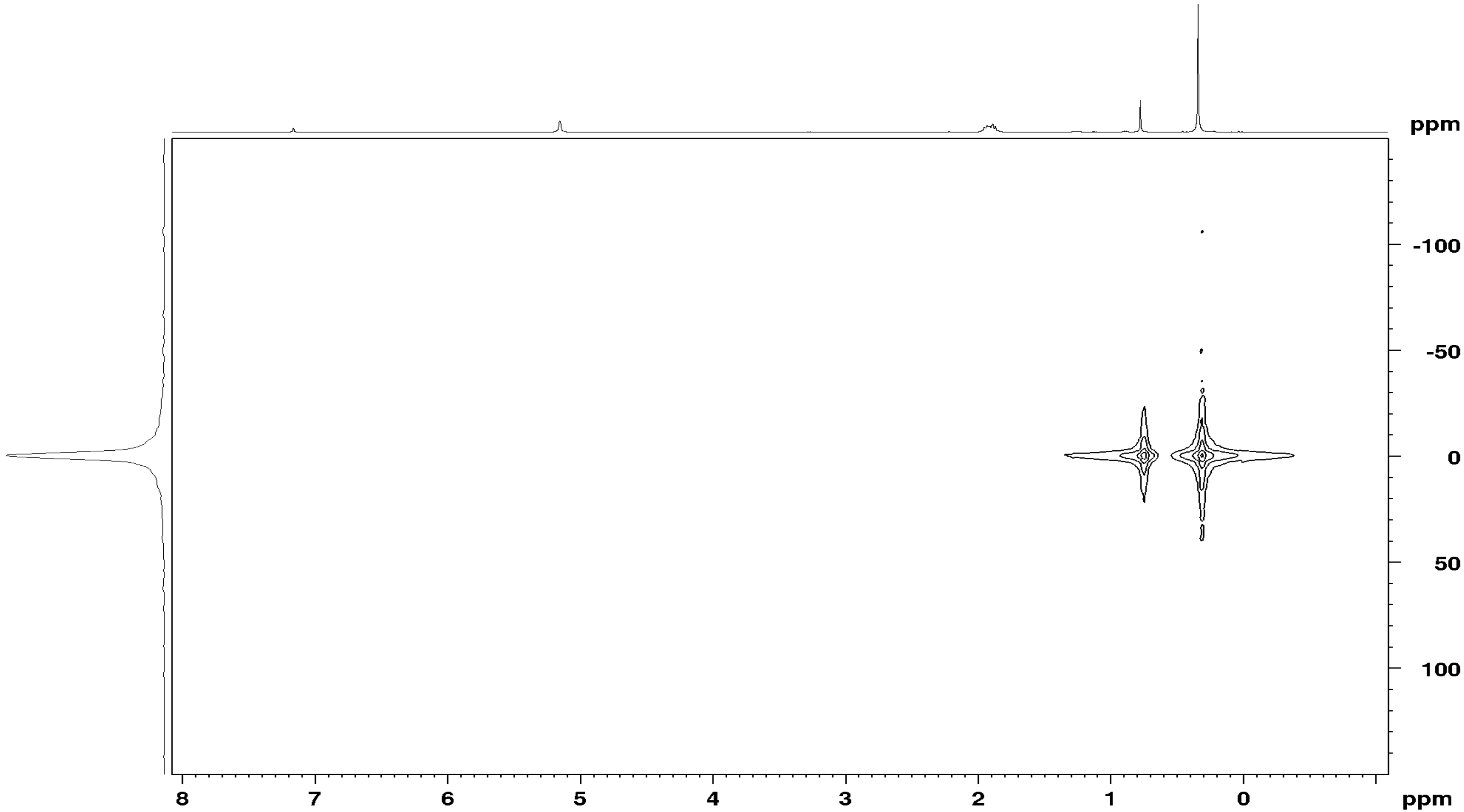

Figure S115. ^{1}H NMR spectrum (500 MHz, CD_2Cl_2, 223 K) of 9b

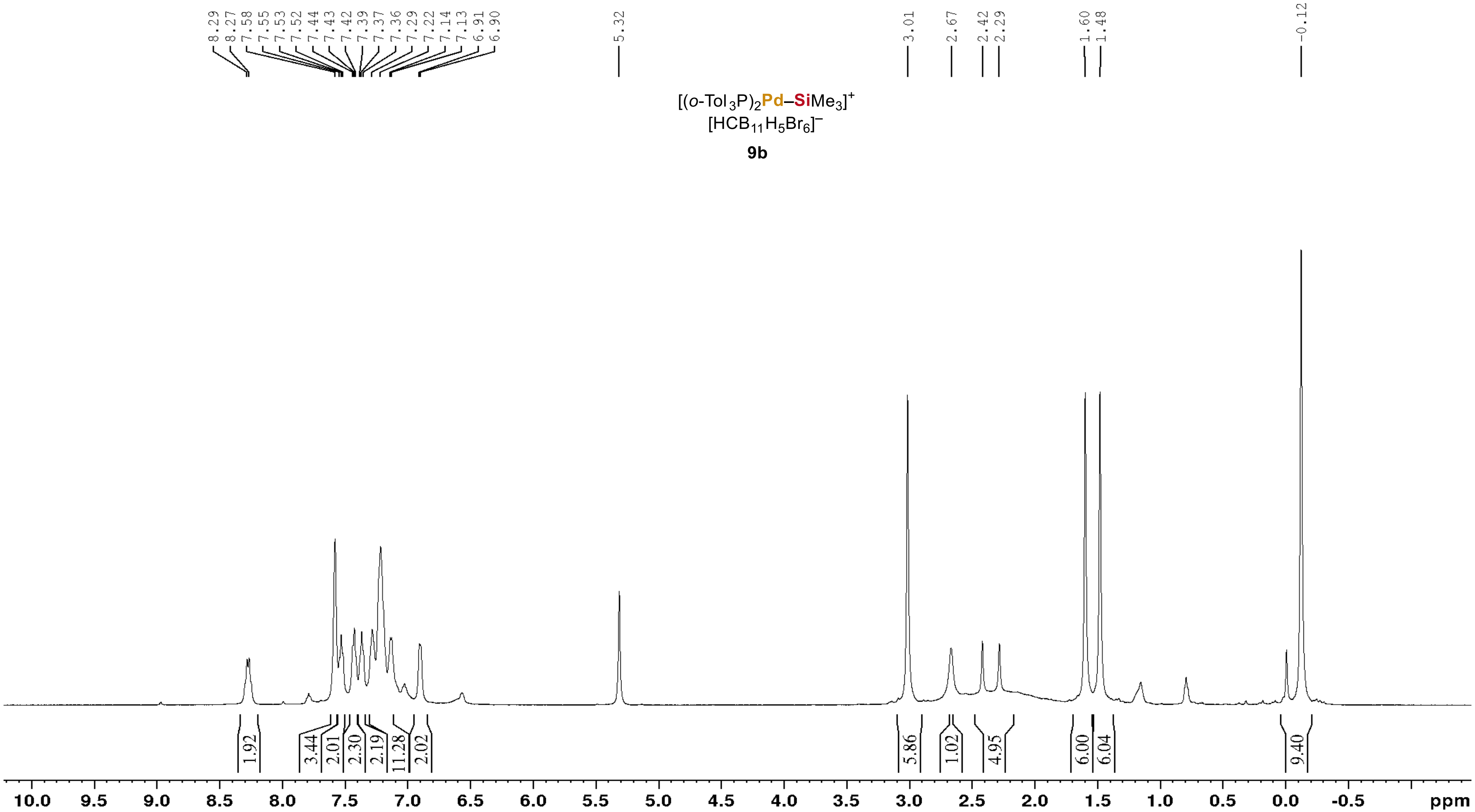

Figure S116. $^{31}P\{^1H\}$ **NMR spectrum (500 MHz, CD_2Cl_2, 223 K) of 9b**

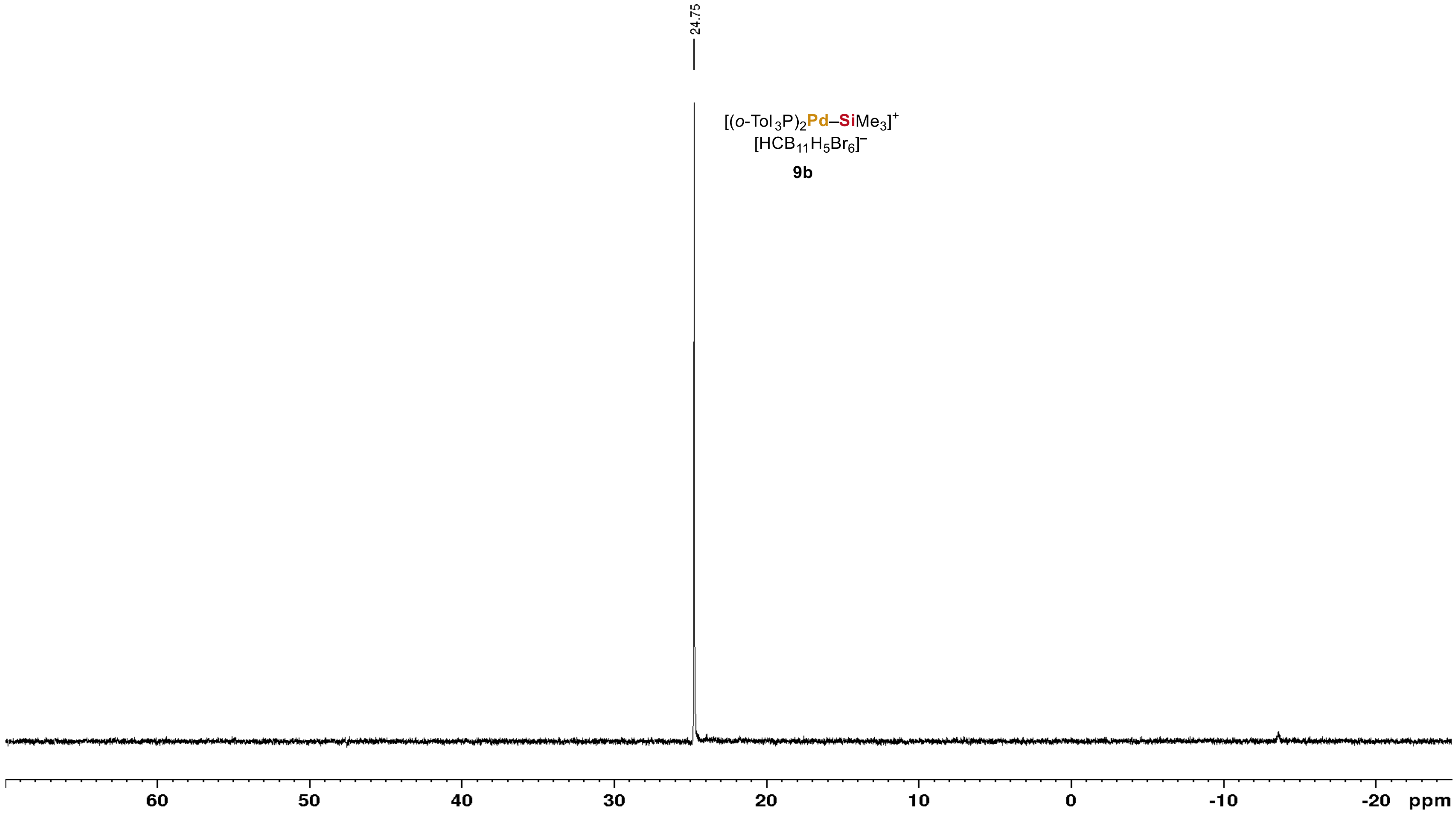

Figure S117. $^{1}H/^{31}P$ HMQC NMR spectrum (500 MHz, CD_2Cl_2, 223 K, optimized for *J* = 7 Hz, NUS 50%) of 9b

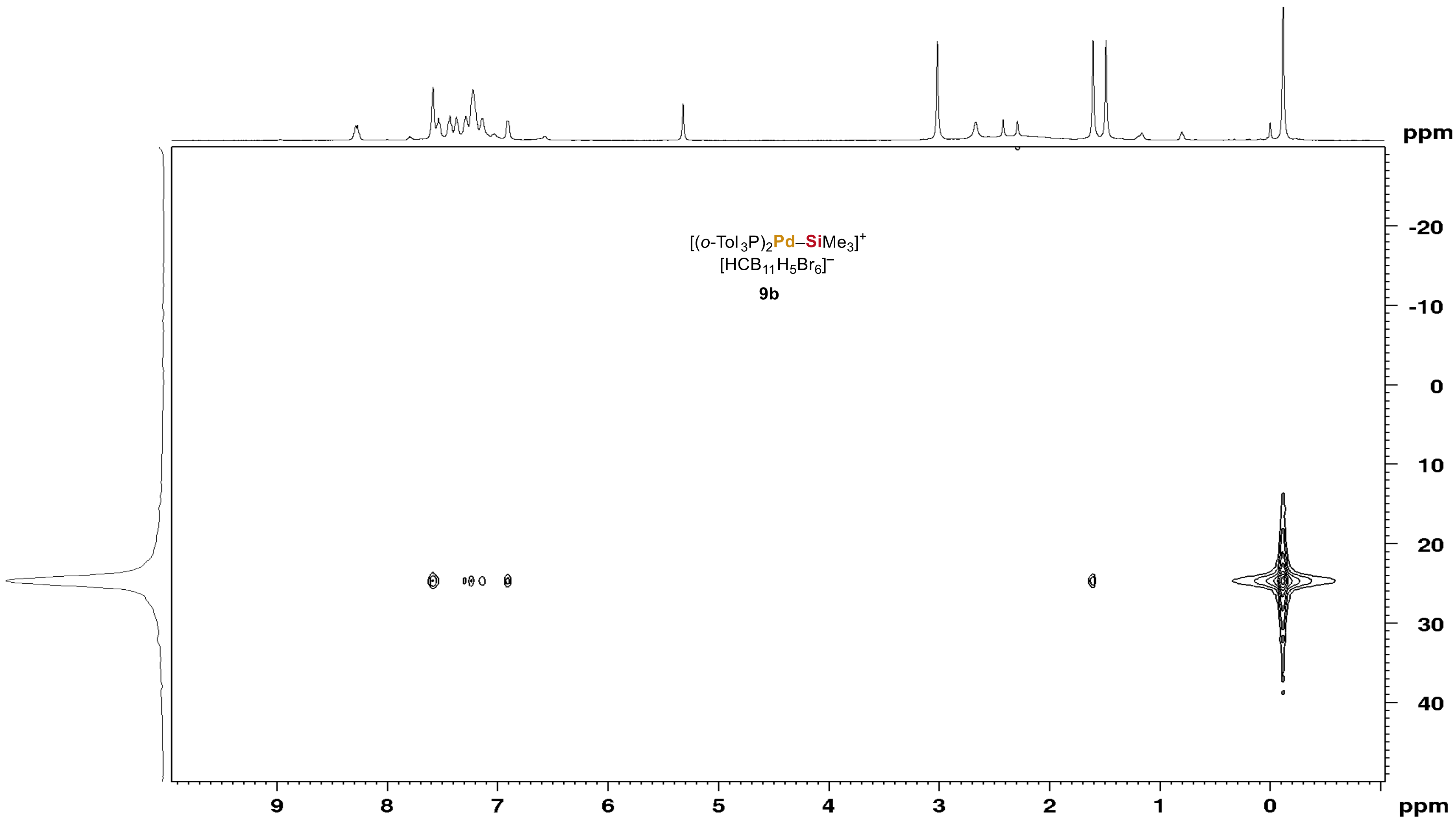

Figure S118. **$^{1}H/^{29}Si$ HMQC NMR spectrum (500/99 MHz, CD_2Cl_2, 223 K, optimized for *J* = 7 Hz) of 9b**

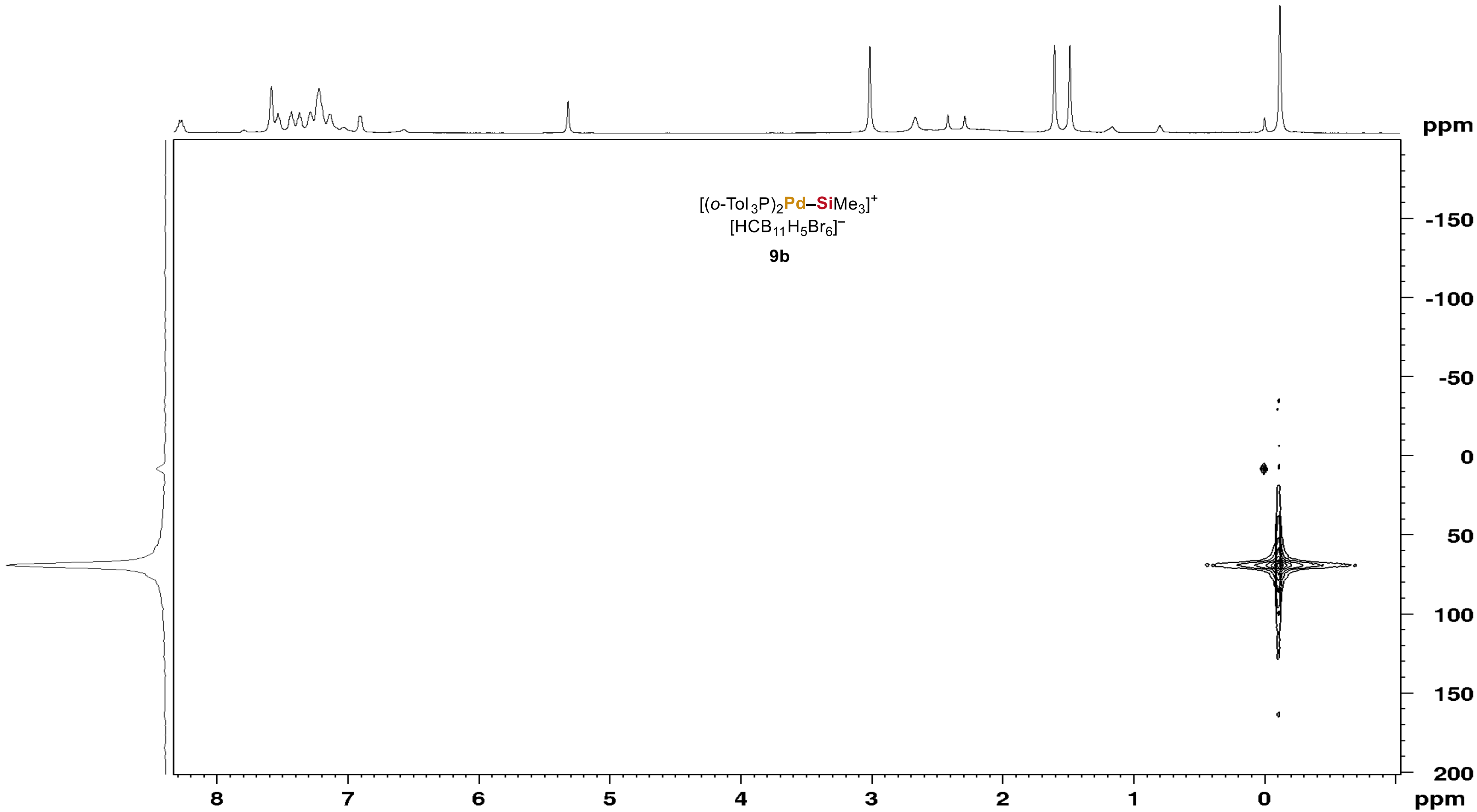

Figure S119. $^{13}C\{^{1}H\}$ **NMR spectrum (126 MHz, CD_2Cl_2, 223 K) of 9b**

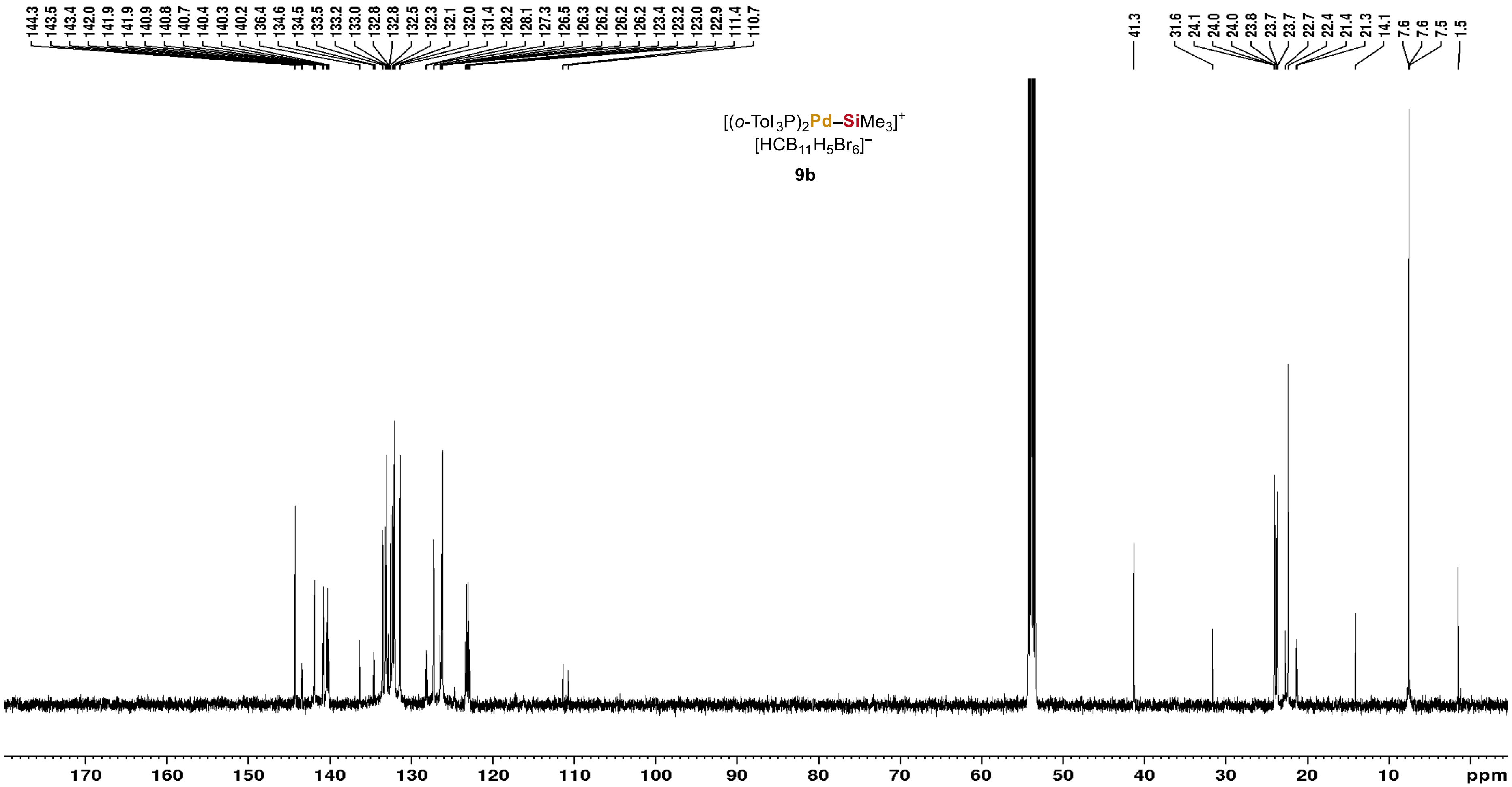

Figure S120. ^{11}B NMR spectrum (160 MHz, CD_2Cl_2, 223 K) of 9b

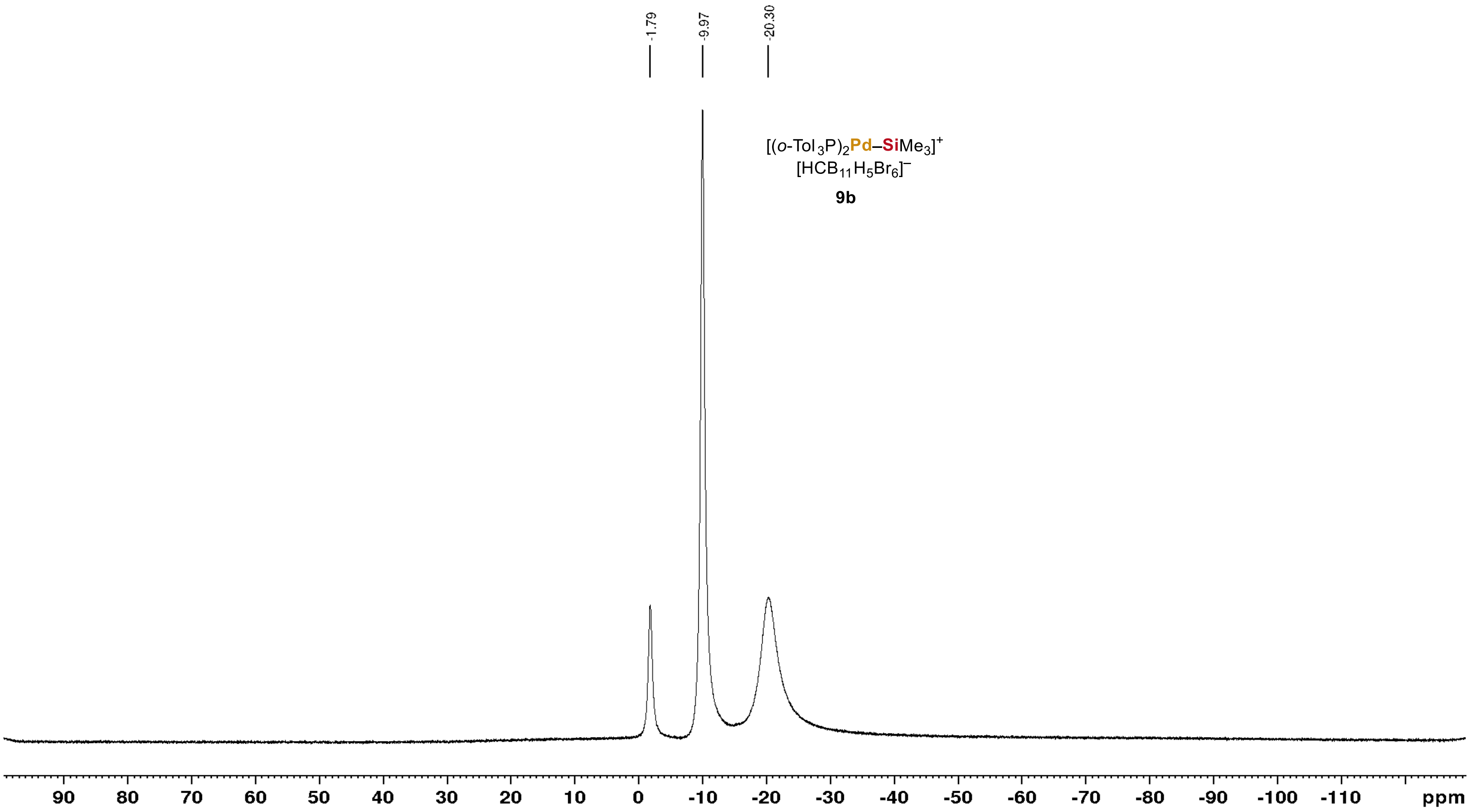

Figure S121. **^{1}H NMR spectrum (500 MHz, 1,2-$C_6D_4Cl_2$, 298 K) of 10b**

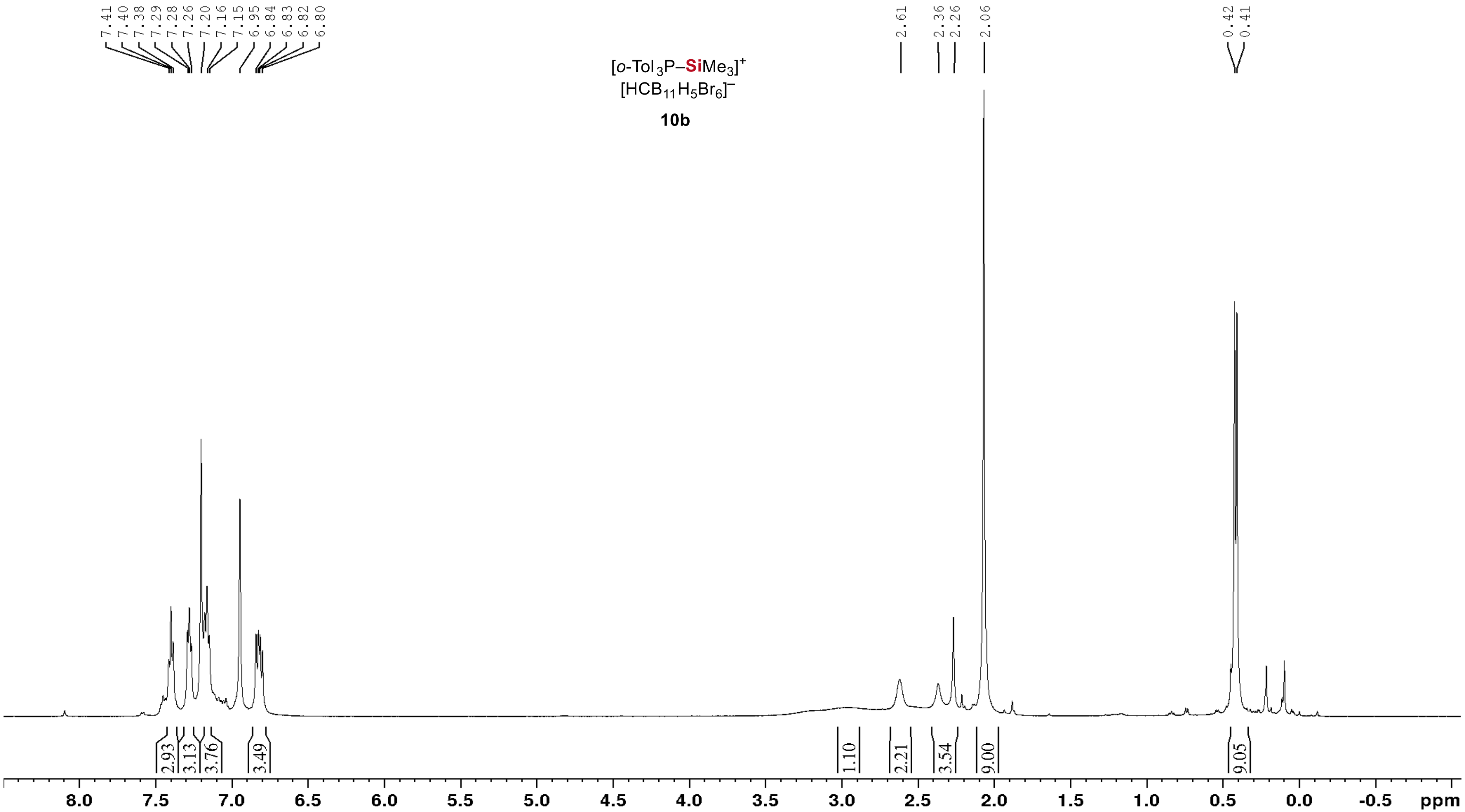

Figure S122. $^{31}P\{^1H\}$ **NMR spectrum (202 MHz, 1,2-** $C_6D_4Cl_2$ **, 298 K) of 10b**

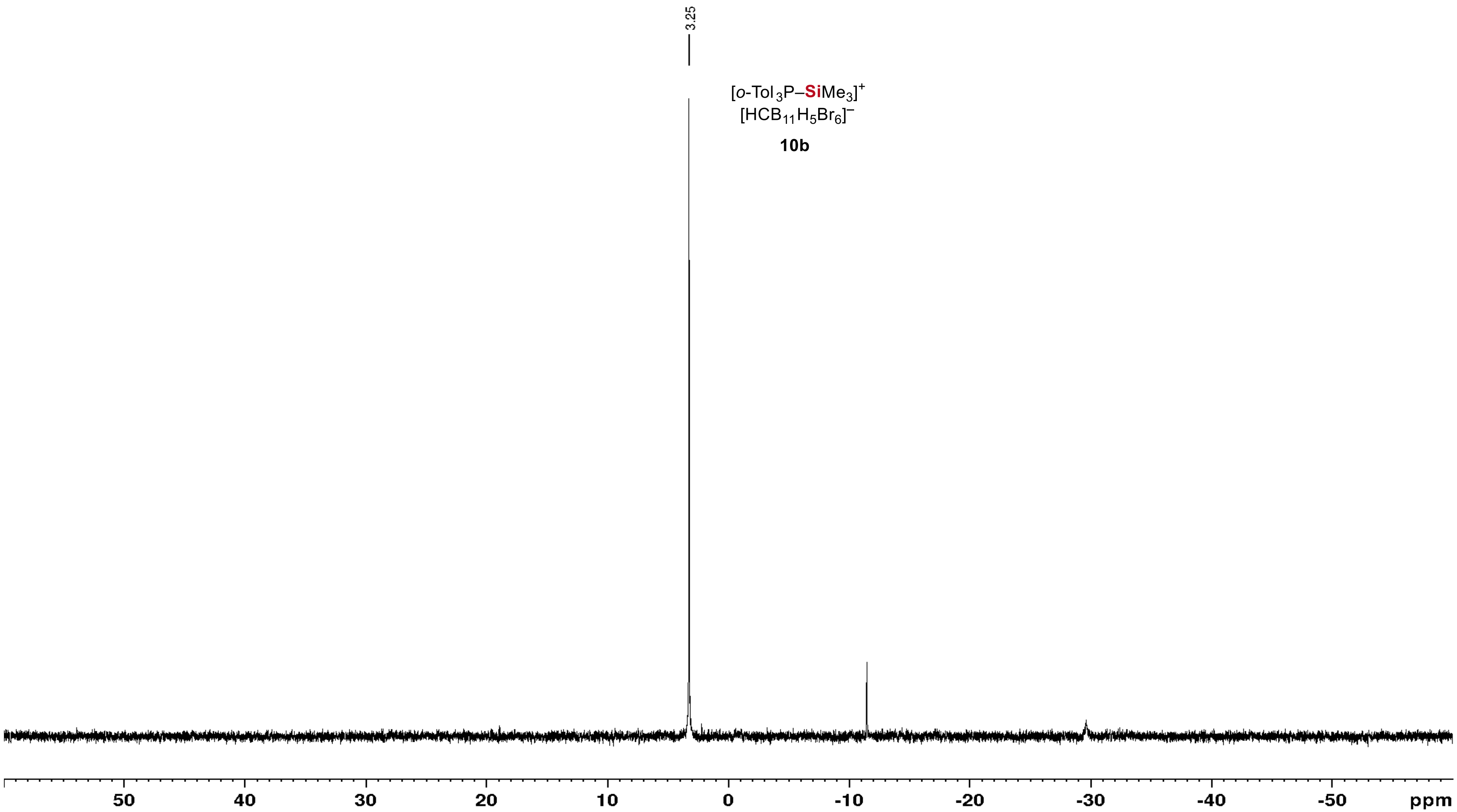

Figure S123. $^{1}H/^{29}Si$ **HMQC NMR spectrum (500/99 MHz, 1,2-$C_6D_4Cl_2$, 298 K, optimized for *J* = 7 Hz) of 10b**

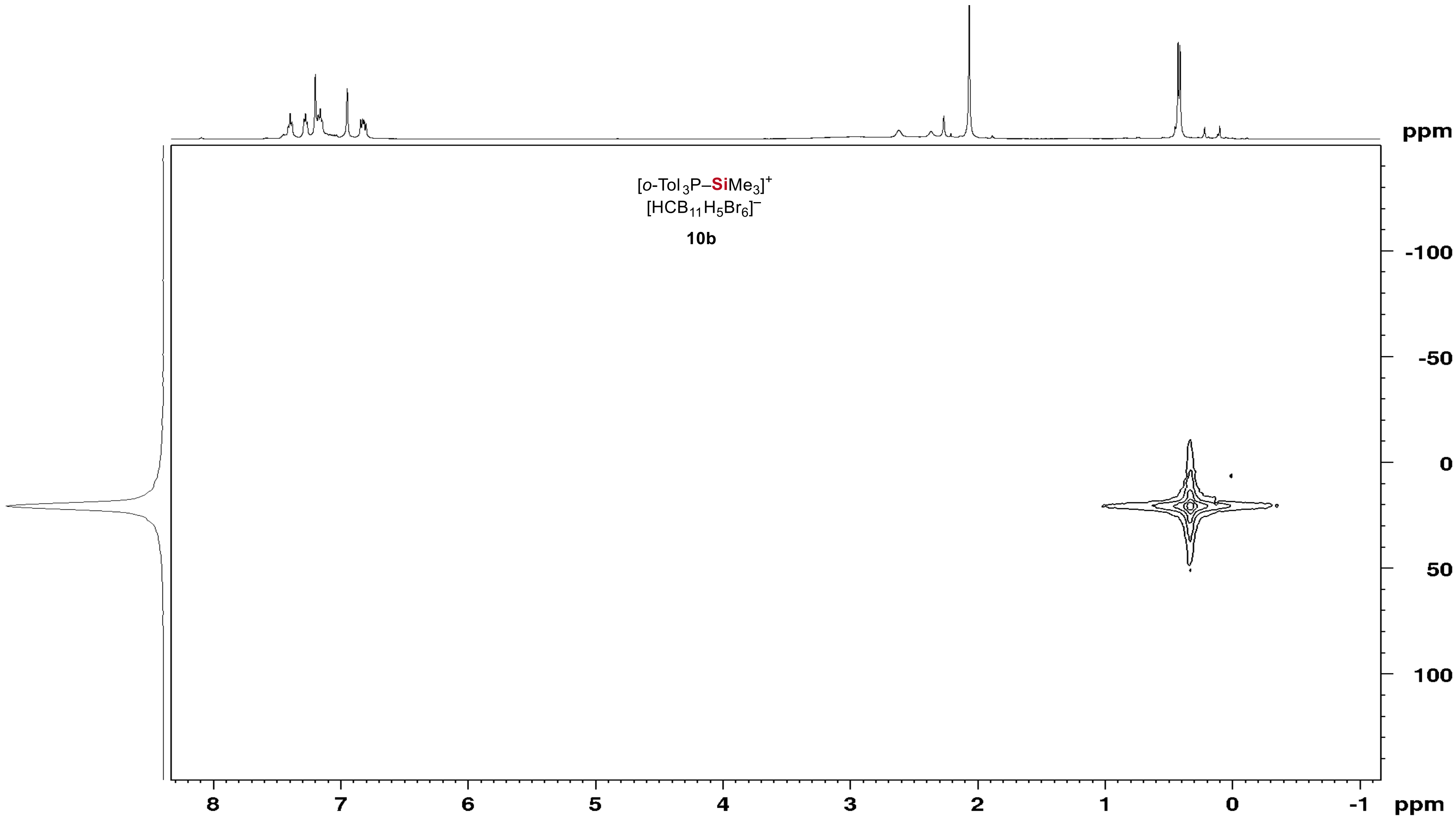

Figure S124. $^{13}C\{^1H\}$ **NMR spectrum (126 MHz, 1,2-** $C_6D_4Cl_2$**, 298 K) of 10b**

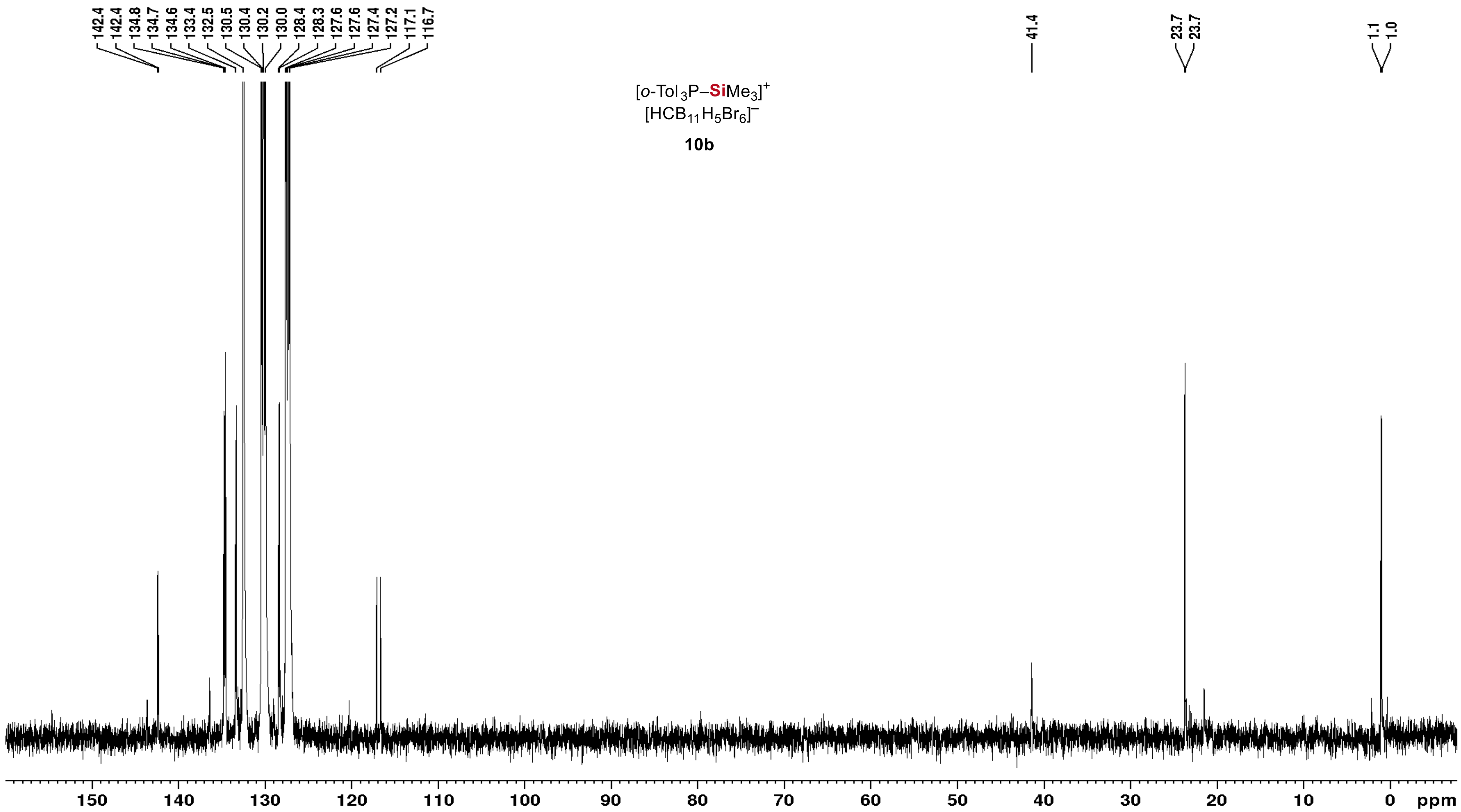

Figure S125. **^{1}H NMR spectrum (500 MHz, CD_2Cl_2, 298 K) of 11•MeCN**

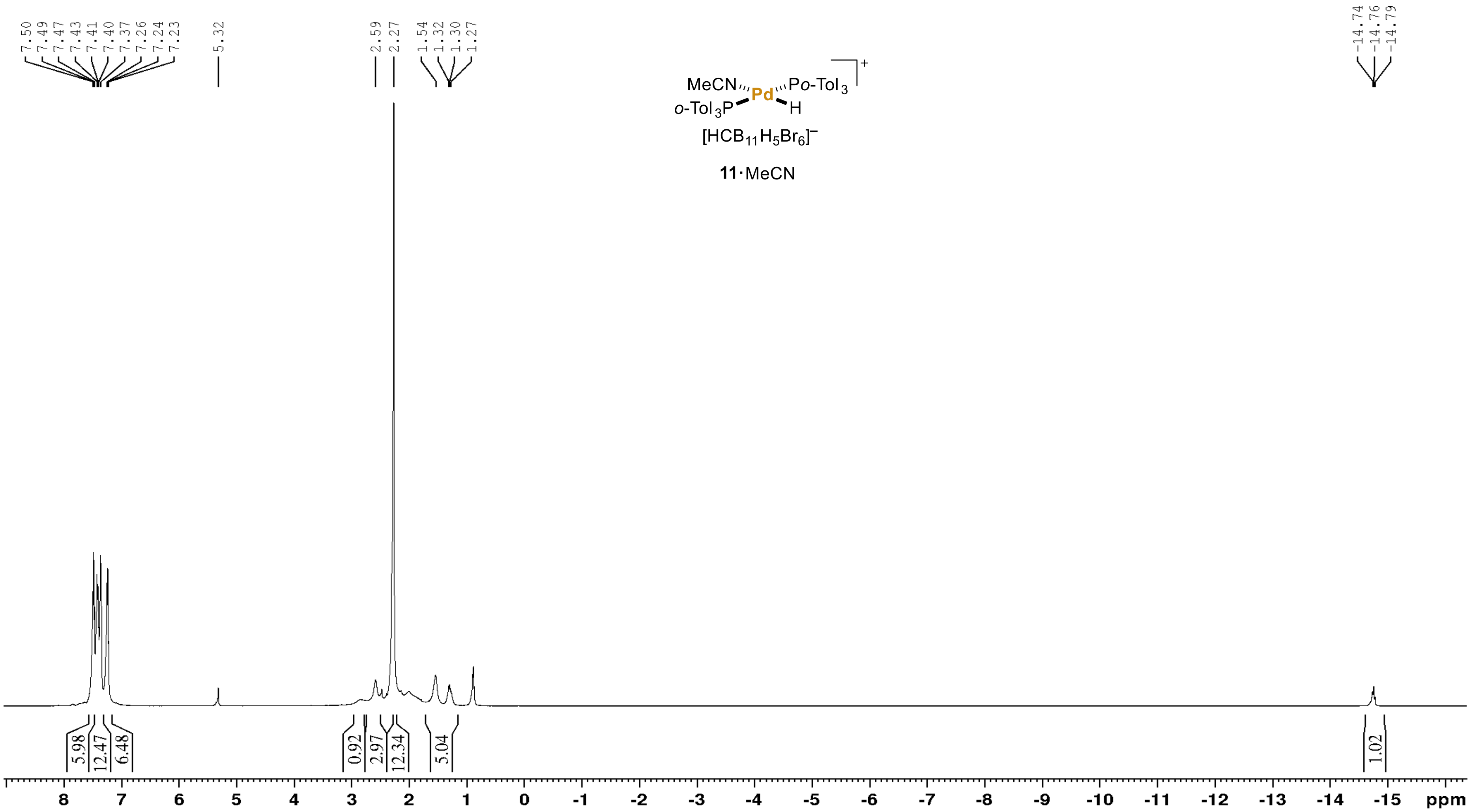

Figure S126. $^{31}P\{^1H\}$ NMR spectrum (202 MHz, CD_2Cl_2, 298 K) of 11•MeCN

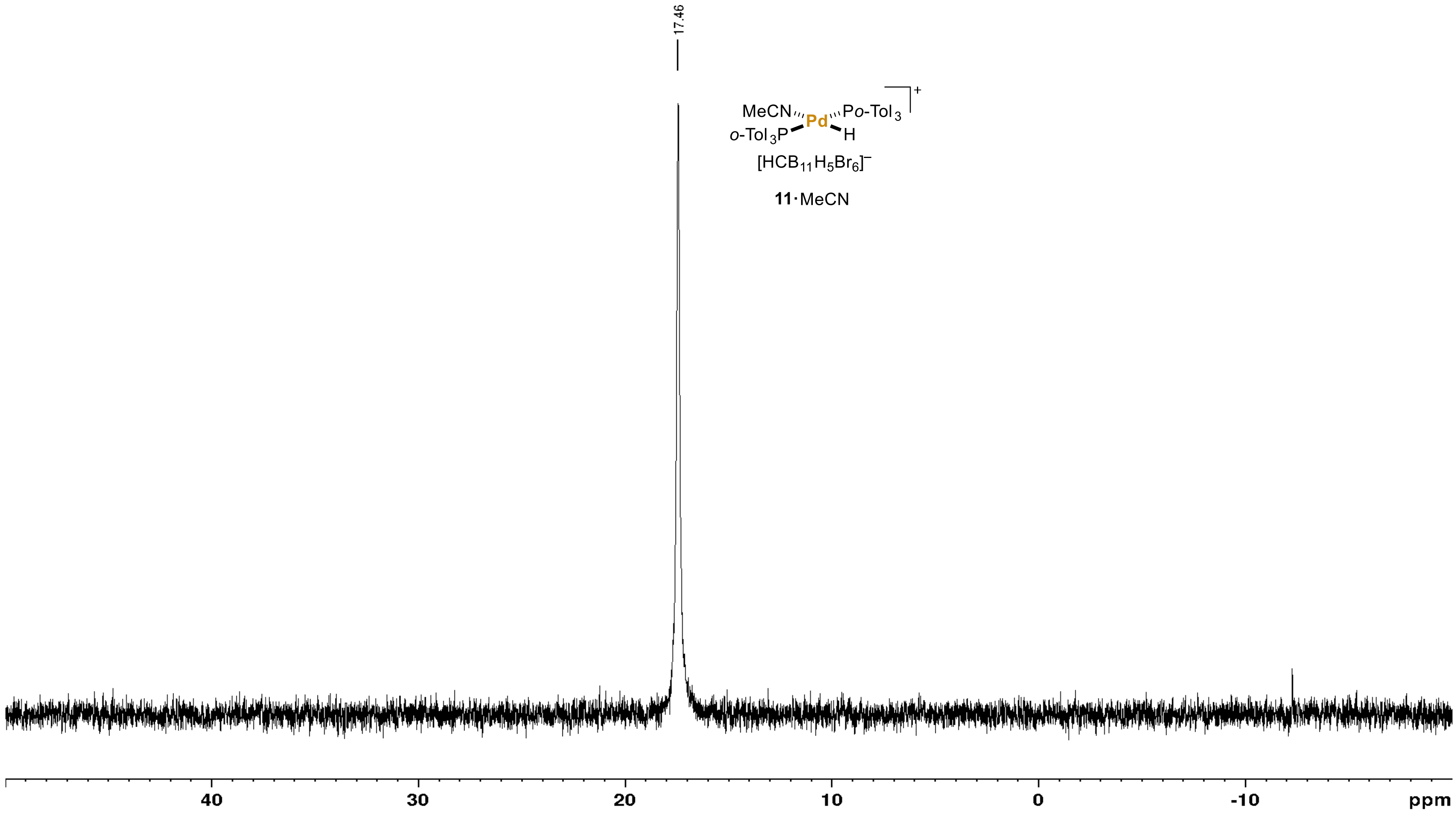

Figure S127. $^{13}C\{^{1}H\}$ **NMR spectrum (126 MHz,** CD_2Cl_2**, 298 K) of 11•MeCN**

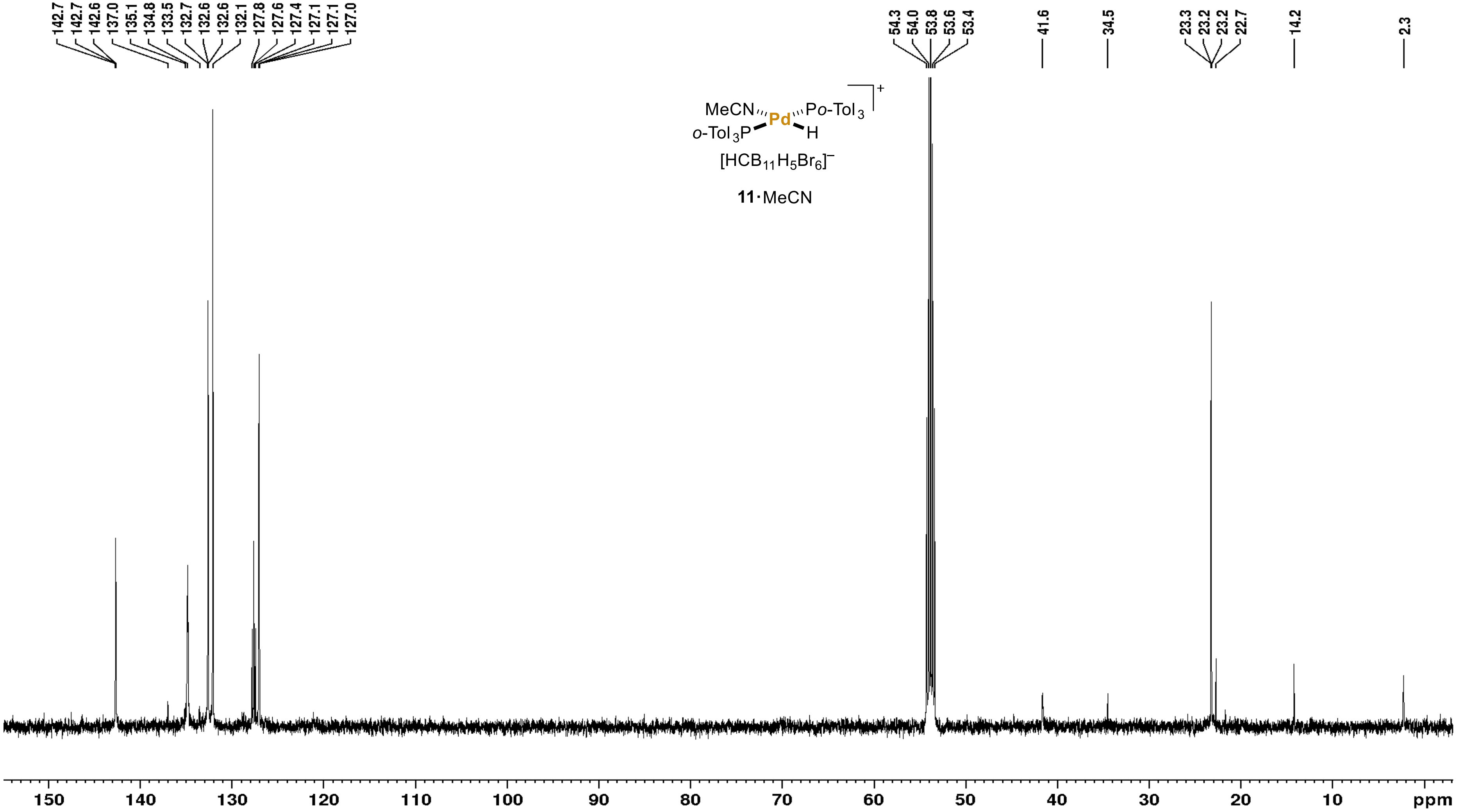

Figure S128. ^{1}H NMR spectrum (500 MHz, CD_2Cl_2, 298 K) of 12

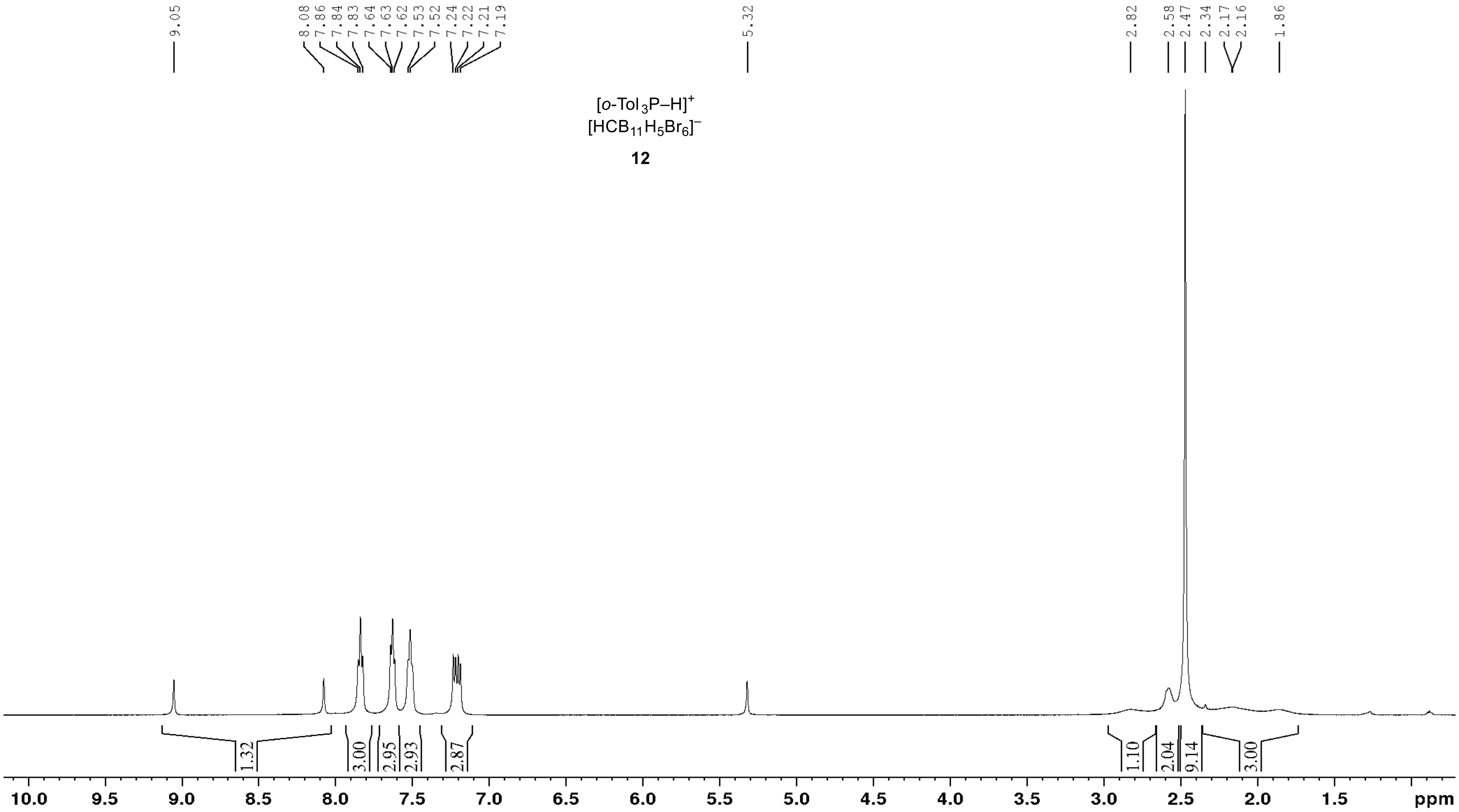

Figure S129. $^{31}P\{^1H\}$ NMR spectrum (202 MHz, CD_2Cl_2, 298 K) of 12

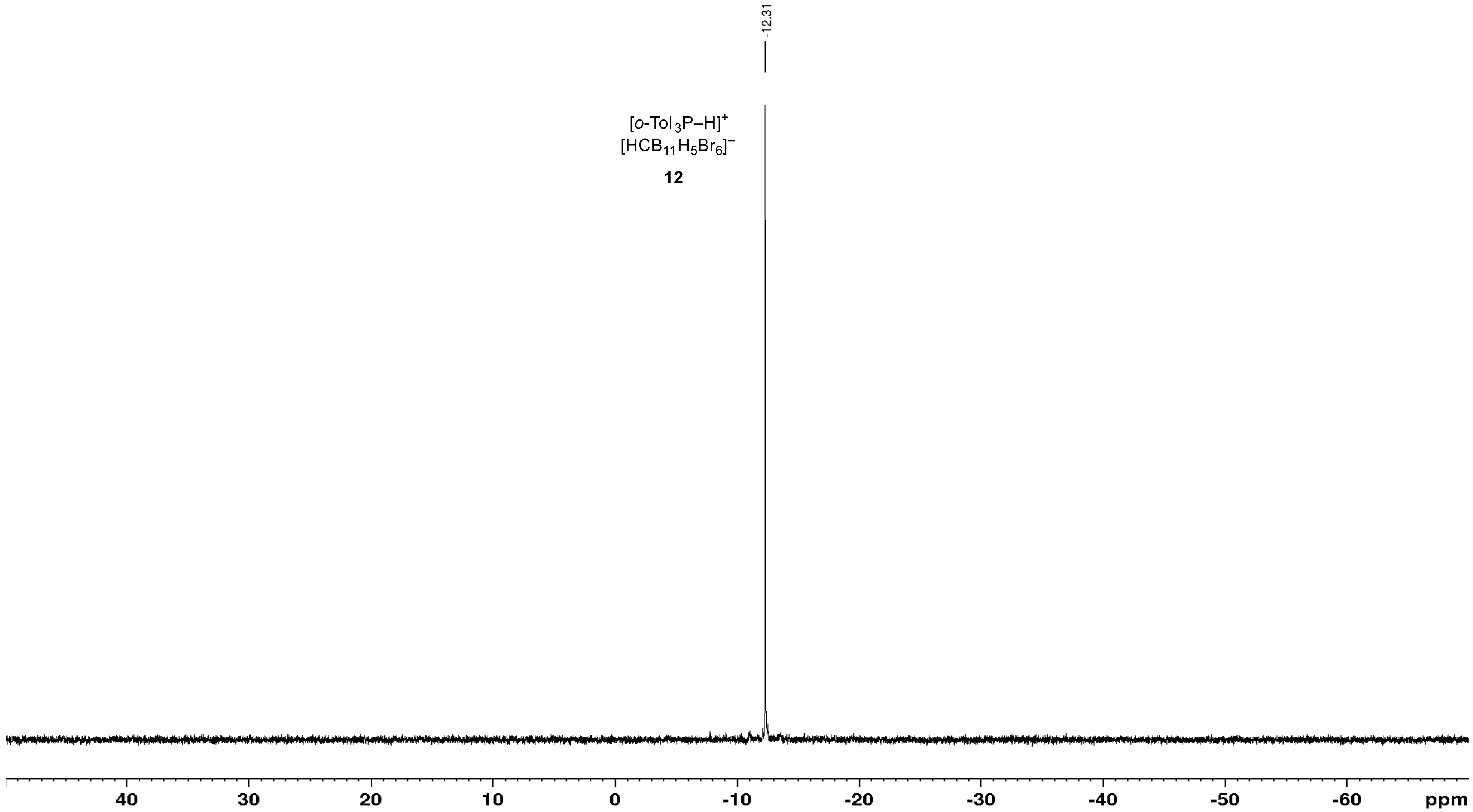

Figure S130. $^{13}C\{^{1}H\}$ **NMR spectrum (126 MHz, CD_2Cl_2, 298 K) of 12**

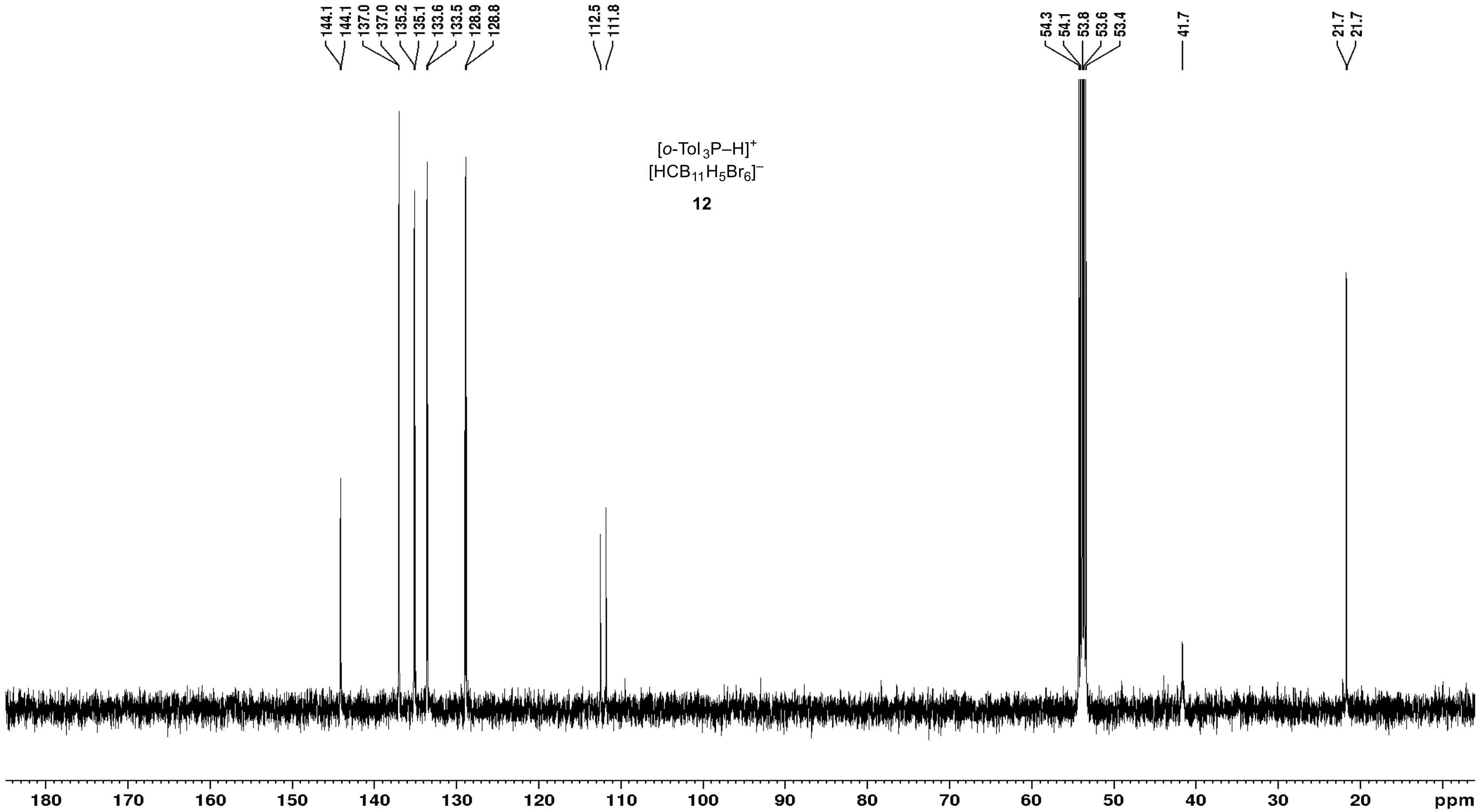

9 Computational Data

Computational Details: The quantum chemical DFT calculations have been performed with the TURBOMOLE 7.4 suite of programs [63] The structures are fully optimized at the TPSS-D3/def2-TZVP + COSMO level of theory, which combines the TPSS meta-GGA density functional [64] with the BJ-damped DFT-D3 dispersion correction [65, 66] and the def2-TZVP basis set [67, 68] using the Conductor-like Screening Model (COSMO) continuum solvation model [69] for CH_2Cl_2 solvent (dielectric constant ε = 8.93 and solvent radius R_{solv} = 2.94 Å). The density-fitting RI-J approach [67, 70, 71] is used to accelerate the geometry optimization and numerical harmonic frequency calculations [72] in solution. The optimized structures are characterized by frequency analysis to identify the nature of located stationary points (no imaginary frequency for true minima and only one imaginary frequency for transition state) and to provide thermal corrections (at 298.15 K and 1 atm) according to the modified ideal gas–rigid rotor–harmonic oscillator model [73]. This choice of dispersion-corrected meta-GGA functional makes the efficient exploration of all potential reaction paths possible.

The final solvation free energies in CH_2Cl_2 are computed with the COSMO-RS solvation model [74] (parameter file: BP_TZVP_C30_1601.ctd) using the COSMOtherm program package [75] on the above TPSS-D3 optimized structures, and corrected by +1.89 kcal·mol^{-1} to account for higher reference solute concentration of 1 mol·L^{-1} usually used in solution. To check the effects of the chosen DFT functional on the reaction energies and barriers, single-point calculations at the meta-GGA TPSS-D3 [64] and hybrid-meta-GGA PW6B95-D3 [76] levels are performed using a larger def2-QZVP basis set [68, 77]. The final reaction Gibbs free energies (ΔG) are determined from the electronic single-point energies plus TPSS-D3 thermal corrections and COSMO-RS solvation free energies. In our discussion, higher-level PW6B95-D3 Gibbs free energies (in kcal·mol^{-1}, at 298.15 K and 1 mol·L^{-1} standard state concentration) will be used unless specified otherwise. Meta-GGA functionals like TPSS tend to underestimate reaction barriers that can be evidently improved by using hybrid meta-GGA functionals like PW6B95. The applied DFT methods in combination with the large AO basis set provide usually accurate electronic energies leading to errors for chemical energies (including barriers) on the order of typically 1–2 kcal·mol^{-1}. This has been tested thoroughly for the huge data base GMTKN55 [78] which is the common standard in the field of DFT benchmarking.

Table S4. TPSS-D3/def2-TZVP + COSMO computed lowest imaginary frequency (ImF), zero-point energies (ZPE), gas-phase enthalpic (Hc) and Gibbs free-energy (Gc) corrections; the COSMO-RS computed solvation enthalpic (Hsol) and Gibbs free-energy (Gsol) corrections in CH_2Cl_2 solution; TPSS-D3/def2-QZVP and PW6B95-D3/def2-QZVP single-point energies (TPSS-D3 and PW6B95-D3); the relative electronic energies (ΔE_T and ΔE_P) and Gibbs free-energies (ΔG_T and ΔG_P) at the TPSS-D3 and PW6B95-D3 levels. Each structure is labeled either by its molecular formula or a specific number in bold (see main-text Scheme 5). Transition structures (with only one imaginary frequency) are indicated by the "**TS**" prefix.

Reactions in CH_2Cl_2	ImF cm^{-1}	ZPE kcal/mol	Hc kcal/mol	Gc kcal/mol	Hsol kcal/mol	Gsol kcal/mol	TPSS-D3 E_h	PW6B95-D3 E_h	G_P E_h	ΔE_T kcal/mol	ΔE_P kcal/mol	*ΔG_P kcal/mol*	ΔG_T kcal/mol
Silylium Me_3Si^+ transfer from $Me_3Si^+CB^-$ to neutral Pd complex is -19.4 kcal/mol exergonic.													
$Me_3Si^+CB^-$ + **8**	0	587.12	632.99	510.20	-66.39	-49.49	-18607.63763	-18618.13338	-18617.39317	0.00	0.00	*0.00*	0.00
**9b**$^+$ + CB^-	0	590.78	635.63	515.59	-121.05	-102.60	-18607.59355	-18618.08829	-18617.42410	27.66	28.29	*-19.41*	-20.04
*The total reaction of **1a** + **5b** is -5.2 kcal/mol exergonic to form the allylsilane **3ab**, but almost neutral to form the vinylsilane **4ab**.*													
**1a** + **5b**	0	211.68	225.16	168.99	-17.20	-10.68	-875.98553	-876.85187	-876.59355	0.00	0.00	*0.00*	0.00
**3ab** + **1v**	0	211.51	225.24	169.91	-17.14	-10.57	-875.99643	-876.86182	-876.60187	-6.84	-6.25	*-5.22*	-5.81
**4ab** + **1v**	0	211.77	225.43	170.23	-16.69	-10.23	-875.98895	-876.85479	-876.59379	-2.14	-1.83	*-0.15*	-0.45
*Silylium transfer from **9b**$^+$ to **1a** via **TS1**$^+$ is the rate-limiting step, while the two β-hydride eliminations (via **TS2**$^+$ and **TS3**$^+$) determine the allylsilane-to-vinylsilane selectivity.*													
**1a** + **9b**$^+$	0	614.12	652.85	547.42	-70.97	-57.26	-3196.13917	-3199.27500	-3198.48786	0.00	0.00	*-10.00*	0.00
**TS1**$^+$	-37	612.77	652.36	559.67	-70.52	-58.81	-3196.09935	-3199.23734	-3198.43617	24.99	23.63	*22.44*	33.79
**8** + **13ab**$^+$	0	612.06	651.02	543.79	-89.26	-74.40	-3196.07320	-3199.21019	-3198.45614	41.39	40.67	*9.91*	20.63
**14ab**$^+$	0	615.63	654.44	564.04	-65.40	-53.98	-3196.15537	-3199.28782	-3198.47196	-10.17	-8.04	*-0.02*	7.85
**TS2**$^+$	138i	612.13	651.00	560.52	-64.70	-53.61	-3196.14455	-3199.27710	-3198.46627	-3.38	-1.32	*3.55*	11.48
**11**$^+$ + **3ab**	0	610.34	649.50	541.56	-74.29	-59.48	-3196.12321	-3199.25733	-3198.48304	10.02	11.09	*-6.98*	1.95
**TS3**$^+$	118i	612.82	651.41	561.55	-65.79	-54.37	-3196.13265	-3199.26573	-3198.45446	4.09	5.82	*10.96*	19.23
**11**$^+$ + **4ab**	0	610.60	649.70	541.88	-73.85	-59.14	-3196.11572	-3199.25030	-3198.47496	14.72	15.50	*-1.91*	7.31

*Direct proton transfer from **11**$^+$ to allylsilane **5b** (via **TS5**$^+$) is kinetically less favorable than hydropalladation (via **TS4**$^+$).*													
**11**$^+$ + **5b**	0	560.00	596.32	494.98	-67.97	-54.63	-2964.90999	-2967.78763	-2967.07984	23.24	21.17	*-1.76*	0.31
**TS4**$^+$	92i	561.71	597.80	512.36	-62.27	-51.61	-2964.93099	-2967.80640	-2967.06912	10.06	9.39	*4.96*	5.64
**14vb**$^+$	0	565.56	601.33	516.76	-62.40	-51.58	-2964.94702	-2967.82136	-2967.07703	0.00	0.00	*0.00*	0.00
**TS5**$^+$	29i	559.96	596.54	509.29	-63.55	-52.40	-2964.92414	-2967.79159	-2967.06048	14.36	18.68	*10.39*	6.07
**8** + **13vb**$^+$	0	561.13	597.60	496.05	-88.45	-74.52	-2964.86407	-2967.74360	-2967.06583	52.06	48.79	*7.03*	10.30
*β-Silyl elimination (via **TS6**$^+$) is kinetically less favorable than Pd elimination from **14vb**$^+$ followed by silylium ion transfer via **TS7**$^+$.*													
**14vb**$^+$	0	565.56	601.33	516.76	-62.40	-51.58	-2964.94702	-2967.82136	-2967.07703	0.00	0.00	*0.00*	0.00
**TS6**$^+$	86i	565.67	601.12	517.42	-61.45	-51.20	-2964.90020	-2967.77296	-2967.02696	29.38	30.37	*31.42*	30.44
**8** + **13vb**$^+$	0	561.13	597.60	496.05	-88.45	-74.52	-2964.86407	-2967.74360	-2967.06583	52.06	48.79	*7.03*	10.30
**TS7**$^+$	82i	561.95	598.94	511.22	-68.49	-57.10	-2964.88678	-2967.76667	-2967.03998	37.80	34.32	*23.25*	26.74
**9b**$^+$ + **1v**	0	563.61	599.75	501.75	-64.59	-52.30	-2964.93685	-2967.81526	-2967.09297	6.38	3.83	*-10.00*	-7.45

Figure S131. The TPSS-D3/def2-TZVP + COSMO computed molecular structures of the transition states **TS1**$^+$–**TS7**$^+$ shown in the catalytic cycle of the migratory alkene silylation (see main-text Scheme 5). Color code: Pd blue, Si gray-green, P orange, C black, H gray. Most H atoms are omitted for clarity. Bond distances are given in Å.

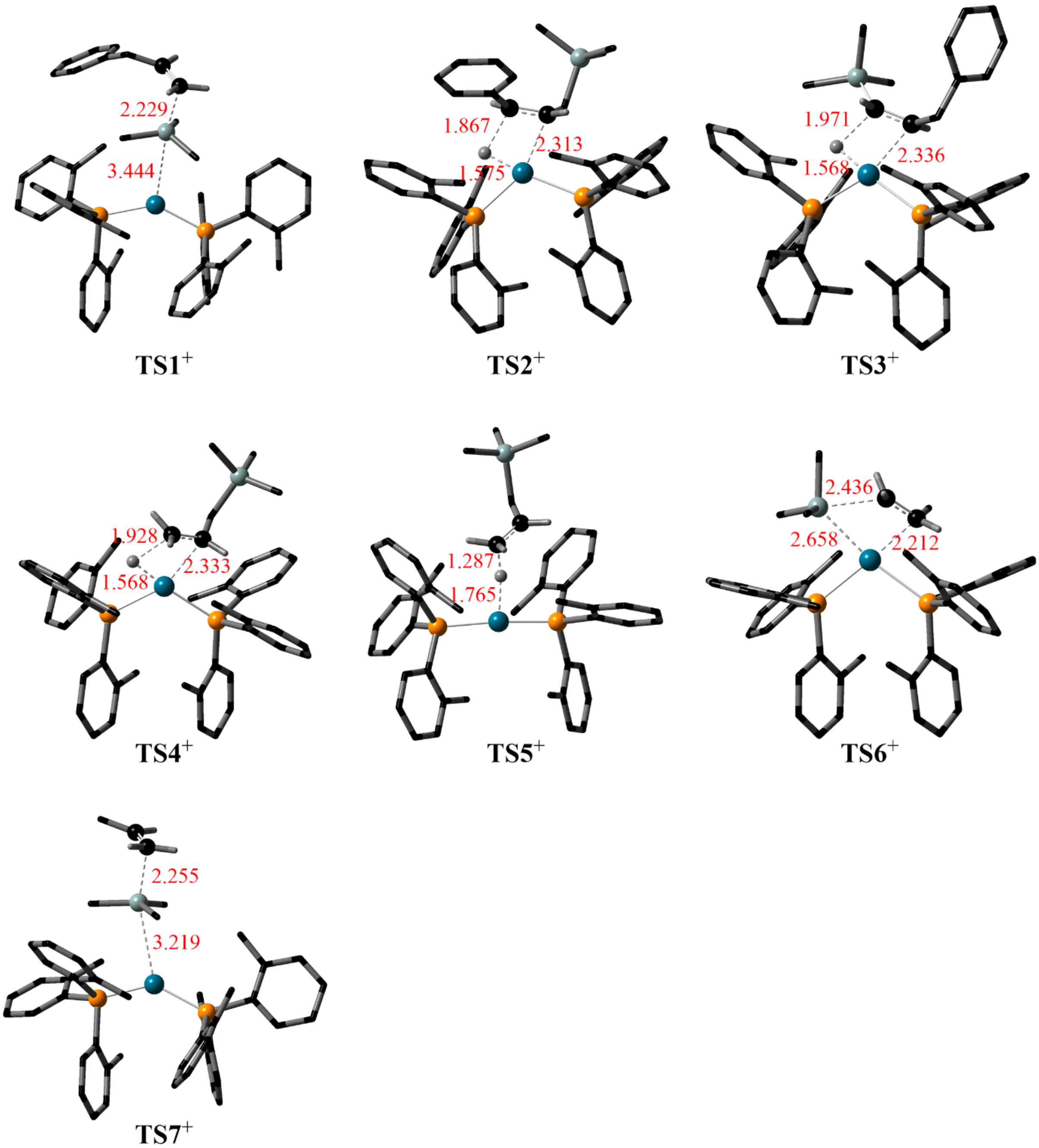

Table S5. The TPSS-D3/def2-TZVP + COSMO optimized atomic Cartesian coordinates (in Å) in CH_2Cl_2 solution. Each structure is labeled by a specific number (see main-text Scheme 5), followed by the number of atoms, the total energy, and the detailed atomic coordinates.

Me_3Si^+: silylium ion
13
Energy = -409.1683333572

Si	-0.0001789	0.0000421	0.0017876
C	-0.0176085	1.8302904	0.0007216
H	0.5364235	2.1918511	0.8775775
H	0.5217343	2.1888366	-0.8866870
C	-1.5762595	-0.9305556	0.0002809
H	-2.1657599	-0.6331806	0.8781155
H	-2.1573133	-0.6418496	-0.8862111
C	1.5937447	-0.8996471	0.0008532
H	1.6306438	-1.5587843	0.8787893
H	1.6343529	-1.5474368	-0.8854956
H	-1.4261767	-2.0122960	0.0058566
H	2.4556167	-0.2289535	0.0065404
H	-1.0292210	2.2416843	0.0078713

CB⁻: carborate anion
24
Energy = -15760.36023887

B	0.0000033	1.5183875	-0.7552862
B	1.4439106	0.4692111	-0.7551718
B	0.8925096	-1.2284673	-0.7553548
B	-0.8925119	-1.2284695	-0.7553514
B	-1.4439056	0.4692138	-0.7551706
B	0.0000016	-0.0001192	0.1908630
B	-0.0000040	-1.5116769	-2.2568876
B	-1.4376022	-0.4671513	-2.2568094
B	-0.8884656	1.2227911	-2.2568094
B	0.8884761	1.2227848	-2.2568105
B	1.4376027	-0.4671613	-2.2568120
C	0.0000003	-0.0001977	-3.0362190
H	-0.0000004	-0.0000982	-4.1183336
H	-0.0000194	-2.5152000	-2.8803359
H	-2.3918685	-0.7772421	-2.8802828
H	-1.4782850	2.0344174	-2.8803710
H	1.4783046	2.0344039	-2.8803727
H	2.3918560	-0.7772685	-2.8802943
Br	-0.0000122	3.2836744	0.1010002
Br	3.1218350	1.0156391	0.1019357
Br	1.9306150	-2.6562938	0.1005748
Br	-1.9305907	-2.6563171	0.1005568
Br	-3.1218398	1.0156146	0.1019372
Br	-0.0000097	-0.0004739	2.1442376

$Me_3Si^+CB^-$: silylium carborate
37
Energy = -16169.58219207

B	0.3486933	-2.4958521	1.6347628
B	1.3518982	-1.8093467	0.3118541
B	2.8023234	-2.8508635	0.1132142
B	2.6896765	-4.1864557	1.3056270
B	1.1734707	-3.9671023	2.2456620
B	2.1185607	-2.5537221	1.7219063
B	2.2459817	-4.4599271	-0.3827677
B	1.2508852	-5.1424751	0.9265114
B	-0.1849622	-4.1082403	1.1245241
B	-0.0752071	-2.7872513	-0.0586067
B	1.4273203	-3.0046937	-0.9903024
C	0.5498230	-4.3326184	-0.3929869
H	0.0144171	-4.9340589	-1.1160920
H	2.8079231	-5.1775039	-1.1324237
H	1.1559672	-6.3126127	1.0463573
H	-1.2318439	-4.5932618	1.3724597
H	-1.0464932	-2.3910562	-0.5993507
H	1.4428763	-2.7512575	-2.1427498
Br	-0.7160857	-1.3539945	2.8112292
Br	1.3690729	0.1024746	-0.1061908
Br	4.5251980	-2.1124810	-0.4416103
Br	4.2863732	-4.9742199	2.1146232
Br	1.0488510	-4.5053914	4.1215435
Br	3.1613158	-1.6554621	3.1956200
Si	3.5040958	0.7678692	3.1879642
C	4.4822429	0.7656935	4.7700774
H	4.7638253	1.8059377	4.9821435
H	3.8946967	0.3969426	5.6161895
H	5.4017623	0.1788975	4.6848141
C	4.5112387	1.1394598	1.6796885
H	3.9706324	0.9271936	0.7554475
H	4.7545529	2.2104919	1.7048798
H	5.4468199	0.5729518	1.6881948
C	1.8235705	1.5286295	3.3439375
H	1.9640271	2.6143249	3.4361483
H	1.1948167	1.3318709	2.4736489
H	1.3161742	1.1691152	4.2437541

1a: $PhCH_2CH=CH_2$
19
Energy = -349.1720228602

C	3.4127201	0.6081716	-0.2473439
H	3.6286389	0.9499886	0.7633071
H	4.0594692	0.9747925	-1.0401434
C	2.4004732	-0.2246828	-0.4979449
H	2.2052221	-0.5427700	-1.5227707
C	1.4565712	-0.7643476	0.5450050
H	1.5056392	-1.8607312	0.5541398
H	1.7733011	-0.4133518	1.5349651
C	0.0184912	-0.3478091	0.2846765
C	-0.9700769	-1.2987820	0.0087868
C	-0.3409755	1.0068499	0.3070110
C	-2.2901319	-0.9098426	-0.2341720
H	-0.7041399	-2.3533830	-0.0131982

C -1.6576689 1.3998550 0.0689649
H 0.4199719 1.7570027 0.5099311
C -2.6381163 0.4412467 -0.2035933
H -3.0447258 -1.6627034 -0.4461068
H -1.9200001 2.4542204 0.0944819
H -3.6638239 0.7465551 -0.3910121

1v: $CH_3CH=CH_2$
9
Energy = -117.9798792861
C -1.2831434 -0.2366263 -0.0877217
H -1.2849522 -1.3047459 -0.2992287
H -2.2511884 0.2538031 -0.0258712
C -0.1437772 0.4373511 0.0871584
H -0.1903841 1.5070632 0.2972401
C 1.2305688 -0.1608694 0.0187438
H 1.8304787 0.3285319 -0.7595546
H 1.7669938 -0.0145526 0.9653042
H 1.1897739 -1.2333590 -0.1960742

3ab: $PhCH=CHCH_2SiMe_3$
31
Energy = -757.9737558217
C 3.6800697 0.5341737 -0.1199420
H 4.1054561 1.5297018 -0.3073089
C 2.2026585 0.5400220 -0.3213504
H 1.8463733 1.0868116 -1.1946553
C 1.3148398 -0.1217921 0.4447298
H 1.6879337 -0.6606858 1.3173946
H 3.9375937 0.2443886 0.9072158
C -0.1330831 -0.2082703 0.2395939
C -0.9128214 -0.9059062 1.1817435
C -0.7981562 0.3703371 -0.8602593
C -2.2953420 -1.0198121 1.0386708
H -0.4191281 -1.3622427 2.0370345
C -2.1777419 0.2587196 -1.0030428
H -0.2304788 0.9107327 -1.6126900
C -2.9372529 -0.4366903 -0.0550769
H -2.8710584 -1.5645689 1.7823068
H -2.6663350 0.7140494 -1.8605564
H -4.0138461 -0.5228095 -0.1714673
Si 4.5389520 -0.6970629 -1.3030944
C 6.4041751 -0.5291570 -1.0961780
H 6.9357110 -1.2335899 -1.7478795
H 6.7406764 0.4831582 -1.3509949
H 6.7074731 -0.7347272 -0.0623865
C 3.9843218 -2.4383379 -0.8532103
H 4.2853144 -2.6974648 0.1691664
H 2.8935183 -2.5284299 -0.9153220
H 4.4247166 -3.1791949 -1.5315493
C 4.0447979 -0.3010924 -3.0773029
H 2.9640625 -0.4158628 -3.2229510
H 4.3136397 0.7280224 -3.3452006
H 4.5495932 -0.9735438 -3.7816752

4ab: $PhCH_2CH=CHSiMe_3$
31
Energy = -757.9653172817
C 3.1297352 1.2258664 -0.0362097
H 3.7113702 0.3712375 -0.3996854
C 1.9691947 0.9468009 0.5772713
H 1.3429143 1.7603674 0.9494267
C 1.3977218 -0.4328594 0.7812134
H 1.2406898 -0.6167714 1.8520457
H 2.1183607 -1.1784692 0.4219705
C 0.0696707 -0.6090509 0.0628586
C -1.0800443 -1.0043787 0.7555581
C -0.0258660 -0.3722726 -1.3153316
C -2.2968293 -1.1693267 0.0887608
H -1.0210928 -1.1860477 1.8265562
C -1.2369901 -0.5387866 -1.9859660
H 0.8574729 -0.0492740 -1.8611646
C -2.3785564 -0.9383965 -1.2851604
H -3.1792897 -1.4773086 0.6434302
H -1.2926879 -0.3543549 -3.0556518
H -3.3233795 -1.0657669 -1.8063045
Si 3.7987113 2.9475323 -0.3350703
C 3.9779508 3.2178036 -2.1941696
H 4.4024438 4.2065086 -2.4088880
H 4.6396999 2.4649737 -2.6398294
H 3.0059751 3.1500506 -2.6972154
C 2.6171738 4.2242737 0.3872649
H 1.6244711 4.1591563 -0.0739190
H 2.4965805 4.0940124 1.4693512
H 2.9993589 5.2378011 0.2138389
C 5.4957129 3.1086550 0.4738038
H 5.4296625 2.9776606 1.5604623
H 6.1898825 2.3530244 0.0858831
H 5.9318721 4.0961258 0.2776331

5b: $CH_2=CHCH_2SiMe_3$
21
Energy = -526.7703815624
Si -0.7491584 0.0614877 0.0131008
C 0.8933034 1.0100526 0.2192848
H 0.7551512 1.7359252 1.0331599
H 1.0728217 1.5760737 -0.7044600
C 2.0442131 0.1021957 0.5168826
C 2.9822841 -0.2843745 -0.3556410
H 2.0811168 -0.3005534 1.5303042
H 3.7675114 -0.9807495 -0.0739115
H 2.9924405 0.0823910 -1.3806170
C -2.1364596 1.3155860 -0.2208420
H -3.1015112 0.8126349 -0.3592878
H -2.2242137 1.9754897 0.6509097
H -1.9569850 1.9440759 -1.1017132
C -0.6197781 -1.0523504 -1.4988588
H -0.4633420 -0.4632611 -2.4108383
H 0.2214398 -1.7484893 -1.4008975
H -1.5346662 -1.6423957 -1.6326245
C -1.0726495 -0.9762082 1.5523078

H -1.1147272 -0.3497941 2.4518097
H -2.0283788 -1.5082408 1.4697262
H -0.2856974 -1.7253592 1.7002770

8: (o-$Tol_3P)_2Pd$
87
Energy = -2437.601398676

Pd	-0.0246049	-0.4528615	0.2095623
P	2.2355136	-0.2809018	0.0358431
P	-2.2511771	-0.0127127	0.0811099
C	3.0153645	-1.3784918	-1.2039052
C	2.6648853	-2.7496127	-1.2383192
C	3.9085459	-0.8660790	-2.1551761
C	3.2227608	-3.5525895	-2.2400474
C	4.4523151	-1.6830053	-3.1450780
H	4.1755391	0.1863611	-2.1186299
C	4.1050167	-3.0333736	-3.1881982
H	2.9553941	-4.6063779	-2.2742322
H	5.1415179	-1.2667866	-3.8746274
H	4.5183802	-3.6819924	-3.9558210
C	2.7140882	1.4111033	-0.4841948
C	3.6369446	2.1481818	0.2722695
C	2.0805145	2.0203655	-1.5969325
C	3.9289281	3.4757358	-0.0367377
H	4.1286962	1.6750246	1.1171626
C	2.3896434	3.3537937	-1.8885659
C	3.2968873	4.0840033	-1.1208963
H	4.6443542	4.0277357	0.5665583
H	1.8983490	3.8267390	-2.7359388
H	3.5090925	5.1201103	-1.3705638
C	3.2067234	-0.5791171	1.5617826
C	4.3347303	-1.4120956	1.5349404
C	2.7763797	-0.0248810	2.7927175
C	5.0381580	-1.7122109	2.7003681
H	4.6624543	-1.8312527	0.5881730
C	3.5003747	-0.3362756	3.9499352
C	4.6173364	-1.1714125	3.9150949
H	5.9082941	-2.3613687	2.6567733
H	3.1743351	0.0862881	4.8977783
H	5.1543651	-1.3970106	4.8324389
C	-2.5582812	1.4507448	-0.9802470
C	-3.4499669	1.3659043	-2.0595566
C	-1.8237773	2.6463514	-0.7752708
C	-3.6138006	2.4280074	-2.9473663
H	-4.0193896	0.4528246	-2.2068401
C	-2.0051021	3.6995291	-1.6788946
C	-2.8823744	3.6002995	-2.7587562
H	-4.3076605	2.3373192	-3.7785184
H	-1.4365502	4.6146628	-1.5295457
H	-2.9944152	4.4347740	-3.4457489
C	-3.3131552	-1.3247624	-0.6341659
C	-4.5072487	-1.6971514	-0.0000283
C	-2.8917048	-2.0199662	-1.7944309
C	-5.2855712	-2.7463660	-0.4860674
H	-4.8275056	-1.1584888	0.8869772
C	-3.6907088	-3.0679575	-2.2671428
C	-4.8734996	-3.4364759	-1.6257830
H	-6.2064625	-3.0191581	0.0219013
H	-3.3715616	-3.6056917	-3.1572088
H	-5.4684028	-4.2579809	-2.0157428
C	-3.0656396	0.3950582	1.6686557
C	-3.8663520	1.5392146	1.7911762
C	-2.8352839	-0.4239001	2.8001817
C	-4.4338699	1.8928996	3.0142024
H	-4.0417781	2.1589212	0.9163881
C	-3.4151218	-0.0511979	4.0185514
C	-4.2043525	1.0936455	4.1342754
H	-5.0503969	2.7843844	3.0892887
H	-3.2398789	-0.6743064	4.8927459
H	-4.6373020	1.3582090	5.0952012
C	1.5845836	0.8878890	2.8851771
H	0.7160087	0.4481595	2.3618661
H	1.3213218	1.0814688	3.9286205
H	1.7795722	1.8481111	2.3921052
C	1.0966298	1.2825551	-2.4603504
H	0.6055051	1.9623739	-3.1611988
H	1.5851895	0.4814440	-3.0275277
H	0.3312155	0.7942360	-1.8300656
C	1.7305276	-3.3556022	-0.2267279
H	1.4713669	-4.3820630	-0.5000635
H	2.1805185	-3.3646792	0.7734865
H	0.8065372	-2.7541514	-0.1432861
C	-2.0050829	-1.6759055	2.7182290
H	-1.8227215	-2.0895018	3.7138314
H	-2.5008317	-2.4413587	2.1088686
H	-1.0377762	-1.4680702	2.2250290
C	-1.6306756	-1.6568598	-2.5294457
H	-1.3979664	-2.4013959	-3.2956388
H	-1.7203400	-0.6766034	-3.0130929
H	-0.7813858	-1.5729414	-1.8273417
C	-0.8670622	2.8115415	0.3715644
H	-1.3951940	2.8309961	1.3324071
H	-0.1749857	1.9510003	0.4149921
H	-0.2891112	3.7334655	0.2683444

9b$^+$: (o-$Tol_3P)_2PdSiMe_3^+$
100
Energy = -2846.854647062

Pd	-0.0084387	-0.3640873	0.0488309
P	-2.3519329	0.0283579	0.1479362
P	2.2721831	-0.0247113	-0.1651007
C	-2.3995059	1.5212998	-0.9178788
C	-1.6881954	2.6777434	-0.5189929
C	-3.1529293	1.5365696	-2.0997906
C	-1.7514007	3.8090131	-1.3387713
C	-3.1929787	2.6724367	-2.9052160
H	-3.7313430	0.6616979	-2.3780378
C	-2.4854935	3.8123791	-2.5240573
H	-1.2090745	4.7010773	-1.0371889
H	-3.7804804	2.6669288	-3.8182244
H	-2.5093624	4.7040847	-3.1435406
C	-3.5129392	-1.1683498	-0.5933927

C	-4.6856631	-1.4979068	0.1047292
C	-3.2135636	-1.8260929	-1.8102989
C	-5.5660185	-2.4571861	-0.3882647
H	-4.9094317	-1.0031896	1.0435585
C	-4.1164636	-2.7835268	-2.2870596
C	-5.2815316	-3.1010130	-1.5918456
H	-6.4663405	-2.6993321	0.1679226
H	-3.8885563	-3.2938124	-3.2195044
H	-5.9596020	-3.8524076	-1.9857744
C	-3.2189212	0.6442627	1.6445638
C	-4.2269850	1.6040336	1.4355100
C	-2.9262912	0.2152976	2.9537601
C	-4.9357366	2.1438733	2.5021796
H	-4.4525864	1.9330748	0.4261970
C	-3.6432551	0.7841603	4.0168993
C	-4.6360027	1.7351967	3.8034644
H	-5.7103283	2.8822237	2.3187607
H	-3.4088872	0.4669991	5.0299242
H	-5.1723660	2.1581549	4.6478905
C	3.3321105	-0.5083404	-1.5867976
C	4.6477386	-0.0030828	-1.5248942
C	2.9162171	-1.2273275	-2.7232424
C	5.5604225	-0.2437172	-2.5412151
H	4.9537324	0.5851525	-0.6647073
C	3.8605868	-1.4640565	-3.7374873
C	5.1639401	-0.9916791	-3.6540870
H	6.5696713	0.1495800	-2.4683639
H	3.5464330	-2.0255875	-4.6138245
H	5.8668158	-1.1932503	-4.4571644
C	2.3805735	1.8000735	-0.3265815
C	3.1740630	2.5517534	0.5508267
C	1.7094924	2.4368457	-1.3949024
C	3.2830897	3.9326707	0.4063016
H	3.7195101	2.0509063	1.3446246
C	1.8322033	3.8249364	-1.5189966
C	2.5999289	4.5728737	-0.6277022
H	3.8982603	4.5010086	1.0971887
H	1.3142974	4.3224643	-2.3345594
H	2.6710321	5.6500709	-0.7460874
C	3.2182072	-0.4831998	1.3260916
C	4.2737705	-1.4023425	1.2143333
C	2.8162486	-0.0337416	2.6093110
C	4.9616681	-1.8486321	2.3394904
H	4.5592482	-1.7778487	0.2382486
C	3.5431113	-0.4755816	3.7206561
C	4.6059049	-1.3681214	3.5986644
H	5.7703797	-2.5642586	2.2280013
H	3.2439607	-0.1271064	4.7057214
H	5.1396909	-1.6996072	4.4843677
C	-1.8970077	-0.8355446	3.2502520
H	-1.1052626	-0.8228360	2.4958769
H	-1.4507813	-0.6797235	4.2358826
H	-2.3435417	-1.8366511	3.2426887
C	-1.9637106	-1.5428043	-2.5939494
H	-1.7044230	-2.3888623	-3.2349279
H	-2.0693490	-0.6552230	-3.2275585
H	-1.1211818	-1.3462401	-1.9145296
C	-0.9147867	2.7487337	0.7705430
H	-0.1579988	3.5345914	0.7291038
H	-1.5809377	2.9432351	1.6188903
H	-0.3997051	1.8000331	0.9904466
C	1.6103362	0.8356886	2.8357923
H	1.7633655	1.8662182	2.5013078
H	1.3443905	0.8539714	3.8951627
H	0.7517730	0.4423414	2.2675796
C	0.9288305	1.6751025	-2.4317924
H	0.3700293	0.8315302	-1.9940372
H	0.2079811	2.3221552	-2.9349708
H	1.6034005	1.2514077	-3.1845985
C	1.5092571	-1.7026142	-2.9513233
H	1.4963481	-2.7447382	-3.2822164
H	0.9053440	-1.6126268	-2.0460944
H	1.0314757	-1.1037013	-3.7348997
Si	0.2760872	-2.5956906	0.7793076
C	-1.3843588	-3.4736212	0.6766982
H	-1.2191953	-4.5046522	1.0211953
H	-2.1565348	-3.0249190	1.3054058
H	-1.7642398	-3.5177540	-0.3472039
C	0.9470399	-2.6914039	2.5398025
H	0.5863596	-1.9271177	3.2285933
H	2.0396409	-2.6646115	2.5358874
H	0.6316272	-3.6698988	2.9291031
C	1.4819623	-3.5757085	-0.2859960
H	1.6862519	-4.5057883	0.2632412
H	2.4396118	-3.0714477	-0.4460213
H	1.0607228	-3.8446258	-1.2571454

11$^+$: (o-$Tol_3P)_2PdH^+$

88

Energy = -2438.036772056

Pd	0.0503453	0.0171458	0.0115157
H	-0.0563779	-0.9278263	1.2043279
P	2.3497627	-0.1789453	0.0801355
P	-2.2788417	0.0920699	0.0394146
C	2.9079396	-1.4573951	-1.0913460
C	2.4557201	-2.7937703	-0.9683938
C	3.7288335	-1.0877299	-2.1677058
C	2.8612114	-3.7151136	-1.9395924
C	4.1111534	-2.0243057	-3.1255195
H	4.0727433	-0.0620173	-2.2546043
C	3.6746382	-3.3429080	-3.0099253
H	2.5286233	-4.7461011	-1.8499060
H	4.7486680	-1.7236613	-3.9510390
H	3.9673374	-4.0832638	-3.7486568
C	2.9922134	1.4005402	-0.5668664
C	4.2270073	1.9018271	-0.1399339
C	2.2210683	2.1134529	-1.5072065
C	4.7038312	3.1143099	-0.6380612
H	4.8096958	1.3454747	0.5881045
C	2.7167595	3.3211347	-2.0018728
C	3.9470441	3.8227567	-1.5702407
H	5.6588259	3.5013972	-0.2965227

H	2.1326256	3.8762690	-2.7310277
H	4.3089678	4.7687570	-1.9614098
C	3.2128869	-0.4555599	1.6507730
C	4.1985646	-1.4491683	1.7334285
C	2.8899243	0.3298353	2.7820108
C	4.8655527	-1.6869835	2.9330116
H	4.4436398	-2.0359666	0.8532315
C	3.5741160	0.0703026	3.9743185
C	4.5480570	-0.9252940	4.0571324
H	5.6267254	-2.4593187	2.9853901
H	3.3362628	0.6652043	4.8524703
H	5.0594096	-1.1020492	4.9988787
C	-2.7358376	1.5721191	-0.9277549
C	-3.5401890	1.4267289	-2.0681928
C	-2.2028425	2.8410122	-0.5928713
C	-3.8168197	2.5179054	-2.8883228
H	-3.9500123	0.4520588	-2.3137516
C	-2.4945410	3.9202442	-1.4345503
C	-3.2875862	3.7685025	-2.5714046
H	-4.4388311	2.3885207	-3.7685609
H	-2.0880509	4.8979420	-1.1884439
H	-3.4901915	4.6256207	-3.2067453
C	-3.0826840	-1.3219536	-0.7865905
C	-4.2851253	-1.8195194	-0.2620674
C	-2.5096775	-1.9199672	-1.9329572
C	-4.9221740	-2.9098123	-0.8495606
H	-4.7218198	-1.3509470	0.6139512
C	-3.1682650	-3.0138643	-2.5050412
C	-4.3579180	-3.5112600	-1.9736419
H	-5.8513034	-3.2834236	-0.4304095
H	-2.7363609	-3.4798825	-3.3868945
H	-4.8420962	-4.3644967	-2.4394655
C	-3.1159529	0.2529731	1.6502338
C	-3.9815121	1.3391250	1.8518625
C	-2.9132314	-0.6892653	2.6857478
C	-4.6271569	1.5241548	3.0712933
H	-4.1597260	2.0389036	1.0417238
C	-3.5769349	-0.4822197	3.9011088
C	-4.4164986	0.6115346	4.1039547
H	-5.2948866	2.3694412	3.2067127
H	-3.4350036	-1.2061097	4.6996810
H	-4.9148430	0.7406950	5.0602456
C	1.8652190	1.4313425	2.7288642
H	0.9049402	1.0577117	2.3467062
H	1.7053290	1.8595210	3.7210669
H	2.1824796	2.2358279	2.0546540
C	0.8923271	1.5886420	-1.9900113
H	0.1242692	1.6301994	-1.1736001
H	0.4520724	2.2283967	-2.7594694
H	0.9721878	0.5800618	-2.4097519
C	1.5593795	-3.2491950	0.1533789
H	1.4685193	-4.3378992	0.1514419
H	1.9366018	-2.9355333	1.1322008
H	0.5550288	-2.8191351	0.0471269
C	-2.0393394	-1.9078193	2.5393851
H	-2.3484179	-2.6814027	3.2473925
H	-2.0780799	-2.3285854	1.5310837
H	-0.9896274	-1.6655459	2.7553638
C	-1.2425207	-1.4101799	-2.5648264
H	-0.4058185	-1.4378378	-1.8459748
H	-0.9648091	-2.0183810	-3.4289125
H	-1.3534752	-0.3725479	-2.9004385
C	-1.3711464	3.0790538	0.6417927
H	-2.0084692	3.2665670	1.5135410
H	-0.7481197	2.2066910	0.8927702
H	-0.7162019	3.9437974	0.5081619

13ab$^+$: $PhCH_2CHCH_2SiMe_3^+$
32
Energy = -758.3825651473

Si	2.0748990	-0.2240331	-0.1448893
C	1.0545268	1.4186692	0.8387907
H	1.9327098	1.8860240	1.2770168
H	0.3931052	0.8773678	1.5104458
C	0.5758666	1.8898226	-0.3573066
H	1.2158291	2.5508766	-0.9417090
C	1.3287249	-1.6244344	0.8256381
H	1.8001708	-2.5569827	0.4891618
H	1.5116774	-1.5119683	1.8982414
H	0.2515983	-1.7011787	0.6490960
C	1.7996643	-0.3493362	-1.9837632
H	0.7666053	-0.5859603	-2.2482208
H	2.1166399	0.5473587	-2.5242007
H	2.4265490	-1.1826555	-2.3286297
C	3.8304086	0.2279949	0.2727655
H	3.9820373	0.3159200	1.3526026
H	4.4864885	-0.5664896	-0.1053718
H	4.1268478	1.1664946	-0.2073107
C	-0.7631983	1.6154251	-0.9488841
H	-1.2664160	2.5987535	-0.9110338
H	-0.6438190	1.4173551	-2.0208166
C	-1.5935686	0.5500471	-0.2756666
C	-1.8450554	-0.6598141	-0.9322069
C	-2.1098297	0.7432229	1.0129401
C	-2.5752705	-1.6707047	-0.3048739
H	-1.4779070	-0.8109739	-1.9440565
C	-2.8363977	-0.2668882	1.6428354
H	-1.9506050	1.6897354	1.5238456
C	-3.0645682	-1.4792771	0.9878408
H	-2.7626198	-2.6039201	-0.8276024
H	-3.2293024	-0.1045959	2.6421181
H	-3.6306532	-2.2653304	1.4785727

13vb$^+$: $CH_3CHCH_2SiMe_3^+$
22
Energy = -527.1901644473

Si	-0.6756234	-0.0462916	-0.0440338
C	0.9479098	1.2913626	-0.4769140
H	0.9142604	1.9066551	0.4218664
H	0.5430724	1.7271471	-1.3884367
C	1.8651918	0.2747666	-0.5739261
H	1.9772112	-0.2184928	-1.5390543

C -2.0263062 1.2189209 -0.2684551
H -2.9754181 0.7247019 -0.0214328
H -1.8992296 2.0736433 0.4021125
H -2.0817804 1.5745124 -1.3013382
C -0.6985403 -1.4084666 -1.3100082
H -0.6367295 -1.0128582 -2.3291926
H 0.1083312 -2.1326493 -1.1604407
H -1.6511668 -1.9449146 -1.2165563
C -0.4487707 -0.6027264 1.7139933
H -0.2600983 0.2393744 2.3874219
H -1.3853921 -1.0796141 2.0312476
H 0.3515857 -1.3389534 1.8283510
C 2.7115547 -0.2284312 0.5232259
H 2.6399274 -1.3194973 0.6074274
H 3.7548166 -0.0300424 0.2312321
H 2.5097412 0.2540293 1.4802404

14ab^{+}: (o-$Tol_3P)_2PdCH(CH_2Ph)CH_2SiMe_3^{+}$
119
Energy = -3196.039171330
Pd 0.1034594 -0.1800220 0.2131899
P -2.1901792 -0.7387824 -0.0704139
P 2.4566577 -0.1436578 0.2561147
C -2.3432552 -2.5600460 -0.1254185
C -1.6592193 -3.2670897 -1.1410033
C -3.1468728 -3.2509366 0.7909037
C -1.7752661 -4.6607999 -1.1693401
C -3.2534045 -4.6391016 0.7392351
H -3.7066301 -2.6965256 1.5371517
C -2.5548804 -5.3471731 -0.2384213
H -1.2482292 -5.2137671 -1.9431475
H -3.8798723 -5.1605520 1.4566860
H -2.6238089 -6.4301270 -0.2843713
C -3.2495477 -0.1437365 1.3001555
C -4.2955482 0.7531601 1.0215262
C -2.9123850 -0.4155126 2.6462958
C -5.0049499 1.3744334 2.0442379
H -4.5418542 0.9872312 -0.0073929
C -3.6402955 0.2222045 3.6596240
C -4.6744511 1.1093369 3.3730951
H -5.8039513 2.0684222 1.8014110
H -3.3775963 0.0171019 4.6945740
H -5.2142887 1.5937449 4.1813597
C -3.1445999 -0.3051176 -1.5844116
C -4.4338785 -0.8726850 -1.6486776
C -2.6798104 0.4799191 -2.6557892
C -5.2728772 -0.6381060 -2.7283977
H -4.7778830 -1.5033887 -0.8339543
C -3.5479908 0.7090555 -3.7374342
C -4.8272782 0.1701665 -3.7788990
H -6.2634959 -1.0817750 -2.7526044
H -3.1924999 1.3167356 -4.5659345
H -5.4718626 0.3666077 -4.6306561
C 3.4617993 -0.8332459 1.6367301
C 4.8201030 -1.0461595 1.3232981
C 2.9821045 -1.1724207 2.9142592
C 5.7028825 -1.5618179 2.2610292
H 5.1831227 -0.7999020 0.3299009
C 3.8929745 -1.6979410 3.8475569
C 5.2333082 -1.8880711 3.5372760
H 6.7457024 -1.7125774 1.9991984
H 3.5251368 -1.9669382 4.8346975
H 5.9100883 -2.2970272 4.2818545
C 2.9479469 -1.2906732 -1.0791679
C 3.8402124 -0.9122082 -2.0894627
C 2.4389097 -2.6084670 -1.0383213
C 4.2106581 -1.8157670 -3.0837908
H 4.2583216 0.0892648 -2.0892607
C 2.8220897 -3.4975355 -2.0466599
C 3.6919474 -3.1099496 -3.0664989
H 4.9014770 -1.5087769 -3.8631955
H 2.4342608 -4.5129978 -2.0257888
H 3.9686144 -3.8207648 -3.8395451
C 3.2450299 1.4731879 -0.0717822
C 4.0928776 2.0267075 0.9049595
C 2.9299288 2.2285899 -1.2263027
C 4.6530906 3.2893103 0.7385291
H 4.3171284 1.4630440 1.8032452
C 3.4996945 3.5014874 -1.3665589
C 4.3587184 4.0297034 -0.4063888
H 5.3052962 3.6943650 1.5058251
H 3.2586310 4.0831629 -2.2526344
H 4.7861731 5.0180673 -0.5463756
C -1.2904797 1.0467772 -2.7414962
H -0.6977319 0.7953573 -1.8558734
H -0.7704486 0.6439326 -3.6174602
H -1.3122115 2.1359020 -2.8505609
C -1.8120083 -1.3626608 3.0404641
H -1.3042680 -1.0073592 3.9407683
H -2.2066885 -2.3618924 3.2550552
H -1.0694641 -1.4714637 2.2424039
C -0.8690750 -2.5662854 -2.2145963
H -0.1726175 -3.2519508 -2.7016952
H -1.5385918 -2.1555879 -2.9792138
H -0.2882843 -1.7196483 -1.8157210
C 2.0288043 1.7172810 -2.3184003
H 2.6115435 1.3517280 -3.1707688
H 1.3790954 2.5167407 -2.6838112
H 1.4036726 0.8889701 -1.9723857
C 1.5573825 -3.0914762 0.0828188
H 0.7573791 -2.3657437 0.3323815
H 1.0659906 -4.0320333 -0.1726529
H 2.1338303 -3.2351734 1.0035732
C 1.5461934 -1.0312199 3.3318005
H 1.4356111 -0.2702491 4.1121461
H 0.9121882 -0.7482186 2.4853371
H 1.1736401 -1.9765916 3.7385812
C -1.4280806 2.4093382 0.4727506
H -1.8762775 1.9156382 -0.3981192
H -2.0792158 2.2039802 1.3274453
C 0.4614247 1.9691875 2.1371749
H 1.4421856 1.4871136 2.2041666

H	-0.2199537	1.4233091	2.7976316
C	-0.0490012	1.8864917	0.7076852
H	0.6839910	2.2345106	-0.0248482
Si	-1.7052956	4.2394336	-0.0709599
C	-3.3201376	4.1604733	-1.0426752
H	-4.1401115	3.8096978	-0.4040942
H	-3.5949725	5.1522500	-1.4213285
H	-3.2525134	3.4795363	-1.8991782
C	-1.9436084	5.4248095	1.3613448
H	-1.0148728	5.6318697	1.8979539
H	-2.3343718	6.3747299	0.9737723
H	-2.6719054	5.0319128	2.0799347
C	-0.3065871	4.7953742	-1.1982698
H	0.6580815	4.8024420	-0.6810580
H	-0.2167767	4.1463623	-2.0766518
H	-0.4996912	5.8128280	-1.5593016
C	0.6095011	3.3914278	2.6452300
C	-0.0630045	3.8114580	3.7970162
C	1.4751359	4.2827524	2.0003340
C	0.1358312	5.0958933	4.3058536
H	-0.7399509	3.1259331	4.3014139
C	1.6700072	5.5686943	2.5010237
H	2.0124554	3.9609513	1.1134420
C	1.0029808	5.9782944	3.6586252
H	-0.3897143	5.4088742	5.2036189
H	2.3459899	6.2500211	1.9918601
H	1.1555214	6.9794819	4.0510971

14vb$^+$: (o-Tol$_3$P)$_2$PdCH(CH$_3$)CH$_2$SiMe$_3$$^+$

109

Energy = -2964.842796876

Pd	0.1490323	-0.1989118	0.1769772
P	-2.1528801	-0.6833010	-0.1238667
P	2.4859431	-0.2231847	0.1573418
C	-2.3207165	-2.4890933	-0.3768412
C	-1.5812941	-3.1101907	-1.4100555
C	-3.1939858	-3.2491467	0.4123529
C	-1.7127165	-4.4926905	-1.5784686
C	-3.3176604	-4.6233369	0.2195709
H	-3.7876995	-2.7583721	1.1775752
C	-2.5628184	-5.2492295	-0.7720437
H	-1.1405075	-4.9796164	-2.3643387
H	-3.9977168	-5.1978260	0.8413391
H	-2.6414916	-6.3215830	-0.9254364
C	-3.1337268	-0.2562504	1.3607578
C	-4.1386896	0.7216200	1.2646139
C	-2.7768636	-0.7606892	2.6338119
C	-4.8013344	1.1891714	2.3954214
H	-4.3957178	1.1324530	0.2948287
C	-3.4620242	-0.2789082	3.7566780
C	-4.4643413	0.6823639	3.6500746
H	-5.5715728	1.9478003	2.2941331
H	-3.1894600	-0.6660235	4.7354438
H	-4.9712525	1.0393537	4.5417510
C	-3.1782222	-0.0754345	-1.5278672
C	-4.4991323	-0.5691702	-1.5467050
C	-2.7417030	0.7721630	-2.5633825
C	-5.3938170	-0.2015993	-2.5409801
H	-4.8243113	-1.2475650	-0.7634161
C	-3.6668819	1.1368293	-3.5573994
C	-4.9746995	0.6700972	-3.5509413
H	-6.4071942	-0.5911722	-2.5308003
H	-3.3337595	1.7939512	-4.3570826
H	-5.6628141	0.9715611	-4.3353051
C	3.5222748	-0.5309897	1.6441934
C	4.9089505	-0.6010132	1.3980030
C	3.0348221	-0.7426627	2.9472948
C	5.8112993	-0.8443419	2.4227827
H	5.2787453	-0.4568694	0.3872120
C	3.9682693	-0.9861507	3.9708187
C	5.3340753	-1.0329024	3.7239462
H	6.8752403	-0.8887310	2.2110217
H	3.5981462	-1.1483953	4.9802322
H	6.0264887	-1.2231896	4.5388462
C	2.9942903	-1.6277953	-0.8970883
C	3.9954618	-1.4778560	-1.8646150
C	2.3951192	-2.8893688	-0.6881373
C	4.3901583	-2.5575772	-2.6523020
H	4.4682207	-0.5097358	-2.0012832
C	2.7996281	-3.9577461	-1.4940155
C	3.7816649	-3.7991820	-2.4726192
H	5.1645655	-2.4257641	-3.4019265
H	2.3390300	-4.9309464	-1.3443896
H	4.0735495	-4.6456752	-3.0871940
C	3.1825203	1.2994399	-0.5702567
C	3.9096610	2.1734045	0.2561119
C	2.8453316	1.7037266	-1.8847941
C	4.3195418	3.4224712	-0.2009718
H	4.1547179	1.8742915	1.2693520
C	3.2760492	2.9620026	-2.3234721
C	4.0046283	3.8174112	-1.5005940
H	4.8774919	4.0812204	0.4574444
H	3.0189234	3.2747667	-3.3324050
H	4.3161205	4.7900622	-1.8697399
C	-1.3306504	1.2725578	-2.7081101
H	-0.6867488	0.9116950	-1.9007003
H	-0.9091863	0.9245003	-3.6565927
H	-1.2965161	2.3673266	-2.7220845
C	-1.6992528	-1.7917306	2.8373437
H	-1.2363137	-1.6690066	3.8192669
H	-2.1018323	-2.8086762	2.7798593
H	-0.9176228	-1.7108049	2.0728459
C	-0.7115561	-2.3296700	-2.3594993
H	-0.0446089	-2.9909609	-2.9166596
H	-1.3247888	-1.7715270	-3.0768116
H	-0.0909557	-1.5860975	-1.8323798
C	2.0504076	0.8442174	-2.8307247
H	2.7029334	0.1895425	-3.4181422
H	1.4881685	1.4692065	-3.5282494
H	1.3475117	0.1976999	-2.2932455
C	1.3852766	-3.1240024	0.4028414
H	0.5461989	-2.4014598	0.3572039

```
H    0.9419460  -4.1190543   0.3282047
H    1.8376035  -3.0156509   1.3947027
C    1.5759244  -0.7652604   3.3130794
H    1.3594805  -0.0402390   4.1041200
H    0.9362420  -0.5409839   2.4521384
H    1.2994892  -1.7553557   3.6908573
C   -1.3091037   2.4224027   0.4826968
H   -1.8497168   2.0538996  -0.3944139
H   -1.8873387   2.1490001   1.3712888
C    0.7694562   2.1635298   1.8700412
H    0.8725564   3.2515825   1.9961402
H    1.7740182   1.7410403   1.9292638
H    0.1758728   1.7938612   2.7105982
C    0.0818958   1.8915484   0.5507328
H    0.6963197   2.1688009  -0.3105180
Si  -1.4160175   4.3366581   0.2793504
C   -3.0826764   4.6478214  -0.5371617
H   -3.8994459   4.2570950   0.0818099
H   -3.2556295   5.7208317  -0.6819538
H   -3.1428575   4.1605916  -1.5176053
C   -1.3574125   5.1921547   1.9530723
H   -0.3933875   5.0662775   2.4577669
H   -1.5272475   6.2688811   1.8280946
H   -2.1388449   4.8046079   2.6174172
C   -0.0043814   4.9159982  -0.8202959
H    0.9719574   4.7467755  -0.3512024
H   -0.0034443   4.3940444  -1.7843850
H   -0.0935715   5.9899541  -1.0233364
```

TS1^{+}: silylium ion transfer from **9b**$^{+}$ to **1a**
119
Energy = -3195.991982446

```
Pd   0.2637468   0.0925693  -0.1255425
P    2.3957679   0.9234345  -0.1256112
P   -0.8794538  -1.8964693  -0.1998127
C    3.2397055  -0.0555221   1.1847252
C    3.6488894   0.4286347   2.4485118
C    3.2889266  -1.4410638   0.9370315
C    4.1448337  -0.4952158   3.3824361
C    3.7953710  -2.3341661   1.8731500
H    2.8985127  -1.8263990   0.0007021
C    4.2367626  -1.8562423   3.1074308
H    4.4574290  -0.1230023   4.3555222
H    3.8215065  -3.3943734   1.6427578
H    4.6273515  -2.5382979   3.8573967
C    3.1804955   0.4208383  -1.7193343
C    4.2952104  -0.4265714  -1.7852736
C    2.6146115   0.9092402  -2.9232197
C    4.8446928  -0.8097285  -3.0064958
H    4.7489458  -0.7886125  -0.8698947
C    3.1903549   0.5223466  -4.1392946
C    4.2894666  -0.3329465  -4.1930271
H    5.7061764  -1.4712230  -3.0256438
H    2.7514430   0.8939101  -5.0623466
H    4.7061688  -0.6241790  -5.1531532
C    2.9382618   2.6788966  -0.0256724
C    1.9014785   3.6163253   0.0654656
C    4.2765841   3.1278360  -0.0932313
C    2.1507870   4.9854834   0.1039148
H    0.8851046   3.2405761   0.1001576
C    4.5109421   4.5084404  -0.0483870
C    3.4703743   5.4326050   0.0487173
H    1.3291305   5.6926086   0.1752280
H    5.5386311   4.8626734  -0.0853325
H    3.6924094   6.4955348   0.0814167
C   -2.0603638  -2.3399264  -1.5398831
C   -2.1850612  -3.6933354  -1.8956261
C   -2.8142855  -1.3677998  -2.2301131
C   -3.0470504  -4.0970155  -2.9106233
H   -1.5926480  -4.4362206  -1.3692289
C   -3.6709349  -1.7928187  -3.2557450
C   -3.7946415  -3.1375535  -3.5961935
H   -3.1301381  -5.1491696  -3.1673258
H   -4.2453848  -1.0453488  -3.7975622
H   -4.4658141  -3.4349183  -4.3971537
C    0.4210207  -3.1699478  -0.4512375
C    0.6534639  -4.1653156   0.5069540
C    1.2290001  -3.1158665  -1.6129346
C    1.6922255  -5.0824504   0.3541895
H    0.0103245  -4.2274234   1.3794894
C    2.2718089  -4.0396213  -1.7410172
C    2.5136875  -5.0119009  -0.7698918
H    1.8579546  -5.8428500   1.1121502
H    2.9065341  -3.9877663  -2.6224150
H    3.3346470  -5.7123451  -0.8953899
C   -1.7505971  -2.4279420   1.3264369
C   -3.0703226  -2.9010092   1.2515719
C   -1.1561527  -2.2289355   2.6001934
C   -3.8029818  -3.1879718   2.4011142
H   -3.5324548  -3.0366953   0.2796693
C   -1.9091056  -2.5368154   3.7405386
C   -3.2174490  -3.0095464   3.6538977
H   -4.8237545  -3.5496441   2.3155042
H   -1.4551778  -2.3861629   4.7172889
H   -3.7763301  -3.2300429   4.5590887
C    5.4525736   2.1940040  -0.2034311
H    5.3158099   1.2948457   0.4042518
H    6.3693070   2.6975554   0.1154428
H    5.5887856   1.8626988  -1.2388636
C    1.4044399   1.8022374  -2.9428521
H    1.0529544   1.9549221  -3.9665318
H    1.6123435   2.7804261  -2.4953287
H    0.5928923   1.3493984  -2.3482439
C    3.5726289   1.8745687   2.8756361
H    2.6403563   2.3485820   2.5628224
H    4.3880208   2.4681711   2.4485143
H    3.6462035   1.9398707   3.9647111
C    0.2299442  -1.6731408   2.7670865
H    1.0013499  -2.4023393   2.4994738
H    0.4013052  -1.3549776   3.7985790
H    0.3893485  -0.8141161   2.0883413
C    0.9839996  -2.1165683  -2.7084310
```

H	1.8575855	-2.0259540	-3.3573193
H	0.7601753	-1.1260006	-2.2822821
H	0.1193570	-2.4070916	-3.3169054
C	-2.7314133	0.0981705	-1.9096040
H	-3.2955062	0.3303165	-0.9999058
H	-1.6891149	0.3977483	-1.7317657
H	-3.1494674	0.6985865	-2.7211243
C	-2.9488550	3.8652018	2.6157320
H	-2.1622826	4.6162779	2.5942056
H	-3.1092701	3.3440232	3.5578489
C	-5.2065022	3.1368124	1.7072512
H	-5.9747797	3.9016595	1.9184155
H	-5.2210772	2.4412960	2.5501546
C	-3.9287682	3.8961966	1.6654316
H	-3.7734063	4.5344081	0.7970924
Si	-1.8134151	2.2787002	1.5379821
C	-0.3099995	2.4876546	2.6224286
H	0.1470305	3.4762790	2.5321912
H	-0.5569416	2.2961519	3.6712513
H	0.4153729	1.7342915	2.2883797
C	-2.8131153	0.7730402	1.9284441
H	-2.1508278	-0.0953465	1.8831272
H	-3.2238419	0.8221275	2.9426019
H	-3.6269263	0.6113151	1.2199673
C	-1.7162692	2.9022096	-0.2147304
H	-2.6761329	2.7846844	-0.7267131
H	-1.4190739	3.9559071	-0.2539906
H	-0.9681744	2.3045240	-0.7542063
C	-5.5696664	2.4272214	0.4091388
C	-6.0038197	1.0979261	0.4544429
C	-5.4981768	3.0754412	-0.8294181
C	-6.3518159	0.4233825	-0.7171224
H	-6.0756240	0.5891170	1.4124145
C	-5.8457688	2.4019693	-2.0010383
H	-5.1830047	4.1141135	-0.8889925
C	-6.2712045	1.0736687	-1.9483195
H	-6.6823466	-0.6097426	-0.6650261
H	-5.7846198	2.9179116	-2.9546617
H	-6.5373699	0.5495282	-2.8611743

TS2^{+}: β-hydride elimination from **14ab**$^{+}$ to form **3ab**

119

Energy = -3196.026554609

Pd	0.1011930	0.4623453	-0.1045580
P	-2.2487159	0.6338606	-0.0905150
P	1.0190037	-1.7631618	0.2001787
C	-3.3216612	-0.5554518	0.8107215
C	-3.5052411	-1.8729205	0.3348561
C	-3.9796255	-0.1440361	1.9802628
C	-4.3363307	-2.7328673	1.0615580
C	-4.7909408	-1.0212703	2.6943577
H	-3.8738937	0.8761523	2.3292986
C	-4.9684006	-2.3258450	2.2335081
H	-4.4765897	-3.7465503	0.6960296
H	-5.2852359	-0.6823898	3.5997590
H	-5.5994362	-3.0204213	2.7803516
C	-2.6939921	2.2670317	0.6425886
C	-3.4458400	3.1772738	-0.1178046
C	-2.2541474	2.6360890	1.9323980
C	-3.7643712	4.4378804	0.3811295
H	-3.7828791	2.8990596	-1.1098389
C	-2.6113811	3.8977638	2.4248407
C	-3.3527110	4.7972591	1.6645165
H	-4.3391415	5.1289163	-0.2282694
H	-2.2862799	4.1754355	3.4242348
H	-3.6065430	5.7722581	2.0701490
C	-3.0358930	0.7076973	-1.7526999
C	-4.3994394	0.3669801	-1.8112496
C	-2.3776157	1.1207742	-2.9333552
C	-5.1056156	0.3913976	-3.0089680
H	-4.9143330	0.0749810	-0.9024051
C	-3.1059386	1.1193571	-4.1320880
C	-4.4488870	0.7570171	-4.1829748
H	-6.1571498	0.1214192	-3.0211886
H	-2.5976009	1.4234739	-5.0434057
H	-4.9787092	0.7683995	-5.1309418
C	2.1106122	-2.0980924	1.6514493
C	2.7185214	-3.3701628	1.6608030
C	2.3173640	-1.2300488	2.7395435
C	3.5660243	-3.7622333	2.6870752
H	2.5222813	-4.0578128	0.8435809
C	3.1891768	-1.6412806	3.7634728
C	3.8154226	-2.8808185	3.7428690
H	4.0280034	-4.7445349	2.6640406
H	3.3572752	-0.9671871	4.5998230
H	4.4824462	-3.1664503	4.5510687
C	-0.0346614	-3.2608180	0.3508913
C	0.0879573	-4.3230863	-0.5555501
C	-0.8943838	-3.3823878	1.4627383
C	-0.6567262	-5.4887124	-0.3940283
H	0.7843381	-4.2476788	-1.3839494
C	-1.6292338	-4.5640981	1.6094137
C	-1.5249443	-5.6071298	0.6920874
H	-0.5532761	-6.2992085	-1.1092937
H	-2.2985464	-4.6579682	2.4605119
H	-2.1121189	-6.5107040	0.8282604
C	2.1018440	-2.1205791	-1.2410692
C	3.4909654	-2.2319696	-1.0707191
C	1.5668148	-2.1380972	-2.5527275
C	4.3424288	-2.4024694	-2.1598186
H	3.9180077	-2.1723156	-0.0767328
C	2.4404836	-2.3229647	-3.6314817
C	3.8140852	-2.4617094	-3.4478495
H	5.4129176	-2.4847918	-1.9981361
H	2.0267998	-2.3378253	-4.6365494
H	4.4664619	-2.5971440	-4.3052895
C	-0.9583224	1.6173874	-2.9832621
H	-0.2727581	0.9472687	-2.4499613
H	-0.6236407	1.7063260	-4.0197776
H	-0.8724572	2.6039148	-2.5130264
C	-1.4113587	1.7417199	2.8019108

H	-0.4310340	2.1996666	2.9708869
H	-1.8806723	1.5883520	3.7793862
H	-1.2497302	0.7604828	2.3492367
C	-2.8842956	-2.3670906	-0.9398920
H	-2.6873947	-3.4402338	-0.8801686
H	-3.5471933	-2.1839620	-1.7933481
H	-1.9429598	-1.8541601	-1.1429940
C	0.1045710	-1.9315826	-2.8356217
H	-0.4703324	-2.8539165	-2.7016639
H	-0.0456453	-1.5839430	-3.8605182
H	-0.3193340	-1.1840761	-2.1526753
C	-1.0028243	-2.3097280	2.5107341
H	-0.8430076	-1.3160401	2.0779309
H	-1.9887981	-2.3232196	2.9813028
H	-0.2453947	-2.4561684	3.2899263
C	1.5880812	0.0676879	2.9344487
H	2.2303085	0.8164021	3.4020614
H	1.1953806	0.4769473	1.9960257
H	0.7308961	-0.0865259	3.5995128
C	2.6700336	1.0272327	-1.9795622
H	2.7850442	-0.0322170	-2.2108094
H	1.9640916	1.4587671	-2.6987352
C	1.4725590	2.4182417	-0.2725865
H	1.1150567	2.9856638	-1.1316082
H	-0.3382413	1.9647286	-0.2814324
C	2.2173055	1.2689737	-0.5774627
H	2.8136680	0.8223618	0.2166019
Si	4.3782950	1.8484233	-2.2637241
C	4.9366382	1.3992531	-4.0010450
H	4.2226503	1.7572649	-4.7521233
H	5.9129079	1.8458449	-4.2246568
H	5.0272316	0.3126975	-4.1113953
C	4.1868212	3.7100040	-2.0715959
H	3.8777344	3.9805380	-1.0549980
H	5.1381845	4.2171341	-2.2727846
H	3.4413272	4.1102956	-2.7691547
C	5.5871224	1.1918749	-0.9797996
H	5.2939476	1.4822222	0.0361839
H	5.6505983	0.0987547	-1.0136510
H	6.5920760	1.5928876	-1.1575726
C	1.5461419	3.2020869	0.9769144
C	0.5999968	4.2119007	1.2120004
C	2.5992825	3.0386401	1.8887725
C	0.6863528	5.0168742	2.3431051
H	-0.2160479	4.3514095	0.5075356
C	2.6823075	3.8395001	3.0255205
H	3.3691698	2.2969902	1.6995811
C	1.7236981	4.8273969	3.2596435
H	-0.0575537	5.7890184	2.5129824
H	3.5042594	3.7023038	3.7217931
H	1.7919219	5.4539447	4.1439077

TS3^{+}: β-hydride elimination from **14ab**$^{+}$ to form **4ab**
119
Energy = -3196.014761569

Pd	-0.4931289	0.0125356	0.1533038
P	1.8127882	0.7967327	-0.0642963
P	-0.7786238	-2.3394940	0.0371455
C	3.2345488	-0.3178871	-0.3935041
C	3.6241321	-1.2393588	0.6014620
C	4.0038513	-0.1758994	-1.5563587
C	4.7713664	-2.0090856	0.3794734
C	5.1372289	-0.9588717	-1.7611073
H	3.7244840	0.5639448	-2.2994321
C	5.5207750	-1.8830159	-0.7881943
H	5.0740488	-2.7231941	1.1408157
H	5.7190763	-0.8406741	-2.6702694
H	6.4036932	-2.4982088	-0.9352634
C	1.8136391	1.9630179	-1.4917652
C	2.1609249	3.3133314	-1.3302169
C	1.3561174	1.5215571	-2.7582537
C	2.0562366	4.2223092	-2.3809020
H	2.5209082	3.6679581	-0.3716362
C	1.2489400	2.4537498	-3.7971939
C	1.5900636	3.7928736	-3.6211034
H	2.3332117	5.2598947	-2.2216689
H	0.8917977	2.1112713	-4.7651127
H	1.4945214	4.4917310	-4.4467366
C	2.5940691	1.7311277	1.3268127
C	3.8551275	2.2908638	1.0384340
C	2.0856724	1.8250403	2.6372915
C	4.5737403	2.9945306	1.9951596
H	4.2776492	2.1651854	0.0461935
C	2.8266695	2.5439998	3.5898167
C	4.0459823	3.1341901	3.2812234
H	5.5375789	3.4249248	1.7412331
H	2.4308117	2.6231825	4.5993257
H	4.5915755	3.6843449	4.0422581
C	-1.1734419	-3.1035297	-1.5935035
C	-0.9229120	-4.4868584	-1.6934159
C	-1.7186653	-2.4249481	-2.7026246
C	-1.1571844	-5.1857512	-2.8706153
H	-0.5308539	-5.0211677	-0.8349715
C	-1.9182338	-3.1453728	-3.8914084
C	-1.6407238	-4.5037662	-3.9880335
H	-0.9529110	-6.2510840	-2.9151099
H	-2.3159700	-2.6152205	-4.7531408
H	-1.8085881	-5.0281957	-4.9240743
C	0.5455205	-3.4515557	0.6575269
C	0.3780349	-4.0941633	1.8939500
C	1.7092633	-3.6946418	-0.1067954
C	1.3524248	-4.9542014	2.3929341
H	-0.5314859	-3.9399496	2.4633136
C	2.6643874	-4.5794032	0.4067334
C	2.5031276	-5.1989570	1.6434014
H	1.2058457	-5.4360809	3.3547173
H	3.5577097	-4.7725926	-0.1810877
H	3.2685911	-5.8725192	2.0174878
C	-2.2510657	-2.7849146	1.0556492
C	-3.2661880	-3.5707657	0.4892354
C	-2.4046623	-2.2978360	2.3729805

C	-4.4276511	-3.8657729	1.1991040
H	-3.1561201	-3.9470156	-0.5209337
C	-3.5752068	-2.6161411	3.0718148
C	-4.5847715	-3.3849798	2.4973981
H	-5.2022929	-4.4691637	0.7358145
H	-3.6885465	-2.2508793	4.0896631
H	-5.4847639	-3.6093212	3.0623085
C	0.8377889	1.1329955	3.1115777
H	0.2260666	0.7738552	2.2764217
H	1.1012709	0.2623016	3.7226592
H	0.2299459	1.7933769	3.7373464
C	1.0223685	0.0840957	-3.0431836
H	0.4988786	-0.3768254	-2.1960451
H	0.3895393	-0.0017423	-3.9287101
H	1.9294410	-0.5045920	-3.2181193
C	2.8802864	-1.3802542	1.9009730
H	2.9823951	-2.3930698	2.2977729
H	3.2718151	-0.6773852	2.6451583
H	1.8125392	-1.1695955	1.7749640
C	-1.3726542	-1.4421514	3.0585240
H	-1.3131596	-1.6876985	4.1230901
H	-1.6410633	-0.3825441	2.9800535
H	-0.3755788	-1.5620938	2.6271992
C	1.9411104	-3.0868730	-1.4620413
H	1.4501094	-2.1167498	-1.5530083
H	3.0102877	-2.9501868	-1.6400864
H	1.5402110	-3.7315139	-2.2521335
C	-2.1765683	-0.9951916	-2.6833238
H	-3.2385151	-0.9466940	-2.4217756
H	-1.6325379	-0.3924961	-1.9478173
H	-2.0633455	-0.5379683	-3.6695450
C	-2.1472893	1.5580115	0.6364574
H	-2.0058363	-0.3769199	0.2904215
H	-2.2037092	1.5359031	1.7298911
C	-0.3505590	3.3265236	0.9587619
H	-0.3698209	3.0596739	2.0156359
H	0.6873959	3.4502524	0.6489104
C	-1.0605828	2.2785024	0.1322671
H	-1.0167803	2.4519519	-0.9432996
Si	-3.8386203	1.4974801	-0.2255377
C	-4.6731223	-0.1687162	-0.0166982
H	-5.6954503	-0.1365286	-0.4120405
H	-4.1453041	-0.9854877	-0.5162640
H	-4.7345414	-0.4279070	1.0469545
C	-4.8300446	2.7981378	0.7075764
H	-4.3369196	3.7755554	0.6664734
H	-5.8278544	2.9001325	0.2630882
H	-4.9594310	2.5267797	1.7617714
C	-3.6256127	2.0678419	-2.0027724
H	-2.8558497	1.5208456	-2.5532785
H	-4.5681824	1.9630103	-2.5525709
H	-3.3526794	3.1300066	-2.0102944
C	-1.1128109	4.6251281	0.7508825
C	-1.0464215	5.3011791	-0.4741882
C	-1.9485895	5.1231619	1.7558094
C	-1.7982121	6.4564453	-0.6872268
H	-0.4027566	4.9203941	-1.2630087
C	-2.6996316	6.2808645	1.5463587
H	-2.0140865	4.6007577	2.7073634
C	-2.6289795	6.9486423	0.3223782
H	-1.7376162	6.9725144	-1.6414034
H	-3.3419391	6.6580847	2.3371341
H	-3.2165264	7.8468733	0.1560772

TS4^{+}: hydropalladation of **5b** and **11**$^{+}$
109
Energy = -2964.825868637

Pd	0.0150496	0.7354828	-0.4696363
P	2.3384779	0.5436102	-0.1112303
P	-1.3449896	-1.2099912	0.0289674
C	3.2249487	-1.0510803	-0.2997201
C	3.0765399	-2.0944052	0.6411812
C	4.0657459	-1.2328856	-1.4093564
C	3.7661253	-3.2918767	0.4201882
C	4.7317988	-2.4376584	-1.6153283
H	4.2182303	-0.4194897	-2.1089751
C	4.5769429	-3.4760575	-0.6968560
H	3.6518441	-4.0950566	1.1431824
H	5.3720836	-2.5582953	-2.4837957
H	5.0908912	-4.4213026	-0.8453760
C	3.1676195	1.6572001	-1.3254720
C	4.0518378	2.6387121	-0.8516292
C	2.8689549	1.5966041	-2.7065082
C	4.6243869	3.5673991	-1.7177254
H	4.2867060	2.6849047	0.2058775
C	3.4537487	2.5428782	-3.5571765
C	4.3200128	3.5226668	-3.0775512
H	5.3001120	4.3225171	-1.3275620
H	3.2218740	2.5015107	-4.6187655
H	4.7548899	4.2449096	-3.7622833
C	2.9213585	1.1839888	1.5092572
C	4.1272045	0.6560674	2.0029400
C	2.2536632	2.1902100	2.2458847
C	4.6593904	1.0722100	3.2190108
H	4.6556427	-0.0940860	1.4243193
C	2.8005396	2.5796555	3.4765579
C	3.9819809	2.0315828	3.9693692
H	5.5917575	0.6447643	3.5749133
H	2.2840743	3.3443807	4.0508983
H	4.3737756	2.3597757	4.9276337
C	-2.4093619	-1.9359518	-1.2980546
C	-3.2150502	-3.0152162	-0.8784515
C	-2.4348903	-1.5471378	-2.6504953
C	-4.0672215	-3.6701776	-1.7553313
H	-3.1686702	-3.3403335	0.1563742
C	-3.3045640	-2.2249330	-3.5234495
C	-4.1180422	-3.2640517	-3.0919943
H	-4.6812897	-4.4930427	-1.4022262
H	-3.3205411	-1.9277554	-4.5692241
H	-4.7763092	-3.7669302	-3.7944644
C	-0.6187572	-2.7773392	0.6381477
C	-1.0090807	-3.3253913	1.8674215

C 0.2677483 -3.4842170 -0.2023675
C -0.5111002 -4.5562134 2.2877767
H -1.7207886 -2.7948711 2.4919344
C 0.7497387 -4.7222772 0.2366281
C 0.3754943 -5.2562986 1.4681584
H -0.8189050 -4.9661205 3.2450383
H 1.4373977 -5.2699234 -0.4025169
H 0.7707610 -6.2171347 1.7848161
C -2.5547833 -0.7136322 1.3200251
C -3.9265418 -0.6781664 1.0234990
C -2.1083341 -0.2207035 2.5710595
C -4.8542367 -0.1979630 1.9451566
H -4.2767175 -1.0191590 0.0564553
C -3.0597061 0.2512687 3.4838638
C -4.4205553 0.2628112 3.1862836
H -5.9091549 -0.1823909 1.6886952
H -2.7147606 0.6288831 4.4429876
H -5.1328765 0.6405264 3.9136522
C 1.0227458 2.9119160 1.7678166
H 0.2320937 2.2133051 1.4650576
H 0.6311054 3.5568915 2.5584911
H 1.2464403 3.5385047 0.8967986
C 1.9846246 0.5363699 -3.3076462
H 1.3543226 0.0601639 -2.5484359
H 1.3352659 0.9600007 -4.0791945
H 2.5828603 -0.2542265 -3.7749840
C 2.2535976 -1.9459110 1.8878221
H 1.8407772 -2.9097456 2.1942676
H 2.8617278 -1.5538955 2.7112435
H 1.4289164 -1.2486793 1.7333413
C -0.6549268 -0.1684379 2.9511921
H -0.2881620 -1.1476611 3.2768310
H -0.4907565 0.5421386 3.7646196
H -0.0386765 0.1388135 2.0959255
C 0.6673632 -2.9686417 -1.5584706
H 0.7367982 -1.8749082 -1.5631328
H 1.6374541 -3.3732519 -1.8564271
H -0.0737329 -3.2534255 -2.3148871
C -1.5563445 -0.4808018 -3.2445480
H -2.1417197 0.3978327 -3.5394195
H -0.7829794 -0.1593979 -2.5402610
H -1.0603401 -0.8562888 -4.1448607
C -2.5741282 2.4884469 0.4037781
H -2.8656369 1.7489475 1.1541544
H -1.8518783 3.1738025 0.8624527
C -1.0094652 2.4564726 -1.5760752
H -0.6492218 3.4403336 -1.2909190
H -0.8386087 2.1729199 -2.6085698
C -2.0047771 1.8497944 -0.8139988
H -2.5939188 1.0630539 -1.2805114
Si -4.1525938 3.4948624 -0.0168767
C -4.9120391 4.0319739 1.6160432
H -5.8161986 4.6293919 1.4487208
H -5.1878786 3.1610612 2.2215003
H -4.2100388 4.6404478 2.1984541
C -3.6519402 4.9828030 -1.0518554
H -3.1906260 4.6769854 -1.9985179
H -4.5278360 5.5967332 -1.2940086
H -2.9360654 5.6178153 -0.5162862
C -5.3402819 2.4033460 -0.9848350
H -5.6043399 1.5021830 -0.4209467
H -6.2676334 2.9467758 -1.2021459
H -4.9100454 2.0895738 -1.9433887
H 0.7462984 2.0593501 -0.8851432

TS5^{+}: proton transfer from **11**$^{+}$ to **5b**
109
Energy = -2964.819327777
Pd -0.4132526 0.4562693 -0.2756005
P -2.3375211 -0.8228525 -0.0684988
P 1.3940612 1.8928631 -0.0998835
C -3.7622323 0.1682766 -0.6346520
C -3.9148358 1.5004076 -0.1815293
C -4.6774251 -0.3693277 -1.5496431
C -4.9757724 2.2547562 -0.6935807
C -5.7289844 0.4001949 -2.0438336
H -4.5635011 -1.3988095 -1.8761038
C -5.8729890 1.7204013 -1.6189260
H -5.0993698 3.2802933 -0.3545347
H -6.4273836 -0.0302090 -2.7553140
H -6.6834452 2.3338238 -2.0018766
C -2.3717324 -2.3722493 -1.0292115
C -2.6470487 -3.5866688 -0.3816637
C -2.0206214 -2.3708475 -2.4008617
C -2.5565658 -4.7981220 -1.0627430
H -2.9340867 -3.5793498 0.6653705
C -1.9357129 -3.6009409 -3.0639161
C -2.1919914 -4.8045715 -2.4091758
H -2.7701390 -5.7278553 -0.5439947
H -1.6607725 -3.6079113 -4.1158160
H -2.1118452 -5.7427519 -2.9505374
C -2.7640423 -1.2943925 1.6475300
C -4.0395399 -0.9878731 2.1459242
C -1.8122887 -1.9073057 2.4956646
C -4.3786269 -1.2615857 3.4688565
H -4.7724902 -0.5277543 1.4907769
C -2.1728030 -2.1698875 3.8219937
C -3.4376126 -1.8496972 4.3131917
H -5.3707129 -1.0144268 3.8346167
H -1.4426556 -2.6368186 4.4784629
H -3.6859696 -2.0614564 5.3491208
C 2.4202876 2.5096850 -1.4938925
C 3.3189355 3.5483180 -1.1781504
C 2.3378292 2.0401610 -2.8191448
C 4.1490096 4.1027909 -2.1425726
H 3.3615141 3.9214660 -0.1589005
C 3.1837283 2.6196058 -3.7808279
C 4.0812199 3.6297067 -3.4565450
H 4.8375856 4.8987556 -1.8755739
H 3.1205059 2.2661833 -4.8072738
H 4.7194793 4.0553719 -4.2255118
C 0.8257518 3.4712963 0.6250862

C	1.3783084	3.9714176	1.8113521
C	-0.1859090	4.1962162	-0.0467919
C	0.9229378	5.1684422	2.3604938
H	2.1765408	3.4227190	2.3029845
C	-0.6315225	5.3921368	0.5260970
C	-0.0933046	5.8760589	1.7187657
H	1.3599405	5.5427894	3.2814612
H	-1.4129869	5.9544321	0.0207171
H	-0.4624740	6.8066559	2.1400912
C	2.5976092	1.1730629	1.0741635
C	3.9243303	0.9577155	0.6655430
C	2.1759806	0.7092899	2.3479631
C	4.8415867	0.3150346	1.4946243
H	4.2437945	1.3058063	-0.3108628
C	3.1151452	0.0629117	3.1594548
C	4.4347087	-0.1337103	2.7502509
H	5.8637587	0.1671521	1.1589220
H	2.7982023	-0.2942752	4.1360128
H	5.1367313	-0.6372894	3.4084378
C	-0.4497046	-2.3117123	2.0072479
H	0.0126456	-1.5018913	1.4279513
H	0.2039243	-2.5647262	2.8460097
H	-0.5163782	-3.1867719	1.3485807
C	-1.7699971	-1.0992096	-3.1645401
H	-1.1496115	-1.2868692	-4.0446845
H	-2.7124474	-0.6515036	-3.5009835
H	-1.2742972	-0.3477412	-2.5314824
C	-3.0153991	2.1041336	0.8632176
H	-3.1246790	3.1901269	0.9005187
H	-3.2345627	1.6972645	1.8572423
H	-1.9504355	1.8774057	0.6669895
C	0.7736165	0.8925281	2.8628944
H	0.5922733	1.9255906	3.1778036
H	0.5859505	0.2362543	3.7158352
H	0.0334595	0.6699513	2.0751686
C	-0.7535005	3.7426518	-1.3663627
H	-1.0030616	2.6690927	-1.3526279
H	-1.6563003	4.3045414	-1.6179677
H	-0.0240981	3.8828564	-2.1733112
C	1.3820591	0.9699969	-3.2756475
H	1.9242565	0.0679547	-3.5822566
H	0.6744969	0.6988605	-2.4849822
H	0.8104831	1.3143019	-4.1434545
C	2.9113179	-2.6188909	0.5672096
H	3.6620642	-2.0619440	1.1392831
H	2.0685236	-2.9067386	1.2016289
C	1.2127351	-1.9413568	-1.1738497
H	0.5228009	-2.6970796	-0.8013696
H	1.1155271	-1.7423512	-2.2388571
H	0.6581543	-0.8798349	-0.7034325
C	2.5125387	-1.9475569	-0.6429948
H	3.2772425	-1.3759682	-1.1675451
Si	3.8070438	-4.2966980	0.1148277
C	4.3482231	-5.0153700	1.7605975
H	3.4888862	-5.2065238	2.4132742
H	4.8700723	-5.9669907	1.6026702
H	5.0327617	-4.3381171	2.2838982
C	2.5549832	-5.3925116	-0.7492302
H	2.1965603	-4.9378923	-1.6797136
H	3.0065829	-6.3591016	-1.0023142
H	1.6868781	-5.5874682	-0.1086695
C	5.2710778	-3.8983513	-0.9866441
H	4.9510208	-3.4919533	-1.9529857
H	5.9387684	-3.1688471	-0.5130861
H	5.8549488	-4.8050071	-1.1846084

TS6^{+}: β-silyl elimination of **14vb**$^{+}$

109

Energy = -2964.796640475

Pd	-0.1001156	-1.3202260	0.2148387
P	2.1213757	-0.0006953	-0.0167224
P	-1.8771770	0.3712793	0.0341872
C	2.3348448	1.8418909	-0.0135114
C	1.8537115	2.5973877	-1.1035653
C	3.1003601	2.4820999	0.9733752
C	2.1303405	3.9680209	-1.1504782
C	3.3517531	3.8503059	0.9173087
H	3.5408922	1.9071695	1.7775178
C	2.8598984	4.6013274	-0.1500927
H	1.7507262	4.5434712	-1.9906975
H	3.9444229	4.3191092	1.6972784
H	3.0525315	5.6687417	-0.2072149
C	3.3876091	-0.5131408	1.2333148
C	4.5287477	-1.2156230	0.8145977
C	3.2132701	-0.2640115	2.6166641
C	5.5094136	-1.6164042	1.7197311
H	4.6604008	-1.4544745	-0.2337583
C	4.2250173	-0.6479290	3.5055437
C	5.3704450	-1.3100216	3.0714900
H	6.3781748	-2.1609495	1.3621121
H	4.0868921	-0.4446769	4.5645427
H	6.1340329	-1.6033068	3.7858478
C	3.0371899	-0.3263987	-1.6049767
C	4.1995741	0.4556528	-1.7748998
C	2.6585928	-1.2094839	-2.6338313
C	4.9732950	0.3781059	-2.9237670
H	4.4976640	1.1400735	-0.9879298
C	3.4491692	-1.2632974	-3.7958736
C	4.5898003	-0.4873213	-3.9509293
H	5.8621157	0.9944085	-3.0195127
H	3.1424997	-1.9344354	-4.5943324
H	5.1729156	-0.5518182	-4.8649651
C	-2.7972624	0.6935978	1.6174045
C	-3.7572142	1.7239001	1.5426476
C	-2.5471810	0.0837727	2.8639766
C	-4.4833146	2.1296835	2.6534809
H	-3.9272041	2.2211404	0.5932958
C	-3.2864168	0.5175596	3.9777714
C	-4.2451664	1.5182308	3.8863252
H	-5.2196855	2.9220149	2.5592308
H	-3.0857610	0.0525901	4.9399388
H	-4.7949799	1.8276802	4.7703980

C	-1.5618528	2.1610912	-0.3643526
C	-2.1515544	2.7608900	-1.4880022
C	-0.8804911	2.9762212	0.5643532
C	-2.0418734	4.1294908	-1.7193838
H	-2.7325428	2.1656115	-2.1802253
C	-0.7913849	4.3510676	0.3207714
C	-1.3537294	4.9321495	-0.8104432
H	-2.5063002	4.5629840	-2.6002101
H	-0.2573643	4.9687725	1.0379609
H	-1.2631973	6.0015398	-0.9775134
C	-3.2077728	-0.0051764	-1.1992979
C	-4.5476667	-0.1267644	-0.7994173
C	-2.8955729	-0.1379755	-2.5727575
C	-5.5618246	-0.3827551	-1.7170530
H	-4.8113618	-0.0239915	0.2455388
C	-3.9311556	-0.3836243	-3.4827158
C	-5.2540382	-0.5088552	-3.0709004
H	-6.5863967	-0.4799991	-1.3707196
H	-3.6798603	-0.4886600	-4.5351527
H	-6.0356467	-0.7065288	-3.7985920
C	1.4668297	-2.1224389	-2.5864201
H	0.7069811	-1.7695331	-1.8769112
H	1.7693780	-3.1301293	-2.2789155
H	1.0055465	-2.2072631	-3.5738849
C	1.9528404	0.3353159	3.1778923
H	1.9496706	1.4275423	3.1220811
H	1.8292677	0.0530566	4.2265128
H	1.0823121	-0.0235161	2.6191578
C	1.1145444	1.9689605	-2.2465277
H	0.5835573	1.0727419	-1.9212045
H	1.8073421	1.6711455	-3.0420421
H	0.3867152	2.6676467	-2.6643692
C	-1.4963536	-0.0109067	-3.1061113
H	-1.2631818	1.0250773	-3.3712903
H	-1.3661024	-0.6199734	-4.0045378
H	-0.7637551	-0.3311148	-2.3584754
C	-0.3049114	2.4354868	1.8385564
H	0.5833451	3.0013504	2.1261356
H	-0.0263631	1.3853749	1.7278744
H	-1.0345688	2.4990386	2.6538082
C	-1.5317591	-0.9973537	3.1155170
H	-2.0231572	-1.9461167	3.3536108
H	-0.8834560	-1.1703707	2.2433527
H	-0.8972783	-0.7326297	3.9664844
C	0.3208903	-3.5776484	0.1796143
H	0.3423250	-3.8962062	-0.8582030
H	-0.1511362	-4.2647698	0.8683812
C	1.6347733	-3.0414621	2.2097156
H	0.7079893	-3.3164955	2.7208358
H	2.0619815	-2.1656750	2.6898384
H	2.3562866	-3.8590203	2.3442600
C	1.3942642	-2.8655113	0.7348721
H	2.2569991	-2.6471490	0.1112130
Si	-2.0473813	-3.0962645	-0.1267311
C	-2.2373950	-3.1069993	-2.0040795
H	-1.9057552	-4.0914966	-2.3543661
H	-3.2907689	-2.9829310	-2.2774166
H	-1.6580861	-2.3519868	-2.5296381
C	-3.5096732	-2.5299876	0.9286433
H	-3.6128580	-1.4753136	1.1586892
H	-4.4127384	-2.8577882	0.3974855
H	-3.4604411	-3.0839965	1.8721694
C	-2.1876421	-4.9509672	0.2808620
H	-3.2125597	-5.2477178	0.0177612
H	-1.4983382	-5.5783381	-0.2937755
H	-2.0503463	-5.1646264	1.3479676

TS7$^+$: silylium ion transfer from **13vb**$^+$ to **8**

109

Energy = -2964.791972141

Pd	0.0236221	0.4091912	-0.2308953
P	2.3351811	0.2090615	-0.1093526
P	-1.7685354	-0.9452662	0.1001069
C	2.5234510	-0.6904532	1.4800875
C	2.7385701	0.0044062	2.6936755
C	2.2476862	-2.0672665	1.5228121
C	2.7661312	-0.7295189	3.8862805
C	2.2702450	-2.7747856	2.7205183
H	1.9830971	-2.5895951	0.6116990
C	2.5538288	-2.1062892	3.9108546
H	2.9335254	-0.1945572	4.8184920
H	2.0504618	-3.8374143	2.7184300
H	2.5772863	-2.6439928	4.8545379
C	2.9308927	-0.9247529	-1.4337128
C	3.7216801	-2.0530264	-1.1731223
C	2.6057482	-0.6061552	-2.7734531
C	4.1507421	-2.8888014	-2.2022986
H	4.0086190	-2.2876777	-0.1543013
C	3.0458802	-1.4573971	-3.7928588
C	3.8011640	-2.5968294	-3.5196608
H	4.7571539	-3.7600054	-1.9714702
H	2.7873129	-1.2157448	-4.8211454
H	4.1216679	-3.2443944	-4.3309004
C	3.7705371	1.3814206	-0.1139298
C	5.0405279	0.8118295	0.0832871
C	3.6561185	2.7706890	-0.2987946
C	6.1885479	1.5935303	0.0979465
H	5.1251094	-0.2601257	0.2344168
C	4.8285030	3.5448240	-0.2970870
C	6.0806179	2.9735471	-0.1010236
H	7.1589363	1.1331304	0.2598253
H	4.7442002	4.6196109	-0.4423070
H	6.9689334	3.5989965	-0.0984848
C	-3.0061421	-1.4106710	-1.1907341
C	-3.8476598	-2.4980990	-0.8812727
C	-3.0986444	-0.8099440	-2.4624575
C	-4.7899475	-2.9692956	-1.7848145
H	-3.7499864	-2.9804365	0.0871215
C	-4.0578059	-1.3033145	-3.3647092
C	-4.8983198	-2.3604163	-3.0389978
H	-5.4287711	-3.8064696	-1.5191444
H	-4.1272679	-0.8454384	-4.3488703

H	-5.6261028	-2.7184863	-3.7617404
C	-1.1231323	-2.6266336	0.4738505
C	-1.3940563	-3.2468324	1.7001912
C	-0.3605281	-3.3106007	-0.5023990
C	-0.9002411	-4.5176373	1.9882499
H	-2.0096253	-2.7355902	2.4334179
C	0.1302549	-4.5832738	-0.1897852
C	-0.1269329	-5.1868744	1.0410374
H	-1.1185388	-4.9777972	2.9477191
H	0.7271321	-5.1072895	-0.9328262
H	0.2712690	-6.1748176	1.2548994
C	-2.7964992	-0.5092654	1.5647912
C	-4.1901048	-0.3790956	1.4443207
C	-2.1799269	-0.1742248	2.7973484
C	-4.9741305	0.0772413	2.5008101
H	-4.6699729	-0.6246594	0.5034375
C	-2.9894977	0.2820903	3.8465335
C	-4.3699732	0.4125282	3.7118834
H	-6.0489094	0.1715287	2.3739668
H	-2.5141965	0.5429592	4.7894236
H	-4.9667785	0.7729680	4.5449957
C	2.3352033	3.4742229	-0.4629359
H	1.5054842	2.7663228	-0.3910412
H	2.2789573	3.9786113	-1.4338073
H	2.2092425	4.2397219	0.3113867
C	1.8075953	0.6176830	-3.1321844
H	1.6461376	0.6760420	-4.2117302
H	2.3098835	1.5363755	-2.8067138
H	0.8300153	0.5926475	-2.6236012
C	2.8604459	1.5057996	2.7718284
H	2.1428174	1.9927706	2.1013156
H	3.8578527	1.8563564	2.4876965
H	2.6565687	1.8429010	3.7920773
C	-0.7006920	-0.3030684	3.0371864
H	-0.4358825	-1.3035130	3.3956197
H	-0.3655126	0.4206325	3.7847626
H	-0.1285750	-0.1458181	2.1099408
C	-0.0891836	-2.7273112	-1.8620170
H	0.7676589	-3.2152156	-2.3329410
H	0.1191394	-1.6502880	-1.7867388
H	-0.9608989	-2.8449359	-2.5170833
C	-2.2058705	0.3063906	-2.9377114
H	-2.7869520	1.2113394	-3.1469445
H	-1.4363965	0.5463443	-2.1934013
H	-1.7027062	0.0210390	-3.8676732
C	-2.3153541	5.2081199	0.3110680
H	-1.2825441	5.5135610	0.4607819
H	-2.9050712	5.0115582	1.2051445
C	-4.3886693	5.4371367	-1.1223056
H	-4.6033696	4.8701540	-2.0357089
H	-4.7354419	6.4614566	-1.3261967
H	-4.9502670	5.0345426	-0.2778816
C	-2.9341554	5.5103036	-0.8668580
H	-2.3133862	5.8351434	-1.7011931
Si	-1.7701089	3.0814695	-0.2034896
C	-0.8865292	2.9143485	1.4360814
H	-0.2479024	3.7748952	1.6528615
H	-1.6134960	2.7832319	2.2434765
H	-0.2497502	2.0113236	1.3967339
C	-3.4538201	2.3105507	-0.3738625
H	-3.3634688	1.2440551	-0.5901588
H	-4.0294965	2.4122321	0.5506033
H	-4.0143028	2.7654887	-1.1953539
C	-0.8012267	3.3405724	-1.7758492
H	-1.4849734	3.4413271	-2.6251736
H	-0.1783194	4.2382031	-1.7152800
H	-0.1443516	2.4844223	-1.9652429

10 References

15. Q. Wu, Z.-W. Qu, L. Omann, E. Irran, H. F. T. Klare, and M. Oestreich, “Cleavage of Unactivated Si–C(sp^3) Bonds with Reed's Carborane Acids: Formation of Known and Unknown Silylium Ions”, *Angewandte Chemie International Edition* **57**, (2018): 9176–9179.

41. L. Omann, B. Pudasaini, E. Irran, H. F. T. Klare, M.-H. Baik, and M. Oestreich, “Thermodynamic *versus* kinetic control in substituent redistribution reactions of silylium ions steered by the counteranion”, *Chemical Science* **9**, (2018): 5600–5607.

63. TURBOMOLE GmbH, TURBOMOLE V7.4, Program Package for Electronic Structure Calculations, Karlsruhe (Germany), 2019.

64. J. Tao, J. P. Perdew, V. N. Staroverov, and G. E. Scuseria, “Climbing the Density Functional Ladder: Nonempirical Meta-Generalized Gradient Approximation Designed for Molecules and Solids”, *Physical Review Letters* **91**, (2003): 146401.

65. S. Grimme, J. Antony, S. Ehrlich, and H. Krieg, “A consistent and accurate *ab initio* parametrization of density functional dispersion correction (DFT-D) for the 94 elements H-Pu”, *The Journal of Chemical Physics* **132**, (2010): 154104.

66. S. Grimme, S. Ehrlich, and L. Goerigk, “Effect of the Damping Function in Dispersion Corrected Density Functional Theory”, *Journal of Computational Chemistry* **32**, (2011): 1456–1465.

67. F. Weigend, M. Häser, H. Patzelt, and R. Ahlrichs, “RI-MP2: optimized auxiliary basis sets and demonstration of efficiency”, *Chemical Physics Letters* **294**, (1998): 143–152.

68. F. Weigend and R. Ahlrichs, “Balanced basis sets of split valence, triple zeta valence and quadruple zeta valence quality for H to Rn: Design and assessment of accuracy”, *Physical Chemistry Chemical Physics* **7**, (2005): 3297–3305.

69. A. Klamt and G. Schüürmann, “COSMO: a new approach to dielectric screening in solvents with explicit expressions for the screening energy and its gradient”, *Journal of the Chemical Society, Perkin Transactions 2* **22**, (1993): 799–805.

70. K. Eichkorn, F. Weigend, O. Treutler, and R. Ahlrichs, “Auxiliary basis sets for main row atoms and transition metals and their use to approximate Coulomb potentials”, *Theoretical Chemistry Accounts* **97**, (1997): 119–124.

71. F. Weigend, “Accurate Coulomb-fitting basis sets for H to Rn”, *Physical Chemistry Chemical Physics* **8**, (2006): 1057–1065.

72. P. Deglmann, K. May, F. Furche, and R. Ahlrichs, “Nuclear second analytical derivative calculations using auxiliary basis set expansions”, *Chemical Physics Letters* **384**, (2004): 103–107.

73. S. Grimme, “Supramolecular Binding Thermodynamics by Dispersion-Corrected Density Functional Theory”, *Chemistry – A European Journal* **18**, (2012): 9955–9964.

74. F. Eckert and A. Klamt, “Fast Solvent Screening via Quantum Chemistry: COSMO-RS Approach”, *AIChE Journal* **48**, (2002): 369–385.

75. COSMOlogic GmbH & Co. KG, COSMOtherm Version C3.0 Release 16.01, Solvation Thermodynamics Calculation Software, Leverkusen (Germany), 2015.

76. Y. Zhao and D. G. Truhlar, “Design of Density Functionals That Are Broadly Accurate for Thermochemistry, Thermochemical Kinetics, and Nonbonded Interactions”, *Journal of Physical Chemistry A* **109**, (2005): 5656–5667.

77. F. Weigend, F. Furche, and R. Ahlrichs, “Gaussian basis sets of quadruple zeta valence quality for atoms H–Kr”, *The Journal of Chemical Physics* **119**, (2003): 12753–12762.

78. L. Goerigk, A. Hansen, C. Bauer, S. Ehrlich, A. Najibi, and S. Grimme, “A look at the density functional theory zoo with the advanced GMTKN55 database for general main group thermochemistry, kinetics and noncovalent interactions”, *Physical Chemistry Chemical Physics* **19**, (2017): 32184–32215.

85. C. A. Reed, “H^+, CH_3^+, and R_3Si^+ Carborane Reagents: When Triflates Fail”, *Accounts of Chemical Research* **43**, (2010): 121–128.

86. Z. Xie, R. Bau, A. Benesi, and C. A. Reed, “The Silylium Ion (R_3Si^+) Problem: Effect of Alkyl Substituents R”, *Organometallics* **14**, (1995): 3933–3941.

87. T. He, G. Wang, P.-W. Long, S. Kemper, E. Irran, H. F. T. Klare, and M. Oestreich, “Intramolecular Friedel–Crafts alkylation with a silylium-ion-activated cyclopropyl group: formation of tricyclic ring systems from benzyl-substituted vinylcyclopropanes and hydrosilanes”, *Chemical Science* **12**, (2021): 569–575.

88. J. B. Lambert, S. Zhang, and S. M. Ciro, “Silyl Cations in the Solid and in Solution”, *Organometallics* **13**, (1994): 2430–2443.

89. H. Zuo, H. F. T. Klare, and M. Oestreich, “Intramolecular 7-*endo*-*dig*-Selective Carbosilylation of Internal Alkynes Involving Silylium-Ion Regeneration”, *Journal of Organic Chemistry* **88**, (2023): 4024–4027.

90. J. Dunn and A. P. Dobbs, “Synthesis and reactions of donor cyclopropanes: efficient routes to *cis*- and *trans*-tetrahydrofurans”, *Tetrahedron* **71**, (2015): 7386–7414.

91. Z. Li, C. Yang, H. Zheng, H. Qiu, and G. Lai, “Selective mono- and di-allylation and allenylation of chlorosilanes using indium”, *Journal of Organometallic Chemistry* **693**, (2008): 3771–3779.

92. Agilent Technologies, CrysAlisPro, Data Collection and Processing Software for Agilent X-ray Diffractometers, Yarnton, Oxfordshire (United Kingdom), 2012.

93. G. M. Sheldrick, “Phase Annealing in *SHELX-90*: Direct Methods for Larger Structures”, *Acta Crystallographica Section A: Foundations and Advances* **46**, (1990): 467–473.

94. G. M. Sheldrick, “A short history of *SHELX*”, *Acta Crystallographica Section A: Foundations of Crystallography* **64**, (2008): 112–122.

95. Cambridge Crystallographic Data Centre, Mercury 2024.3.0, Crystal Structure Visualization and Analysis Software, Cambridge (United Kingdom), 2024.

96. P. Gomes, C. Gosmini, and J. Périchon, “New Chemical Cross-Coupling between Aryl Halides and Allylic Acetates Using a Cobalt Catalyst”, *Organic Letters* **5**, (2003): 1043–1045.

97. C. Li, M. Li, J. Li, W. Wu, and H. Jiang, “Palladium-catalyzed oxidative allylation of bis[(pinacolato)boryl]methane: synthesis of homoallylic boronic esters”, *Chemical Communications* **54**, (2018): 66–69.

98. S. Lin, C.-X. Song, G.-X. Cai, W.-H. Wang, and Z.-J. Shi, “Intra/Intermolecular Direct Allylic Alkylation via Pd(II)-Catalyzed Allylic C–H Activation”, *Journal of the American Chemical Society* **130**, (2008): 12901–12903.

99. J. Lu, Y. Yao, L. Li, and N. Fu, “Dual Transition Metal Electrocatalysis: Direct Decarboxylative Alkenylation of Aliphatic Carboxylic Acids”, *Journal of the American Chemical Society* **145**, (2023): 26774–26782.

100. F.-P. Wu, D. Li, J.-B. Peng, and X.-F. Wu, “Carbonylative Transformation of Allylarenes with CO Surrogates: Tunable Synthesis of 4-Arylbutanoic Acids, 2-Arylbutanoic Acids, and 4-Arylbutanals”, *Organic Letters* **21**, (2019): 5699–5703.

101. H. Qrareya, C. Raviola, S. Protti, M. Fagnoni, and A. Albini, “Transition-Metal-Free Arylations via Photogenerated Triplet 4-Alkyl- and 4-Trimethylsilylphenyl Cations”, *Journal of Organic Chemistry* **78**, (2013): 6016–6024.

102. M. O. Akram, P. S. Mali, and N. T. Patil, “Cross-Coupling Reactions of Aryldiazonium Salts with Allylsilanes under Merged Gold/Visible-Light Photoredox Catalysis”, *Organic Letters* **19**, (2017): 3075–3078.

103. J. Albarrán-Velo, V. Gotor-Fernández, and I. Lavandera, “Markovnikov Wacker-Tsuji Oxidation of Allyl(hetero)arenes and Application in a One-Pot Photo-Metal-Biocatalytic Approach to Enantioenriched Amines and Alcohols”, *Advanced Synthesis & Catalysis* **363**, (2021): 4096–4108.

104. S. Doobary, A. T. Sedikides, H. P. Caldora, D. L. Poole, and A. J. J. Lennox, “Electrochemical Vicinal Difluorination of Alkenes: Scalable and Amenable to Electron-Rich Substrates”, *Angewandte Chemie International Edition* **59**, (2020): 1155–1160.

105. Y. Ikenoue, Y. Saida, M.-A. Kira, H. Tomozawa, H. Yashima, and M. Kobayashi, “A facile preparation of a self-doped conducting polymer”, *Journal of the Chemical Society, Chemical Communications* **26**, (1990): 1694–1695.

106. K. Kubota, P. Dai, B. L. Pentelute, and S. L. Buchwald, “Palladium Oxidative Addition Complexes for Peptide and Protein Cross-linking”, *Journal of the American Chemical Society* **140**, (2018): 3128–3133.

107. S. K. Bhunia, P. Das, S. Nandi, and R. Jana, “Carboxylation of Aryl Triflates with CO_2 Merging Palladium and Visible-Light-Photoredox Catalysts”, *Organic Letters* **21**, (2019): 4632–4637.

108. P. M. MacQueen, R. Holley, S. Ghorai, and T. J. Colacot, “Convenient One-Pot Synthesis of $L_2Pd(0)$ Complexes for Cross-Coupling Catalysis”, *Organometallics* **42**, (2023): 2644–2650.
